# THE CONCISE GUIDE TO PHARMACOLOGY 2019/20: Enzymes

**DOI:** 10.1111/bph.14752

**Published:** 2019-11-11

**Authors:** Stephen PH Alexander, Doriano Fabbro, Eamonn Kelly, Alistair Mathie, John A Peters, Emma L Veale, Jane F Armstrong, Elena Faccenda, Simon D Harding, Adam J Pawson, Joanna L Sharman, Christopher Southan, Jamie A Davies, CGTP Collaborators, Annie Beuve, Detlev Boison, Peter Brouckaert, John C Burnett, Kathryn Burns, Carmen Dessauer, Andreas Friebe, John Garthwaite, Jürg Gertsch, Nuala Helsby, Angelo A Izzo, Doris Koesling, Michaela Kuhn, Rennolds Ostrom, Andreas Papapetropoulos, Lincoln R Potter, Nigel J Pyne, Susan Pyne, Michael Russwurm, Harald HHW Schmidt, Roland Seifert, Johannes‐Peter Stasch, Csaba Szabo, Mario van der Stelt, Albert van der Vliet, Val Watts

**Affiliations:** ^1^ School Sciences Nottingham Medical School Nottingham NG7 2UH UK; ^2^ PIQUR Therapeutics Basel 4057 Switzerland; ^3^ School of Physiology, Pharmacology and Neuroscience University of Bristol Bristol BS8 1TD UK; ^4^ Medway School of Pharmacy The Universities of Greenwich and Kent at Medway Anson Building, Central Avenue, Chatham Maritime Chatham Kent ME4 4TB UK; ^5^ Neuroscience Division Medical Education Institute, Ninewells Hospital and Medical School, University of Dundee Dundee DD1 9SY UK; ^6^ Centre for Discovery Brain Sciences University of Edinburgh Edinburgh EH8 9XD UK; ^7^ New Jersey USA; ^8^ Portland USA; ^9^ Ghent Belgium; ^10^ Rochester USA; ^11^ Auckland New Zealand; ^12^ Houston USA; ^13^ Wurzburg Germany; ^14^ London UK; ^15^ Bern Switzerland; ^16^ Naples Italy; ^17^ Bochum Germany; ^18^ Irvine USA; ^19^ Athens Greece; ^20^ Minneapolis USA; ^21^ Glasgow UK; ^22^ Maastricht Netherlands; ^23^ Hannover Germany; ^24^ Rome Italy; ^25^ Galveston USA; ^26^ Leiden The Netherlands; ^27^ Burlington USA; ^28^ West Lafayette USA

## Abstract

The Concise Guide to PHARMACOLOGY 2019/20 is the fourth in this series of biennial publications. The Concise Guide provides concise overviews of the key properties of nearly 1800 human drug targets with an emphasis on selective pharmacology (where available), plus links to the open access knowledgebase source of drug targets and their ligands (http://www.guidetopharmacology.org), which provides more detailed views of target and ligand properties. Although the Concise Guide represents approximately 400 pages, the material presented is substantially reduced compared to information and links presented on the website. It provides a permanent, citable, point‐in‐time record that will survive database updates. The full contents of this section can be found at http://onlinelibrary.wiley.com/doi/10.1111/bph.14752. Enzymes are one of the six major pharmacological targets into which the Guide is divided, with the others being: G protein‐coupled receptors, ion channels, nuclear hormone receptors, catalytic receptors and transporters. These are presented with nomenclature guidance and summary information on the best available pharmacological tools, alongside key references and suggestions for further reading. The landscape format of the Concise Guide is designed to facilitate comparison of related targets from material contemporary to mid‐2019, and supersedes data presented in the 2017/18, 2015/16 and 2013/14 Concise Guides and previous Guides to Receptors and Channels. It is produced in close conjunction with the International Union of Basic and Clinical Pharmacology Committee on Receptor Nomenclature and Drug Classification (NC‐IUPHAR), therefore, providing official IUPHAR classification and nomenclature for human drug targets, where appropriate.

1

### Conflict of interest

The authors state that there are no conflicts of interest to disclose.

### Overview

Enzymes are protein catalysts facilitating the conversion of substrates into products. The Nomenclature Committee of the International Union of Biochemistry and Molecular Biology (NC‐IUBMB) classifies enzymes into families, using a four number code, on the basis of the reactions they catalyse. There are six main families:

EC 1.‐.‐.‐ Oxidoreductases;

EC 2.‐.‐.‐ Transferases;

EC 3.‐.‐.‐ Hydrolases;

EC 4.‐.‐.‐ Lyases;

EC 5.‐.‐.‐ Isomerases;

EC 6.‐.‐.‐ Ligases.

Although there are many more enzymes than receptors in biology, and many drugs that target prokaryotic enzymes are effective medicines, overall the number of enzyme drug targets is relatively small [http://www.ncbi.nlm.nih.gov/pubmed/17139284?dopt=AbstractPlus, http://www.ncbi.nlm.nih.gov/pubmed/24016212?dopt=AbstractPlus], which is not to say that they are of modest importance.

The majority of drugs which act on enzymes act as inhibitors; one exception is metformin, which appears to stimulate activity of AMP‐activated protein kinase, albeit through an imprecisely‐defined mechanism. Kinetic assays allow discrimination of competitive, non‐competitive, and un‐competitive inhibitors. The majority of inhibitors are competitive (acting at the enzyme's ligand recognition site), non‐competitive (acting at a distinct site; potentially interfering with co‐factor or co‐enzyme binding) or of mixed type. One rare example of an uncompetitive inhibitor is lithium ions, which are effective inhibitors at inositol monophosphatase only in the presence of high substrate concentrations. Some inhibitors are irreversible, including a group known as suicide substrates, which bind to the ligand recognition site and then couple covalently to the enzyme. It is beyond the scope of the Guide to give mechanistic information about the inhibitors described, although generally this information is available from the indicated literature.

Many enzymes require additional entities for functional activity. Some of these are used in the catalytic steps, while others promote a particular conformational change. Co‐factors are tightly bound to the enzyme and include metal ions and heme groups. Co‐enzymes are typically small molecules which accept or donate functional groups to assist in the enzymatic reaction. Examples include ATP, NAD, NADP and S‐adenosylmethionine, as well as a number of vitamins, such as riboflavin (vitamin B1) and thiamine (vitamin B2). Where co‐factors/co‐enzymes have been identified, the Guide indicates their involvement.

### Family structure

– http://www.guidetopharmacology.org/GRAC/FamilyDisplayForward?familyId=933

S301 Acetylcholine turnover

S302 Adenosine turnover

S303 Amino acid hydroxylases

S304 L‐Arginine turnover

S304 2.1.1.‐ Protein arginine N‐methyltransferases

S305 Arginase

S305 Arginine:glycine amidinotransferase

S305 Dimethylarginine dimethylaminohydrolases

S306 Nitric oxide synthases

S307 Carbonic anhydrases

S308 Carboxylases and decarboxylases

S308 Carboxylases

S309 Decarboxylases

S311 Catecholamine turnover

S313 Ceramide turnover

S313 Serine palmitoyltransferase

– http://www.guidetopharmacology.org/GRAC/FamilyDisplayForward?familyId=791

S314 Ceramide synthase

S314 Sphingolipid ∆^4^‐desaturase

S315 Sphingomyelin synthase

S315 Sphingomyelin phosphodiesterase

S316 Neutral sphingomyelinase coupling factors

S316 Ceramide glucosyltransferase

S316 Acid ceramidase

S317 Neutral ceramidases

S317 Alkaline ceramidases

S318 Ceramide kinase

– http://www.guidetopharmacology.org/GRAC/FamilyDisplayForward?familyId=981

S319 Chromatin modifying enzymes

– http://www.guidetopharmacology.org/GRAC/FamilyDisplayForward?familyId=869

S319 2.1.1.‐ Protein arginine N‐methyltransferases

– http://www.guidetopharmacology.org/GRAC/FamilyDisplayForward?familyId=871

– http://www.guidetopharmacology.org/GRAC/FamilyDisplayForward?familyId=872

S320 3.5.1.‐ Histone deacetylases (HDACs)

– http://www.guidetopharmacology.org/GRAC/FamilyDisplayForward?familyId=873

– http://www.guidetopharmacology.org/GRAC/FamilyDisplayForward?familyId=884

– http://www.guidetopharmacology.org/GRAC/FamilyDisplayForward?familyId=558

– http://www.guidetopharmacology.org/GRAC/FamilyDisplayForward?familyId=626

– http://www.guidetopharmacology.org/GRAC/FamilyDisplayForward?familyId=631

S321 Cyclic nucleotide turnover/signalling

S321 Adenylyl cyclases (ACs)

S323 Exchange protein activated by cyclic AMP (EPACs)

S323 Phosphodiesterases, 3’,5’‐cyclic nucleotide (PDEs)

S327 Cytochrome P450

S327 CYP1 family

S328 CYP2 family

S329 CYP3 family

S330 CYP4 family

S331 CYP5, CYP7 and CYP8 families

S332 CYP11, CYP17, CYP19, CYP20 and CYP21 families

S333 CYP24, CYP26 and CYP27 families

S333 CYP39, CYP46 and CYP51 families

– http://www.guidetopharmacology.org/GRAC/FamilyDisplayForward?familyId=1020

S334 DNA topoisomerases

S335 Endocannabinoid turnover

S336 *N*‐Acylethanolamine turnover

S337 2‐Acylglycerol ester turnover

S338 Eicosanoid turnover

S338 Cyclooxygenase

S339 Prostaglandin synthases

S341 Lipoxygenases

S342 Leukotriene and lipoxin metabolism

S343 GABA turnover

S344 Glycerophospholipid turnover

S344 Phosphoinositide‐specific phospholipase C

S346 Phospholipase A_2_

S348 Phosphatidylcholine‐specific phospholipase D

S349 Lipid phosphate phosphatases

S349 Phosphatidylinositol kinases

S350 1‐phosphatidylinositol 4‐kinase family

S351 Phosphatidylinositol‐4‐phosphate 3‐kinase family

S351 Phosphatidylinositol 3‐kinase family

S351 Phosphatidylinositol‐4,5‐bisphosphate 3‐kinase family

S352 1‐phosphatidylinositol‐3‐phosphate 5‐kinase family

S353 Type I PIP kinases (1‐phosphatidylinositol‐4‐phosphate 5‐kinase family)

S353 Type II PIP kinases (1‐phosphatidylinositol‐5‐phosphate 4‐kinase family)

S354 Sphingosine kinase

S356 Phosphatidylinositol phosphate kinases

S356 Haem oxygenase

S358 Hydrogen sulphide synthesis

S358 Hydrolases

S360 Inositol phosphate turnover

S360 Inositol 1,4,5‐trisphosphate 3‐kinases

S360 Inositol polyphosphate phosphatases

S361 Inositol monophosphatase

– http://www.guidetopharmacology.org/GRAC/FamilyDisplayForward?familyId=1004

S361 Kinases (EC 2.7.x.x)

– http://www.guidetopharmacology.org/GRAC/FamilyDisplayForward?familyId=677

– http://www.guidetopharmacology.org/GRAC/FamilyDisplayForward?familyId=454

– http://www.guidetopharmacology.org/GRAC/FamilyDisplayForward?familyId=500

– http://www.guidetopharmacology.org/GRAC/FamilyDisplayForward?familyId=573

S362 Rho kinase

– http://www.guidetopharmacology.org/GRAC/FamilyDisplayForward?familyId=283

– http://www.guidetopharmacology.org/GRAC/FamilyDisplayForward?familyId=507

– http://www.guidetopharmacology.org/GRAC/FamilyDisplayForward?familyId=508

– http://www.guidetopharmacology.org/GRAC/FamilyDisplayForward?familyId=937

– http://www.guidetopharmacology.org/GRAC/FamilyDisplayForward?familyId=583

– http://www.guidetopharmacology.org/GRAC/FamilyDisplayForward?familyId=587

– http://www.guidetopharmacology.org/GRAC/FamilyDisplayForward?familyId=604

– http://www.guidetopharmacology.org/GRAC/FamilyDisplayForward?familyId=284

– http://www.guidetopharmacology.org/GRAC/FamilyDisplayForward?familyId=285

S362 Protein kinase C (PKC) family

S363 Alpha subfamily

S363 Delta subfamily

S364 Eta subfamily

– http://www.guidetopharmacology.org/GRAC/FamilyDisplayForward?familyId=535

– http://www.guidetopharmacology.org/GRAC/FamilyDisplayForward?familyId=287

– http://www.guidetopharmacology.org/GRAC/FamilyDisplayForward?familyId=601

– http://www.guidetopharmacology.org/GRAC/FamilyDisplayForward?familyId=466

– http://www.guidetopharmacology.org/GRAC/FamilyDisplayForward?familyId=539

– http://www.guidetopharmacology.org/GRAC/FamilyDisplayForward?familyId=540

– http://www.guidetopharmacology.org/GRAC/FamilyDisplayForward?familyId=541

– http://www.guidetopharmacology.org/GRAC/FamilyDisplayForward?familyId=705

– http://www.guidetopharmacology.org/GRAC/FamilyDisplayForward?familyId=614

– http://www.guidetopharmacology.org/GRAC/FamilyDisplayForward?familyId=616

– http://www.guidetopharmacology.org/GRAC/FamilyDisplayForward?familyId=644

– http://www.guidetopharmacology.org/GRAC/FamilyDisplayForward?familyId=678

– http://www.guidetopharmacology.org/GRAC/FamilyDisplayForward?familyId=448

– http://www.guidetopharmacology.org/GRAC/FamilyDisplayForward?familyId=468

– http://www.guidetopharmacology.org/GRAC/FamilyDisplayForward?familyId=469

– http://www.guidetopharmacology.org/GRAC/FamilyDisplayForward?familyId=450

– http://www.guidetopharmacology.org/GRAC/FamilyDisplayForward?familyId=471

– http://www.guidetopharmacology.org/GRAC/FamilyDisplayForward?familyId=472

– http://www.guidetopharmacology.org/GRAC/FamilyDisplayForward?familyId=556

– http://www.guidetopharmacology.org/GRAC/FamilyDisplayForward?familyId=874

– http://www.guidetopharmacology.org/GRAC/FamilyDisplayForward?familyId=558

– http://www.guidetopharmacology.org/GRAC/FamilyDisplayForward?familyId=576

– http://www.guidetopharmacology.org/GRAC/FamilyDisplayForward?familyId=463

S264 FRAP subfamily

– http://www.guidetopharmacology.org/GRAC/FamilyDisplayForward?familyId=528

– http://www.guidetopharmacology.org/GRAC/FamilyDisplayForward?familyId=530

– http://www.guidetopharmacology.org/GRAC/FamilyDisplayForward?familyId=531

– http://www.guidetopharmacology.org/GRAC/FamilyDisplayForward?familyId=598

– http://www.guidetopharmacology.org/GRAC/FamilyDisplayForward?familyId=465

– http://www.guidetopharmacology.org/GRAC/FamilyDisplayForward?familyId=536

– http://www.guidetopharmacology.org/GRAC/FamilyDisplayForward?familyId=537

– http://www.guidetopharmacology.org/GRAC/FamilyDisplayForward?familyId=538

– http://www.guidetopharmacology.org/GRAC/FamilyDisplayForward?familyId=596

– http://www.guidetopharmacology.org/GRAC/FamilyDisplayForward?familyId=608

– http://www.guidetopharmacology.org/GRAC/FamilyDisplayForward?familyId=626

– http://www.guidetopharmacology.org/GRAC/FamilyDisplayForward?familyId=631

– http://www.guidetopharmacology.org/GRAC/FamilyDisplayForward?familyId=679

– http://www.guidetopharmacology.org/GRAC/FamilyDisplayForward?familyId=561

– http://www.guidetopharmacology.org/GRAC/FamilyDisplayForward?familyId=562

– http://www.guidetopharmacology.org/GRAC/FamilyDisplayForward?familyId=452

– http://www.guidetopharmacology.org/GRAC/FamilyDisplayForward?familyId=474

– http://www.guidetopharmacology.org/GRAC/FamilyDisplayForward?familyId=475

– http://www.guidetopharmacology.org/GRAC/FamilyDisplayForward?familyId=476

– http://www.guidetopharmacology.org/GRAC/FamilyDisplayForward?familyId=477

– http://www.guidetopharmacology.org/GRAC/FamilyDisplayForward?familyId=478

– http://www.guidetopharmacology.org/GRAC/FamilyDisplayForward?familyId=479

– http://www.guidetopharmacology.org/GRAC/FamilyDisplayForward?familyId=480

– http://www.guidetopharmacology.org/GRAC/FamilyDisplayForward?familyId=700

– http://www.guidetopharmacology.org/GRAC/FamilyDisplayForward?familyId=481

– http://www.guidetopharmacology.org/GRAC/FamilyDisplayForward?familyId=482

– http://www.guidetopharmacology.org/GRAC/FamilyDisplayForward?familyId=483

– http://www.guidetopharmacology.org/GRAC/FamilyDisplayForward?familyId=484

– http://www.guidetopharmacology.org/GRAC/FamilyDisplayForward?familyId=563

– http://www.guidetopharmacology.org/GRAC/FamilyDisplayForward?familyId=566

– http://www.guidetopharmacology.org/GRAC/FamilyDisplayForward?familyId=571

– http://www.guidetopharmacology.org/GRAC/FamilyDisplayForward?familyId=572

– http://www.guidetopharmacology.org/GRAC/FamilyDisplayForward?familyId=459

– http://www.guidetopharmacology.org/GRAC/FamilyDisplayForward?familyId=512

– http://www.guidetopharmacology.org/GRAC/FamilyDisplayForward?familyId=513

– http://www.guidetopharmacology.org/GRAC/FamilyDisplayForward?familyId=585

– http://www.guidetopharmacology.org/GRAC/FamilyDisplayForward?familyId=599

– http://www.guidetopharmacology.org/GRAC/FamilyDisplayForward?familyId=600

– http://www.guidetopharmacology.org/GRAC/FamilyDisplayForward?familyId=605

– http://www.guidetopharmacology.org/GRAC/FamilyDisplayForward?familyId=607

– http://www.guidetopharmacology.org/GRAC/FamilyDisplayForward?familyId=609

– http://www.guidetopharmacology.org/GRAC/FamilyDisplayForward?familyId=630

– http://www.guidetopharmacology.org/GRAC/FamilyDisplayForward?familyId=635

– http://www.guidetopharmacology.org/GRAC/FamilyDisplayForward?familyId=636

– http://www.guidetopharmacology.org/GRAC/FamilyDisplayForward?familyId=680

– http://www.guidetopharmacology.org/GRAC/FamilyDisplayForward?familyId=564

– http://www.guidetopharmacology.org/GRAC/FamilyDisplayForward?familyId=627

– http://www.guidetopharmacology.org/GRAC/FamilyDisplayForward?familyId=640

– http://www.guidetopharmacology.org/GRAC/FamilyDisplayForward?familyId=681

– http://www.guidetopharmacology.org/GRAC/FamilyDisplayForward?familyId=568

S365 Cyclin‐dependent kinase (CDK) family

– http://www.guidetopharmacology.org/GRAC/FamilyDisplayForward?familyId=485

– http://www.guidetopharmacology.org/GRAC/FamilyDisplayForward?familyId=492

S365 CDK4 subfamily

– http://www.guidetopharmacology.org/GRAC/FamilyDisplayForward?familyId=495

– http://www.guidetopharmacology.org/GRAC/FamilyDisplayForward?familyId=496

– http://www.guidetopharmacology.org/GRAC/FamilyDisplayForward?familyId=497

– http://www.guidetopharmacology.org/GRAC/FamilyDisplayForward?familyId=489

– http://www.guidetopharmacology.org/GRAC/FamilyDisplayForward?familyId=494

– http://www.guidetopharmacology.org/GRAC/FamilyDisplayForward?familyId=490

– http://www.guidetopharmacology.org/GRAC/FamilyDisplayForward?familyId=498

– http://www.guidetopharmacology.org/GRAC/FamilyDisplayForward?familyId=491

– http://www.guidetopharmacology.org/GRAC/FamilyDisplayForward?familyId=570

– http://www.guidetopharmacology.org/GRAC/FamilyDisplayForward?familyId=455

– http://www.guidetopharmacology.org/GRAC/FamilyDisplayForward?familyId=503

– http://www.guidetopharmacology.org/GRAC/FamilyDisplayForward?familyId=504

– http://www.guidetopharmacology.org/GRAC/FamilyDisplayForward?familyId=505

– http://www.guidetopharmacology.org/GRAC/FamilyDisplayForward?familyId=506

– http://www.guidetopharmacology.org/GRAC/FamilyDisplayForward?familyId=457

S366 GSK subfamily

– http://www.guidetopharmacology.org/GRAC/FamilyDisplayForward?familyId=288

– http://www.guidetopharmacology.org/GRAC/FamilyDisplayForward?familyId=514

– http://www.guidetopharmacology.org/GRAC/FamilyDisplayForward?familyId=515

– http://www.guidetopharmacology.org/GRAC/FamilyDisplayForward?familyId=518

– http://www.guidetopharmacology.org/GRAC/FamilyDisplayForward?familyId=519

– http://www.guidetopharmacology.org/GRAC/FamilyDisplayForward?familyId=520

– http://www.guidetopharmacology.org/GRAC/FamilyDisplayForward?familyId=612

– http://www.guidetopharmacology.org/GRAC/FamilyDisplayForward?familyId=620

– http://www.guidetopharmacology.org/GRAC/FamilyDisplayForward?familyId=682

– http://www.guidetopharmacology.org/GRAC/FamilyDisplayForward?familyId=683

– http://www.guidetopharmacology.org/GRAC/FamilyDisplayForward?familyId=451

– http://www.guidetopharmacology.org/GRAC/FamilyDisplayForward?familyId=473

– http://www.guidetopharmacology.org/GRAC/FamilyDisplayForward?familyId=557

– http://www.guidetopharmacology.org/GRAC/FamilyDisplayForward?familyId=559

– http://www.guidetopharmacology.org/GRAC/FamilyDisplayForward?familyId=560

– http://www.guidetopharmacology.org/GRAC/FamilyDisplayForward?familyId=565

– http://www.guidetopharmacology.org/GRAC/FamilyDisplayForward?familyId=567

– http://www.guidetopharmacology.org/GRAC/FamilyDisplayForward?familyId=577

– http://www.guidetopharmacology.org/GRAC/FamilyDisplayForward?familyId=578

– http://www.guidetopharmacology.org/GRAC/FamilyDisplayForward?familyId=580

– http://www.guidetopharmacology.org/GRAC/FamilyDisplayForward?familyId=584

– http://www.guidetopharmacology.org/GRAC/FamilyDisplayForward?familyId=586

– http://www.guidetopharmacology.org/GRAC/FamilyDisplayForward?familyId=588

– http://www.guidetopharmacology.org/GRAC/FamilyDisplayForward?familyId=589

– http://www.guidetopharmacology.org/GRAC/FamilyDisplayForward?familyId=590

– http://www.guidetopharmacology.org/GRAC/FamilyDisplayForward?familyId=591

– http://www.guidetopharmacology.org/GRAC/FamilyDisplayForward?familyId=592

– http://www.guidetopharmacology.org/GRAC/FamilyDisplayForward?familyId=593

– http://www.guidetopharmacology.org/GRAC/FamilyDisplayForward?familyId=594

– http://www.guidetopharmacology.org/GRAC/FamilyDisplayForward?familyId=595

S367 Polo‐like kinase (PLK) family

– http://www.guidetopharmacology.org/GRAC/FamilyDisplayForward?familyId=462

– http://www.guidetopharmacology.org/GRAC/FamilyDisplayForward?familyId=526

– http://www.guidetopharmacology.org/GRAC/FamilyDisplayForward?familyId=527

– http://www.guidetopharmacology.org/GRAC/FamilyDisplayForward?familyId=597

– http://www.guidetopharmacology.org/GRAC/FamilyDisplayForward?familyId=617

– http://www.guidetopharmacology.org/GRAC/FamilyDisplayForward?familyId=618

– http://www.guidetopharmacology.org/GRAC/FamilyDisplayForward?familyId=628

– http://www.guidetopharmacology.org/GRAC/FamilyDisplayForward?familyId=633

– http://www.guidetopharmacology.org/GRAC/FamilyDisplayForward?familyId=634

– http://www.guidetopharmacology.org/GRAC/FamilyDisplayForward?familyId=637

– http://www.guidetopharmacology.org/GRAC/FamilyDisplayForward?familyId=639

– http://www.guidetopharmacology.org/GRAC/FamilyDisplayForward?familyId=641

– http://www.guidetopharmacology.org/GRAC/FamilyDisplayForward?familyId=642

– http://www.guidetopharmacology.org/GRAC/FamilyDisplayForward?familyId=643

– http://www.guidetopharmacology.org/GRAC/FamilyDisplayForward?familyId=684

– http://www.guidetopharmacology.org/GRAC/FamilyDisplayForward?familyId=449

– http://www.guidetopharmacology.org/GRAC/FamilyDisplayForward?familyId=470

– http://www.guidetopharmacology.org/GRAC/FamilyDisplayForward?familyId=615

– http://www.guidetopharmacology.org/GRAC/FamilyDisplayForward?familyId=890

– http://www.guidetopharmacology.org/GRAC/FamilyDisplayForward?familyId=685

S367 STE7 family

– http://www.guidetopharmacology.org/GRAC/FamilyDisplayForward?familyId=621

– http://www.guidetopharmacology.org/GRAC/FamilyDisplayForward?familyId=467

– http://www.guidetopharmacology.org/GRAC/FamilyDisplayForward?familyId=542

– http://www.guidetopharmacology.org/GRAC/FamilyDisplayForward?familyId=543

– http://www.guidetopharmacology.org/GRAC/FamilyDisplayForward?familyId=544

– http://www.guidetopharmacology.org/GRAC/FamilyDisplayForward?familyId=544

– http://www.guidetopharmacology.org/GRAC/FamilyDisplayForward?familyId=546

– http://www.guidetopharmacology.org/GRAC/FamilyDisplayForward?familyId=547

– http://www.guidetopharmacology.org/GRAC/FamilyDisplayForward?familyId=548

– http://www.guidetopharmacology.org/GRAC/FamilyDisplayForward?familyId=549

– http://www.guidetopharmacology.org/GRAC/FamilyDisplayForward?familyId=622

– http://www.guidetopharmacology.org/GRAC/FamilyDisplayForward?familyId=550

– http://www.guidetopharmacology.org/GRAC/FamilyDisplayForward?familyId=551

– http://www.guidetopharmacology.org/GRAC/FamilyDisplayForward?familyId=552

– http://www.guidetopharmacology.org/GRAC/FamilyDisplayForward?familyId=624

– http://www.guidetopharmacology.org/GRAC/FamilyDisplayForward?familyId=686

– http://www.guidetopharmacology.org/GRAC/FamilyDisplayForward?familyId=936

S368 Abl family

S368 Ack family

– http://www.guidetopharmacology.org/GRAC/FamilyDisplayForward?familyId=569

– http://www.guidetopharmacology.org/GRAC/FamilyDisplayForward?familyId=574

– http://www.guidetopharmacology.org/GRAC/FamilyDisplayForward?familyId=575

S369 Janus kinase (JakA) family

S369 Src family

– http://www.guidetopharmacology.org/GRAC/FamilyDisplayForward?familyId=625

S370 Tec family

– http://www.guidetopharmacology.org/GRAC/FamilyDisplayForward?familyId=687

– http://www.guidetopharmacology.org/GRAC/FamilyDisplayForward?familyId=579

– http://www.guidetopharmacology.org/GRAC/FamilyDisplayForward?familyId=582

– http://www.guidetopharmacology.org/GRAC/FamilyDisplayForward?familyId=458

– http://www.guidetopharmacology.org/GRAC/FamilyDisplayForward?familyId=510

– http://www.guidetopharmacology.org/GRAC/FamilyDisplayForward?familyId=511

– http://www.guidetopharmacology.org/GRAC/FamilyDisplayForward?familyId=461

– http://www.guidetopharmacology.org/GRAC/FamilyDisplayForward?familyId=521

– http://www.guidetopharmacology.org/GRAC/FamilyDisplayForward?familyId=522

– http://www.guidetopharmacology.org/GRAC/FamilyDisplayForward?familyId=523

– http://www.guidetopharmacology.org/GRAC/FamilyDisplayForward?familyId=524

– http://www.guidetopharmacology.org/GRAC/FamilyDisplayForward?familyId=525

S371 RAF family

– http://www.guidetopharmacology.org/GRAC/FamilyDisplayForward?familyId=613

– http://www.guidetopharmacology.org/GRAC/FamilyDisplayForward?familyId=632

S372 Lanosterol biosynthesis pathway

– http://www.guidetopharmacology.org/GRAC/FamilyDisplayForward?familyId=928

– http://www.guidetopharmacology.org/GRAC/FamilyDisplayForward?familyId=993

S374 Nucleoside synthesis and metabolism

S376 Paraoxonase (PON) family

S377 Peptidases and proteinases

– http://www.guidetopharmacology.org/GRAC/FamilyDisplayForward?familyId=707

S377 A1: Pepsin

– http://www.guidetopharmacology.org/GRAC/FamilyDisplayForward?familyId=708

S377 A22: Presenilin

– http://www.guidetopharmacology.org/GRAC/FamilyDisplayForward?familyId=709

– http://www.guidetopharmacology.org/GRAC/FamilyDisplayForward?familyId=728

– http://www.guidetopharmacology.org/GRAC/FamilyDisplayForward?familyId=731

– http://www.guidetopharmacology.org/GRAC/FamilyDisplayForward?familyId=729

– http://www.guidetopharmacology.org/GRAC/FamilyDisplayForward?familyId=730

– http://www.guidetopharmacology.org/GRAC/FamilyDisplayForward?familyId=732

– http://www.guidetopharmacology.org/GRAC/FamilyDisplayForward?familyId=931

– http://www.guidetopharmacology.org/GRAC/FamilyDisplayForward?familyId=711

– http://www.guidetopharmacology.org/GRAC/FamilyDisplayForward?familyId=733

S378 C14: Caspase

– http://www.guidetopharmacology.org/GRAC/FamilyDisplayForward?familyId=711

– http://www.guidetopharmacology.org/GRAC/FamilyDisplayForward?familyId=735

– http://www.guidetopharmacology.org/GRAC/FamilyDisplayForward?familyId=712

– http://www.guidetopharmacology.org/GRAC/FamilyDisplayForward?familyId=736

– http://www.guidetopharmacology.org/GRAC/FamilyDisplayForward?familyId=713

S378 M1: Aminopeptidase N

S379 M2: Angiotensin‐converting (ACE and ACE2)

S379 M10: Matrix metallopeptidase

S380 M12: Astacin/Adamalysin

– http://www.guidetopharmacology.org/GRAC/FamilyDisplayForward?familyId=740

– http://www.guidetopharmacology.org/GRAC/FamilyDisplayForward?familyId=742

– http://www.guidetopharmacology.org/GRAC/FamilyDisplayForward?familyId=714

– http://www.guidetopharmacology.org/GRAC/FamilyDisplayForward?familyId=743

– http://www.guidetopharmacology.org/GRAC/FamilyDisplayForward?familyId=715

– http://www.guidetopharmacology.org/GRAC/FamilyDisplayForward?familyId=744

– http://www.guidetopharmacology.org/GRAC/FamilyDisplayForward?familyId=716

– http://www.guidetopharmacology.org/GRAC/FamilyDisplayForward?familyId=745

– http://www.guidetopharmacology.org/GRAC/FamilyDisplayForward?familyId=717

– http://www.guidetopharmacology.org/GRAC/FamilyDisplayForward?familyId=746

– http://www.guidetopharmacology.org/GRAC/FamilyDisplayForward?familyId=718

– http://www.guidetopharmacology.org/GRAC/FamilyDisplayForward?familyId=747

– http://www.guidetopharmacology.org/GRAC/FamilyDisplayForward?familyId=860

S380 M28: Aminopeptidase Y

– http://www.guidetopharmacology.org/GRAC/FamilyDisplayForward?familyId=719

S381 M19: Membrane dipeptidase

– http://www.guidetopharmacology.org/GRAC/FamilyDisplayForward?familyId=720

– http://www.guidetopharmacology.org/GRAC/FamilyDisplayForward?familyId=750

– http://www.guidetopharmacology.org/GRAC/FamilyDisplayForward?familyId=721

S381 S1: Chymotrypsin

– http://www.guidetopharmacology.org/GRAC/FamilyDisplayForward?familyId=722

– http://www.guidetopharmacology.org/GRAC/FamilyDisplayForward?familyId=878

S382 T1: Proteasome

– http://www.guidetopharmacology.org/GRAC/FamilyDisplayForward?familyId=753

– http://www.guidetopharmacology.org/GRAC/FamilyDisplayForward?familyId=723

– http://www.guidetopharmacology.org/GRAC/FamilyDisplayForward?familyId=754

– http://www.guidetopharmacology.org/GRAC/FamilyDisplayForward?familyId=724

S382 S8: Subtilisin

– http://www.guidetopharmacology.org/GRAC/FamilyDisplayForward?familyId=725

S383 S9: Prolyl oligopeptidase

– http://www.guidetopharmacology.org/GRAC/FamilyDisplayForward?familyId=756

– http://www.guidetopharmacology.org/GRAC/FamilyDisplayForward?familyId=757

– http://www.guidetopharmacology.org/GRAC/FamilyDisplayForward?familyId=917

– http://www.guidetopharmacology.org/GRAC/FamilyDisplayForward?familyId=947

– http://www.guidetopharmacology.org/GRAC/FamilyDisplayForward?familyId=980

– http://www.guidetopharmacology.org/GRAC/FamilyDisplayForward?familyId=948

S383 Poly ADP‐ribose polymerases

S384 Prolyl hydroxylases

S384 Sphingosine 1‐phosphate turnover

S385 Sphingosine kinase

S386 Sphingosine 1‐phosphate phosphatase

S387 Sphingosine 1‐phosphate lyase

S387 Thyroid hormone turnover

– http://www.guidetopharmacology.org/GRAC/FamilyDisplayForward?familyId=988

– http://www.guidetopharmacology.org/GRAC/FamilyDisplayForward?familyId=840

– http://www.guidetopharmacology.org/GRAC/FamilyDisplayForward?familyId=922

– http://www.guidetopharmacology.org/GRAC/FamilyDisplayForward?familyId=912

– http://www.guidetopharmacology.org/GRAC/FamilyDisplayForward?familyId=899

S388 1.14.13.9 Kynurenine 3‐monooxygenase

– http://www.guidetopharmacology.org/GRAC/FamilyDisplayForward?familyId=877

– http://www.guidetopharmacology.org/GRAC/FamilyDisplayForward?familyId=843

– http://www.guidetopharmacology.org/GRAC/FamilyDisplayForward?familyId=846

– http://www.guidetopharmacology.org/GRAC/FamilyDisplayForward?familyId=844

– http://www.guidetopharmacology.org/GRAC/FamilyDisplayForward?familyId=978

– http://www.guidetopharmacology.org/GRAC/FamilyDisplayForward?familyId=1003

– http://www.guidetopharmacology.org/GRAC/FamilyDisplayForward?familyId=979

– http://www.guidetopharmacology.org/GRAC/FamilyDisplayForward?familyId=908

S389 2.5.1.58 Protein farnesyltransferase

– http://www.guidetopharmacology.org/GRAC/FamilyDisplayForward?familyId=926

– http://www.guidetopharmacology.org/GRAC/FamilyDisplayForward?familyId=994

– http://www.guidetopharmacology.org/GRAC/FamilyDisplayForward?familyId=880

– http://www.guidetopharmacology.org/GRAC/FamilyDisplayForward?familyId=850

– http://www.guidetopharmacology.org/GRAC/FamilyDisplayForward?familyId=841

– http://www.guidetopharmacology.org/GRAC/FamilyDisplayForward?familyId=909

S390 3.5.1.‐ Histone deacetylases (HDACs)

– http://www.guidetopharmacology.org/GRAC/FamilyDisplayForward?familyId=925

S391 3.5.3.15 Peptidyl arginine deiminases (PADI)

S391 3.6.5.2 Small monomeric GTPases

S391 RAS subfamily

S392 RAB subfamily

– http://www.guidetopharmacology.org/GRAC/FamilyDisplayForward?familyId=849

– http://www.guidetopharmacology.org/GRAC/FamilyDisplayForward?familyId=845

– http://www.guidetopharmacology.org/GRAC/FamilyDisplayForward?familyId=847

## http://www.guidetopharmacology.org/GRAC/FamilyDisplayForward?familyId=765

### Overview

Acetylcholine is familiar as a neurotransmitter in the central nervous system and in the periphery. In the somatic nervous system, it activates http://www.guidetopharmacology.org/GRAC/FamilyDisplayForward?familyId=76 at the skeletal neuromuscular junction. It is also employed in the autonomic nervous system, in both parasympathetic and sympathetic branches; in the former, at the smooth muscle neuromuscular junction, activating http://www.guidetopharmacology.org/GRAC/FamilyDisplayForward?familyId=2. In the latter, acetylcholine is involved as a neurotransmitter at the ganglion, activating nicotinic acetylcholine receptors. Acetylcholine is synthesised in neurones through the action of choline O‐acetyltransferase and metabolised after release through the extracellular action of acetylcholinesterase and cholinesterase. Choline is accumulated from the extracellular medium by selective transporters (see http://www.guidetopharmacology.org/GRAC/FamilyDisplayForward?familyId=143#show_object_914 and the http://www.guidetopharmacology.org/GRAC/FamilyDisplayForward?familyId=233 family). Acetylcholine is accumulated in synaptic vesicles through the action of the vesicular acetylcholine transporter http://www.guidetopharmacology.org/GRAC/FamilyDisplayForward?familyId=193.

**Table:** 

Nomenclature	http://www.guidetopharmacology.org/GRAC/ObjectDisplayForward?objectId=2480	http://www.guidetopharmacology.org/GRAC/ObjectDisplayForward?objectId=2465	http://www.guidetopharmacology.org/GRAC/ObjectDisplayForward?objectId=2471
Common abbreviation	ChAT	AChE	BChE
HGNC, UniProt	https://www.genenames.org/data/gene‐symbol‐report/#!/hgnc_id/HGNC:1912, http://www.uniprot.org/uniprot/P28329	https://www.genenames.org/data/gene‐symbol‐report/#!/hgnc_id/HGNC:108, http://www.uniprot.org/uniprot/P22303	https://www.genenames.org/data/gene‐symbol‐report/#!/hgnc_id/HGNC:983, http://www.uniprot.org/uniprot/P06276
EC number	http://www.genome.jp/dbget‐bin/www_bget?ec:2.3.1.6: http://www.guidetopharmacology.org/GRAC/LigandDisplayForward?ligandId=3038 + http://www.guidetopharmacology.org/GRAC/LigandDisplayForward?ligandId=4551 = http://www.guidetopharmacology.org/GRAC/LigandDisplayForward?ligandId=294 + http://www.guidetopharmacology.org/GRAC/LigandDisplayForward?ligandId=3044	http://www.genome.jp/dbget‐bin/www_bget?ec:3.1.1.7: http://www.guidetopharmacology.org/GRAC/LigandDisplayForward?ligandId=294 + H_2_O = http://www.guidetopharmacology.org/GRAC/LigandDisplayForward?ligandId=1058 + http://www.guidetopharmacology.org/GRAC/LigandDisplayForward?ligandId=4551 + H^+^	http://www.genome.jp/dbget‐bin/www_bget?ec:3.1.1.7: http://www.guidetopharmacology.org/GRAC/LigandDisplayForward?ligandId=294 + H_2_O = http://www.guidetopharmacology.org/GRAC/LigandDisplayForward?ligandId=1058 + http://www.guidetopharmacology.org/GRAC/LigandDisplayForward?ligandId=4551 + H^+^
Inhibitors	http://www.guidetopharmacology.org/GRAC/LigandDisplayForward?ligandId=8807 (pIC50 6.5) [http://www.ncbi.nlm.nih.gov/pubmed/3351860?dopt=AbstractPlus] – Mouse	http://www.guidetopharmacology.org/GRAC/LigandDisplayForward?ligandId=6687 (p*K*_i_ 7.5) [http://www.ncbi.nlm.nih.gov/pubmed/18479118?dopt=AbstractPlus], http://www.guidetopharmacology.org/GRAC/LigandDisplayForward?ligandId=6693 (pIC_50_ 6.3) [http://www.ncbi.nlm.nih.gov/pubmed/12182861?dopt=AbstractPlus], http://www.guidetopharmacology.org/GRAC/LigandDisplayForward?ligandId=6602 (pIC_50_ 5.4) [http://www.ncbi.nlm.nih.gov/pubmed/16570913?dopt=AbstractPlus]	http://www.guidetopharmacology.org/GRAC/LigandDisplayForward?ligandId=6602 (pIC_50_ 7.4) [http://www.ncbi.nlm.nih.gov/pubmed/16570913?dopt=AbstractPlus], http://www.guidetopharmacology.org/GRAC/LigandDisplayForward?ligandId=6687 (p*K*_i_ 7.2) [http://www.ncbi.nlm.nih.gov/pubmed/18479118?dopt=AbstractPlus]
Sub/family‐selective inhibitors	–	http://www.guidetopharmacology.org/GRAC/LigandDisplayForward?ligandId=6598 (pIC_50_ 7.6–7.8) [http://www.ncbi.nlm.nih.gov/pubmed/16570913?dopt=AbstractPlus]	http://www.guidetopharmacology.org/GRAC/LigandDisplayForward?ligandId=6598 (pIC_50_ 7.6–7.8) [http://www.ncbi.nlm.nih.gov/pubmed/16570913?dopt=AbstractPlus]
Selective inhibitors	–	http://www.guidetopharmacology.org/GRAC/LigandDisplayForward?ligandId=6599 (pIC_50_ 7.7–8.3) [http://www.ncbi.nlm.nih.gov/pubmed/1738151?dopt=AbstractPlus, http://www.ncbi.nlm.nih.gov/pubmed/8039548?dopt=AbstractPlus, http://www.ncbi.nlm.nih.gov/pubmed/16570913?dopt=AbstractPlus], http://www.guidetopharmacology.org/GRAC/LigandDisplayForward?ligandId=6600 (pIC_50_ 7.7) [http://www.ncbi.nlm.nih.gov/pubmed/12675140?dopt=AbstractPlus]	http://www.guidetopharmacology.org/GRAC/LigandDisplayForward?ligandId=6601 (pIC_50_ 8.5) [http://www.ncbi.nlm.nih.gov/pubmed/12675140?dopt=AbstractPlus]
Comments	Splice variants of choline O‐acetyltransferase are suggested to be differentially distributed in the periphery and CNS (see [http://www.ncbi.nlm.nih.gov/pubmed/21382474?dopt=AbstractPlus]).	–	–

### Comments

A number of organophosphorus compounds inhibit acetylcholinesterase and cholinesterase irreversibly, including pesticides such as chlorpyrifos‐oxon, and nerve agents such as tabun, soman and sarin. AChE is unusual in its exceptionally high turnover rate which has been calculated at 740 000/min/molecule [http://www.ncbi.nlm.nih.gov/pubmed/13785664?dopt=AbstractPlus].

### Further reading on Acetylcholine turnover

Li Q *et al*. (2017) Recent progress in the identification of selective butyrylcholinesterase inhibitors for Alzheimer's disease. *Eur J Med Chem* **132**: 294–309 https://www.ncbi.nlm.nih.gov/pubmed/28371641?dopt=AbstractPlus

Lockridge O. (2015) Review of human butyrylcholinesterase structure, function, genetic variants, history of use in the clinic, and potential therapeutic uses. *Pharmacol Ther* **148**: 34–46 https://www.ncbi.nlm.nih.gov/pubmed/25448037?dopt=AbstractPlus

Masson P *et al*. (2016) Slow‐binding inhibition of cholinesterases, pharmacological and toxicological relevance. *Arch Biochem Biophys* **593**: 60–8 https://www.ncbi.nlm.nih.gov/pubmed/26874196?dopt=AbstractPlus

Rotundo RL. (2017) Biogenesis, assembly and trafficking of acetylcholinesterase. *J Neurochem* **142 Suppl 2**: 52–58 https://www.ncbi.nlm.nih.gov/pubmed/28326552?dopt=AbstractPlus

Silman I *et al*. (2017) Recent developments in structural studies on acetylcholinesterase. *J Neurochem* **142 Suppl 2**: 19–25 https://www.ncbi.nlm.nih.gov/pubmed/28503857?dopt=AbstractPlus

## http://www.guidetopharmacology.org/GRAC/FamilyDisplayForward?familyId=248

### Overview

A multifunctional, ubiquitous molecule, http://www.guidetopharmacology.org/GRAC/LigandDisplayForward?ligandId=2844 acts at cell‐surface G protein‐coupled receptors, as well as numerous enzymes, including protein kinases and adenylyl cyclase. Ex‐tracellular adenosine is thought to be produced either by export or by metabolism, predominantly through ecto‐5’‐nucleotidase activity (also producing inorganic phosphate). It is inactivated either by extracellular metabolism *via* adenosine deaminase (also producing ammonia) or, following uptake by nucleoside transporters, *via* adenosine deaminase or adenosine kinase (requiring http://www.guidetopharmacology.org/GRAC/LigandDisplayForward?ligandId=1713 as co‐substrate). Intracellular adenosine may be produced by cytosolic 5’‐nucleotidases or through S‐adenosylhomocysteine hydrolase (also producing http://www.guidetopharmacology.org/GRAC/LigandDisplayForward?ligandId=5198).

**Table:** 

Nomenclature	http://www.guidetopharmacology.org/GRAC/ObjectDisplayForward?objectId=1230	http://www.guidetopharmacology.org/GRAC/ObjectDisplayForward?objectId=1231	http://www.guidetopharmacology.org/GRAC/ObjectDisplayForward?objectId=1232	http://www.guidetopharmacology.org/GRAC/ObjectDisplayForward?objectId=1233
Systematic nomenclature	–	–	CD73	–
Common abbreviation	ADA	ADK	NT5E	SAHH
HGNC, UniProt	https://www.genenames.org/data/gene‐symbol‐report/#!/hgnc_id/HGNC:186, http://www.uniprot.org/uniprot/P00813	https://www.genenames.org/data/gene‐symbol‐report/#!/hgnc_id/HGNC:257, http://www.uniprot.org/uniprot/P55263	https://www.genenames.org/data/gene‐symbol‐report/#!/hgnc_id/HGNC:8021, http://www.uniprot.org/uniprot/P21589	https://www.genenames.org/data/gene‐symbol‐report/#!/hgnc_id/HGNC:343, http://www.uniprot.org/uniprot/P23526
EC number	http://www.genome.jp/dbget‐bin/www_bget?ec:3.5.4.4: http://www.guidetopharmacology.org/GRAC/LigandDisplayForward?ligandId=2844 + H_2_O = http://www.guidetopharmacology.org/GRAC/LigandDisplayForward?ligandId=4554 + NH_3_	http://www.genome.jp/dbget‐bin/www_bget?ec:2.7.1.20	http://www.genome.jp/dbget‐bin/www_bget?ec:3.1.3.5	http://www.genome.jp/dbget‐bin/www_bget?ec:3.3.1.1
Rank order of affinity	http://www.guidetopharmacology.org/GRAC/LigandDisplayForward?ligandId=5109 *>* http://www.guidetopharmacology.org/GRAC/LigandDisplayForward?ligandId=2844	http://www.guidetopharmacology.org/GRAC/LigandDisplayForward?ligandId=2844	http://www.guidetopharmacology.org/GRAC/LigandDisplayForward?ligandId=2455, http://www.guidetopharmacology.org/GRAC/LigandDisplayForward?ligandId=5123, http://www.guidetopharmacology.org/GRAC/LigandDisplayForward?ligandId=5124, http://www.guidetopharmacology.org/GRAC/LigandDisplayForward?ligandId=5125 *>* http://www.guidetopharmacology.org/GRAC/LigandDisplayForward?ligandId=5120, http://www.guidetopharmacology.org/GRAC/LigandDisplayForward?ligandId=5122	–
Endogenous substrates	–	–	–	http://www.guidetopharmacology.org/GRAC/LigandDisplayForward?ligandId=5265
Products	http://www.guidetopharmacology.org/GRAC/LigandDisplayForward?ligandId=5110, http://www.guidetopharmacology.org/GRAC/LigandDisplayForward?ligandId=4554	http://www.guidetopharmacology.org/GRAC/LigandDisplayForward?ligandId=2455	http://www.guidetopharmacology.org/GRAC/LigandDisplayForward?ligandId=4566, http://www.guidetopharmacology.org/GRAC/LigandDisplayForward?ligandId=4554, http://www.guidetopharmacology.org/GRAC/LigandDisplayForward?ligandId=4556, http://www.guidetopharmacology.org/GRAC/LigandDisplayForward?ligandId=2844	http://www.guidetopharmacology.org/GRAC/LigandDisplayForward?ligandId=2844
Inhibitors	–	–	–	http://www.guidetopharmacology.org/GRAC/LigandDisplayForward?ligandId=8392 (p*K*_i_ 12.3) [http://www.ncbi.nlm.nih.gov/pubmed/3457563?dopt=AbstractPlus] – Hamster
Selective inhibitors	http://www.guidetopharmacology.org/GRAC/LigandDisplayForward?ligandId=4805 (pIC50 10.8) [http://www.ncbi.nlm.nih.gov/pubmed/849330?dopt=AbstractPlus], http://www.guidetopharmacology.org/GRAC/LigandDisplayForward?ligandId=5179 (p*K*_i_ 8.8) [http://www.ncbi.nlm.nih.gov/pubmed/849330?dopt=AbstractPlus]	http://www.guidetopharmacology.org/GRAC/LigandDisplayForward?ligandId=5130 (pIC_50_ 10.2) [http://www.ncbi.nlm.nih.gov/pubmed/11160637?dopt=AbstractPlus], http://www.guidetopharmacology.org/GRAC/LigandDisplayForward?ligandId=5131 (pIC_50_ 8.8) [http://www.ncbi.nlm.nih.gov/pubmed/11082453?dopt=AbstractPlus]	http://www.guidetopharmacology.org/GRAC/LigandDisplayForward?ligandId=5092 (pIC_50_ 8.7) [http://www.ncbi.nlm.nih.gov/pubmed/1169962?dopt=AbstractPlus]	http://www.guidetopharmacology.org/GRAC/LigandDisplayForward?ligandId=5115 (pIC_50_ 8.5) [http://www.ncbi.nlm.nih.gov/pubmed/7470463?dopt=AbstractPlus]
Comments	–	The enzyme exists in two isoforms derived from alternative splicing of a single gene product: a short isoform, ADK‐S, located in the cytoplasm is responsible for the regulation of intra‐ and extracellular levels of adenosine and hence adenosine receptor activation; a long isoform, ADK‐L, located in the nucleus contributes to the regulation of DNA methylation [http://www.ncbi.nlm.nih.gov/pubmed/23592612?dopt=AbstractPlus, http://www.ncbi.nlm.nih.gov/pubmed/23863710?dopt=AbstractPlus].	Pharmacological inhibition of CD73 is being investigated as a novel cancer immunotherapy strategy [http://www.ncbi.nlm.nih.gov/pubmed/21537079?dopt=AbstractPlus].	–

### Comments

An extracellular adenosine deaminase activity, termed ADA2 or adenosine deaminase growth factor (ADGF, https://www.genenames.org/data/gene‐symbol‐report/#!/hgnc_id/HGNC:1839, http://www.uniprot.org/uniprot/Q9NZK5) has been identified [http://www.ncbi.nlm.nih.gov/pubmed/24933472?dopt=AbstractPlus, http://www.ncbi.nlm.nih.gov/pubmed/16245011?dopt=AbstractPlus], which is insensitive to http://www.guidetopharmacology.org/GRAC/LigandDisplayForward?ligandId=5179 [http://www.ncbi.nlm.nih.gov/pubmed/20147294?dopt=AbstractPlus]. Other forms of adenosine deaminase act on ribonucleic acids and may be divided into two families: https://www.genenames.org/data/gene‐symbol‐report/#!/hgnc_id/HGNC:228 (http://www.uniprot.org/uniprot/Q9BUB4) deaminates transfer RNA; https://www.genenames.org/data/gene‐symbol‐report/#!/hgnc_id/HGNC:225 (http://www.genome.jp/dbget‐bin/www_bget?ec:3.5.4.37, also known as 136 kDa double‐stranded RNA‐binding protein, P136, K88DSRBP, Interferon‐inducible protein 4); https://www.genenames.org/data/gene‐symbol‐report/#!/hgnc_id/HGNC:226 (EC 3.5.‐.‐, , also known as dsRNA adenosine deaminase) andhttps://www.genenames.org/data/gene‐symbol‐report/#!/hgnc_id/HGNC:227 (EC 3.5.‐.‐, also known as dsRNA adenosine deaminase B2, RNA‐dependent adenosine deaminase 3) act on double‐stranded RNA. Particular polymorphisms of the ADA gene result in loss‐of‐function and severe combined immunodeficiency syndrome. Adenosine deaminase is able to complex with dipeptidyl peptidase IV (http://www.genome.jp/dbget‐bin/www_bget?ec:3.4.14.5, https://www.genenames.org/data/gene‐symbol‐report/#!/hgnc_id/HGNC:3009, also known as T‐cell activation antigen CD26, TP103, adenosine deaminase complexing protein 2) to form a cell‐surface activity [http://www.ncbi.nlm.nih.gov/pubmed/8101391?dopt=AbstractPlus].

### Further reading on Adenosine turnover

Boison D. (2016) Adenosinergic signaling in epilepsy. *Neuropharmacology* **104**: 131–9 https://www.ncbi.nlm.nih.gov/pubmed/26341819?dopt=AbstractPlus

Cortés A *et al*. (2015) Moonlighting adenosine deaminase: a target protein for drug development. *Med Res Rev* **35**: 85–125 https://www.ncbi.nlm.nih.gov/pubmed/24933472?dopt=AbstractPlus

Nishikura K. (2016) A‐to‐I editing of coding and non‐coding RNAs by ADARs. *Nat Rev Mol Cell Biol* **17**: 83–96 https://www.ncbi.nlm.nih.gov/pubmed/26648264?dopt=AbstractPlus

Sawynok J. (2016) Adenosine receptor targets for pain. *Neuroscience* **338**: 1–18 https://www.ncbi.nlm.nih.gov/pubmed/26500181?dopt=AbstractPlus

Xiao Y *et al*. (2015) Role of S‐adenosylhomocysteine in cardiovascular disease and its potential epi‐genetic mechanism. *Int. J. Biochem. Cell Biol*. **67**: 158–66 https://www.ncbi.nlm.nih.gov/pubmed/26117455?dopt=AbstractPlus

## http://www.guidetopharmacology.org/GRAC/FamilyDisplayForward?familyId=249

### Overview

The amino acid hydroxylases (monooxygenases), EC.1.14.16.‐, are iron‐containing enzymes which utilise molecular oxygen and http://www.guidetopharmacology.org/GRAC/LigandDisplayForward?ligandId=5276 as co‐substrate and co‐factor, respectively. In humans, as well as in other mammals, there are two distinct L‐Tryptophan hydroxylase 2 genes. In humans, these genes are located on chromosomes 11 and 12 and encode two different homologous enzymes, TPH1 and TPH2.

**Table:** 

Nomenclature	http://www.guidetopharmacology.org/GRAC/ObjectDisplayForward?objectId=1240	http://www.guidetopharmacology.org/GRAC/ObjectDisplayForward?objectId=1243	http://www.guidetopharmacology.org/GRAC/ObjectDisplayForward?objectId=1241	http://www.guidetopharmacology.org/GRAC/ObjectDisplayForward?objectId=1242
HGNC, UniProt	https://www.genenames.org/data/gene‐symbol‐report/#!/hgnc_id/HGNC:8582, http://www.uniprot.org/uniprot/P00439	https://www.genenames.org/data/gene‐symbol‐report/#!/hgnc_id/HGNC:11782, http://www.uniprot.org/uniprot/P07101	https://www.genenames.org/data/gene‐symbol‐report/#!/hgnc_id/HGNC:12008, http://www.uniprot.org/uniprot/P17752	https://www.genenames.org/data/gene‐symbol‐report/#!/hgnc_id/HGNC:20692, http://www.uniprot.org/uniprot/Q8IWU9
EC number	http://www.genome.jp/dbget‐bin/www_bget?ec:1.14.16.1: http://www.guidetopharmacology.org/GRAC/LigandDisplayForward?ligandId=3313 + O_2_ ‐> http://www.guidetopharmacology.org/GRAC/LigandDisplayForward?ligandId=4791	http://www.genome.jp/dbget‐bin/www_bget?ec:1.14.16.2: http://www.guidetopharmacology.org/GRAC/LigandDisplayForward?ligandId=4791 + O_2_ ‐> http://www.guidetopharmacology.org/GRAC/LigandDisplayForward?ligandId=3639	http://www.genome.jp/dbget‐bin/www_bget?ec:1.14.16.4	http://www.genome.jp/dbget‐bin/www_bget?ec:1.14.16.4
Endogenous substrates	http://www.guidetopharmacology.org/GRAC/LigandDisplayForward?ligandId=3313	http://www.guidetopharmacology.org/GRAC/LigandDisplayForward?ligandId=4791	http://www.guidetopharmacology.org/GRAC/LigandDisplayForward?ligandId=717	http://www.guidetopharmacology.org/GRAC/LigandDisplayForward?ligandId=717
Products	http://www.guidetopharmacology.org/GRAC/LigandDisplayForward?ligandId=4791	http://www.guidetopharmacology.org/GRAC/LigandDisplayForward?ligandId=3639	http://www.guidetopharmacology.org/GRAC/LigandDisplayForward?ligandId=4671	http://www.guidetopharmacology.org/GRAC/LigandDisplayForward?ligandId=4671
Cofactors	http://www.guidetopharmacology.org/GRAC/LigandDisplayForward?ligandId=5276	http://www.guidetopharmacology.org/GRAC/LigandDisplayForward?ligandId=5276, Fe^2+^	–	–
Endogenous activators	Protein kinase A‐mediated phosphorylation (Rat) [http://www.ncbi.nlm.nih.gov/pubmed/182695?dopt=AbstractPlus]	Protein kinase A‐mediated phosphorylation [http://www.ncbi.nlm.nih.gov/pubmed/33381?dopt=AbstractPlus]	Protein kinase A‐mediated phosphorylation [http://www.ncbi.nlm.nih.gov/pubmed/8592157?dopt=AbstractPlus]	Protein kinase A‐mediated phosphorylation [http://www.ncbi.nlm.nih.gov/pubmed/8592157?dopt=AbstractPlus]
Inhibitors	–	–	http://www.guidetopharmacology.org/GRAC/LigandDisplayForward?ligandId=9490 [http://www.ncbi.nlm.nih.gov/pubmed/26206858?dopt=AbstractPlus]	–
Selective inhibitors	http://www.guidetopharmacology.org/GRAC/LigandDisplayForward?ligandId=5093 [http://www.ncbi.nlm.nih.gov/pubmed/944951?dopt=AbstractPlus] – Rat, http://www.guidetopharmacology.org/GRAC/LigandDisplayForward?ligandId=5240	http://www.guidetopharmacology.org/GRAC/LigandDisplayForward?ligandId=5095, http://www.guidetopharmacology.org/GRAC/LigandDisplayForward?ligandId=5114, http://www.guidetopharmacology.org/GRAC/LigandDisplayForward?ligandId=5117, http://www.guidetopharmacology.org/GRAC/LigandDisplayForward?ligandId=6956	http://www.guidetopharmacology.org/GRAC/LigandDisplayForward?ligandId=5095, http://www.guidetopharmacology.org/GRAC/LigandDisplayForward?ligandId=5126 [http://www.ncbi.nlm.nih.gov/pubmed/6457252?dopt=AbstractPlus], http://www.guidetopharmacology.org/GRAC/LigandDisplayForward?ligandId=5240, http://www.guidetopharmacology.org/GRAC/LigandDisplayForward?ligandId=4613	http://www.guidetopharmacology.org/GRAC/LigandDisplayForward?ligandId=5095, http://www.guidetopharmacology.org/GRAC/LigandDisplayForward?ligandId=5126 [http://www.ncbi.nlm.nih.gov/pubmed/6457252?dopt=AbstractPlus], http://www.guidetopharmacology.org/GRAC/LigandDisplayForward?ligandId=5240, http://www.guidetopharmacology.org/GRAC/LigandDisplayForward?ligandId=4613
Comments	PAH is an iron bound homodimer or ‐tetramer from the same structural family as tyrosine 3‐monooxygenase and the tryptophan hydroxylases. Deficiency or loss‐of‐function of PAH is associated with http://omim.org/entry/612349	TH is a homotetramer, which is inhibited by dopamine and other catecholamines in a physiological negative feedback pathway [http://www.ncbi.nlm.nih.gov/pubmed/21176768?dopt=AbstractPlus].	–	–

### Further reading on Amino acid hydroxylases

Bauer IE *et al*. (2015) Serotonergic gene variation in substance use pharmacotherapy: a systematic review. *Pharmacogenomics* **16**: 1307–14 https://www.ncbi.nlm.nih.gov/pubmed/26265436?dopt=AbstractPlus

Daubner SC *et al*. (2011) Tyrosine hydroxylase and regulation of dopamine synthesis. *Arch Biochem Biophys* **508**: 1–12 https://www.ncbi.nlm.nih.gov/pubmed/21176768?dopt=AbstractPlus

Flydal MI *et al*. (2013) Phenylalanine hydroxylase: function, structure, and regulation. *IUBMB Life* **65**: 341–9 https://www.ncbi.nlm.nih.gov/pubmed/23457044?dopt=AbstractPlus

Roberts KM *et al*. (2013) Mechanisms of tryptophan and tyrosine hydroxylase. *IUBMB Life* **65**: 350–7 https://www.ncbi.nlm.nih.gov/pubmed/23441081?dopt=AbstractPlus

Tekin I *et al*. (2014) Complex molecular regulation of tyrosine hydroxylase. *J Neural Transm* **121**: 1451–81 https://www.ncbi.nlm.nih.gov/pubmed/24866693?dopt=AbstractPlus

Walen K *et al*. (2017) Tyrosine and tryptophan hydroxylases as therapeutic targets in human disease. *Expert Opin Ther Targets* **21**: 167–180 https://www.ncbi.nlm.nih.gov/pubmed/27973928?dopt=AbstractPlus

## http://www.guidetopharmacology.org/GRAC/FamilyDisplayForward?familyId=239

### Overview

http://www.guidetopharmacology.org/GRAC/LigandDisplayForward?ligandId=721 is a basic amino acid with a guanidino sidechain. As an amino acid, metabolism of L‐arginine to form http://www.guidetopharmacology.org/GRAC/LigandDisplayForward?ligandId=725, catalysed by arginase, forms the last step of the urea production cycle. L‐Ornithine may be utilised as a precursor of polyamines (see http://www.guidetopharmacology.org/GRAC/FamilyDisplayForward?familyId=240) or recycled via http://www.guidetopharmacology.org/GRAC/LigandDisplayForward?ligandId=5324 to L‐arginine. L‐Arginine http://www.guidetopharmacology.org/GRAC/FamilyDisplayForward?familyId=240#Decarboxylases to form http://www.guidetopharmacology.org/GRAC/LigandDisplayForward?ligandId=4127, although the prominence of this pathway in human tissues is uncertain. L‐Arginine may be used as a precursor for http://www.guidetopharmacology.org/GRAC/LigandDisplayForward?ligandId=5325 formation in the http://www.guidetopharmacology.org/GRAC/LigandDisplayForward?ligandId=4496 synthesis pathway under the influence of arginine:glycine amidinotransferase with L‐ornithine as a byproduct. Nitric oxide synthase uses L‐arginine to generate nitric oxide, with http://www.guidetopharmacology.org/GRAC/LigandDisplayForward?ligandId=722 also as a byproduct. L‐Arginine in proteins may be subject to post‐translational modification through methylation, catalysed by protein arginine methyltransferases. Subsequent proteolysis can liberate asymmetric http://www.guidetopharmacology.org/GRAC/LigandDisplayForward?ligandId=5229 (ADMA), which is an endogenous inhibitor of nitric oxide synthase activities. ADMA is hydrolysed by dimethylarginine dimethylhydrolase activities to generate http://www.guidetopharmacology.org/GRAC/LigandDisplayForward?ligandId=722 and http://www.guidetopharmacology.org/GRAC/LigandDisplayForward?ligandId=5177.

## http://www.guidetopharmacology.org/GRAC/FamilyDisplayForward?familyId=254

### Overview

Protein arginine *N*‐methyltransferases (PRMT, EC 2.1.1.‐) encompass histone arginine *N*‐methyltransferases (PRMT4, PRMT7, http://www.genome.jp/kegg‐bin/search_brite?option=‐a&search_string=2.1.1.125) and myelin basic protein *N*‐methyltransferases (PRMT7, http://www.genome.jp/kegg‐bin/search_brite?option=‐a&search_string=2.1.1.126). They are dimeric or tetrameric enzymes which use http://www.guidetopharmacology.org/GRAC/LigandDisplayForward?ligandId=4786 as a methyl donor, generating http://www.guidetopharmacology.org/GRAC/LigandDisplayForward?ligandId=5265 as a by‐product. They generate both mono‐methylated and di‐methylated products; these may be symmetric (http://www.guidetopharmacology.org/GRAC/LigandDisplayForward?ligandId=5271) or asymmetric (http://www.guidetopharmacology.org/GRAC/LigandDisplayForward?ligandId=5229) versions, where both guanidine nitrogens are monomethylated or one of the two is dimethylated, respectively.

Information on members of this family may be found in the http://www.guidetopharmacology.org/GRAC/FamilyDisplayForward?familyId=254.

## http://www.guidetopharmacology.org/GRAC/FamilyDisplayForward?familyId=250

### Overview

Arginase (http://www.genome.jp/kegg‐bin/search_brite?option=‐a&search_string=3.5.3.1) are manganese‐containing isoforms, which appear to show differential distribution, where the ARG1 isoform predominates in the liver and erythrocytes, while ARG2 is associated more with the kidney.

Information on members of this family may be found in the http://www.guidetopharmacology.org/GRAC/FamilyDisplayForward?familyId=250.

### Comments

http://www.guidetopharmacology.org/GRAC/LigandDisplayForward?ligandId=5227, an intermediate in NOS metabolism of L‐arginine acts as a weak inhibitor and may function as a physiological regulator of arginase activity. Although isoform‐selective inhibitors of arginase are not available, examples of inhibitors selective for arginase compared to NOS are http://www.guidetopharmacology.org/GRAC/LigandDisplayForward?ligandId=5091 [http://www.ncbi.nlm.nih.gov/pubmed/10637120?dopt=AbstractPlus], http://www.guidetopharmacology.org/GRAC/LigandDisplayForward?ligandId=5264 [http://www.ncbi.nlm.nih.gov/pubmed/11478904?dopt=AbstractPlus, http://www.ncbi.nlm.nih.gov/pubmed/11258879?dopt=AbstractPlus] and http://www.guidetopharmacology.org/GRAC/LigandDisplayForward?ligandId=5107 [http://www.ncbi.nlm.nih.gov/pubmed/10454520?dopt=AbstractPlus, http://www.ncbi.nlm.nih.gov/pubmed/11478904?dopt=AbstractPlus].

## http://www.guidetopharmacology.org/GRAC/FamilyDisplayForward?familyId=251

**Table:** 

Nomenclature	http://www.guidetopharmacology.org/GRAC/ObjectDisplayForward?objectId=1246
Common abbreviation	AGAT
HGNC, UniProt	https://www.genenames.org/data/gene‐symbol‐report/#!/hgnc_id/HGNC:4175, http://www.uniprot.org/uniprot/P50440
EC number	http://www.genome.jp/dbget‐bin/www_bget?ec:2.1.4.1

## http://www.guidetopharmacology.org/GRAC/FamilyDisplayForward?familyId=252

### Overview

Dimethylarginine dimethylaminohydrolases (DDAH, http://www.genome.jp/kegg‐bin/search_brite?option=‐a&search_string=3.5.3.18) are cytoplasmic enzymes which hydrolyse http://www.guidetopharmacology.org/GRAC/LigandDisplayForward?ligandId=5229 to form http://www.guidetopharmacology.org/GRAC/LigandDisplayForward?ligandId=5177 and http://www.guidetopharmacology.org/GRAC/LigandDisplayForward?ligandId=722.

**Table:** 

Nomenclature	http://www.guidetopharmacology.org/GRAC/ObjectDisplayForward?objectId=1247	http://www.guidetopharmacology.org/GRAC/ObjectDisplayForward?objectId=1248
Common abbreviation	DDAH1	DDAH2
HGNC, UniProt	https://www.genenames.org/data/gene‐symbol‐report/#!/hgnc_id/HGNC:2715, http://www.uniprot.org/uniprot/O94760	https://www.genenames.org/data/gene‐symbol‐report/#!/hgnc_id/HGNC:2716, http://www.uniprot.org/uniprot/O95865
EC number	http://www.genome.jp/dbget‐bin/www_bget?ec:3.5.3.18	http://www.genome.jp/dbget‐bin/www_bget?ec:3.5.3.18
Cofactors	http://www.guidetopharmacology.org/GRAC/LigandDisplayForward?ligandId=566	–
Inhibitors	http://www.guidetopharmacology.org/GRAC/LigandDisplayForward?ligandId=8830 (p*K*_i_ 5.7) [http://www.ncbi.nlm.nih.gov/pubmed/19013076?dopt=AbstractPlus]	–

## http://www.guidetopharmacology.org/GRAC/FamilyDisplayForward?familyId=253

### Overview

Nitric oxide synthases (NOS, http://www.genome.jp/kegg‐bin/search_brite?option=‐a&search_string=1.14.13.39) are a family of oxidoreductases that synthesize nitric oxide (NO.) via the NADPH and oxygen‐dependent consumption of http://www.guidetopharmacology.org/GRAC/LigandDisplayForward?ligandId=721 with the resultant by‐product, http://www.guidetopharmacology.org/GRAC/LigandDisplayForward?ligandId=722. There are 3 NOS isoforms and they are related by their capacity to produce NO, highly conserved organization of functional domains and significant homology at the amino acid level. NOS isoforms are functionally distinguished by the cell type where they are expressed, intracellular targeting and transcriptional and post‐translation mechanisms regulating enzyme activity. The nomenclature suggested by **NC‐IUPHAR** of NOS I, II and III [http://www.ncbi.nlm.nih.gov/pubmed/9228663?dopt=AbstractPlus] has not gained wide acceptance, and the 3 isoforms are more commonly referred to as neuronal NOS (nNOS), inducible NOS (iNOS) and endothelial NOS (eNOS) which reflect the location of expression (nNOS and eNOS) and inducible expression (iNOS). All are dimeric enzymes that shuttle electrons from NADPH, which binds to a C‐terminal reductase domain, through the flavins FAD and FMN to the oxygenase domain of the other monomer to enable the BH4‐dependent reduction of heme bound oxygen for insertion into the substrate, L‐arginine. Electron flow from reductase to oxygenase domain is controlled by calmodulin binding to canonical calmodulin binding motif located between these domains. eNOS and nNOS isoforms are activated at concentrations of calcium greater than 100 nM, while iNOS shows higher affinity for Ca^2+^/http://www.guidetopharmacology.org/GRAC/LigandDisplayForward?ligandId=2351 (https://www.genenames.org/data/gene‐symbol‐report/#!/hgnc_id/HGNC:1442, http://www.uniprot.org/uniprot/P62158) with great avidity and is essentially calcium‐independent and constitutively active. Efficient stimulus‐dependent coupling of nNOS and eNOS is achieved *via* subcellular targeting through respective N‐terminal PDZ and fatty acid acylation domains whereas iNOS is largely cytosolic and function is independent of intracellular location. nNOS is primarily expressed in the brain and neuronal tissue, iNOS in immune cells such as macrophages and eNOS in the endothelial layer of the vasculature although exceptions in other cells have been documented. http://www.guidetopharmacology.org/GRAC/LigandDisplayForward?ligandId=5213 and related modified arginine analogues are inhibitors of all three isoforms, with IC_50_ values in the micromolar range.

**Table:** 

Nomenclature	http://www.guidetopharmacology.org/GRAC/ObjectDisplayForward?objectId=1249	http://www.guidetopharmacology.org/GRAC/ObjectDisplayForward?objectId=1250	http://www.guidetopharmacology.org/GRAC/ObjectDisplayForward?objectId=1251
Common abbreviation	eNOS	iNOS	nNOS
HGNC, UniProt	https://www.genenames.org/data/gene‐symbol‐report/#!/hgnc_id/HGNC:7876, http://www.uniprot.org/uniprot/P29474	https://www.genenames.org/data/gene‐symbol‐report/#!/hgnc_id/HGNC:7873, http://www.uniprot.org/uniprot/P35228	https://www.genenames.org/data/gene‐symbol‐report/#!/hgnc_id/HGNC:7872, http://www.uniprot.org/uniprot/P29475
EC number	http://www.genome.jp/dbget‐bin/www_bget?ec:1.14.13.39	http://www.genome.jp/dbget‐bin/www_bget?ec:1.14.13.39	http://www.genome.jp/dbget‐bin/www_bget?ec:1.14.13.39
Endogenous Substrate	http://www.guidetopharmacology.org/GRAC/LigandDisplayForward?ligandId=721	http://www.guidetopharmacology.org/GRAC/LigandDisplayForward?ligandId=721	http://www.guidetopharmacology.org/GRAC/LigandDisplayForward?ligandId=721
Products	http://www.guidetopharmacology.org/GRAC/LigandDisplayForward?ligandId=2509, http://www.guidetopharmacology.org/GRAC/LigandDisplayForward?ligandId=722	http://www.guidetopharmacology.org/GRAC/LigandDisplayForward?ligandId=2509, http://www.guidetopharmacology.org/GRAC/LigandDisplayForward?ligandId=722	http://www.guidetopharmacology.org/GRAC/LigandDisplayForward?ligandId=722, http://www.guidetopharmacology.org/GRAC/LigandDisplayForward?ligandId=2509
Cofactors	oxygen, BH4, http://www.guidetopharmacology.org/GRAC/LigandDisplayForward?ligandId=566, http://www.guidetopharmacology.org/GRAC/LigandDisplayForward?ligandId=5185, http://www.guidetopharmacology.org/GRAC/LigandDisplayForward?ligandId=3041, http://www.guidetopharmacology.org/GRAC/LigandDisplayForward?ligandId=4349, http://www.guidetopharmacology.org/GRAC/LigandDisplayForward?ligandId=5184	http://www.guidetopharmacology.org/GRAC/LigandDisplayForward?ligandId=4349, http://www.guidetopharmacology.org/GRAC/LigandDisplayForward?ligandId=5185, http://www.guidetopharmacology.org/GRAC/LigandDisplayForward?ligandId=5184, oxygen, http://www.guidetopharmacology.org/GRAC/LigandDisplayForward?ligandId=3041, http://www.guidetopharmacology.org/GRAC/LigandDisplayForward?ligandId=566, BH4	http://www.guidetopharmacology.org/GRAC/LigandDisplayForward?ligandId=5184, http://www.guidetopharmacology.org/GRAC/LigandDisplayForward?ligandId=4349, oxygen, BH4, http://www.guidetopharmacology.org/GRAC/LigandDisplayForward?ligandId=5185, http://www.guidetopharmacology.org/GRAC/LigandDisplayForward?ligandId=3041, http://www.guidetopharmacology.org/GRAC/LigandDisplayForward?ligandId=566
Selective inhibitors	–	http://www.guidetopharmacology.org/GRAC/LigandDisplayForward?ligandId=5102 (pIC_50_ 8.2) [http://www.ncbi.nlm.nih.gov/pubmed/9030556?dopt=AbstractPlus], http://www.guidetopharmacology.org/GRAC/LigandDisplayForward?ligandId=5111 (pIC_50_ 7.4) [http://www.ncbi.nlm.nih.gov/pubmed/8937711?dopt=AbstractPlus], http://www.guidetopharmacology.org/GRAC/LigandDisplayForward?ligandId=5246 (pIC_50_ 7.3) [http://www.ncbi.nlm.nih.gov/pubmed/7523409?dopt=AbstractPlus], http://www.guidetopharmacology.org/GRAC/LigandDisplayForward?ligandId=5231 (pIC_50_ 5.5) [http://www.ncbi.nlm.nih.gov/pubmed/7525961?dopt=AbstractPlus], http://www.guidetopharmacology.org/GRAC/LigandDisplayForward?ligandId=5135 [http://www.ncbi.nlm.nih.gov/pubmed/1378415?dopt=AbstractPlus]	http://www.guidetopharmacology.org/GRAC/LigandDisplayForward?ligandId=5113 (pIC_50_ 6.1–6.5) [http://www.ncbi.nlm.nih.gov/pubmed/7544863?dopt=AbstractPlus], http://www.guidetopharmacology.org/GRAC/LigandDisplayForward?ligandId=5127 (pIC_50_ 5.3) [http://www.ncbi.nlm.nih.gov/pubmed/7693279?dopt=AbstractPlus]

### Comments

The reductase domain of NOS catalyses the reduction of cytochrome c and other redox‐active dyes [http://www.ncbi.nlm.nih.gov/pubmed/9433128?dopt=AbstractPlus]. NADPH:O_2_ oxidoreductase catalyses the formation of superoxide anion/http://www.guidetopharmacology.org/GRAC/LigandDisplayForward?ligandId=2448 in the absence of http://www.guidetopharmacology.org/GRAC/LigandDisplayForward?ligandId=721 and http://www.guidetopharmacology.org/GRAC/LigandDisplayForward?ligandId=5276.

### Further reading on Nitric oxide synthases

Garcia‐Ortiz A and Serrador JM (2018) Nitric Oxide Signaling in T Cell‐Mediated Immunity *Trends Mol Med* **24**: 412–427 https://www.ncbi.nlm.nih.gov/pubmed/29519621

Lundberg JO *et al*. (2015) Strategies to increase nitric oxide signalling in cardiovascular disease. *Nat Rev Drug Discov* **14**: 623–41 https://www.ncbi.nlm.nih.gov/pubmed/26265312?dopt=AbstractPlus

Oliveira‐Paula GH *et al*. (2016) Endothelial nitric oxide synthase: From biochemistry and gene structure to clinical implications of NOS3 polymorphisms. *Gene* **575**: 584–99 https://www.ncbi.nlm.nih.gov/pubmed/26428312?dopt=AbstractPlus

Stuehr DJ and Haque MM (2019) Nitric oxide synthase enzymology in the 20 years after the Nobel Prize. *BrJPharmacol* **176**: 177–188 https://www.ncbi.nlm.nih.gov/pubmed/30402946

Wallace JL (2019) Nitric oxide in the gastrointestinal tract: opportunities for drug development. *Br J Pharmacol* **176**: 147–154 https://www.ncbi.nlm.nih.gov/pubmed/30357812

### Further reading on L‐Arginine turnover

Lai L *et al*. (2016) Modulating DDAH/NOS Pathway to Discover Vasoprotective Insulin Sensitizers. *J Diabetes Res* **2016**: 1982096 https://www.ncbi.nlm.nih.gov/pubmed/26770984?dopt=AbstractPlus

Moncada S *et al*. (1997) International Union of Pharmacology Nomenclature in Nitric Oxide Re‐search. *Pharmacol. Rev*. **49**: 137–42 https://www.ncbi.nlm.nih.gov/pubmed/9228663?dopt=AbstractPlus

Pekarova M *et al*. (2015) The crucial role of l‐arginine in macrophage activation: What you need to know about it. *Life Sci*. **137**: 44–8 https://www.ncbi.nlm.nih.gov/pubmed/26188591?dopt=AbstractPlus

Pudlo M *et al*. (2017) Arginase Inhibitors: A Rational Approach Over One Century. *Med Res Rev* **37**: 475–513 https://www.ncbi.nlm.nih.gov/pubmed/27862081?dopt=AbstractPlus

Sudar‐Milovanovic E *et al*. (2016) Benefits of L‐Arginine on Cardiovascular System. *Mini Rev Med Chem* **16**: 94–103 https://www.ncbi.nlm.nih.gov/pubmed/26471966?dopt=AbstractPlus

## http://www.guidetopharmacology.org/GRAC/FamilyDisplayForward?familyId=842

### Overview

Carbonic anhydrases facilitate the interconversion of water and carbon dioxide with bicarbonate ions and protons (EC 4.2.1.1), with over a dozen gene products identified in man. The enzymes function in acid‐base balance and the movement of carbon dioxide and water. They are targetted for therapeutic gain by particular antiglaucoma agents and diuretics.

**Table:** 

Nomenclature	http://www.guidetopharmacology.org/GRAC/ObjectDisplayForward?objectId=2597	http://www.guidetopharmacology.org/GRAC/ObjectDisplayForward?objectId=2749	http://www.guidetopharmacology.org/GRAC/ObjectDisplayForward?objectId=2747	http://www.guidetopharmacology.org/GRAC/ObjectDisplayForward?objectId=2748	http://www.guidetopharmacology.org/GRAC/ObjectDisplayForward?objectId=2598
Common abbreviation	CA I	CA VII	CA XII	CA XIII	CA XIV
HGNC, UniProt	https://www.genenames.org/data/gene‐symbol‐report/#!/hgnc_id/HGNC:1368, http://www.uniprot.org/uniprot/P00915	https://www.genenames.org/data/gene‐symbol‐report/#!/hgnc_id/HGNC:1381, http://www.uniprot.org/uniprot/P43166	https://www.genenames.org/data/gene‐symbol‐report/#!/hgnc_id/HGNC:1371, http://www.uniprot.org/uniprot/O43570	https://www.genenames.org/data/gene‐symbol‐report/#!/hgnc_id/HGNC:14914, http://www.uniprot.org/uniprot/Q8N1Q1	https://www.genenames.org/data/gene‐symbol‐report/#!/hgnc_id/HGNC:1372, http://www.uniprot.org/uniprot/Q9ULX7
EC number	http://www.genome.jp/dbget‐bin/www_bget?ec:4.2.1.1	http://www.genome.jp/dbget‐bin/www_bget?ec:4.2.1.1	http://www.genome.jp/dbget‐bin/www_bget?ec:4.2.1.1	http://www.genome.jp/dbget‐bin/www_bget?ec:4.2.1.1	http://www.genome.jp/dbget‐bin/www_bget?ec:4.2.1.1
Inhibitors	http://www.guidetopharmacology.org/GRAC/LigandDisplayForward?ligandId=7147 (p*K*_i_ 6.5)	http://www.guidetopharmacology.org/GRAC/LigandDisplayForward?ligandId=6828 (p*K*_i_ 8.7) [http://www.ncbi.nlm.nih.gov/pubmed/23965175?dopt=AbstractPlus], http://www.guidetopharmacology.org/GRAC/LigandDisplayForward?ligandId=6792 (p*K*_i_ 8.6) [http://www.ncbi.nlm.nih.gov/pubmed/20605094?dopt=AbstractPlus], http://www.guidetopharmacology.org/GRAC/LigandDisplayForward?ligandId=6797 (p*K*_i_ 8.6) [http://www.ncbi.nlm.nih.gov/pubmed/23965175?dopt=AbstractPlus], http://www.guidetopharmacology.org/GRAC/LigandDisplayForward?ligandId=7147 (p*K*_i_ 8.6) [http://www.ncbi.nlm.nih.gov/pubmed/19119014?dopt=AbstractPlus]	http://www.guidetopharmacology.org/GRAC/LigandDisplayForward?ligandId=10149 (p*K*_i_ 8.4) [http://www.ncbi.nlm.nih.gov/pubmed/26233435?dopt=AbstractPlus]	–	–

### Further reading on Carbonic anhydrases

Imtaiyaz Hassan M, Shajee B, Waheed A, Ahmad F and Sly WS. (2013) Structure, function and applications of carbonic anhydrase isozymes. *Bioorg Med Chem* **21**: 1570–70 https://www.ncbi.nlm.nih.gov/pubmed/22607884

Supuran CT (2017) Advances in structure‐based drug discovery of carbonic anhydrase inhibitors. *Expert Opin Drug Discov* **12**: 61–88 https://www.ncbi.nlm.nih.gov/pubmed/27783541

Supuran CT (2018) Carbonic anhydrase activators. *Future Med Chem* **10**: 561–573 https://www.ncbi.nlm.nih.gov/pubmed/29478330

## http://www.guidetopharmacology.org/GRAC/FamilyDisplayForward?familyId=240

## http://www.guidetopharmacology.org/GRAC/FamilyDisplayForward?familyId=255

### Overview

The carboxylases allow the production of new carbon‐carbon bonds by introducing HCO_3_^‐^ or CO_2_ into target molecules. Two groups of carboxylase activities, some of which are bidirectional, can be defined on the basis of the cofactor requirement, making use of http://www.guidetopharmacology.org/GRAC/LigandDisplayForward?ligandId=4787 (EC 6.4.1.‐) or http://www.guidetopharmacology.org/GRAC/LigandDisplayForward?ligandId=5286 (EC 4.1.1.‐).

**Table:** 

Nomenclature	http://www.guidetopharmacology.org/GRAC/ObjectDisplayForward?objectId=1262	http://www.guidetopharmacology.org/GRAC/ObjectDisplayForward?objectId=1263	http://www.guidetopharmacology.org/GRAC/ObjectDisplayForward?objectId=1264	http://www.guidetopharmacology.org/GRAC/ObjectDisplayForward?objectId=1265	http://www.guidetopharmacology.org/GRAC/ObjectDisplayForward?objectId=1268
Common abbreviation	PC	ACC1	ACC2	PCCA,PCCB	GGCX
HGNC, UniProt	https://www.genenames.org/data/gene‐symbol‐report/#!/hgnc_id/HGNC:8636, http://www.uniprot.org/uniprot/P11498	https://www.genenames.org/data/gene‐symbol‐report/#!/hgnc_id/HGNC:84, http://www.uniprot.org/uniprot/Q13085	https://www.genenames.org/data/gene‐symbol‐report/#!/hgnc_id/HGNC:85, http://www.uniprot.org/uniprot/O00763	–	https://www.genenames.org/data/gene‐symbol‐report/#!/hgnc_id/HGNC:4247, http://www.uniprot.org/uniprot/P38435
Subunits	–	–	–	http://www.guidetopharmacology.org/GRAC/ObjectDisplayForward?objectId=1267, http://www.guidetopharmacology.org/GRAC/ObjectDisplayForward?objectId=1266	–
EC number	http://www.genome.jp/dbget‐bin/www_bget?ec:6.4.1.1	http://www.genome.jp/dbget‐bin/www_bget?ec:6.4.1.2	http://www.genome.jp/dbget‐bin/www_bget?ec:6.4.1.2	http://www.genome.jp/dbget‐bin/www_bget?ec:6.4.1.3	http://www.genome.jp/dbget‐bin/www_bget?ec:4.1.1.90
Endogenous substrates	http://www.guidetopharmacology.org/GRAC/LigandDisplayForward?ligandId=1713, http://www.guidetopharmacology.org/GRAC/LigandDisplayForward?ligandId=4809	http://www.guidetopharmacology.org/GRAC/LigandDisplayForward?ligandId=1713, http://www.guidetopharmacology.org/GRAC/LigandDisplayForward?ligandId=3038	http://www.guidetopharmacology.org/GRAC/LigandDisplayForward?ligandId=3038, http://www.guidetopharmacology.org/GRAC/LigandDisplayForward?ligandId=1713	http://www.guidetopharmacology.org/GRAC/LigandDisplayForward?ligandId=5248, http://www.guidetopharmacology.org/GRAC/LigandDisplayForward?ligandId=1713	glutamyl peptides
Products	P_i_, http://www.guidetopharmacology.org/GRAC/LigandDisplayForward?ligandId=1712, http://www.guidetopharmacology.org/GRAC/LigandDisplayForward?ligandId=5236	P_i_, http://www.guidetopharmacology.org/GRAC/LigandDisplayForward?ligandId=1712, http://www.guidetopharmacology.org/GRAC/LigandDisplayForward?ligandId=5219	P_i_, http://www.guidetopharmacology.org/GRAC/LigandDisplayForward?ligandId=1712, http://www.guidetopharmacology.org/GRAC/LigandDisplayForward?ligandId=5219	http://www.guidetopharmacology.org/GRAC/LigandDisplayForward?ligandId=1712, http://www.guidetopharmacology.org/GRAC/LigandDisplayForward?ligandId=5223, P_i_	carboxyglutamyl peptides
Cofactors	http://www.guidetopharmacology.org/GRAC/LigandDisplayForward?ligandId=4787	http://www.guidetopharmacology.org/GRAC/LigandDisplayForward?ligandId=4787	http://www.guidetopharmacology.org/GRAC/LigandDisplayForward?ligandId=4787	http://www.guidetopharmacology.org/GRAC/LigandDisplayForward?ligandId=4787	http://www.guidetopharmacology.org/GRAC/LigandDisplayForward?ligandId=5286, http://www.guidetopharmacology.org/GRAC/LigandDisplayForward?ligandId=3041
Inhibitors	–	–	–	–	http://www.guidetopharmacology.org/GRAC/LigandDisplayForward?ligandId=6960
Selective inhibitors	–	http://www.guidetopharmacology.org/GRAC/LigandDisplayForward?ligandId=8884 (pIC_50_ 8) [http://www.ncbi.nlm.nih.gov/pubmed/23981033?dopt=AbstractPlus], http://www.guidetopharmacology.org/GRAC/LigandDisplayForward?ligandId=5280 (pIC_50_ 4.9) [http://www.ncbi.nlm.nih.gov/pubmed/14612531?dopt=AbstractPlus]	http://www.guidetopharmacology.org/GRAC/LigandDisplayForward?ligandId=8884 (pIC_50_ 8.4) [http://www.ncbi.nlm.nih.gov/pubmed/23981033?dopt=AbstractPlus], http://www.guidetopharmacology.org/GRAC/LigandDisplayForward?ligandId=5280 (pIC_50_ 4.9) [http://www.ncbi.nlm.nih.gov/pubmed/14612531?dopt=AbstractPlus]	–	–
Comments	–	Citrate and other dicarboxylic acids are allosteric activators of acetyl‐CoA carboxylase.	Citrate and other dicarboxylic acids are allosteric activators of acetyl‐CoA carboxylase.	Propionyl‐CoA carboxylase is able to function in both forward and reverse activity modes, as a ligase (carboxylase) or lyase (decarboxylase), respectively.	Loss‐of‐function mutations in γ‐glutamyl carboxylase are associated with http://omim.org/entry/277450.

### Comments

Dicarboxylic acids including http://www.guidetopharmacology.org/GRAC/LigandDisplayForward?ligandId=2478 are able to activate ACC1/ACC2 activity allosterically. PCC is able to function in forward and reverse modes as a ligase (carboxylase) or lyase (decarboxylase) activity, respectively. Loss‐of‐function mutations in GGCX are associated with clotting disorders.

## http://www.guidetopharmacology.org/GRAC/FamilyDisplayForward?familyId=256

### Overview

The decarboxylases generate CO_2_ and the indicated products from acidic substrates, requiring http://www.guidetopharmacology.org/GRAC/LigandDisplayForward?ligandId=5249 or http://www.guidetopharmacology.org/GRAC/LigandDisplayForward?ligandId=4809 as a co‐factor.

**Table:** 

Nomenclature	http://www.guidetopharmacology.org/GRAC/ObjectDisplayForward?objectId=1272	http://www.guidetopharmacology.org/GRAC/ObjectDisplayForward?objectId=1273	http://www.guidetopharmacology.org/GRAC/ObjectDisplayForward?objectId=1274
Common abbreviation	GAD1	GAD2	HDC
HGNC, UniProt	https://www.genenames.org/data/gene‐symbol‐report/#!/hgnc_id/HGNC:4092, http://www.uniprot.org/uniprot/Q99259	https://www.genenames.org/data/gene‐symbol‐report/#!/hgnc_id/HGNC:4093, http://www.uniprot.org/uniprot/Q05329	https://www.genenames.org/data/gene‐symbol‐report/#!/hgnc_id/HGNC:4855, http://www.uniprot.org/uniprot/P19113
EC number	http://www.genome.jp/dbget‐bin/www_bget?ec:4.1.1.15: http://www.guidetopharmacology.org/GRAC/LigandDisplayForward?ligandId=1369 + H^+^ ‐> http://www.guidetopharmacology.org/GRAC/LigandDisplayForward?ligandId=1067 + CO_2_	http://www.genome.jp/dbget‐bin/www_bget?ec:4.1.1.15: http://www.guidetopharmacology.org/GRAC/LigandDisplayForward?ligandId=1369 + H^+^ ‐> http://www.guidetopharmacology.org/GRAC/LigandDisplayForward?ligandId=1067 + CO_2_	http://www.genome.jp/dbget‐bin/www_bget?ec:4.1.1.22
Endogenous substrates	http://www.guidetopharmacology.org/GRAC/LigandDisplayForward?ligandId=1369, http://www.guidetopharmacology.org/GRAC/LigandDisplayForward?ligandId=3309	http://www.guidetopharmacology.org/GRAC/LigandDisplayForward?ligandId=1369, http://www.guidetopharmacology.org/GRAC/LigandDisplayForward?ligandId=3309	http://www.guidetopharmacology.org/GRAC/LigandDisplayForward?ligandId=3310
Products	http://www.guidetopharmacology.org/GRAC/LigandDisplayForward?ligandId=1067	http://www.guidetopharmacology.org/GRAC/LigandDisplayForward?ligandId=1067	http://www.guidetopharmacology.org/GRAC/LigandDisplayForward?ligandId=1204
Cofactors	http://www.guidetopharmacology.org/GRAC/LigandDisplayForward?ligandId=5249	http://www.guidetopharmacology.org/GRAC/LigandDisplayForward?ligandId=5249	http://www.guidetopharmacology.org/GRAC/LigandDisplayForward?ligandId=5249
Selective inhibitors	http://www.guidetopharmacology.org/GRAC/LigandDisplayForward?ligandId=5267	http://www.guidetopharmacology.org/GRAC/LigandDisplayForward?ligandId=5267	http://www.guidetopharmacology.org/GRAC/LigandDisplayForward?ligandId=5134, http://www.guidetopharmacology.org/GRAC/LigandDisplayForward?ligandId=5189 [http://www.ncbi.nlm.nih.gov/pubmed/7452304?dopt=AbstractPlus]
Comments	http://www.guidetopharmacology.org/GRAC/LigandDisplayForward?ligandId=3309 is a less rapidly metabolised substrate of mouse brain glutamic acid decarboxylase generating β‐alanine [http://www.ncbi.nlm.nih.gov/pubmed/4700449?dopt=AbstractPlus]. Autoantibodies against GAD1 and GAD2 are elevated in type 1 diabetes mellitus and neurological disorders (see Further reading).		–

**Table:** 

Nomenclature	http://www.guidetopharmacology.org/GRAC/ObjectDisplayForward?objectId=1270	http://www.guidetopharmacology.org/GRAC/ObjectDisplayForward?objectId=1271	http://www.guidetopharmacology.org/GRAC/ObjectDisplayForward?objectId=1275	http://www.guidetopharmacology.org/GRAC/ObjectDisplayForward?objectId=1276	http://www.guidetopharmacology.org/GRAC/ObjectDisplayForward?objectId=1277	http://www.guidetopharmacology.org/GRAC/ObjectDisplayForward?objectId=1269
Common abbreviation	ADC	AADC	MLYCD	ODC	PSDC	SAMDC
HGNC, UniProt	https://www.genenames.org/data/gene‐symbol‐report/#!/hgnc_id/HGNC:29957, http://www.uniprot.org/uniprot/Q96A70	https://www.genenames.org/data/gene‐symbol‐report/#!/hgnc_id/HGNC:2719, http://www.uniprot.org/uniprot/P20711	https://www.genenames.org/data/gene‐symbol‐report/#!/hgnc_id/HGNC:7150, http://www.uniprot.org/uniprot/O95822	https://www.genenames.org/data/gene‐symbol‐report/#!/hgnc_id/HGNC:8109, http://www.uniprot.org/uniprot/P11926	https://www.genenames.org/data/gene‐symbol‐report/#!/hgnc_id/HGNC:8999, http://www.uniprot.org/uniprot/Q9UG56	https://www.genenames.org/data/gene‐symbol‐report/#!/hgnc_id/HGNC:457, http://www.uniprot.org/uniprot/P17707
EC number	http://www.genome.jp/dbget‐bin/www_bget?ec:4.1.1.19	http://www.genome.jp/dbget‐bin/www_bget?ec:4.1.1.28: http://www.guidetopharmacology.org/GRAC/LigandDisplayForward?ligandId=3639 ‐> http://www.guidetopharmacology.org/GRAC/LigandDisplayForward?ligandId=940 + CO_2_ http://www.guidetopharmacology.org/GRAC/LigandDisplayForward?ligandId=4671 ‐> http://www.guidetopharmacology.org/GRAC/LigandDisplayForward?ligandId=5 + CO_2_ This enzyme also catalyses the following reaction:: http://www.guidetopharmacology.org/GRAC/LigandDisplayForward?ligandId=717 ‐> http://www.guidetopharmacology.org/GRAC/LigandDisplayForward?ligandId=125 + CO_2_	http://www.genome.jp/dbget‐bin/www_bget?ec:4.1.1.9	http://www.genome.jp/dbget‐bin/www_bget?ec:4.1.1.17	http://www.genome.jp/dbget‐bin/www_bget?ec:4.1.1.65	http://www.genome.jp/dbget‐bin/www_bget?ec:4.1.1.50
Endogenous substrates	http://www.guidetopharmacology.org/GRAC/LigandDisplayForward?ligandId=721	http://www.guidetopharmacology.org/GRAC/LigandDisplayForward?ligandId=3639, http://www.guidetopharmacology.org/GRAC/LigandDisplayForward?ligandId=4671, http://www.guidetopharmacology.org/GRAC/LigandDisplayForward?ligandId=717	http://www.guidetopharmacology.org/GRAC/LigandDisplayForward?ligandId=5219	http://www.guidetopharmacology.org/GRAC/LigandDisplayForward?ligandId=725	http://www.guidetopharmacology.org/GRAC/LigandDisplayForward?ligandId=3638	http://www.guidetopharmacology.org/GRAC/LigandDisplayForward?ligandId=4786
Products	http://www.guidetopharmacology.org/GRAC/LigandDisplayForward?ligandId=4127 [http://www.ncbi.nlm.nih.gov/pubmed/14738999?dopt=AbstractPlus]	http://www.guidetopharmacology.org/GRAC/LigandDisplayForward?ligandId=5, http://www.guidetopharmacology.org/GRAC/LigandDisplayForward?ligandId=940	http://www.guidetopharmacology.org/GRAC/LigandDisplayForward?ligandId=3038	http://www.guidetopharmacology.org/GRAC/LigandDisplayForward?ligandId=2388	http://www.guidetopharmacology.org/GRAC/LigandDisplayForward?ligandId=2796	http://www.guidetopharmacology.org/GRAC/LigandDisplayForward?ligandId=5121
Cofactors	http://www.guidetopharmacology.org/GRAC/LigandDisplayForward?ligandId=5249	http://www.guidetopharmacology.org/GRAC/LigandDisplayForward?ligandId=5249	http://www.guidetopharmacology.org/GRAC/LigandDisplayForward?ligandId=5249	http://www.guidetopharmacology.org/GRAC/LigandDisplayForward?ligandId=5249	http://www.guidetopharmacology.org/GRAC/LigandDisplayForward?ligandId=4809	http://www.guidetopharmacology.org/GRAC/LigandDisplayForward?ligandId=4809
Selective inhibitors	–	http://www.guidetopharmacology.org/GRAC/LigandDisplayForward?ligandId=5116, http://www.guidetopharmacology.org/GRAC/LigandDisplayForward?ligandId=5217, http://www.guidetopharmacology.org/GRAC/LigandDisplayForward?ligandId=5150 [http://www.ncbi.nlm.nih.gov/pubmed/22384042?dopt=AbstractPlus], http://www.guidetopharmacology.org/GRAC/LigandDisplayForward?ligandId=5159	–	http://www.guidetopharmacology.org/GRAC/LigandDisplayForward?ligandId=5139 (pIC_50_ 7.5) [http://www.ncbi.nlm.nih.gov/pubmed/1573631?dopt=AbstractPlus], http://www.guidetopharmacology.org/GRAC/LigandDisplayForward?ligandId=5176 (p*K*_d_ 4.9) [http://www.ncbi.nlm.nih.gov/pubmed/12859253?dopt=AbstractPlus]	–	http://www.guidetopharmacology.org/GRAC/LigandDisplayForward?ligandId=5268 (pIC_50_ 8) [http://www.ncbi.nlm.nih.gov/pubmed/8340919?dopt=AbstractPlus]
Comments	The presence of a functional ADC activity in human tissues has been questioned [http://www.ncbi.nlm.nih.gov/pubmed/14763899?dopt=AbstractPlus].	AADC is a homodimer.	Inhibited by AMP‐activated protein kinase‐evoked phosphorylation [http://www.ncbi.nlm.nih.gov/pubmed/10854420?dopt=AbstractPlus]	The activity of ODC is regulated by the presence of an antizyme (http://www.ensembl.org/Homo_sapiens/Gene/Summary?g=ENSG00000104904;r=19:2269520‐2273487;t=ENST00000322297) and an ODC antizyme inhibitor (http://www.ensembl.org/Homo_sapiens/Gene/Summary?g=ENSG00000155096;r=8:103838585‐103906092).	S‐allylglycine is also an inhibitor of SAMDC [http://www.ncbi.nlm.nih.gov/pubmed/438812?dopt=AbstractPlus].	http://www.guidetopharmacology.org/GRAC/LigandDisplayForward?ligandId=5267 is also an inhibitor of SAMDC [http://www.ncbi.nlm.nih.gov/pubmed/438812?dopt=AbstractPlus].

### Further reading on Carboxylases and decarboxylases

Bale S *et al*. (2010) Structural biology of S‐adenosylmethionine decarboxylase. *Amino Acids* **38**: 451–60 [https://www.ncbi.nlm.nih.gov/pubmed/19997761?dopt=AbstractPlus

Di Bartolomeo F *et al*. (2017) Cell biology, physiology and enzymology of phosphatidylserine decarboxylase. *Biochim Biophys Acta Mol Cell Biol Lipids* **1862**: 25–38 https://www.ncbi.nlm.nih.gov/pubmed/27650064

Jitrapakdee S *et al*. (2008) Structure, mechanism and regulation of pyruvate carboxylase. *Biochem. J*. **413**: 369–87 https://www.ncbi.nlm.nih.gov/pubmed/18613815?dopt=AbstractPlus

Lietzan AD *et al*. (2014) Functionally diverse biotin‐dependent enzymes with oxaloacetate decarboxylase activity. *Arch. Biochem. Biophys*. **544**: 75–86 https://www.ncbi.nlm.nih.gov/pubmed/24184447?dopt=AbstractPlus

Sanchez‐Jimenez F *et al*. (2016) Structural and functional analogies and differences between histi‐dine decarboxylase and aromatic l‐amino acid decarboxylase molecular networks: Biomedical implications *Pharmacol Res* **114**: 90–102 https://www.ncbi.nlm.nih.gov/pubmed/27769832

Salie MJ and Thelen JJ (2016) Regulation and structure of the heteromeric acetyl‐CoA carboxylase. *Biochim Biophys Acta* **1861**: 1207–1213 https://www.ncbi.nlm.nih.gov/pubmed/27091637

Tong L. (2013) Structure and function of biotin‐dependent carboxylases. *Cell. Mol. Life Sci*. **70**: 863–91 https://www.ncbi.nlm.nih.gov/pubmed/22869039?dopt=AbstractPlus

Vance JE *et al*. (2013) Formation and function of phosphatidylserine and phosphatidylethanolamine in mammalian cells. *Biochim. Biophys. Acta* **1831**: 543–54 https://www.ncbi.nlm.nih.gov/pubmed/22960354?dopt=AbstractPlus

## http://www.guidetopharmacology.org/GRAC/FamilyDisplayForward?familyId=766

### Overview

Catecholamines are defined by the presence of two adjacent hydroxyls on a benzene ring with a sidechain containing an amine. The predominant catacholamines in mammalian biology are the neurotransmitter/hormones http://www.guidetopharmacology.org/GRAC/LigandDisplayForward?ligandId=940, http://www.guidetopharmacology.org/GRAC/LigandDisplayForward?ligandId=505 (norepinephrine) and http://www.guidetopharmacology.org/GRAC/LigandDisplayForward?ligandId=479 (epinephrine). These hormone/transmitters are synthesized by sequential metabolism from http://www.guidetopharmacology.org/GRAC/LigandDisplayForward?ligandId=3313 via http://www.guidetopharmacology.org/GRAC/LigandDisplayForward?ligandId=4791. Hydroxylation of http://www.guidetopharmacology.org/GRAC/LigandDisplayForward?ligandId=4791 generates http://www.guidetopharmacology.org/GRAC/LigandDisplayForward?ligandId=3639, which is decarboxylated to form http://www.guidetopharmacology.org/GRAC/LigandDisplayForward?ligandId=940. Hydroxylation of the ethylamine sidechain generates http://www.guidetopharmacology.org/GRAC/LigandDisplayForward?ligandId=505 (norepinephrine), which can be methylated to form http://www.guidetopharmacology.org/GRAC/LigandDisplayForward?ligandId=479 (epinephrine). In particular neuronal and adrenal chromaffin cells, the catecholamines http://www.guidetopharmacology.org/GRAC/LigandDisplayForward?ligandId=940, http://www.guidetopharmacology.org/GRAC/LigandDisplayForward?ligandId=505 and http://www.guidetopharmacology.org/GRAC/LigandDisplayForward?ligandId=479 are accumulated into vesicles under the influence of the http://www.guidetopharmacology.org/GRAC/FamilyDisplayForward?familyId=193 (VMAT1/SLC18A1 and VMAT2/SLC18A2). After release into the synapse or the blood‐stream, catecholamines are accumulated through the action cell‐surface transporters, primarily the dopamine (http://www.guidetopharmacology.org/GRAC/FamilyDisplayForward?familyId=144#Monoamine_transporter_subfamily) and norepinephrine transporter (http://www.guidetopharmacology.org/GRAC/FamilyDisplayForward?familyId=144#Monoamine transporter subfamily). The primary routes of metabolism of these catecholamines are oxidation via monoamine oxidase activities of methylation via catechol O‐methyltransferase.

**Table:** 

Nomenclature	http://www.guidetopharmacology.org/GRAC/ObjectDisplayForward?objectId=1240	http://www.guidetopharmacology.org/GRAC/ObjectDisplayForward?objectId=2527	http://www.guidetopharmacology.org/GRAC/ObjectDisplayForward?objectId=1243	http://www.guidetopharmacology.org/GRAC/ObjectDisplayForward?objectId=2486
Common abbreviation	–	TAT	–	DBH
HGNC, UniProt	https://www.genenames.org/data/gene‐symbol‐report/#!/hgnc_id/HGNC:8582, http://www.uniprot.org/uniprot/P00439	https://www.genenames.org/data/gene‐symbol‐report/#!/hgnc_id/HGNC:11573, http://www.uniprot.org/uniprot/P17735	https://www.genenames.org/data/gene‐symbol‐report/#!/hgnc_id/HGNC:11782, http://www.uniprot.org/uniprot/P07101	https://www.genenames.org/data/gene‐symbol‐report/#!/hgnc_id/HGNC:2689, http://www.uniprot.org/uniprot/P09172
EC number	http://www.genome.jp/dbget‐bin/www_bget?ec:1.14.16.1: http://www.guidetopharmacology.org/GRAC/LigandDisplayForward?ligandId=3313 + O_2_ ‐> http://www.guidetopharmacology.org/GRAC/LigandDisplayForward?ligandId=4791	http://www.genome.jp/dbget‐bin/www_bget?ec:2.6.1.5: http://www.guidetopharmacology.org/GRAC/LigandDisplayForward?ligandId=4791 + http://www.guidetopharmacology.org/GRAC/LigandDisplayForward?ligandId=3636 ‐> http://www.guidetopharmacology.org/GRAC/LigandDisplayForward?ligandId=6629 + http://www.guidetopharmacology.org/GRAC/LigandDisplayForward?ligandId=1369	http://www.genome.jp/dbget‐bin/www_bget?ec:1.14.16.2: http://www.guidetopharmacology.org/GRAC/LigandDisplayForward?ligandId=4791 + O_2_ ‐> http://www.guidetopharmacology.org/GRAC/LigandDisplayForward?ligandId=3639	http://www.genome.jp/dbget‐bin/www_bget?ec:1.14.17.1: http://www.guidetopharmacology.org/GRAC/LigandDisplayForward?ligandId=940 + O_2_ = http://www.guidetopharmacology.org/GRAC/LigandDisplayForward?ligandId=505 + H_2_O
Endogenous substrates	http://www.guidetopharmacology.org/GRAC/LigandDisplayForward?ligandId=3313	–	http://www.guidetopharmacology.org/GRAC/LigandDisplayForward?ligandId=4791	–
Products	http://www.guidetopharmacology.org/GRAC/LigandDisplayForward?ligandId=4791	–	http://www.guidetopharmacology.org/GRAC/LigandDisplayForward?ligandId=3639	–
Cofactors	http://www.guidetopharmacology.org/GRAC/LigandDisplayForward?ligandId=5276	http://www.guidetopharmacology.org/GRAC/LigandDisplayForward?ligandId=5249	http://www.guidetopharmacology.org/GRAC/LigandDisplayForward?ligandId=5276, Fe^2+^	http://www.guidetopharmacology.org/GRAC/LigandDisplayForward?ligandId=4164, http://www.guidetopharmacology.org/GRAC/LigandDisplayForward?ligandId=4781
Endogenous activators	Protein kinase A‐mediated phosphorylation (Rat) [http://www.ncbi.nlm.nih.gov/pubmed/182695?dopt=AbstractPlus]	–	Protein kinase A‐mediated phosphorylation [http://www.ncbi.nlm.nih.gov/pubmed/33381?dopt=AbstractPlus]	–
Selective inhibitors	http://www.guidetopharmacology.org/GRAC/LigandDisplayForward?ligandId=5093 [http://www.ncbi.nlm.nih.gov/pubmed/944951?dopt=AbstractPlus] – Rat, http://www.guidetopharmacology.org/GRAC/LigandDisplayForward?ligandId=5240	–	http://www.guidetopharmacology.org/GRAC/LigandDisplayForward?ligandId=5095, http://www.guidetopharmacology.org/GRAC/LigandDisplayForward?ligandId=5114, http://www.guidetopharmacology.org/GRAC/LigandDisplayForward?ligandId=5117, http://www.guidetopharmacology.org/GRAC/LigandDisplayForward?ligandId=6956	http://www.guidetopharmacology.org/GRAC/LigandDisplayForward?ligandId=6630 (pIC_50_ 8) [http://www.ncbi.nlm.nih.gov/pubmed/9283721?dopt=AbstractPlus]
Comments	PAH is an iron bound homodimer or ‐tetramer from the same structural family as tyrosine 3‐monooxygenase and the tryptophan hydroxylases. Deficiency or loss‐of‐function of PAH is associated with http://omim.org/entry/612349	Tyrosine may also be metabolized in the liver by tyrosine transaminase to generate http://www.guidetopharmacology.org/GRAC/LigandDisplayForward?ligandId=6629, which can be further metabolized to homogentisic acid. TAT is a homodimer, where loss‐of‐function mutations are associated with http://omim.org/entry/276600.	TH is a homotetramer, which is inhibited by dopamine and other catecholamines in a physiological negative feedback pathway [http://www.ncbi.nlm.nih.gov/pubmed/21176768?dopt=AbstractPlus].	DBH is a homotetramer. A protein structurally‐related to DBH (http://www.genenames.org/data/hgnc_data.php?hgnc_id=21063, http://www.uniprot.org/uniprot/Q6UVY6) has been described and for which a function has yet to be identified [http://www.ncbi.nlm.nih.gov/pubmed/9751809?dopt=AbstractPlus].

**Table:** 

Nomenclature	http://www.guidetopharmacology.org/GRAC/ObjectDisplayForward?objectId=1271	http://www.guidetopharmacology.org/GRAC/ObjectDisplayForward?objectId=2496	http://www.guidetopharmacology.org/GRAC/ObjectDisplayForward?objectId=2472
Common abbreviation	AADC	PNMT	COMT
HGNC, UniProt	https://www.genenames.org/data/gene‐symbol‐report/#!/hgnc_id/HGNC:2719, http://www.uniprot.org/uniprot/P20711	https://www.genenames.org/data/gene‐symbol‐report/#!/hgnc_id/HGNC:9160, http://www.uniprot.org/uniprot/P11086	https://www.genenames.org/data/gene‐symbol‐report/#!/hgnc_id/HGNC:2228, http://www.uniprot.org/uniprot/P21964
EC number	http://www.genome.jp/dbget‐bin/www_bget?ec:4.1.1.28: http://www.guidetopharmacology.org/GRAC/LigandDisplayForward?ligandId=3639 ‐> http://www.guidetopharmacology.org/GRAC/LigandDisplayForward?ligandId=940 + CO_2_ http://www.guidetopharmacology.org/GRAC/LigandDisplayForward?ligandId=4671 ‐> http://www.guidetopharmacology.org/GRAC/LigandDisplayForward?ligandId=5 + CO_2_ This enzyme also catalyses the following reaction:: http://www.guidetopharmacology.org/GRAC/LigandDisplayForward?ligandId=717 ‐> http://www.guidetopharmacology.org/GRAC/LigandDisplayForward?ligandId=125 + CO_2_	http://www.genome.jp/dbget‐bin/www_bget?ec:2.1.1.28: http://www.guidetopharmacology.org/GRAC/LigandDisplayForward?ligandId=505 ‐> http://www.guidetopharmacology.org/GRAC/LigandDisplayForward?ligandId=479	http://www.genome.jp/dbget‐bin/www_bget?ec:2.1.1.6: S‐adenosyl‐L‐methionine + a catechol = S‐adenosyl‐L‐homocysteine + a guaiacol http://www.guidetopharmacology.org/GRAC/LigandDisplayForward?ligandId=505 ‐> http://www.guidetopharmacology.org/GRAC/LigandDisplayForward?ligandId=6643 http://www.guidetopharmacology.org/GRAC/LigandDisplayForward?ligandId=940 ‐> http://www.guidetopharmacology.org/GRAC/LigandDisplayForward?ligandId=6642 http://www.guidetopharmacology.org/GRAC/LigandDisplayForward?ligandId=6633 ‐> http://www.guidetopharmacology.org/GRAC/LigandDisplayForward?ligandId=6645 http://www.guidetopharmacology.org/GRAC/LigandDisplayForward?ligandId=479 ‐> http://www.guidetopharmacology.org/GRAC/LigandDisplayForward?ligandId=6644
Endogenous substrates	http://www.guidetopharmacology.org/GRAC/LigandDisplayForward?ligandId=3639, http://www.guidetopharmacology.org/GRAC/LigandDisplayForward?ligandId=4671, http://www.guidetopharmacology.org/GRAC/LigandDisplayForward?ligandId=717	–	http://www.guidetopharmacology.org/GRAC/LigandDisplayForward?ligandId=4786
Products	http://www.guidetopharmacology.org/GRAC/LigandDisplayForward?ligandId=5, http://www.guidetopharmacology.org/GRAC/LigandDisplayForward?ligandId=940	–	http://www.guidetopharmacology.org/GRAC/LigandDisplayForward?ligandId=6646 (soluble enzyme) (p*K*_i_ 9.6) [http://www.ncbi.nlm.nih.gov/pubmed/7703232?dopt=AbstractPlus], http://www.guidetopharmacology.org/GRAC/LigandDisplayForward?ligandId=6646 (membrane‐bound enzyme) (p*K*_i_ 9.5) [http://www.ncbi.nlm.nih.gov/pubmed/7703232?dopt=AbstractPlus], http://www.guidetopharmacology.org/GRAC/LigandDisplayForward?ligandId=6647 (soluble enzyme) (p*K*_i_ 9.5) [http://www.ncbi.nlm.nih.gov/pubmed/7703232?dopt=AbstractPlus], http://www.guidetopharmacology.org/GRAC/LigandDisplayForward?ligandId=6647 (membrane‐bound enzyme) (p*K*_i_ 8.7) [http://www.ncbi.nlm.nih.gov/pubmed/7703232?dopt=AbstractPlus]
Cofactors	http://www.guidetopharmacology.org/GRAC/LigandDisplayForward?ligandId=5249	http://www.guidetopharmacology.org/GRAC/LigandDisplayForward?ligandId=4786	–
Inhibitors	–	http://www.guidetopharmacology.org/GRAC/LigandDisplayForward?ligandId=6631 (p*K*_i_ 7.6) [http://www.ncbi.nlm.nih.gov/pubmed/6268095?dopt=AbstractPlus]	COMT appears to exist in both membrane‐bound and soluble forms. COMT has also been described to methylate steroids, particularly hydroxyestradiols
Selective inhibitors	http://www.guidetopharmacology.org/GRAC/LigandDisplayForward?ligandId=5116, http://www.guidetopharmacology.org/GRAC/LigandDisplayForward?ligandId=5217, http://www.guidetopharmacology.org/GRAC/LigandDisplayForward?ligandId=5150 [http://www.ncbi.nlm.nih.gov/pubmed/22384042?dopt=AbstractPlus], http://www.guidetopharmacology.org/GRAC/LigandDisplayForward?ligandId=5159	–	–
Comments	AADC is a homodimer.	–	–

**Table:** 

Nomenclature	http://www.guidetopharmacology.org/GRAC/ObjectDisplayForward?objectId=2489	http://www.guidetopharmacology.org/GRAC/ObjectDisplayForward?objectId=2490
Common abbreviation	MAO‐A	MAO‐B
HGNC, UniProt	https://www.genenames.org/data/gene‐symbol‐report/#!/hgnc_id/HGNC:6833, http://www.uniprot.org/uniprot/P21397	https://www.genenames.org/data/gene‐symbol‐report/#!/hgnc_id/HGNC:6834, http://www.uniprot.org/uniprot/P27338
EC number	http://www.genome.jp/dbget‐bin/www_bget?ec:1.4.3.4 http://www.guidetopharmacology.org/GRAC/LigandDisplayForward?ligandId=479 ‐> http://www.guidetopharmacology.org/GRAC/LigandDisplayForward?ligandId=6633 + NH_3_ http://www.guidetopharmacology.org/GRAC/LigandDisplayForward?ligandId=505 ‐> http://www.guidetopharmacology.org/GRAC/LigandDisplayForward?ligandId=6633 + NH_3_ http://www.guidetopharmacology.org/GRAC/LigandDisplayForward?ligandId=2150 ‐> http://www.guidetopharmacology.org/GRAC/LigandDisplayForward?ligandId=6635 + NH_3_ http://www.guidetopharmacology.org/GRAC/LigandDisplayForward?ligandId=940 ‐> http://www.guidetopharmacology.org/GRAC/LigandDisplayForward?ligandId=6632 + NH_3_ http://www.guidetopharmacology.org/GRAC/LigandDisplayForward?ligandId=5 ‐> http://www.guidetopharmacology.org/GRAC/LigandDisplayForward?ligandId=6634 + NH_3_	http://www.genome.jp/dbget‐bin/www_bget?ec:1.4.3.4
Cofactors	–	http://www.guidetopharmacology.org/GRAC/LigandDisplayForward?ligandId=5184 +
Inhibitors	–	http://www.guidetopharmacology.org/GRAC/LigandDisplayForward?ligandId=6641 (pIC_50_ 7.8) [http://www.ncbi.nlm.nih.gov/pubmed/11159700?dopt=AbstractPlus], http://www.guidetopharmacology.org/GRAC/LigandDisplayForward?ligandId=7266 (Irreversible inhibition) (p*K*_i_ 7.8) [http://www.ncbi.nlm.nih.gov/pubmed/18426226?dopt=AbstractPlus], http://www.guidetopharmacology.org/GRAC/LigandDisplayForward?ligandId=6640 (p*K*_i_ 7.1) [http://www.ncbi.nlm.nih.gov/pubmed/2122653?dopt=AbstractPlus, http://www.ncbi.nlm.nih.gov/pubmed/16137882?dopt=AbstractPlus], http://www.guidetopharmacology.org/GRAC/LigandDisplayForward?ligandId=6639 (p*K*_i_ 5.7–6) [http://www.ncbi.nlm.nih.gov/pubmed/15974574?dopt=AbstractPlus, http://www.ncbi.nlm.nih.gov/pubmed/21377879?dopt=AbstractPlus], http://www.guidetopharmacology.org/GRAC/LigandDisplayForward?ligandId=5281 (pIC_50_ 4.7) [http://www.ncbi.nlm.nih.gov/pubmed/15110846?dopt=AbstractPlus]
Selective inhibitors	http://www.guidetopharmacology.org/GRAC/LigandDisplayForward?ligandId=5184	http://www.guidetopharmacology.org/GRAC/LigandDisplayForward?ligandId=8291 (p*K*_i_ 6.3) [http://www.ncbi.nlm.nih.gov/pubmed/15027868?dopt=AbstractPlus]
Comments	http://www.guidetopharmacology.org/GRAC/LigandDisplayForward?ligandId=7428 (p*K*_i_ 8.3) [http://www.ncbi.nlm.nih.gov/pubmed/21680183?dopt=AbstractPlus], http://www.guidetopharmacology.org/GRAC/LigandDisplayForward?ligandId=7266 (Irreversible inhibition) (p*K*_i_ 7.3) [http://www.ncbi.nlm.nih.gov/pubmed/18426226?dopt=AbstractPlus], http://www.guidetopharmacology.org/GRAC/LigandDisplayForward?ligandId=5281 (pIC_50_ 4.7) [http://www.ncbi.nlm.nih.gov/pubmed/15110846?dopt=AbstractPlus], http://www.guidetopharmacology.org/GRAC/LigandDisplayForward?ligandId=6639 (p*K*_i_ 4.2) [http://www.ncbi.nlm.nih.gov/pubmed/21377879?dopt=AbstractPlus], http://www.guidetopharmacology.org/GRAC/LigandDisplayForward?ligandId=6637 [http://www.ncbi.nlm.nih.gov/pubmed/10333983?dopt=AbstractPlus], http://www.guidetopharmacology.org/GRAC/LigandDisplayForward?ligandId=6636, http://www.guidetopharmacology.org/GRAC/LigandDisplayForward?ligandId=6638 [http://www.ncbi.nlm.nih.gov/pubmed/9564636?dopt=AbstractPlus]	–

### Further reading on Catecholamine turnover

Bastos P *et al*. (2017) Catechol‐O‐Methyltransferase (COMT): An Update on Its Role in Cancer, Neuro‐and Cardiovascular Diseases. *Rev Biochem Pharmacol* **173**: 1–39 https://www.ncbi.nlm.nih.gov/pubmed/28456872

Deshwal S *etal*. (2017) Emerging role of monoamine oxidase as a therapeutic target for cardiovascular disease. *Curr Opin Pharmacol* **33**: 64–69 https://www.ncbi.nlm.nih.gov/pubmed/28528298?dopt=AbstractPlus

Fisar Z. (2016) Drugs related to monoamine oxidase activity. *Prog. Neuropsychopharmacol. Biol. Psychiatry* **69**: 112–24 https://www.ncbi.nlm.nih.gov/pubmed/26944656?dopt=AbstractPlus

Ramsay RR. (2016) Molecular aspects of monoamine oxidase B. *Prog. Neuropsychopharmacol. Biol. Psychiatry* **69**: 81–9 https://www.ncbi.nlm.nih.gov/pubmed/26891670?dopt=AbstractPlus

Walen K *et al*. (2017) Tyrosine and tryptophan hydroxylases as therapeutic targets in human disease. *Expert Opin. Ther. Targets* **21**: 167–180 https://www.ncbi.nlm.nih.gov/pubmed/27973928?dopt=AbstractPlus

## http://www.guidetopharmacology.org/GRAC/FamilyDisplayForward?familyId=767

### Overview

Ceramides are a family of sphingophospholipids synthesized in the endoplasmic reticulum, which mediate cell stress responses, including apoptosis, autophagy and senescence, Serine palmitoyltransferase generates http://www.guidetopharmacology.org/GRAC/LigandDisplayForward?ligandId=6654, which is reduced to http://www.guidetopharmacology.org/GRAC/LigandDisplayForward?ligandId=2453 (dihydrosphingosine). N‐Acylation allows the formation of dihydroceramides, which are subsequently reduced to form ceramides. Once synthesized, ceramides are trafficked from the ER to the Golgi bound to the ceramide transfer protein, CERT (https://www.genenames.org/data/gene‐symbol‐report/#!/hgnc_id/HGNC:2205, http://www.uniprot.org/uniprot/Q9Y5P4). Ceramide can be metabolized via multiple routes, ensuring tight regulation of its cellular levels. Addition of phosphocholine generates sphingomyelin while carbohydrate is added to form glucosyl‐ or galactosylceramides.

Ceramidase re‐forms sphingosine or sphinganine from ceramide or dihydroceramide. Phosphorylation of ceramide generates ceramide phosphate. The determination of accurate kinetic parameters for many of the enzymes in the sphingolipid metabolic pathway is complicated by the lipophilic nature of the substrates.

## http://www.guidetopharmacology.org/GRAC/FamilyDisplayForward?familyId=788

### Overview

The functional enzyme is a heterodimer of SPT1 (LCB1) with either SPT2 (LCB2) or SPT3 (LCB2B); the small subunits of SPT (ssSPTa or ssSPTb) bind to the heterodimer to enhance enzymatic activity. The complexes of SPT1/SPT2/ssSPTa and SPT1/SPT2/ssSPTb were most active with palmitoylCoA as substrate, with the latter complex also showing some activity with stearoylCoA [http://www.ncbi.nlm.nih.gov/pubmed/19416851?dopt=AbstractPlus]. Complexes involving SPT3 appeared more broad in substrate selectivity, with incorporation of myristoylCoA prominent for SPT1/SPT3/ssSPTa complexes, while SP1/SPT3/ssSPTb complexes had similar activity with C16, C18 and C20 acylCoAs [http://www.ncbi.nlm.nih.gov/pubmed/19416851?dopt=AbstractPlus].

**Table:** 

Nomenclature	http://www.guidetopharmacology.org/GRAC/ObjectDisplayForward?objectId=2509	http://www.guidetopharmacology.org/GRAC/ObjectDisplayForward?objectId=2510	http://www.guidetopharmacology.org/GRAC/ObjectDisplayForward?objectId=2511	http://www.guidetopharmacology.org/GRAC/ObjectDisplayForward?objectId=2512	http://www.guidetopharmacology.org/GRAC/ObjectDisplayForward?objectId=2513
Common abbreviation	SPT1	SPT2	SPT3	SPTSSA	SPTSSB
HGNC, UniProt	https://www.genenames.org/data/gene‐symbol‐report/#!/hgnc_id/HGNC:11277, http://www.uniprot.org/uniprot/O15269	https://www.genenames.org/data/gene‐symbol‐report/#!/hgnc_id/HGNC:11278, http://www.uniprot.org/uniprot/O15270	https://www.genenames.org/data/gene‐symbol‐report/#!/hgnc_id/HGNC:16253, http://www.uniprot.org/uniprot/Q9NUV7	https://www.genenames.org/data/gene‐symbol‐report/#!/hgnc_id/HGNC:20361, http://www.uniprot.org/uniprot/Q969W0	https://www.genenames.org/data/gene‐symbol‐report/#!/hgnc_id/HGNC:24045, http://www.uniprot.org/uniprot/Q8NFR3
EC number	http://www.genome.jp/dbget‐bin/www_bget?ec:2.3.1.50: http://www.guidetopharmacology.org/GRAC/LigandDisplayForward?ligandId=726 + http://www.guidetopharmacology.org/GRAC/LigandDisplayForward?ligandId=6765 ‐*>* http://www.guidetopharmacology.org/GRAC/LigandDisplayForward?ligandId=6654+ http://www.guidetopharmacology.org/GRAC/LigandDisplayForward?ligandId=3044 + CO_2_	http://www.genome.jp/dbget‐bin/www_bget?ec:2.3.1.50: http://www.guidetopharmacology.org/GRAC/LigandDisplayForward?ligandId=726 + http://www.guidetopharmacology.org/GRAC/LigandDisplayForward?ligandId=6765 ‐*>* http://www.guidetopharmacology.org/GRAC/LigandDisplayForward?ligandId=6654 + http://www.guidetopharmacology.org/GRAC/LigandDisplayForward?ligandId=3044 + CO_2_	http://www.genome.jp/dbget‐bin/www_bget?ec:2.3.1.50: http://www.guidetopharmacology.org/GRAC/LigandDisplayForward?ligandId=726 + http://www.guidetopharmacology.org/GRAC/LigandDisplayForward?ligandId=6765 ‐*>* http://www.guidetopharmacology.org/GRAC/LigandDisplayForward?ligandId=6654+ http://www.guidetopharmacology.org/GRAC/LigandDisplayForward?ligandId=3044 + CO_2_	–	–
Cofactors	http://www.guidetopharmacology.org/GRAC/LigandDisplayForward?ligandId=5249	http://www.guidetopharmacology.org/GRAC/LigandDisplayForward?ligandId=5249	http://www.guidetopharmacology.org/GRAC/LigandDisplayForward?ligandId=5249	–	–
Selective inhibitors	http://www.guidetopharmacology.org/GRAC/LigandDisplayForward?ligandId=6664 (p*K*_i_ 9.6) [http://www.ncbi.nlm.nih.gov/pubmed/7794249?dopt=AbstractPlus] – Mouse	http://www.guidetopharmacology.org/GRAC/LigandDisplayForward?ligandId=6664 [http://www.ncbi.nlm.nih.gov/pubmed/7794249?dopt=AbstractPlus]	http://www.guidetopharmacology.org/GRAC/LigandDisplayForward?ligandId=6664 [http://www.ncbi.nlm.nih.gov/pubmed/7794249?dopt=AbstractPlus]	–	–

## http://www.guidetopharmacology.org/GRAC/FamilyDisplayForward?familyId=789

### Overview

This family of enzymes, also known as sphingosine *N*‐acyltransferase, is located in the ER facing the cytosol with an as‐yet undefined topology and stoichiometry. Ceramide synthase *in vitro* is sensitive to inhibition by the fungal derived toxin, fumonisin B1.

**Table:** 

Nomenclature	http://www.guidetopharmacology.org/GRAC/ObjectDisplayForward?objectId=2474	http://www.guidetopharmacology.org/GRAC/ObjectDisplayForward?objectId=2475	http://www.guidetopharmacology.org/GRAC/ObjectDisplayForward?objectId=2476	http://www.guidetopharmacology.org/GRAC/ObjectDisplayForward?objectId=2477	http://www.guidetopharmacology.org/GRAC/ObjectDisplayForward?objectId=2478	http://www.guidetopharmacology.org/GRAC/ObjectDisplayForward?objectId=2479
Common abbreviation	CERS1	CERS2	CERS3	CERS4	CERS5	CERS6
HGNC, UniProt	https://www.genenames.org/data/gene‐symbol‐report/#!/hgnc_id/HGNC:14253, http://www.uniprot.org/uniprot/P27544	https://www.genenames.org/data/gene‐symbol‐report/#!/hgnc_id/HGNC:14076, http://www.uniprot.org/uniprot/Q96G23	https://www.genenames.org/data/gene‐symbol‐report/#!/hgnc_id/HGNC:23752, http://www.uniprot.org/uniprot/Q8IU89	https://www.genenames.org/data/gene‐symbol‐report/#!/hgnc_id/HGNC:23747, http://www.uniprot.org/uniprot/Q9HA82	https://www.genenames.org/data/gene‐symbol‐report/#!/hgnc_id/HGNC:23749, http://www.uniprot.org/uniprot/Q8N5B7	https://www.genenames.org/data/gene‐symbol‐report/#!/hgnc_id/HGNC:23826, http://www.uniprot.org/uniprot/Q6ZMG9
EC number	http://www.genome.jp/dbget‐bin/www_bget?ec:2.3.1.24: acylCoA+ http://www.guidetopharmacology.org/GRAC/LigandDisplayForward?ligandId=2453 ‐*>* dihydroceramide + http://www.guidetopharmacology.org/GRAC/LigandDisplayForward?ligandId=3044 http://www.guidetopharmacology.org/GRAC/LigandDisplayForward?ligandId=2452 + acylCoA ‐*>* ceramide + http://www.guidetopharmacology.org/GRAC/LigandDisplayForward?ligandId=3044	http://www.genome.jp/dbget‐bin/www_bget?ec:2.3.1.24: acylCoA + http://www.guidetopharmacology.org/GRAC/LigandDisplayForward?ligandId=2453 ‐*>* dihydroceramide + http://www.guidetopharmacology.org/GRAC/LigandDisplayForward?ligandId=3044 http://www.guidetopharmacology.org/GRAC/LigandDisplayForward?ligandId=2452 + acylCoA ‐*>* ceramide + http://www.guidetopharmacology.org/GRAC/LigandDisplayForward?ligandId=3044	http://www.genome.jp/dbget‐bin/www_bget?ec:2.3.1.24: acylCoA + http://www.guidetopharmacology.org/GRAC/LigandDisplayForward?ligandId=2453 ‐*>* dihydroceramide + http://www.guidetopharmacology.org/GRAC/LigandDisplayForward?ligandId=3044 http://www.guidetopharmacology.org/GRAC/LigandDisplayForward?ligandId=2452 + acylCoA ‐*>* ceramide + http://www.guidetopharmacology.org/GRAC/LigandDisplayForward?ligandId=3044	http://www.genome.jp/dbget‐bin/www_bget?ec:2.3.1.24: acylCoA + http://www.guidetopharmacology.org/GRAC/LigandDisplayForward?ligandId=2453 ‐*>* dihydroceramide + http://www.guidetopharmacology.org/GRAC/LigandDisplayForward?ligandId=3044 http://www.guidetopharmacology.org/GRAC/LigandDisplayForward?ligandId=2452 + acylCoA ‐*>* ceramide + http://www.guidetopharmacology.org/GRAC/LigandDisplayForward?ligandId=3044	http://www.genome.jp/dbget‐bin/www_bget?ec:2.3.1.24: acylCoA + http://www.guidetopharmacology.org/GRAC/LigandDisplayForward?ligandId=2453 ‐*>* dihydroceramide + http://www.guidetopharmacology.org/GRAC/LigandDisplayForward?ligandId=3044 http://www.guidetopharmacology.org/GRAC/LigandDisplayForward?ligandId=2452 + acylCoA ‐*>* ceramide + http://www.guidetopharmacology.org/GRAC/LigandDisplayForward?ligandId=3044	http://www.genome.jp/dbget‐bin/www_bget?ec:2.3.1.24: acylCoA + http://www.guidetopharmacology.org/GRAC/LigandDisplayForward?ligandId=2453 ‐*>* dihydroceramide + http://www.guidetopharmacology.org/GRAC/LigandDisplayForward?ligandId=3044 http://www.guidetopharmacology.org/GRAC/LigandDisplayForward?ligandId=2452 + acylCoA ‐*>* ceramide + http://www.guidetopharmacology.org/GRAC/LigandDisplayForward?ligandId=3044
Substrates	C18‐CoA [http://www.ncbi.nlm.nih.gov/pubmed/12105227?dopt=AbstractPlus]	C24‐ and C26‐CoA [http://www.ncbi.nlm.nih.gov/pubmed/18165233?dopt=AbstractPlus]	C26‐CoA and longer [http://www.ncbi.nlm.nih.gov/pubmed/14511335?dopt=AbstractPlus, http://www.ncbi.nlm.nih.gov/pubmed/18308723?dopt=AbstractPlus]	C18‐, C20‐ and C22‐CoA [http://www.ncbi.nlm.nih.gov/pubmed/12912983?dopt=AbstractPlus]	C16‐CoA [http://www.ncbi.nlm.nih.gov/pubmed/16100120?dopt=AbstractPlus, http://www.ncbi.nlm.nih.gov/pubmed/12912983?dopt=AbstractPlus]	C14‐ and C16‐CoA [http://www.ncbi.nlm.nih.gov/pubmed/15823095?dopt=AbstractPlus]

## http://www.guidetopharmacology.org/GRAC/FamilyDisplayForward?familyId=790

### Overview

DEGS1 and DEGS2 are 4TM proteins.

**Table:** 

Nomenclature	http://www.guidetopharmacology.org/GRAC/ObjectDisplayForward?objectId=2484	http://www.guidetopharmacology.org/GRAC/ObjectDisplayForward?objectId=2485
HGNC, UniProt	https://www.genenames.org/data/gene‐symbol‐report/#!/hgnc_id/HGNC:13709, http://www.uniprot.org/uniprot/O15121	https://www.genenames.org/data/gene‐symbol‐report/#!/hgnc_id/HGNC:20113, http://www.uniprot.org/uniprot/Q6QHC5
EC number	http://www.genome.jp/dbget‐bin/www_bget?ec:1.14.‐.‐	http://www.genome.jp/dbget‐bin/www_bget?ec:1.14.‐.‐
Cofactors	http://www.guidetopharmacology.org/GRAC/LigandDisplayForward?ligandId=2451	http://www.guidetopharmacology.org/GRAC/LigandDisplayForward?ligandId=2451
Inhibitors	http://www.guidetopharmacology.org/GRAC/LigandDisplayForward?ligandId=6041 (p*K*_i_ 6.5) [http://www.ncbi.nlm.nih.gov/pubmed/24875537?dopt=AbstractPlus], http://www.guidetopharmacology.org/GRAC/LigandDisplayForward?ligandId=8879 (pIC_50_ 4.7) [http://www.ncbi.nlm.nih.gov/pubmed/22537678?dopt=AbstractPlus]	–
Comments	Myristoylation of DEGS1 enhances its activity and targets it to the mitochondria [http://www.ncbi.nlm.nih.gov/pubmed/19647031?dopt=AbstractPlus].	–

### Comments

DEGS1 activity is inhibited by a number of natural products, including http://www.guidetopharmacology.org/GRAC/LigandDisplayForward?ligandId=7000 and http://www.guidetopharmacology.org/GRAC/LigandDisplayForward?ligandId=2424 [http://www.ncbi.nlm.nih.gov/pubmed/22200621?dopt=AbstractPlus].

## http://www.guidetopharmacology.org/GRAC/FamilyDisplayForward?familyId=774

### Overview

Following translocation from the ER to the Golgi under the influence of the ceramide transfer protein, sphingomyelin synthases allow the formation of sphingomyelin by the transfer of phosphocholine from the phospholipid phosphatidylcholine. Sphingomyelin synthase‐related protein 1 is structurally related but lacks sphingomyelin synthase activity.

**Table:** 

Nomenclature	http://www.guidetopharmacology.org/GRAC/ObjectDisplayForward?objectId=2520	http://www.guidetopharmacology.org/GRAC/ObjectDisplayForward?objectId=2521	http://www.guidetopharmacology.org/GRAC/ObjectDisplayForward?objectId=2525
HGNC, UniProt	https://www.genenames.org/data/gene‐symbol‐report/#!/hgnc_id/HGNC:29799, http://www.uniprot.org/uniprot/Q86VZ5	https://www.genenames.org/data/gene‐symbol‐report/#!/hgnc_id/HGNC:28395, http://www.uniprot.org/uniprot/Q8NHU3	https://www.genenames.org/data/gene‐symbol‐report/#!/hgnc_id/HGNC:26320, http://www.uniprot.org/uniprot/Q96LT4
EC number	http://www.genome.jp/dbget‐bin/www_bget?ec:2.7.8.27: ceramide + phosphatidylcholine ‐*>* sphingomyelin + diacylglycerol	http://www.genome.jp/dbget‐bin/www_bget?ec:2.7.8.27: ceramide + phosphatidylcholine ‐*>* sphingomyelin + diacylglycerol	http://www.genome.jp/dbget‐bin/www_bget?ec:2.7.8.‐: ceramide + http://www.guidetopharmacology.org/GRAC/LigandDisplayForward?ligandId=2796 ‐*>* ceramide phosphoethanolamine
Inhibitors	http://www.guidetopharmacology.org/GRAC/LigandDisplayForward?ligandId=8835 (pIC_50_ 5.7) [http://www.ncbi.nlm.nih.gov/pubmed/26314925?dopt=AbstractPlus]	http://www.guidetopharmacology.org/GRAC/LigandDisplayForward?ligandId=8834 (pIC_50_ 4.9) [http://www.ncbi.nlm.nih.gov/pubmed/24374347?dopt=AbstractPlus]	–
Comments	–	Palmitoylation of sphingomyelin synthase 2 may allow targeting to the plasma membrane [http://www.ncbi.nlm.nih.gov/pubmed/19233134?dopt=AbstractPlus].	–

## http://www.guidetopharmacology.org/GRAC/FamilyDisplayForward?familyId=773

### Overview

Also known as sphingomyelinase.

**Table:** 

Nomenclature	http://www.guidetopharmacology.org/GRAC/ObjectDisplayForward?objectId=2514	http://www.guidetopharmacology.org/GRAC/ObjectDisplayForward?objectId=2515	http://www.guidetopharmacology.org/GRAC/ObjectDisplayForward?objectId=2516	http://www.guidetopharmacology.org/GRAC/ObjectDisplayForward?objectId=2517	http://www.guidetopharmacology.org/GRAC/ObjectDisplayForward?objectId=2518	http://www.guidetopharmacology.org/GRAC/ObjectDisplayForward?objectId=2519
HGNC, UniProt	https://www.genenames.org/data/gene‐symbol‐report/#!/hgnc_id/HGNC:11120, http://www.uniprot.org/uniprot/P17405	https://www.genenames.org/data/gene‐symbol‐report/#!/hgnc_id/HGNC:11121, http://www.uniprot.org/uniprot/O60906	https://www.genenames.org/data/gene‐symbol‐report/#!/hgnc_id/HGNC:14240, http://www.uniprot.org/uniprot/Q9NY59	https://www.genenames.org/data/gene‐symbol‐report/#!/hgnc_id/HGNC:32949, http://www.uniprot.org/uniprot/Q9NXE4	https://www.genenames.org/data/gene‐symbol‐report/#!/hgnc_id/HGNC:17389, http://www.uniprot.org/uniprot/Q92484	https://www.genenames.org/data/gene‐symbol‐report/#!/hgnc_id/HGNC:21416, http://www.uniprot.org/uniprot/Q92485
EC number	http://www.genome.jp/dbget‐bin/www_bget?ec:3.1.4.12: sphingomyelin ‐*>* ceramide + phosphocholine	http://www.genome.jp/dbget‐bin/www_bget?ec:3.1.4.12: sphingomyelin ‐*>* ceramide + phosphocholine	http://www.genome.jp/dbget‐bin/www_bget?ec:3.1.4.12: sphingomyelin ‐*>* ceramide + phosphocholine	http://www.genome.jp/dbget‐bin/www_bget?ec:3.1.4.12: sphingomyelin ‐*>* ceramide + phosphocholine	http://www.genome.jp/dbget‐bin/www_bget?ec:3.1.4.‐: sphingomyelin ‐*>* ceramide + phosphocholine	http://www.genome.jp/dbget‐bin/www_bget?ec:3.1.4.‐: sphingomyelin ‐*>* ceramide + phosphocholine
Inhibitors	–	http://www.guidetopharmacology.org/GRAC/LigandDisplayForward?ligandId=8847 (p*K*_i_ 5.8) [http://www.ncbi.nlm.nih.gov/pubmed/12482429?dopt=AbstractPlus] – Bovine	–	–	–	–

## http://www.guidetopharmacology.org/GRAC/FamilyDisplayForward?familyId=772

### Overview

Protein FAN [http://www.ncbi.nlm.nih.gov/pubmed/8808629?dopt=AbstractPlus] and polycomb protein EED [http://www.ncbi.nlm.nih.gov/pubmed/20080539?dopt=AbstractPlus] allow coupling between TNF receptors and neutral sphingomyelinase phosphodiesterases.

**Table:** 

Nomenclature	http://www.guidetopharmacology.org/GRAC/ObjectDisplayForward?objectId=2487	http://www.guidetopharmacology.org/GRAC/ObjectDisplayForward?objectId=2495
HGNC, UniProt	https://www.genenames.org/data/gene‐symbol‐report/#!/hgnc_id/HGNC:3188, http://www.uniprot.org/uniprot/O75530	https://www.genenames.org/data/gene‐symbol‐report/#!/hgnc_id/HGNC:8017, http://www.uniprot.org/uniprot/Q92636
Selective inhibitors	http://www.guidetopharmacology.org/GRAC/LigandDisplayForward?ligandId=9525 (Binding) (p*K*_i_ 9.4) [http://www.ncbi.nlm.nih.gov/pubmed/28135237?dopt=AbstractPlus]	–

## http://www.guidetopharmacology.org/GRAC/FamilyDisplayForward?familyId=775

**Table:** 

Nomenclature	http://www.guidetopharmacology.org/GRAC/ObjectDisplayForward?objectId=2528
HGNC, UniProt	https://www.genenames.org/data/gene‐symbol‐report/#!/hgnc_id/HGNC:12524, http://www.uniprot.org/uniprot/Q16739
EC number	http://www.genome.jp/dbget‐bin/www_bget?ec:2.4.1.80: http://www.guidetopharmacology.org/GRAC/LigandDisplayForward?ligandId=1783 + ceramide = http://www.guidetopharmacology.org/GRAC/LigandDisplayForward?ligandId=1749 + glucosylceramide
Inhibitors	http://www.guidetopharmacology.org/GRAC/LigandDisplayForward?ligandId=4841 (p*K*_i_ 5.1) [68]
Comments	Glycoceramides are an extended family of sphingolipids, differing in the content and organization of the sugar moieties, as well as the acyl sidechains.

## http://www.guidetopharmacology.org/GRAC/FamilyDisplayForward?familyId=769

### Overview

The six human ceramidases may be divided on the basis of pH optimae into acid, neutral and alkaline ceramidases, which also differ in their subcellular location.

**Table:** 

Nomenclature	http://www.guidetopharmacology.org/GRAC/ObjectDisplayForward?objectId=2491
HGNC, UniProt	https://www.genenames.org/data/gene‐symbol‐report/#!/hgnc_id/HGNC:735, http://www.uniprot.org/uniprot/Q13510
EC number	http://www.genome.jp/dbget‐bin/www_bget?ec:3.5.1.23: ceramide ‐*>* http://www.guidetopharmacology.org/GRAC/LigandDisplayForward?ligandId=2452 + a fatty acid
Comments	This lysosomal enzyme is proteolysed to form the mature protein made up of two chains from the same gene product [http://www.ncbi.nlm.nih.gov/pubmed/8955159?dopt=AbstractPlus].

## http://www.guidetopharmacology.org/GRAC/FamilyDisplayForward?familyId=770

### Overview

The six human ceramidases may be divided on the basis of pH optimae into acid, neutral and alkaline ceramidases, which also differ in their subcellular location.

**Table:** 

Nomenclature	http://www.guidetopharmacology.org/GRAC/ObjectDisplayForward?objectId=2492	http://www.guidetopharmacology.org/GRAC/ObjectDisplayForward?objectId=2493
HGNC, UniProt	https://www.genenames.org/data/gene‐symbol‐report/#!/hgnc_id/HGNC:18860, http://www.uniprot.org/uniprot/Q9NR71	https://www.genenames.org/data/gene‐symbol‐report/#!/hgnc_id/HGNC:23456, http://www.uniprot.org/uniprot/P0C7U1
EC number	http://www.genome.jp/dbget‐bin/www_bget?ec:3.5.1.23: ceramide ‐> http://www.guidetopharmacology.org/GRAC/LigandDisplayForward?ligandId=2452 + a fatty acid	–
Comments	The enzyme is associated with the plasma membrane [http://www.ncbi.nlm.nih.gov/pubmed/12499379?dopt=AbstractPlus].	–

### Comments

ASAH2B appears to be an enzymatically inactive protein, which may result from gene duplication and truncation.

## http://www.guidetopharmacology.org/GRAC/FamilyDisplayForward?familyId=768

### Overview

The six human ceramidases may be divided on the basis of pH optimae into acid, neutral and alkaline ceramidases, which also differ in their subcellular location.

**Table:** 

Nomenclature	http://www.guidetopharmacology.org/GRAC/ObjectDisplayForward?objectId=2468	http://www.guidetopharmacology.org/GRAC/ObjectDisplayForward?objectId=2469	http://www.guidetopharmacology.org/GRAC/ObjectDisplayForward?objectId=2470
HGNC, UniProt	https://www.genenames.org/data/gene‐symbol‐report/#!/hgnc_id/HGNC:18356, http://www.uniprot.org/uniprot/Q8TDN7	https://www.genenames.org/data/gene‐symbol‐report/#!/hgnc_id/HGNC:23675, http://www.uniprot.org/uniprot/Q5QJU3	https://www.genenames.org/data/gene‐symbol‐report/#!/hgnc_id/HGNC:16066, http://www.uniprot.org/uniprot/Q9NUN7
EC number	http://www.genome.jp/dbget‐bin/www_bget?ec:3.5.1.23: ceramide ‐> http://www.guidetopharmacology.org/GRAC/LigandDisplayForward?ligandId=2452 + a fatty acid	http://www.genome.jp/dbget‐bin/www_bget?ec:3.5.1.23: ceramide ‐> http://www.guidetopharmacology.org/GRAC/LigandDisplayForward?ligandId=2452 + a fatty acid	http://www.genome.jp/dbget‐bin/www_bget?ec:3.5.1.‐
Comments	ACER1 is associated with the ER [http://www.ncbi.nlm.nih.gov/pubmed/17713573?dopt=AbstractPlus].	ACER2 is associated with the Golgi apparatus [http://www.ncbi.nlm.nih.gov/pubmed/16940153?dopt=AbstractPlus].	ACER3 is associated with the ER and Golgi apparatus [http://www.ncbi.nlm.nih.gov/pubmed/11356846?dopt=AbstractPlus].

## http://www.guidetopharmacology.org/GRAC/FamilyDisplayForward?familyId=771

**Table:** 

Nomenclature	http://www.guidetopharmacology.org/GRAC/FamilyDisplayForward?familyId=771
HGNC, UniProt	https://www.genenames.org/data/gene‐symbol‐report/#!/hgnc_id/HGNC:19256, http://www.uniprot.org/uniprot/Q8TCT0
EC number	http://www.genome.jp/dbget‐bin/www_bget?ec:2.7.1.138: ceramide + http://www.guidetopharmacology.org/GRAC/LigandDisplayForward?ligandId=1713 ‐> ceramide 1‐phosphate + http://www.guidetopharmacology.org/GRAC/LigandDisplayForward?ligandId=1712
Inhibitors	http://www.guidetopharmacology.org/GRAC/LigandDisplayForward?ligandId=6668 (pIC_50_ 7.9) [http://www.ncbi.nlm.nih.gov/pubmed/18612076?dopt=AbstractPlus]

### Comments

A ceramide kinase‐like protein has been identified in the human genome (https://www.genenames.org/data/gene‐symbol‐report/#!/hgnc_id/HGNC:21699, http://www.uniprot.org/uniprot/Q49MI3).

### Further reading on Ceramide turnover

Brachtendorf S et al. (2019) Ceramide synthases in cancer therapy and chemoresistance. *Prog Lipid Res* **74**: 160‐185 https://www.ncbi.nlm.nih.gov/pubmed/30953657

Chen Y and Cao Y. (2017) The sphingomyelin synthase family: proteins, diseases, and inhibitors. *Biol Chem* **398**: 1319‐1325 https://www.ncbi.nlm.nih.gov/pubmed/28742512

Fang Z et al. (2019) Ceramide and sphingosine 1‐phosphate in adipose dysfunction. *Prog Lipid Res* **74**: 145‐159 https://www.ncbi.nlm.nih.gov/pubmed/30951736

Hernández‐Corbacho MJ et al. (2017) Sphingolipids in mitochondria. *Biochim Biophys Acta* **1862**: 56‐68 https://www.ncbi.nlm.nih.gov/pubmed/27697478?dopt=AbstractPlus

Ilan Y. (2016) Compounds of the sphingomyelin‐ceramide‐glycosphingolipid pathways as secondary messenger molecules: new targets for novel therapies for fatty liver disease and insulin resistance. *Am. J. Physiol. Gastrointest. Liver Physiol*. **310**: G1102‐17 https://www.ncbi.nlm.nih.gov/pubmed/27173510?dopt=AbstractPlus

Iqbal J et al. (2017) Sphingolipids and Lipoproteins in Health and Metabolic Disorders. *Trends Endocrinol. Metab*. **28**: 506‐518 https://www.ncbi.nlm.nih.gov/pubmed/28462811?dopt=AbstractPlus

Kihara A. (2016) Synthesis and degradation pathways, functions, and pathology of ceramides and epidermal acylceramides. *Prog. Lipid Res*. **63**: 50‐69 https://www.ncbi.nlm.nih.gov/pubmed/27107674?dopt=AbstractPlus

Ogretmen B (2018) Sphingolipid metabolism in cancer signalling and therapy. *Nat Rev Cancer* **18**: 33‐50 https://www.ncbi.nlm.nih.gov/pubmed/29147025

Parashuraman S and D’Angelo. (2019) Visualizing sphingolipid biosynthesis in cells. *Chem Phys Lipids* **218**: 103‐111 https://www.ncbi.nlm.nih.gov/pubmed/30476485

Rodriguez‐Cuenca S et al. (2017) Sphingolipids and glycerophospholipids ‐ The “ying and yang” of lipotoxicity in metabolic diseases. *Prog. Lipid Res*. **66**: 14‐29 https://www.ncbi.nlm.nih.gov/pubmed/28104532?dopt=AbstractPlus

Snider et al. (2019) Approaches for probing and evaluating mammalian sphingolipid metabolism. *Anal Biochem* **575**: 70‐86 https://www.ncbi.nlm.nih.gov/pubmed/30917945

Vogt D et al. (2017) Therapeutic Strategies and Pharmacological Tools Influencing S1P Signaling and Metabolism. *Med Res Rev* **37**: 3‐51 https://www.ncbi.nlm.nih.gov/pubmed/27480072?dopt=AbstractPlus

Wegner MS et al. (2016) The enigma of ceramide synthase regulation in mammalian cells. *Prog. Lipid Res*. **63**: 93‐119 https://www.ncbi.nlm.nih.gov/pubmed/27180613?dopt=AbstractPlus

## http://www.guidetopharmacology.org/GRAC/FamilyDisplayForward?familyId=865

### Overview

Chromatin modifying enzymes, and other chromatin‐modifying proteins, fall into three broad categories: **writers**, **readers** and **erasers**. The function of these proteins is to dynamically maintain cell identity and regulate processes such as differentiation, development, proliferation and genome integrity *via* recognition of specific ’marks’ (covalent post‐translational modifications) on histone proteins and DNA [http://www.ncbi.nlm.nih.gov/pubmed/17320507?dopt=AbstractPlus]. In normal cells, tissues and organs, precise co‐ordination of these proteins ensures expression of only those genes required to specify phenotype or which are required at specific times, for specific functions. Chromatin modifications allow DNA modifications not coded by the DNA sequence to be passed on through the genome and underlies heritable phenomena such as X chromosome inactivation, aging, heterochromatin formation, reprogramming, and gene silencing (epigenetic control).

To date at least eight distinct types of modifications are found on histones. These include small covalent modifications such as acetylation, methylation, and phosphorylation, the attachment of larger modifiers such as ubiquitination or sumoylation, and ADP ribosylation, proline isomerization and deimination. Chromatin modifications and the functions they regulate in cells are reviewed by Kouzarides (2007) [http://www.ncbi.nlm.nih.gov/pubmed/17320507?dopt=AbstractPlus].

**Writer** proteins include the histone methyltransferases, histone acetyltransferases, some kinases and ubiquitin ligases.

**Readers** include proteins which contain methyl‐lysinerecognition motifs such as bromodomains, chromodomains, tudor domains, PHD zinc fingers, PWWP domains and MBT domains.

**Erasers** include the histone demethylases and histone deacetylases (HDACs and sirtuins).

Dysregulated epigenetic control can be associated with human diseases such as cancer [http://www.ncbi.nlm.nih.gov/pubmed/18337604?dopt=AbstractPlus], where awide variety of cellular and protein abberations are known to perturb chromatin structure, gene transcription and ultimately cellular pathways [http://www.ncbi.nlm.nih.gov/pubmed/21941284?dopt=AbstractPlus, http://www.ncbi.nlm.nih.gov/pubmed/24104525?dopt=AbstractPlus]. Due to the reversible nature of epigenetic modifications, chromatin regulators are very tractable targets for drug discovery and the development of novel therapeutics. Indeed, small molecule inhibitors of writers (*e.g*. http://www.guidetopharmacology.org/GRAC/LigandDisplayForward?ligandId=6796 and http://www.guidetopharmacology.org/GRAC/LigandDisplayForward?ligandId=6805 target the DNA methyltransferases DNMT1 and DNMT3 for the treatment of myelodysplastic syndromes [http://www.ncbi.nlm.nih.gov/pubmed/21220589?dopt=AbstractPlus, http://www.ncbi.nlm.nih.gov/pubmed/24523604?dopt=AbstractPlus]) and erasers (*e.g*. the HDAC inhibitors http://www.guidetopharmacology.org/GRAC/LigandDisplayForward?ligandId=6852, http://www.guidetopharmacology.org/GRAC/LigandDisplayForward?ligandId=7006 and http://www.guidetopharmacology.org/GRAC/LigandDisplayForward?ligandId=7496 for the treatment of T‐cell lymphomas [http://www.ncbi.nlm.nih.gov/pubmed/21493798?dopt=AbstractPlus, http://www.ncbi.nlm.nih.gov/pubmed/22124371?dopt=AbstractPlus]) are already being used in the clinic. The search for the next generation of compounds with improved specificity against chromatin‐associated proteins is an area of intense basic and clinical research [http://www.ncbi.nlm.nih.gov/pubmed/25974248?dopt=AbstractPlus]. Current progress in this field is reviewed by Simó‐Riudalbas and Esteller (2015) [http://www.ncbi.nlm.nih.gov/pubmed/25039449?dopt=AbstractPlus].

## http://www.guidetopharmacology.org/GRAC/FamilyDisplayForward?familyId=254

### Overview

Protein arginine N‐methyltransferases (PRMT, EC 2.1.1.‐) encompass histone arginine N‐methyltransferases (PRMT4, PRMT7, http://www.genome.jp/kegg‐bin/search_brite?option=‐a&search_string=2.1.1.125) and myelin basic protein *N*‐methyltransferases (PRMT7, http://www.genome.jp/kegg‐bin/search_brite?option=‐a&search_string=2.1.1.126). They are dimeric or tetrameric enzymes which use http://www.guidetopharmacology.org/GRAC/LigandDisplayForward?ligandId=4786 as a methyl donor, generating http://www.guidetopharmacology.org/GRAC/LigandDisplayForward?ligandId=5265 as a by‐product. They generate both mono‐methylated and dimethylated products; these may be symmetric (http://www.guidetopharmacology.org/GRAC/LigandDisplayForward?ligandId=5271) or asym metric (http://www.guidetopharmacology.org/GRAC/LigandDisplayForward?ligandId=5229) versions, where both guanidine nitrogens are monomethylated or one of the two is dimethylated, respectively.

Information on members of this family may be found in the http://www.guidetopharmacology.org/GRAC/FamilyDisplayForward?familyId=254.

## http://www.guidetopharmacology.org/GRAC/FamilyDisplayForward?familyId=848

### Overview

Histone deacetylases act as erasers of epigenetic acetylation marks on lysine residues in histones. Removal of the acetyl groups facilitates tighter packing of chromatin (heterochromatin formation) leading to transcriptional repression.

The histone deacetylase family has been classified in to five subfamilies based on phylogenetic comparison with yeast homologues:

Class I contains HDACs 1, 2, 3 and 8

Class IIa contains HDACs 4, 5, 7 and 9

Class IIb contains HDACs 6 and 10

Class III contains the sirtuins (SIRT1‐7)

Class IV contains only HDAC11.

Classes I, II and IV use Zn^+^ as a co‐factor, whereas catalysis by Class III enzymes requires NAD^+^ as a co‐factor, and members of this subfamily have ADP‐ribosylase activity in addition to protein deacetylase function [http://www.ncbi.nlm.nih.gov/pubmed/20132909?dopt=AbstractPlus].

HDACs have more general protein deacetylase activity, being able to deacetylate lysine residues in non‐histone proteins [http://www.ncbi.nlm.nih.gov/pubmed/19608861?dopt=AbstractPlus] such as microtubules [http://www.ncbi.nlm.nih.gov/pubmed/12024216?dopt=AbstractPlus], the hsp90 chaperone [http://www.ncbi.nlm.nih.gov/pubmed/15916966?dopt=AbstractPlus] and the tumour suppressor p53 [http://www.ncbi.nlm.nih.gov/pubmed/11099047?dopt=AbstractPlus].

Dysregulated HDACactivity has been identified in cancer cells and tumour tissues [http://www.ncbi.nlm.nih.gov/pubmed/11704848?dopt=AbstractPlus, http://www.ncbi.nlm.nih.gov/pubmed/19383284?dopt=AbstractPlus], making HDACs attractive molecular targets in the search for novel mechanisms to treat cancer [http://www.ncbi.nlm.nih.gov/pubmed/24382387?dopt=AbstractPlus]. Several small molecule HDAC inhibitors are already approved for clinical use: http://www.guidetopharmacology.org/GRAC/LigandDisplayForward?ligandId=7006, http://www.guidetopharmacology.org/GRAC/LigandDisplayForward?ligandId=7496, http://www.guidetopharmacology.org/GRAC/LigandDisplayForward?ligandId=6852, http://www.guidetopharmacology.org/GRAC/LigandDisplayForward?ligandId=7489, http://www.guidetopharmacology.org/GRAC/LigandDisplayForward?ligandId=7496, http://www.guidetopharmacology.org/GRAC/LigandDisplayForward?ligandId=7009 and http://www.guidetopharmacology.org/GRAC/LigandDisplayForward?ligandId=8305. HDACs and HDAC inhibitors currently in development as potential anti‐cancer therapeutics are reviewed by Simó‐Riudalbas and Esteller (2015) [http://www.ncbi.nlm.nih.gov/pubmed/25039449?dopt=AbstractPlus].

**Table:** 

Nomenclature	http://www.guidetopharmacology.org/GRAC/ObjectDisplayForward?objectId=2618
HGNC, UniProt	https://www.genenames.org/data/gene‐symbol‐report/#!/hgnc_id/HGNC:14064, http://www.uniprot.org/uniprot/Q9UBN7
EC number	http://www.genome.jp/dbget‐bin/www_bget?ec:3.5.1.98
Inhibitors	http://www.guidetopharmacology.org/GRAC/LigandDisplayForward?ligandId=7005 (p*K*_i_ 9) [http://www.ncbi.nlm.nih.gov/pubmed/20139990?dopt=AbstractPlus], http://www.guidetopharmacology.org/GRAC/LigandDisplayForward?ligandId=6852 (p*K*_i_ 8.8) [http://www.ncbi.nlm.nih.gov/pubmed/20139990?dopt=AbstractPlus], romidepsin (p*K*_i_ 8) [http://www.ncbi.nlm.nih.gov/pubmed/20139990?dopt=AbstractPlus]
Selective inhibitors	http://www.guidetopharmacology.org/GRAC/LigandDisplayForward?ligandId=7010 (pIC_50_ 8.3) [http://www.ncbi.nlm.nih.gov/pubmed/22262760?dopt=AbstractPlus]

### Further reading on 3.5.1.‐ Histone deacetylases (HDACs)

Ellmeier W *et al*. (2018) Histone deacetylase function in CD4^+^ T cells. *Nat. Rev. Immunol*. **18**: 617‐634 https://www.ncbi.nlm.nih.gov/pubmed/30022149?dopt=AbstractPlus

Maolanon AR *et al*. (2017) Natural and Synthetic Macrocyclic Inhibitors of the Histone Deacetylase Enzymes. *Chembiochem* **18**: 5‐49 https://www.ncbi.nlm.nih.gov/pubmed/27748555?dopt=AbstractPlus

Micelli C *et al*. (2015) Histone deacetylases: structural determinants of inhibitor selectivity. *Drug Discov Today* **20**: 718‐35 https://www.ncbi.nlm.nih.gov/pubmed/25687212?dopt=AbstractPlus

Millard CJ *et al*. (2017) Targeting Class I Histone Deacetylases in a "Complex" Environment. *Trends Pharmacol Sci* **38**: 363‐377 https://www.ncbi.nlm.nih.gov/pubmed/28139258?dopt=AbstractPlus

Roche J *et al*. (2016) Inside HDACs with more selective HDAC inhibitors. *Eur J Med Chem* **121**: 451‐483 https://www.ncbi.nlm.nih.gov/pubmed/27318122?dopt=AbstractPlus

Zagni C *et al*. (2017) The Search for Potent, Small‐Molecule HDACIs in Cancer Treatment: A Decade After Vorinostat. *Med Res Rev* **37**: 1373‐1428 https://www.ncbi.nlm.nih.gov/pubmed/28181261?dopt=AbstractPlus

### Further reading on Chromatin modifying enzymes

Angus SP *et al*. (2018) Epigenetic Mechanisms Regulating Adaptive Responses to Targeted Kinase Inhibitors in Cancer. *Annu Rev Pharmacol Toxicol* **58**: 209‐229 https://www.ncbi.nlm.nih.gov/pubmed/28934561?dopt=AbstractPlus

Bennett RL *et al*. (2018) Targeting Epigenetics in Cancer. *Annu Rev Pharmacol Toxicol* **58**: 187‐207 https://www.ncbi.nlm.nih.gov/pubmed/28992434?dopt=AbstractPlus

Lauschke VM *et al*. (2018) Pharmacoepigenetics and Toxicoepigenetics: Novel Mechanistic Insights and Therapeutic Opportunities. *Annu Rev Pharmacol Toxicol* **58**: 161‐185 https://www.ncbi.nlm.nih.gov/pubmed/29029592?dopt=AbstractPlus

## http://www.guidetopharmacology.org/GRAC/FamilyDisplayForward?familyId=241

### Overview

Cyclic nucleotides are second messengers generated by cyclase enzymes from precursor triphosphates and hydrolysed by phosphodiesterases. The cellular actions of these cyclic nucleotides are mediated through activation of protein kinases (http://www.guidetopharmacology.org/GRAC/FamilyDisplayForward?familyId=284‐ and http://www.guidetopharmacology.org/GRAC/FamilyDisplayForward?familyId=287‐dependent protein kinases), ion channels (http://www.guidetopharmacology.org/GRAC/FamilyDisplayForward?familyId=71, and http://www.guidetopharmacology.org/GRAC/FamilyDisplayForward?familyId=71) and guanine nucleotide exchange factors (GEFs, http://www.guidetopharmacology.org/GRAC/FamilyDisplayForward?familyId=259).

## http://www.guidetopharmacology.org/GRAC/FamilyDisplayForward?familyId=257

### Overview

Adenylyl cyclase, http://www.genome.jp/kegg‐bin/search_brite?option=‐a&search_string=4.6.1.1, converts http://www.guidetopharmacology.org/GRAC/LigandDisplayForward?ligandId=1713 to http://www.guidetopharmacology.org/GRAC/LigandDisplayForward?ligandId=2352 and pyrophosphate. Mammalian membranedelimited adenylyl cyclases (**nomenclature as approved by the NC‐IUPHAR Subcommittee on Adenylyl cyclases** [http://www.ncbi.nlm.nih.gov/pubmed/28255005?dopt=AbstractPlus]) are typically made up of two clusters of six TM domains separating two intracellular, overlapping catalytic domains that are the target for the nonselective activators Gα_s_ (the stimulatory G protein α subunit) and http://www.guidetopharmacology.org/GRAC/LigandDisplayForward?ligandId=5190 (except AC9, [http://www.ncbi.nlm.nih.gov/pubmed/8662814?dopt=AbstractPlus]). http://www.guidetopharmacology.org/GRAC/LigandDisplayForward?ligandId=2844 and its derivatives (*e.g*. http://www.guidetopharmacology.org/GRAC/LigandDisplayForward?ligandId=5108), acting through the P‐site,are inhibitors of adenylyl cyclase activity [http://www.ncbi.nlm.nih.gov/pubmed/11087399?dopt=AbstractPlus]. Four families of membranous adenylyl cyclase are distinguishable: http://www.guidetopharmacology.org/GRAC/LigandDisplayForward?ligandId=2351 (https://www.genenames.org/data/gene‐symbol‐report/#!/hgnc_id/HGNC:1442 https://www.genenames.org/data/gene‐symbol‐report/#!/hgnc_id/HGNC:1445 https://www.genenames.org/data/gene‐symbol‐report/#!/hgnc_id/HGNC:1449, http://www.uniprot.org/uniprot/P62158)‐stimulated (AC1, AC3 and AC8), Ca^2+^‐ and Gβγ‐inhibitable (AC5, AC6 and AC9), Gβγ‐stimulated and Ca^2+^‐insensitive (AC2, AC4 and AC7), and forskolin‐insensitive (AC9) forms. A soluble adenylyl cyclase (AC10) lacks membrane spanning regions and is insensitive to G proteins.It functions as a cytoplasmic bicarbonate (pH‐insensitive) sensor [http://www.ncbi.nlm.nih.gov/pubmed/10915626?dopt=AbstractPlus].

**Table:** 

Nomenclature	http://www.guidetopharmacology.org/GRAC/ObjectDisplayForward?objectId=1278	http://www.guidetopharmacology.org/GRAC/ObjectDisplayForward?objectId=1279	http://www.guidetopharmacology.org/GRAC/ObjectDisplayForward?objectId=1280	http://www.guidetopharmacology.org/GRAC/ObjectDisplayForward?objectId=1281	http://www.guidetopharmacology.org/GRAC/ObjectDisplayForward?objectId=1282
Common abbreviation	AC1	AC2	AC3	AC4	AC5
HGNC, UniProt	https://www.genenames.org/data/gene‐symbol‐report/#!/hgnc_id/HGNC:232, http://www.uniprot.org/uniprot/Q08828	https://www.genenames.org/data/gene‐symbol‐report/#!/hgnc_id/HGNC:233, http://www.uniprot.org/uniprot/Q08462	https://www.genenames.org/data/gene‐symbol‐report/#!/hgnc_id/HGNC:234, http://www.uniprot.org/uniprot/O60266	https://www.genenames.org/data/gene‐symbol‐report/#!/hgnc_id/HGNC:235, http://www.uniprot.org/uniprot/Q8NFM4	https://www.genenames.org/data/gene‐symbol‐report/#!/hgnc_id/HGNC:236, http://www.uniprot.org/uniprot/O95622
EC number	http://www.genome.jp/dbget‐bin/www_bget?ec:4.6.1.1	http://www.genome.jp/dbget‐bin/www_bget?ec:4.6.1.1	http://www.genome.jp/dbget‐bin/www_bget?ec:4.6.1.1	http://www.genome.jp/dbget‐bin/www_bget?ec:4.6.1.1	http://www.genome.jp/dbget‐bin/www_bget?ec:4.6.1.1
Endogenous activators	http://www.guidetopharmacology.org/GRAC/LigandDisplayForward?ligandId=2351 (https://www.genenames.org/data/gene‐symbol‐report/#!/hgnc_id/HGNC:1445, http://www.uniprot.org/uniprot/P62158), PKC‐evoked phosphorylation [http://www.ncbi.nlm.nih.gov/pubmed/8440678?dopt=AbstractPlus, http://www.ncbi.nlm.nih.gov/pubmed/2022671?dopt=AbstractPlus]	Gβγ, PKC‐evoked phosphorylation, Raf‐evoked phosphorylation [http://www.ncbi.nlm.nih.gov/pubmed/8389756?dopt=AbstractPlus, http://www.ncbi.nlm.nih.gov/pubmed/15385642?dopt=AbstractPlus, http://www.ncbi.nlm.nih.gov/pubmed/8390980?dopt=AbstractPlus, http://www.ncbi.nlm.nih.gov/pubmed/8416978?dopt=AbstractPlus]	http://www.guidetopharmacology.org/GRAC/LigandDisplayForward?ligandId=2351 (https://www.genenames.org/data/gene‐symbol‐report/#!/hgnc_id/HGNC:1445, http://www.uniprot.org/uniprot/P62158), PKC‐evoked phosphorylation [http://www.ncbi.nlm.nih.gov/pubmed/1633161?dopt=AbstractPlus, http://www.ncbi.nlm.nih.gov/pubmed/8440678?dopt=AbstractPlus]	Gβγ [http://www.ncbi.nlm.nih.gov/pubmed/1946437?dopt=AbstractPlus]	PKC‐evoked phophorylation, Gβγ, Raf‐evoked phophorylation [http://www.ncbi.nlm.nih.gov/pubmed/15385642?dopt=AbstractPlus, http://www.ncbi.nlm.nih.gov/pubmed/17110384?dopt=AbstractPlus, http://www.ncbi.nlm.nih.gov/pubmed/8206971?dopt=AbstractPlus]
Activators	http://www.guidetopharmacology.org/GRAC/LigandDisplayForward?ligandId=8809 (pIC_50_ 7.7) [http://www.ncbi.nlm.nih.gov/pubmed/8709105?dopt=AbstractPlus] – Bovine	http://www.guidetopharmacology.org/GRAC/LigandDisplayForward?ligandId=10214 [http://www.ncbi.nlm.nih.gov/pubmed/11602596?dopt=AbstractPlus]	–	–	http://www.guidetopharmacology.org/GRAC/LigandDisplayForward?ligandId=10215 [http://www.ncbi.nlm.nih.gov/pubmed/11602596?dopt=AbstractPlus]
Endogenous inhibitors	Gα_i_, Gα_o_, G_bg_ [http://www.ncbi.nlm.nih.gov/pubmed/8416978?dopt=AbstractPlus, http://www.ncbi.nlm.nih.gov/pubmed/8119955?dopt=AbstractPlus]	–	http://www.guidetopharmacology.org/GRAC/ObjectDisplayForward?objectId=2808, Gβγ, CaM kinase II‐evoked phosphorylation [http://www.ncbi.nlm.nih.gov/pubmed/16275644?dopt=AbstractPlus, http://www.ncbi.nlm.nih.gov/pubmed/11234015?dopt=AbstractPlus, http://www.ncbi.nlm.nih.gov/pubmed/7665559?dopt=AbstractPlus]	PKC‐evoked phophorylation [http://www.ncbi.nlm.nih.gov/pubmed/8900209?dopt=AbstractPlus]	Gα_i_, http://www.guidetopharmacology.org/GRAC/LigandDisplayForward?ligandId=707, PKA‐evoked phosphorylation, Gβγ, http://www.guidetopharmacology.org/GRAC/LigandDisplayForward?ligandId=2509 [http://www.ncbi.nlm.nih.gov/pubmed/17110384?dopt=AbstractPlus, http://www.ncbi.nlm.nih.gov/pubmed/10781930?dopt=AbstractPlus, http://www.ncbi.nlm.nih.gov/pubmed/1618857?dopt=AbstractPlus, http://www.ncbi.nlm.nih.gov/pubmed/7759492?dopt=AbstractPlus, http://www.ncbi.nlm.nih.gov/pubmed/8119955?dopt=AbstractPlus]
Inhibitors	–	http://www.guidetopharmacology.org/GRAC/LigandDisplayForward?ligandId=944 [http://www.ncbi.nlm.nih.gov/pubmed/24008337?dopt=AbstractPlus]	–	–	http://www.guidetopharmacology.org/GRAC/LigandDisplayForward?ligandId=5233 (pIC_50_ 5.2) [http://www.ncbi.nlm.nih.gov/pubmed/24006339?dopt=AbstractPlus, http://www.ncbi.nlm.nih.gov/pubmed/11602596?dopt=AbstractPlus]
Selective inhibitors	http://www.guidetopharmacology.org/GRAC/LigandDisplayForward?ligandId=10092 (pIC_50_ 5.6) [http://www.ncbi.nlm.nih.gov/pubmed/28223412?dopt=AbstractPlus]	–	–	–	–
Nomenclature	http://www.guidetopharmacology.org/GRAC/ObjectDisplayForward?objectId=1283	http://www.guidetopharmacology.org/GRAC/ObjectDisplayForward?objectId=1284	http://www.guidetopharmacology.org/GRAC/ObjectDisplayForward?objectId=1285	http://www.guidetopharmacology.org/GRAC/ObjectDisplayForward?objectId=1286	http://www.guidetopharmacology.org/GRAC/ObjectDisplayForward?objectId=3068
Common abbreviation	AC6	AC7	AC8	AC9	AC10
HGNC, UniProt	https://www.genenames.org/data/gene‐symbol‐report/#!/hgnc_id/HGNC:237, http://www.uniprot.org/uniprot/O43306	https://www.genenames.org/data/gene‐symbol‐report/#!/hgnc_id/HGNC:238, http://www.uniprot.org/uniprot/P51828	https://www.genenames.org/data/gene‐symbol‐report/#!/hgnc_id/HGNC:239, http://www.uniprot.org/uniprot/P40145	https://www.genenames.org/data/gene‐symbol‐report/#!/hgnc_id/HGNC:240, http://www.uniprot.org/uniprot/O60503	https://www.genenames.org/data/gene‐symbol‐report/#!/hgnc_id/HGNC:21285, http://www.uniprot.org/uniprot/Q96PN6
EC number	http://www.genome.jp/dbget‐bin/www_bget?ec:4.6.1.1	http://www.genome.jp/dbget‐bin/www_bget?ec:4.6.1.1	http://www.genome.jp/dbget‐bin/www_bget?ec:4.6.1.1	http://www.genome.jp/dbget‐bin/www_bget?ec:4.6.1.1	–
Endogenous activators	Gβγ, Raf‐evoked phosphorylation [http://www.ncbi.nlm.nih.gov/pubmed/15385642?dopt=AbstractPlus, http://www.ncbi.nlm.nih.gov/pubmed/17110384?dopt=AbstractPlus]	Gβγ, PKC‐evoked phosphorylation [http://www.ncbi.nlm.nih.gov/pubmed/15581358?dopt=AbstractPlus, http://www.ncbi.nlm.nih.gov/pubmed/7961850?dopt=AbstractPlus]	http://www.guidetopharmacology.org/GRAC/LigandDisplayForward?ligandId=2351 (https://www.genenames.org/data/gene‐symbol‐report/#!/hgnc_id/HGNC:1445, http://www.uniprot.org/uniprot/P62158) [http://www.ncbi.nlm.nih.gov/pubmed/8163524?dopt=AbstractPlus]	–	Bicarbonate, Ca^2+^ [http://www.ncbi.nlm.nih.gov/pubmed/10915626?dopt=AbstractPlus, http://www.ncbi.nlm.nih.gov/pubmed/12609998?dopt=AbstractPlus]
Endogenous inhibitors	Gα_i_, http://www.guidetopharmacology.org/GRAC/LigandDisplayForward?ligandId=707, PKA‐evoked phosphorylation, PKC‐evoked phosphorylation, http://www.guidetopharmacology.org/GRAC/LigandDisplayForward?ligandId=2509 [http://www.ncbi.nlm.nih.gov/pubmed/9391159?dopt=AbstractPlus, http://www.ncbi.nlm.nih.gov/pubmed/10781930?dopt=AbstractPlus, http://www.ncbi.nlm.nih.gov/pubmed/10462552?dopt=AbstractPlus, http://www.ncbi.nlm.nih.gov/pubmed/8119955?dopt=AbstractPlus, http://www.ncbi.nlm.nih.gov/pubmed/1379717?dopt=AbstractPlus]	–	PKA‐evoked phosphorylation [http://www.ncbi.nlm.nih.gov/pubmed/22976297?dopt=AbstractPlus]	Ca^2+^/calcineurin [http://www.ncbi.nlm.nih.gov/pubmed/10987815?dopt=AbstractPlus]	–
Inhibitors	http://www.guidetopharmacology.org/GRAC/LigandDisplayForward?ligandId=5233 (pIC_50_ 4.8) [http://www.ncbi.nlm.nih.gov/pubmed/24006339?dopt=AbstractPlus]	–	–	–	http://www.guidetopharmacology.org/GRAC/LigandDisplayForward?ligandId=10216 (pIC_50_ 5–5.5) [http://www.ncbi.nlm.nih.gov/pubmed/16054031?dopt=AbstractPlus], http://www.guidetopharmacology.org/GRAC/LigandDisplayForward?ligandId=10023 (pIC_50_ 5) [http://www.ncbi.nlm.nih.gov/pubmed/27547922?dopt=AbstractPlus]

### Comments

Many of the activators and inhibitors listed are only somewhat selective or have not been tested against all AC isoforms [http://www.ncbi.nlm.nih.gov/pubmed/24006339?dopt=AbstractPlus, http://www.ncbi.nlm.nih.gov/pubmed/24008337?dopt=AbstractPlus]. AC3 shows only modest *in vitro* activation by Ca^2+^/CaM.

### Further reading on Adenylyl cyclases (ACs)

Dessauer CW *et al*. (2017) International Union of Basic and Clinical Pharmacology. CI. Structures and Small Molecule Modulators of Mammalian Adenylyl Cyclases. *Pharmacol. Rev*. **69**: 93‐139 https://www.ncbi.nlm.nih.gov/pubmed/28255005?dopt=AbstractPlus

Halls ML et al. (2017) Adenylyl cyclase signalling complexes ‐ Pharmacological challenges and opportunities. *Pharmacol. Ther*. **172**: 171‐180 https://www.ncbi.nlm.nih.gov/pubmed/28132906?dopt=AbstractPlus

Wiggins SV *et al*. (2018) Pharmacological modulation of the CO_2_/HCO^‐^_3_/pH‐, calcium‐, and ATP‐sensing soluble adenylyl cyclase. *Pharmacol Ther* **190**: 173‐186 https://www.ncbi.nlm.nih.gov/pubmed/29807057

Wu L *et al*. (2016) Adenylate cyclase 3: a new target for anti‐obesity drug development. *Obes Rev* **17**: 907‐14 https://www.ncbi.nlm.nih.gov/pubmed/27256589?dopt=AbstractPlus

## http://www.guidetopharmacology.org/GRAC/FamilyDisplayForward?familyId=259

### Overview

Epacs are members of a family of guanine nucleotide exchange factors (http://www.ensembl.org/Homo_sapiens/Gene/Family/Genes?family=ENSFM00250000000899), which also includes https://www.genenames.org/data/gene‐symbol‐report/#!/hgnc_id/HGNC:16862 (GFR, KIAA0277, MR‐GEF, http://www.uniprot.org/uniprot/Q92565) and https://www.genenames.org/data/gene‐symbol‐report/#!/hgnc_id/HGNC:17428 (Link‐GEFII, http://www.uniprot.org/uniprot/Q9UHV5). They are activated endogenously by http://www.guidetopharmacology.org/GRAC/LigandDisplayForward?ligandId=2352 and with some pharmacological selectivity by http://www.guidetopharmacology.org/GRAC/LigandDisplayForward?ligandId=5172 [http://www.ncbi.nlm.nih.gov/pubmed/12402047?dopt=AbstractPlus]. Once activated, Epacs induce an enhanced activity of the monomeric G proteins, Rap1 and Rap2 by facilitating binding of http://www.guidetopharmacology.org/GRAC/LigandDisplayForward?ligandId=1742 in place of http://www.guidetopharmacology.org/GRAC/LigandDisplayForward?ligandId=2410, leading to activation of http://www.guidetopharmacology.org/GRAC/FamilyDisplayForward?familyId=244 [http://www.ncbi.nlm.nih.gov/pubmed/11715024?dopt=AbstractPlus].

**Table:** 

Nomenclature	http://www.guidetopharmacology.org/GRAC/ObjectDisplayForward?objectId=1292	http://www.guidetopharmacology.org/GRAC/ObjectDisplayForward?objectId=1293
Common abbreviation	Epac1	Epac2
HGNC, UniProt	https://www.genenames.org/data/gene‐symbol‐report/#!/hgnc_id/HGNC:16629, http://www.uniprot.org/uniprot/O95398	https://www.genenames.org/data/gene‐symbol‐report/#!/hgnc_id/HGNC:16626, http://www.uniprot.org/uniprot/Q8WZA2
Inhibitors	http://www.guidetopharmacology.org/GRAC/LigandDisplayForward?ligandId=7917 (pIC_50_ 5.5) [http://www.ncbi.nlm.nih.gov/pubmed/23066090?dopt=AbstractPlus]	http://www.guidetopharmacology.org/GRAC/LigandDisplayForward?ligandId=6556 (pIC_50_ 6.5) [http://www.ncbi.nlm.nih.gov/pubmed/23286832?dopt=AbstractPlus], http://www.guidetopharmacology.org/GRAC/LigandDisplayForward?ligandId=7917 (pIC_50_ 4.4–5.2) [http://www.ncbi.nlm.nih.gov/pubmed/23066090?dopt=AbstractPlus, http://www.ncbi.nlm.nih.gov/pubmed/24256330?dopt=AbstractPlus]

### Further reading on Exchange protein activated by cyclic AMP (EPACs)

Fujita T *et al*. (2017) The role of Epac in the heart. *Cell. Mol. Life Sci*. **74**: 591‐606 https://www.ncbi.nlm.nih.gov/pubmed/27549789?dopt=AbstractPlus

Robichaux WG and Cheng X. (2018) Intracellular cAMP Sensor EPAC: Physiology, Pathophysiology, and Therapeutics Development. *Physiol Rev* **98**: 919‐1053 https://www.ncbi.nlm.nih.gov/pubmed/29537337

Wang P *et al*. (2017) Exchange proteins directly activated by cAMP (EPACs): Emerging therapeutic targets. *Bioorg. Med. Chem. Lett*. **27**: 1633‐1639 https://www.ncbi.nlm.nih.gov/pubmed/28283242?dopt=AbstractPlus

## http://www.guidetopharmacology.org/GRAC/FamilyDisplayForward?familyId=260

### Overview

3’,5’‐Cyclic nucleotide phosphodiesterases (PDEs, 3’,5’‐cyclic‐nucleotide 5’‐nucleotidohydrolase), http://www.genome.jp/kegg‐bin/search_brite?option=‐a&search_string=3.1.4.17, catalyse the hydrolysis of a 3’,5’‐cyclic nucleotide (usually http://www.guidetopharmacology.org/GRAC/LigandDisplayForward?ligandId=2352 or http://www.guidetopharmacology.org/GRAC/LigandDisplayForward?ligandId=2347). http://www.guidetopharmacology.org/GRAC/LigandDisplayForward?ligandId=388 is a nonselective inhibitor with an IC50 value in the millimolar range for all isoforms except PDE 8A, 8B and 9A. A 2’,3’‐cyclic nucleotide 3’‐phosphodiesterase (http://www.genome.jp/kegg‐bin/search_brite?option=‐a&search_string=3.1.4.37 CNPase) activity is associated with myelin formation in the development of the CNS.

**Table:** 

Nomenclature	http://www.guidetopharmacology.org/GRAC/ObjectDisplayForward?objectId=1294	http://www.guidetopharmacology.org/GRAC/ObjectDisplayForward?objectId=1295	http://www.guidetopharmacology.org/GRAC/ObjectDisplayForward?objectId=1296
Common abbreviation	PDE1A	PDE1B	PDE1C
HGNC, UniProt	https://www.genenames.org/data/gene‐symbol‐report/#!/hgnc_id/HGNC:8774, http://www.uniprot.org/uniprot/P54750	https://www.genenames.org/data/gene‐symbol‐report/#!/hgnc_id/HGNC:8775, http://www.uniprot.org/uniprot/Q01064	https://www.genenames.org/data/gene‐symbol‐report/#!/hgnc_id/HGNC:8776, http://www.uniprot.org/uniprot/Q14123
EC number	http://www.genome.jp/dbget‐bin/www_bget?ec:3.1.4.17	http://www.genome.jp/dbget‐bin/www_bget?ec:3.1.4.17	http://www.genome.jp/dbget‐bin/www_bget?ec:3.1.4.17
Rank order of affinity	http://www.guidetopharmacology.org/GRAC/LigandDisplayForward?ligandId=2347 > http://www.guidetopharmacology.org/GRAC/LigandDisplayForward?ligandId=2352	http://www.guidetopharmacology.org/GRAC/LigandDisplayForward?ligandId=2347 > http://www.guidetopharmacology.org/GRAC/LigandDisplayForward?ligandId=2352	http://www.guidetopharmacology.org/GRAC/LigandDisplayForward?ligandId=2347 = http://www.guidetopharmacology.org/GRAC/LigandDisplayForward?ligandId=2352
Endogenous activators	http://www.guidetopharmacology.org/GRAC/LigandDisplayForward?ligandId=2351 (https://www.genenames.org/data/gene‐symbol‐report/#!/hgnc_id/HGNC:1442 https://www.genenames.org/data/gene‐symbol‐report/#!/hgnc_id/HGNC:1445 https://www.genenames.org/data/gene‐symbol‐report/#!/hgnc_id/HGNC:1449, http://www.uniprot.org/uniprot/P62158)	http://www.guidetopharmacology.org/GRAC/LigandDisplayForward?ligandId=2351 (https://www.genenames.org/data/gene‐symbol‐report/#!/hgnc_id/HGNC:1442 https://www.genenames.org/data/gene‐symbol‐report/#!/hgnc_id/HGNC:1445 https://www.genenames.org/data/gene‐symbol‐report/#!/hgnc_id/HGNC:1449, http://www.uniprot.org/uniprot/P62158)	http://www.guidetopharmacology.org/GRAC/LigandDisplayForward?ligandId=2351 (https://www.genenames.org/data/gene‐symbol‐report/#!/hgnc_id/HGNC:1442 https://www.genenames.org/data/gene‐symbol‐report/#!/hgnc_id/HGNC:1445 https://www.genenames.org/data/gene‐symbol‐report/#!/hgnc_id/HGNC:1449, http://www.uniprot.org/uniprot/P62158)
Endogenous inhibitors	–	–	–
Inhibitors	http://www.guidetopharmacology.org/GRAC/LigandDisplayForward?ligandId=9151 (pIC_50_ 5.2) [http://www.ncbi.nlm.nih.gov/pubmed/19303290?dopt=AbstractPlus]	–	–
Sub/family‐selective inhibitors	–	–	–
Selective inhibitors	http://www.guidetopharmacology.org/GRAC/LigandDisplayForward?ligandId=5270 (pIC_50_ 7.2) [http://www.ncbi.nlm.nih.gov/pubmed/8961086?dopt=AbstractPlus], http://www.guidetopharmacology.org/GRAC/LigandDisplayForward?ligandId=5285 (pIC_50_ 5.1) [http://www.ncbi.nlm.nih.gov/pubmed/8557689?dopt=AbstractPlus]	http://www.guidetopharmacology.org/GRAC/LigandDisplayForward?ligandId=5270 (pIC_50_ 7.2) [http://www.ncbi.nlm.nih.gov/pubmed/8961086?dopt=AbstractPlus]	http://www.guidetopharmacology.org/GRAC/LigandDisplayForward?ligandId=5270 (pIC_50_ 7.2) [http://www.ncbi.nlm.nih.gov/pubmed/8961086?dopt=AbstractPlus], vinpocetine (pIC_50_ 4.3) [http://www.ncbi.nlm.nih.gov/pubmed/8557689?dopt=AbstractPlus]
Comments	–	–	–

**Table:** 

Nomenclature	http://www.guidetopharmacology.org/GRAC/ObjectDisplayForward?objectId=1297	http://www.guidetopharmacology.org/GRAC/ObjectDisplayForward?objectId=1298	http://www.guidetopharmacology.org/GRAC/ObjectDisplayForward?objectId=1299
Common abbreviation	PDE2A	PDE3A	PDE3B
HGNC, UniProt	https://www.genenames.org/data/gene‐symbol‐report/#!/hgnc_id/HGNC:8777, http://www.uniprot.org/uniprot/O00408	https://www.genenames.org/data/gene‐symbol‐report/#!/hgnc_id/HGNC:8778, http://www.uniprot.org/uniprot/Q14432	https://www.genenames.org/data/gene‐symbol‐report/#!/hgnc_id/HGNC:8779, http://www.uniprot.org/uniprot/Q13370
EC number	http://www.genome.jp/dbget‐bin/www_bget?ec:3.1.4.17	http://www.genome.jp/dbget‐bin/www_bget?ec:3.1.4.17	http://www.genome.jp/dbget‐bin/www_bget?ec:3.1.4.17
Rank order of affinity	http://www.guidetopharmacology.org/GRAC/LigandDisplayForward?ligandId=2352 ≫ http://www.guidetopharmacology.org/GRAC/LigandDisplayForward?ligandId=2347	–	–
Endogenous activators	http://www.guidetopharmacology.org/GRAC/LigandDisplayForward?ligandId=2347	–	–
Endogenous inhibitors	–	http://www.guidetopharmacology.org/GRAC/LigandDisplayForward?ligandId=2347	http://www.guidetopharmacology.org/GRAC/LigandDisplayForward?ligandId=2347
Inhibitors	http://www.guidetopharmacology.org/GRAC/LigandDisplayForward?ligandId=5225 (pIC_50_ <6.5) [http://www.ncbi.nlm.nih.gov/pubmed/10644042?dopt=AbstractPlus]	http://www.guidetopharmacology.org/GRAC/LigandDisplayForward?ligandId=7148 (pIC_50_ 6.7) [http://www.ncbi.nlm.nih.gov/pubmed/10644042?dopt=AbstractPlus], http://www.guidetopharmacology.org/GRAC/LigandDisplayForward?ligandId=7202 (pIC_50_ 4.8) [http://www.ncbi.nlm.nih.gov/pubmed/2536438?dopt=AbstractPlus]	–
Sub/family‐selective inhibitors	–	–	–
Selective inhibitors	http://www.guidetopharmacology.org/GRAC/LigandDisplayForward?ligandId=5147 (pIC_50_ 8.3–8.8) [http://www.ncbi.nlm.nih.gov/pubmed/15555642?dopt=AbstractPlus], http://www.guidetopharmacology.org/GRAC/LigandDisplayForward?ligandId=5179 (pIC_50_ 5.3) [http://www.ncbi.nlm.nih.gov/pubmed/8730511?dopt=AbstractPlus]	http://www.guidetopharmacology.org/GRAC/LigandDisplayForward?ligandId=5167 (pIC_50_ 7.5) [http://www.ncbi.nlm.nih.gov/pubmed/10644042?dopt=AbstractPlus], http://www.guidetopharmacology.org/GRAC/LigandDisplayForward?ligandId=7114 (pIC_50_ 7.1–7.3) [http://www.ncbi.nlm.nih.gov/pubmed/3027338?dopt=AbstractPlus, http://www.ncbi.nlm.nih.gov/pubmed/1311763?dopt=AbstractPlus, http://www.ncbi.nlm.nih.gov/pubmed/1321910?dopt=AbstractPlus], http://www.guidetopharmacology.org/GRAC/LigandDisplayForward?ligandId=5225 (pIC_50_ 6.3–6.4) [http://www.ncbi.nlm.nih.gov/pubmed/14592490?dopt=AbstractPlus, http://www.ncbi.nlm.nih.gov/pubmed/10644042?dopt=AbstractPlus]	http://www.guidetopharmacology.org/GRAC/LigandDisplayForward?ligandId=5167 (pIC_50_ 7.3) [http://www.ncbi.nlm.nih.gov/pubmed/10644042?dopt=AbstractPlus], http://www.guidetopharmacology.org/GRAC/LigandDisplayForward?ligandId=7148 (pIC_50_ 6.4) [http://www.ncbi.nlm.nih.gov/pubmed/10644042?dopt=AbstractPlus], http://www.guidetopharmacology.org/GRAC/LigandDisplayForward?ligandId=5225 (pIC_50_ 6) [http://www.ncbi.nlm.nih.gov/pubmed/10644042?dopt=AbstractPlus], http://www.guidetopharmacology.org/GRAC/LigandDisplayForward?ligandId=7202 (pIC_50_ 4.5) [http://www.ncbi.nlm.nih.gov/pubmed/10644042?dopt=AbstractPlus]
Comments	http://www.guidetopharmacology.org/GRAC/LigandDisplayForward?ligandId=5179 is also an inhibitor of http://www.guidetopharmacology.org/GRAC/FamilyDisplayForward?familyId=248#show_object_1230 (http://www.genome.jp/kegg‐bin/search_brite?option=‐a&search_string=3.5.4.4).	–	–

**Table:** 

Nomenclature	http://www.guidetopharmacology.org/GRAC/ObjectDisplayForward?objectId=1300	http://www.guidetopharmacology.org/GRAC/ObjectDisplayForward?objectId=1301	http://www.guidetopharmacology.org/GRAC/ObjectDisplayForward?objectId=1302	http://www.guidetopharmacology.org/GRAC/ObjectDisplayForward?objectId=1303	http://www.guidetopharmacology.org/GRAC/ObjectDisplayForward?objectId=1304
Common abbreviation	PDE4A	PDE4B	PDE4C	PDE4D	PDE5A
HGNC, UniProt	https://www.genenames.org/data/gene‐symbol‐report/#!/hgnc_id/HGNC:8780, http://www.uniprot.org/uniprot/P27815	https://www.genenames.org/data/gene‐symbol‐report/#!/hgnc_id/HGNC:8781, http://www.uniprot.org/uniprot/Q07343	https://www.genenames.org/data/gene‐symbol‐report/#!/hgnc_id/HGNC:8782, http://www.uniprot.org/uniprot/Q08493	https://www.genenames.org/data/gene‐symbol‐report/#!/hgnc_id/HGNC:8783, http://www.uniprot.org/uniprot/Q08499	https://www.genenames.org/data/gene‐symbol‐report/#!/hgnc_id/HGNC:8784, http://www.uniprot.org/uniprot/O76074
EC number	http://www.genome.jp/dbget‐bin/www_bget?ec:3.1.4.17	http://www.genome.jp/dbget‐bin/www_bget?ec:3.1.4.17	http://www.genome.jp/dbget‐bin/www_bget?ec:3.1.4.17	http://www.genome.jp/dbget‐bin/www_bget?ec:3.1.4.17	http://www.genome.jp/dbget‐bin/www_bget?ec:3.1.4.17
Rank order of affinity	http://www.guidetopharmacology.org/GRAC/LigandDisplayForward?ligandId=2352 ≫ http://www.guidetopharmacology.org/GRAC/LigandDisplayForward?ligandId=2347	http://www.guidetopharmacology.org/GRAC/LigandDisplayForward?ligandId=2352 ≫ http://www.guidetopharmacology.org/GRAC/LigandDisplayForward?ligandId=2347	http://www.guidetopharmacology.org/GRAC/LigandDisplayForward?ligandId=2352 ≫ http://www.guidetopharmacology.org/GRAC/LigandDisplayForward?ligandId=2347	http://www.guidetopharmacology.org/GRAC/LigandDisplayForward?ligandId=2352 ≫ http://www.guidetopharmacology.org/GRAC/LigandDisplayForward?ligandId=2347	http://www.guidetopharmacology.org/GRAC/LigandDisplayForward?ligandId=2347 > http://www.guidetopharmacology.org/GRAC/LigandDisplayForward?ligandId=2352
Activators	–	–	–	PKA‐mediated phosphorylation [http://www.ncbi.nlm.nih.gov/pubmed/12444918?dopt=AbstractPlus]	Protein kinase A, protein kinase G [http://www.ncbi.nlm.nih.gov/pubmed/10785399?dopt=AbstractPlus]
Inhibitors	http://www.guidetopharmacology.org/GRAC/LigandDisplayForward?ligandId=7399 (pIC_50_ 7.3) [http://www.ncbi.nlm.nih.gov/pubmed/18686943?dopt=AbstractPlus], http://www.guidetopharmacology.org/GRAC/LigandDisplayForward?ligandId=6557 (pIC_50_ 7.2) [http://www.ncbi.nlm.nih.gov/pubmed/9720765?dopt=AbstractPlus]	http://www.guidetopharmacology.org/GRAC/LigandDisplayForward?ligandId=6962 (pIC_50_ 9.4) [http://www.ncbi.nlm.nih.gov/pubmed/19195882?dopt=AbstractPlus], http://www.guidetopharmacology.org/GRAC/LigandDisplayForward?ligandId=7399 (pIC_50_ 7.2) [http://www.ncbi.nlm.nih.gov/pubmed/18686943?dopt=AbstractPlus], http://www.guidetopharmacology.org/GRAC/LigandDisplayForward?ligandId=6557 (pIC_50_ 6.5) [http://www.ncbi.nlm.nih.gov/pubmed/9720765?dopt=AbstractPlus]	http://www.guidetopharmacology.org/GRAC/LigandDisplayForward?ligandId=6557 (pIC_50_ 8.1) [http://www.ncbi.nlm.nih.gov/pubmed/9720765?dopt=AbstractPlus], http://www.guidetopharmacology.org/GRAC/LigandDisplayForward?ligandId=7399 (pIC_50_ 6.6) [http://www.ncbi.nlm.nih.gov/pubmed/18686943?dopt=AbstractPlus]	http://www.guidetopharmacology.org/GRAC/LigandDisplayForward?ligandId=6557 (pIC_50_ 8.4) [http://www.ncbi.nlm.nih.gov/pubmed/9720765?dopt=AbstractPlus], http://www.guidetopharmacology.org/GRAC/LigandDisplayForward?ligandId=9776 (pIC_50_ >7.3) [446], http://www.guidetopharmacology.org/GRAC/LigandDisplayForward?ligandId=9610 (pIC_50_ 6.1) [http://www.ncbi.nlm.nih.gov/pubmed/28613871?dopt=AbstractPlus]	http://www.guidetopharmacology.org/GRAC/LigandDisplayForward?ligandId=6558 (pIC_50_ 8.9) [http://www.ncbi.nlm.nih.gov/pubmed/22100260?dopt=AbstractPlus], http://www.guidetopharmacology.org/GRAC/LigandDisplayForward?ligandId=5225 (pIC_50_ 7.3)
Sub/family‐selective inhibitors	http://www.guidetopharmacology.org/GRAC/LigandDisplayForward?ligandId=5260 (pIC_50_ 9) [http://www.ncbi.nlm.nih.gov/pubmed/9177268?dopt=AbstractPlus], http://www.guidetopharmacology.org/GRAC/LigandDisplayForward?ligandId=9330 (p*K*_i_ 8) [http://www.ncbi.nlm.nih.gov/pubmed/9631241?dopt=AbstractPlus], http://www.guidetopharmacology.org/GRAC/LigandDisplayForward?ligandId=5258 (pIC_50_ 6.5) [http://www.ncbi.nlm.nih.gov/pubmed/9177268?dopt=AbstractPlus]	http://www.guidetopharmacology.org/GRAC/LigandDisplayForward?ligandId=5260 (pIC_50_ 9) [http://www.ncbi.nlm.nih.gov/pubmed/9177268?dopt=AbstractPlus], http://www.guidetopharmacology.org/GRAC/LigandDisplayForward?ligandId=5258 (pIC_50_ 6.4) [http://www.ncbi.nlm.nih.gov/pubmed/9177268?dopt=AbstractPlus]	http://www.guidetopharmacology.org/GRAC/LigandDisplayForward?ligandId=9330 (p*K*_i_ 7.7) [http://www.ncbi.nlm.nih.gov/pubmed/9631241?dopt=AbstractPlus], http://www.guidetopharmacology.org/GRAC/LigandDisplayForward?ligandId=5260 (pIC_50_ 6.5) [http://www.ncbi.nlm.nih.gov/pubmed/9177268?dopt=AbstractPlus], http://www.guidetopharmacology.org/GRAC/LigandDisplayForward?ligandId=5258 (pIC_50_ 5.4) [http://www.ncbi.nlm.nih.gov/pubmed/9177268?dopt=AbstractPlus]	http://www.guidetopharmacology.org/GRAC/LigandDisplayForward?ligandId=9330 (p*K*_i_ 8.1) [http://www.ncbi.nlm.nih.gov/pubmed/9631241?dopt=AbstractPlus], *rolipram* (pIC_50_ 7.2) [http://www.ncbi.nlm.nih.gov/pubmed/9177268?dopt=AbstractPlus], http://www.guidetopharmacology.org/GRAC/LigandDisplayForward?ligandId=5258 (pIC_50_ 6.2) [http://www.ncbi.nlm.nih.gov/pubmed/9177268?dopt=AbstractPlus]	–
Selective inhibitors	http://www.guidetopharmacology.org/GRAC/LigandDisplayForward?ligandId=5292 (pIC_50_ 8.3) [http://www.ncbi.nlm.nih.gov/pubmed/10991987?dopt=AbstractPlus], http://www.guidetopharmacology.org/GRAC/LigandDisplayForward?ligandId=7372 (pIC_50_ 7.8) [http://www.ncbi.nlm.nih.gov/pubmed/24882690?dopt=AbstractPlus]	–	http://www.guidetopharmacology.org/GRAC/LigandDisplayForward?ligandId=7372 (pIC_50_ 6.9) [http://www.ncbi.nlm.nih.gov/pubmed/24882690?dopt=AbstractPlus]	http://www.guidetopharmacology.org/GRAC/LigandDisplayForward?ligandId=7372 (pIC_50_ 7.5) [http://www.ncbi.nlm.nih.gov/pubmed/24882690?dopt=AbstractPlus]	http://www.guidetopharmacology.org/GRAC/LigandDisplayForward?ligandId=7320 (pIC_50_ 9.7) [http://www.ncbi.nlm.nih.gov/pubmed/15837326?dopt=AbstractPlus], http://www.guidetopharmacology.org/GRAC/LigandDisplayForward?ligandId=5275 (pIC_50_ 9.5) [http://www.ncbi.nlm.nih.gov/pubmed/12450574?dopt=AbstractPlus], http://www.guidetopharmacology.org/GRAC/LigandDisplayForward?ligandId=4743 (pIC_50_ 8.4–9) [http://www.ncbi.nlm.nih.gov/pubmed/10385692?dopt=AbstractPlus, http://www.ncbi.nlm.nih.gov/pubmed/23137303?dopt=AbstractPlus], http://www.guidetopharmacology.org/GRAC/LigandDisplayForward?ligandId=7299 (pIC_50_ 8.5) [http://www.ncbi.nlm.nih.gov/pubmed/21189023?dopt=AbstractPlus], http://www.guidetopharmacology.org/GRAC/LigandDisplayForward?ligandId=5270 (pIC_50_ 7.2) [http://www.ncbi.nlm.nih.gov/pubmed/8961086?dopt=AbstractPlus], http://www.guidetopharmacology.org/GRAC/LigandDisplayForward?ligandId=2919 (pIC_50_ 6.8) [http://www.ncbi.nlm.nih.gov/pubmed/10385692?dopt=AbstractPlus]

**Table:** 

Nomenclature	http://www.guidetopharmacology.org/GRAC/ObjectDisplayForward?objectId=1312	http://www.guidetopharmacology.org/GRAC/ObjectDisplayForward?objectId=1313	http://www.guidetopharmacology.org/GRAC/ObjectDisplayForward?objectId=1314	http://www.guidetopharmacology.org/GRAC/ObjectDisplayForward?objectId=1315	http://www.guidetopharmacology.org/GRAC/ObjectDisplayForward?objectId=1316	http://www.guidetopharmacology.org/GRAC/ObjectDisplayForward?objectId=1317
Common abbreviation	PDE6A	PDE6B	PDE6C	PDE6D	PDE6G	PDE6H
HGNC, UniProt	https://www.genenames.org/data/gene‐symbol‐report/#!/hgnc_id/HGNC:8785, http://www.uniprot.org/uniprot/P16499	https://www.genenames.org/data/gene‐symbol‐report/#!/hgnc_id/HGNC:8786, http://www.uniprot.org/uniprot/P35913	https://www.genenames.org/data/gene‐symbol‐report/#!/hgnc_id/HGNC:8787, http://www.uniprot.org/uniprot/P51160	https://www.genenames.org/data/gene‐symbol‐report/#!/hgnc_id/HGNC:8788, http://www.uniprot.org/uniprot/O43924	https://www.genenames.org/data/gene‐symbol‐report/#!/hgnc_id/HGNC:8789, http://www.uniprot.org/uniprot/P18545	https://www.genenames.org/data/gene‐symbol‐report/#!/hgnc_id/HGNC:8790, http://www.uniprot.org/uniprot/Q13956
EC number	http://www.genome.jp/dbget‐bin/www_bget?ec:3.1.4.17	http://www.genome.jp/dbget‐bin/www_bget?ec:3.1.4.17	http://www.genome.jp/dbget‐bin/www_bget?ec:3.1.4.17	http://www.genome.jp/dbget‐bin/www_bget?ec:3.1.4.17	http://www.genome.jp/dbget‐bin/www_bget?ec:3.1.4.17	http://www.genome.jp/dbget‐bin/www_bget?ec:3.1.4.17
Inhibitors	http://www.guidetopharmacology.org/GRAC/LigandDisplayForward?ligandId=8811 (pIC_50_ 8) [http://www.ncbi.nlm.nih.gov/pubmed/19631533?dopt=AbstractPlus]	–	http://www.guidetopharmacology.org/GRAC/LigandDisplayForward?ligandId=4743 (pIC_50_ 7.4) [http://www.ncbi.nlm.nih.gov/pubmed/23137303?dopt=AbstractPlus]	–	–	–

**Table:** 

Nomenclature	http://www.guidetopharmacology.org/GRAC/ObjectDisplayForward?objectId=1305	http://www.guidetopharmacology.org/GRAC/ObjectDisplayForward?objectId=1306	http://www.guidetopharmacology.org/GRAC/ObjectDisplayForward?objectId=1307	http://www.guidetopharmacology.org/GRAC/ObjectDisplayForward?objectId=1308
Common abbreviation	PDE7A	PDE7B	PDE8A	PDE8B
HGNC, UniProt	https://www.genenames.org/data/gene‐symbol‐report/#!/hgnc_id/HGNC:8791, http://www.uniprot.org/uniprot/Q13946	https://www.genenames.org/data/gene‐symbol‐report/#!/hgnc_id/HGNC:8792, http://www.uniprot.org/uniprot/Q9NP56	https://www.genenames.org/data/gene‐symbol‐report/#!/hgnc_id/HGNC:8793, http://www.uniprot.org/uniprot/O60658	https://www.genenames.org/data/gene‐symbol‐report/#!/hgnc_id/HGNC:8794, http://www.uniprot.org/uniprot/O95263
EC number	http://www.genome.jp/dbget‐bin/www_bget?ec:3.1.4.17	http://www.genome.jp/dbget‐bin/www_bget?ec:3.1.4.17	http://www.genome.jp/dbget‐bin/www_bget?ec:3.1.4.17	http://www.genome.jp/dbget‐bin/www_bget?ec:3.1.4.17
Rank order of affinity	http://www.guidetopharmacology.org/GRAC/LigandDisplayForward?ligandId=2352 ≫ http://www.guidetopharmacology.org/GRAC/LigandDisplayForward?ligandId=2347 [http://www.ncbi.nlm.nih.gov/pubmed/8389765?dopt=AbstractPlus]	http://www.guidetopharmacology.org/GRAC/LigandDisplayForward?ligandId=2352 ≫ http://www.guidetopharmacology.org/GRAC/LigandDisplayForward?ligandId=2347 [http://www.ncbi.nlm.nih.gov/pubmed/10872825?dopt=AbstractPlus]	http://www.guidetopharmacology.org/GRAC/LigandDisplayForward?ligandId=2352 ≫ http://www.guidetopharmacology.org/GRAC/LigandDisplayForward?ligandId=2347 [http://www.ncbi.nlm.nih.gov/pubmed/9618252?dopt=AbstractPlus]	http://www.guidetopharmacology.org/GRAC/LigandDisplayForward?ligandId=2352 ≫ http://www.guidetopharmacology.org/GRAC/LigandDisplayForward?ligandId=2347 [http://www.ncbi.nlm.nih.gov/pubmed/9784418?dopt=AbstractPlus]
Inhibitors	http://www.guidetopharmacology.org/GRAC/LigandDisplayForward?ligandId=9151 (pIC_50_ 6.1) [http://www.ncbi.nlm.nih.gov/pubmed/19303290?dopt=AbstractPlus]	http://www.guidetopharmacology.org/GRAC/LigandDisplayForward?ligandId=5154 (pIC_50_ 4.9) [http://www.ncbi.nlm.nih.gov/pubmed/20228279?dopt=AbstractPlus]	–	–
Selective inhibitors	http://www.guidetopharmacology.org/GRAC/LigandDisplayForward?ligandId=5154 (pIC_50_ 6.7–6.8) [http://www.ncbi.nlm.nih.gov/pubmed/20228279?dopt=AbstractPlus, http://www.ncbi.nlm.nih.gov/pubmed/15371556?dopt=AbstractPlus]	http://www.guidetopharmacology.org/GRAC/LigandDisplayForward?ligandId=4807 (pIC_50_ 5.7–6) [http://www.ncbi.nlm.nih.gov/pubmed/10872825?dopt=AbstractPlus, http://www.ncbi.nlm.nih.gov/pubmed/10814504?dopt=AbstractPlus], http://www.guidetopharmacology.org/GRAC/LigandDisplayForward?ligandId=5270 (pIC_50_ 5.8) [http://www.ncbi.nlm.nih.gov/pubmed/10814504?dopt=AbstractPlus]	http://www.guidetopharmacology.org/GRAC/LigandDisplayForward?ligandId=9659 (pIC_50_ 7.4) [http://www.ncbi.nlm.nih.gov/pubmed/24687066?dopt=AbstractPlus], http://www.guidetopharmacology.org/GRAC/LigandDisplayForward?ligandId=4807 (pIC_50_ 5.1) [http://www.ncbi.nlm.nih.gov/pubmed/9618252?dopt=AbstractPlus]	http://www.guidetopharmacology.org/GRAC/LigandDisplayForward?ligandId=4807 (pIC_50_ 4.3) [http://www.ncbi.nlm.nih.gov/pubmed/9784418?dopt=AbstractPlus]
Comments	PDE7A appears to be membrane‐bound or soluble for PDE7A1 and 7A2 splice variants, respectively	–	–	–

**Table:** 

Nomenclature	http://www.guidetopharmacology.org/GRAC/ObjectDisplayForward?objectId=1309	http://www.guidetopharmacology.org/GRAC/ObjectDisplayForward?objectId=1310	http://www.guidetopharmacology.org/GRAC/ObjectDisplayForward?objectId=1311
Common abbreviation	PDE9A	PDE10A	PDE11A
HGNC, UniProt	https://www.genenames.org/data/gene‐symbol‐report/#!/hgnc_id/HGNC:8795, http://www.uniprot.org/uniprot/O76083	https://www.genenames.org/data/gene‐symbol‐report/#!/hgnc_id/HGNC:8772, http://www.uniprot.org/uniprot/Q9Y233	https://www.genenames.org/data/gene‐symbol‐report/#!/hgnc_id/HGNC:8773, http://www.uniprot.org/uniprot/Q9HCR9
EC number	http://www.genome.jp/dbget‐bin/www_bget?ec:3.1.4.17	http://www.genome.jp/dbget‐bin/www_bget?ec:3.1.4.17	http://www.genome.jp/dbget‐bin/www_bget?ec:3.1.4.17
Rank order of affinity	http://www.guidetopharmacology.org/GRAC/LigandDisplayForward?ligandId=2347 ≫ http://www.guidetopharmacology.org/GRAC/LigandDisplayForward?ligandId=2352 [http://www.ncbi.nlm.nih.gov/pubmed/9624146?dopt=AbstractPlus]	http://www.guidetopharmacology.org/GRAC/LigandDisplayForward?ligandId=2352, http://www.guidetopharmacology.org/GRAC/LigandDisplayForward?ligandId=2347 [http://www.ncbi.nlm.nih.gov/pubmed/10373451?dopt=AbstractPlus]	http://www.guidetopharmacology.org/GRAC/LigandDisplayForward?ligandId=2352, http://www.guidetopharmacology.org/GRAC/LigandDisplayForward?ligandId=2347 [http://www.ncbi.nlm.nih.gov/pubmed/10725373?dopt=AbstractPlus]
Inhibitors	http://www.guidetopharmacology.org/GRAC/LigandDisplayForward?ligandId=5270 (pIC_50_ 5.8) [http://www.ncbi.nlm.nih.gov/pubmed/9624146?dopt=AbstractPlus], http://www.guidetopharmacology.org/GRAC/LigandDisplayForward?ligandId=2919 (pIC_50_ 4.5) [http://www.ncbi.nlm.nih.gov/pubmed/9624146?dopt=AbstractPlus]	–	http://www.guidetopharmacology.org/GRAC/LigandDisplayForward?ligandId=7299 (pIC_50_ 6.5) [http://www.ncbi.nlm.nih.gov/pubmed/21189023?dopt=AbstractPlus], http://www.guidetopharmacology.org/GRAC/LigandDisplayForward?ligandId=6559 (pIC_50_ 6.5) [http://www.ncbi.nlm.nih.gov/pubmed/22284362?dopt=AbstractPlus]
Selective inhibitors	–	http://www.guidetopharmacology.org/GRAC/LigandDisplayForward?ligandId=9617 (pIC_50_ 9.4) [http://www.ncbi.nlm.nih.gov/pubmed/19630403?dopt=AbstractPlus]	–

### Comments

PDE1A, 1B and 1C appear to act as soluble homodimers, while PDE2A is a membrane‐bound homodimer. PDE3A and PDE3B are membrane‐bound.

PDE4 isoforms are essentially http://www.guidetopharmacology.org/GRAC/LigandDisplayForward?ligandId=2352 specific. The potency of http://www.guidetopharmacology.org/GRAC/LigandDisplayForward?ligandId=5292 at other members of the PDE4 family has not been reported. PDE4B‐D long forms are inhibited by extracellular signal‐regulated kinase (ERK)‐mediated phosphorylation [http://www.ncbi.nlm.nih.gov/pubmed/10022832?dopt=AbstractPlus, http://www.ncbi.nlm.nih.gov/pubmed/9639573?dopt=AbstractPlus]. PDE4A‐D splice variants can be membrane‐bound or cytosolic [http://www.ncbi.nlm.nih.gov/pubmed/12444918?dopt=AbstractPlus]. PDE4 isoforms may be labelled with http://www.guidetopharmacology.org/GRAC/LigandDisplayForward?ligandId=5313.

PDE6 is a membrane‐bound tetramer composed of two catalytic chains (PDE6A or PDE6C and PDE6B), an inhibitory chain (PDE6G or PDE6H) and the PDE6D chain. The enzyme is essentially http://www.guidetopharmacology.org/GRAC/LigandDisplayForward?ligandId=2347 specific and is activated by the a‐subunit of transducin (Gat) and inhibited by http://www.guidetopharmacology.org/GRAC/LigandDisplayForward?ligandId=4743, http://www.guidetopharmacology.org/GRAC/LigandDisplayForward?ligandId=2919 and http://www.guidetopharmacology.org/GRAC/LigandDisplayForward?ligandId=4807 with potencies lower than those observed for PDE5A. Defects in PDE6B are a cause of retinitis pigmentosa and congenital stationary night blindness.

### Further reading on Phosphodiesterases, 3’,5’‐cyclic nucleotide (PDEs)

Klussmann E. (2016) Protein‐protein interactions of PDE4 family members ‐ Functions, interactions and therapeutic value. *Cell. Signal*. **28**: 713‐8 https://www.ncbi.nlm.nih.gov/pubmed/26498857?dopt=AbstractPlus

Korkmaz‐Icöz S *et al*. (2018) Targeting phosphodiesterase 5 as a therapeutic option against myocardial ischaemia/reperfusion injury and for treating heart failure. *Br. J. Pharmacol*. **175**: 223‐231 https://www.ncbi.nlm.nih.gov/pubmed/28213937?dopt=AbstractPlus

Li H *et al*. (2018) Phosphodiesterase‐4 Inhibitors for the Treatment of Inflammatory Diseases. *Front Pharmacol* **9**: 1048 https://www.ncbi.nlm.nih.gov/pubmed/30386231

Mehta A and Patel BM. (2019) Therapeutic opportunities in colon cancer: Focus on phosphodiesterase inhibitors. *Life Sci* **230**: 150‐161 https://www.ncbi.nlm.nih.gov/pubmed/31125564

Ntontsi P *et al*. (2019) Experimental and investigational phosphodiesterase inhibitors in development for asthma. *Expert Opin Investig Drugs* **28**: 261‐266 https://www.ncbi.nlm.nih.gov/pubmed/30678501

Pauls MM. (2018) The effect of phosphodiesterase‐5 inhibitors on cerebral blood flow in humans: A systematic review. *J Cereb Blood Flow Metab* **38**: 189‐203 https://www.ncbi.nlm.nih.gov/pubmed/29256324

Peng T *et al*. (2018) Inhibitors of phosphodiesterase as cancer therapeutics. *Eur J Med Chem* **150**: 742‐756 https://www.ncbi.nlm.nih.gov/pubmed/29574203

Svensson F *et al*. (2018) Fragment‐Based Drug Discovery of Phosphodiesterase Inhibitors. *J Med Chem* **61**: 1415–1424 https://www.ncbi.nlm.nih.gov/pubmed/28800229

Wahlang B *et al*. (2018) Role of cAMP and phosphodiesterase signaling in liver health and disease. *Cell Signal* **49**: 105‐115 https://www.ncbi.nlm.nih.gov/pubmed/29902522

Zagorska A *et al*. (2018) Phosphodiesterase 10 Inhibitors ‐ Novel Perspectives for Psychiatric and Neurodegenerative Drug Discovery. *Curr Med Chem* **25**: 3455‐3481 https://www.ncbi.nlm.nih.gov/pubmed/29521210

## http://www.guidetopharmacology.org/GRAC/FamilyDisplayForward?familyId=242

### Overview

The cytochrome P450 enzyme family (CYP450), E.C. 1.14.‐.‐, were originally defined by their strong absorbance at 450 nm due to the reduced carbon monoxide‐complexed haem component of the cytochromes. They are an extensive family of haemcontaining monooxygenases with a huge range of both endogenous and exogenous substrates. These include sterols, fat‐soluble vitamins, pesticides and carcinogens as well as drugs. The substrates of some orphan CYP are not known. Listed below are the human enzymes; their relationship with rodent CYP450 enzyme activities is obscure in that the species orthologuemay not catalyse the metabolism of the same substrates. Although the majority of CYP450 enzyme activities are concentrated in the liver, the extrahepatic enzyme activities also contribute to patho/physiological processes. Genetic variation of CYP450 isoforms is widespread and likely underlies a significant proportion of the individual variation to drug administration.

## http://www.guidetopharmacology.org/GRAC/FamilyDisplayForward?familyId=261

**Table:** 

Nomenclature	http://www.guidetopharmacology.org/GRAC/ObjectDisplayForward?objectId=1318	http://www.guidetopharmacology.org/GRAC/ObjectDisplayForward?objectId=1319	http://www.guidetopharmacology.org/GRAC/ObjectDisplayForward?objectId=1320
HGNC, UniProt	https://www.genenames.org/data/gene‐symbol‐report/#!/hgnc_id/HGNC:2595, http://www.uniprot.org/uniprot/P04798	https://www.genenames.org/data/gene‐symbol‐report/#!/hgnc_id/HGNC:2596, http://www.uniprot.org/uniprot/P05177	https://www.genenames.org/data/gene‐symbol‐report/#!/hgnc_id/HGNC:2597, http://www.uniprot.org/uniprot/Q16678
EC number	http://www.genome.jp/dbget‐bin/www_bget?ec:1.14.1.1	http://www.genome.jp/dbget‐bin/www_bget?ec:1.14.1.1	http://www.genome.jp/dbget‐bin/www_bget?ec:1.14.1.1
Inhibitors	http://www.guidetopharmacology.org/GRAC/LigandDisplayForward?ligandId=8748 (pIC_50_ 7.4) [http://www.ncbi.nlm.nih.gov/pubmed/23600958?dopt=AbstractPlus]	http://www.guidetopharmacology.org/GRAC/LigandDisplayForward?ligandId=8748 (pIC_50_ 6.4) [http://www.ncbi.nlm.nih.gov/pubmed/23600958?dopt=AbstractPlus]	stilbenes [http://www.ncbi.nlm.nih.gov/pubmed/28458135?dopt=AbstractPlus]
Comments	CYP1A1 is an extra‐hepatic enzyme. It shows a preference for linear planar aromatic molecules [http://www.ncbi.nlm.nih.gov/pubmed/28698457?dopt=AbstractPlus].	CYP1A2 is constitutively expressed in liver. It shows a preference for triangular planar aromatic molecules [http://www.ncbi.nlm.nih.gov/pubmed/28698457?dopt=AbstractPlus].	Mainly expressed in extra‐hepatic tissues. Can metabolise 17β‐estradiol [http://www.ncbi.nlm.nih.gov/pubmed/28458135?dopt=AbstractPlus], leukotrienes and eicosanoids [http://www.ncbi.nlm.nih.gov/pubmed/23956430?dopt=AbstractPlus]. Mutations have been associated with primary congenitial glaucoma [http://www.ncbi.nlm.nih.gov/pubmed/9097971?dopt=AbstractPlus]

## http://www.guidetopharmacology.org/GRAC/FamilyDisplayForward?familyId=262

**Table:** 

Nomenclature	http://www.guidetopharmacology.org/GRAC/ObjectDisplayForward?objectId=1321	http://www.guidetopharmacology.org/GRAC/ObjectDisplayForward?objectId=1322	http://www.guidetopharmacology.org/GRAC/ObjectDisplayForward?objectId=1323	http://www.guidetopharmacology.org/GRAC/ObjectDisplayForward?objectId=1324	http://www.guidetopharmacology.org/GRAC/ObjectDisplayForward?objectId=1325
HGNC, UniProt	https://www.genenames.org/data/gene‐symbol‐report/#!/hgnc_id/HGNC:2610, http://www.uniprot.org/uniprot/P11509	https://www.genenames.org/data/gene‐symbol‐report/#!/hgnc_id/HGNC:2611, http://www.uniprot.org/uniprot/P20853	https://www.genenames.org/data/gene‐symbol‐report/#!/hgnc_id/HGNC:2608, http://www.uniprot.org/uniprot/Q16696	https://www.genenames.org/data/gene‐symbol‐report/#!/hgnc_id/HGNC:2615, http://www.uniprot.org/uniprot/P20813	https://www.genenames.org/data/gene‐symbol‐report/#!/hgnc_id/HGNC:2622, http://www.uniprot.org/uniprot/P10632
EC number	http://www.genome.jp/dbget‐bin/www_bget?ec:1.14.14.1	http://www.genome.jp/dbget‐bin/www_bget?ec:1.14.14.1	http://www.genome.jp/dbget‐bin/www_bget?ec:1.14.14.1	http://www.genome.jp/dbget‐bin/www_bget?ec:1.14.14.1	http://www.genome.jp/dbget‐bin/www_bget?ec:1.14.14.1
Substrates	http://www.guidetopharmacology.org/GRAC/LigandDisplayForward?ligandId=2585	–	–	–	–
Inhibitors	–	–	–	http://www.guidetopharmacology.org/GRAC/LigandDisplayForward?ligandId=7307 (pIC_50_ 6.7) [http://www.ncbi.nlm.nih.gov/pubmed/16248836?dopt=AbstractPlus], sibutramine (pIC_50_ 5.8) [http://www.ncbi.nlm.nih.gov/pubmed/23777987?dopt=AbstractPlus], http://www.guidetopharmacology.org/GRAC/LigandDisplayForward?ligandId=7622 (p*K*_i_ 5.3) [http://www.ncbi.nlm.nih.gov/pubmed/17682072?dopt=AbstractPlus]	http://www.guidetopharmacology.org/GRAC/LigandDisplayForward?ligandId=7266 (p*K*_i_ 5.1) [http://www.ncbi.nlm.nih.gov/pubmed/16248836?dopt=AbstractPlus]
Comments	Metabolises coumarin [http://www.ncbi.nlm.nih.gov/pubmed/2322567?dopt=AbstractPlus].	CYP2A7 does not incorporate haem and is functionally inactive [http://www.ncbi.nlm.nih.gov/pubmed/16636685?dopt=AbstractPlus]	Metabolises tobacco carcinogen, 4‐methylnitrosoamino)‐1‐(3‐pyridyl)‐1‐butanone [http://www.ncbi.nlm.nih.gov/pubmed/11016631?dopt=AbstractPlus].	Substrates include: efavirenz, bupropion, cyclophosphamide, ketamine, propofol [http://www.ncbi.nlm.nih.gov/pubmed/23152403?dopt=AbstractPlus].	Converts http://www.guidetopharmacology.org/GRAC/LigandDisplayForward?ligandId=2391 to 11(R)‐12(S)‐epoxyeicosatrienoic acid or 14(R)‐15(S)‐epoxyeicosatrienoic acid [http://www.ncbi.nlm.nih.gov/pubmed/7574697?dopt=AbstractPlus]. Drug substrates include http://www.guidetopharmacology.org/GRAC/LigandDisplayForward?ligandId=10018 [http://www.ncbi.nlm.nih.gov/pubmed/26721703?dopt=AbstractPlus].

**Table:** 

Nomenclature	http://www.guidetopharmacology.org/GRAC/ObjectDisplayForward?objectId=1328	http://www.guidetopharmacology.org/GRAC/ObjectDisplayForward?objectId=1329	http://www.guidetopharmacology.org/GRAC/ObjectDisplayForward?objectId=1330	http://www.guidetopharmacology.org/GRAC/ObjectDisplayForward?objectId=1331	http://www.guidetopharmacology.org/GRAC/ObjectDisplayForward?objectId=1332	http://www.guidetopharmacology.org/GRAC/ObjectDisplayForward?objectId=1333
HGNC, UniProt	https://www.genenames.org/data/gene‐symbol‐report/#!/hgnc_id/HGNC:2621, http://www.uniprot.org/uniprot/P33261	https://www.genenames.org/data/gene‐symbol‐report/#!/hgnc_id/HGNC:2625, http://www.uniprot.org/uniprot/P10635	https://www.genenames.org/data/gene‐symbol‐report/#!/hgnc_id/HGNC:2631, http://www.uniprot.org/uniprot/P05181	https://www.genenames.org/data/gene‐symbol‐report/#!/hgnc_id/HGNC:2632, http://www.uniprot.org/uniprot/P24903	https://www.genenames.org/data/gene‐symbol‐report/#!/hgnc_id/HGNC:2634, http://www.uniprot.org/uniprot/P51589	https://www.genenames.org/data/gene‐symbol‐report/#!/hgnc_id/HGNC:20580, http://www.uniprot.org/uniprot/Q6VVX0
EC number	http://www.genome.jp/dbget‐bin/www_bget?ec:1.14.14.51 (S)‐limonene + [reduced NADPH–hemoprotein reductase] + O(2) <=> (‐)‐trans‐carveol + [oxidized NADPH–hemoprotein reductase] + H(2)O	http://www.genome.jp/dbget‐bin/www_bget?ec:1.14.14.1	http://www.genome.jp/dbget‐bin/www_bget?ec:1.14.14.1	http://www.genome.jp/dbget‐bin/www_bget?ec:1.14.14.1	http://www.genome.jp/dbget‐bin/www_bget?ec:1.14.14.1	http://www.genome.jp/dbget‐bin/www_bget?ec:1.14.13.15
Inhibitors	http://www.guidetopharmacology.org/GRAC/LigandDisplayForward?ligandId=8777 (pIC_50_ 7.3) [120]	–	http://www.guidetopharmacology.org/GRAC/LigandDisplayForward?ligandId=8756 (p*K*_i_ 7.4) [http://www.ncbi.nlm.nih.gov/pubmed/17125252?dopt=AbstractPlus]	–	http://www.guidetopharmacology.org/GRAC/LigandDisplayForward?ligandId=8832 (pIC_50_ 6.8) [http://www.ncbi.nlm.nih.gov/pubmed/16495056?dopt=AbstractPlus], http://www.guidetopharmacology.org/GRAC/LigandDisplayForward?ligandId=2608 (pIC_50_ 5.1) [http://www.ncbi.nlm.nih.gov/pubmed/16495056?dopt=AbstractPlus]	–
Selective inhibitors	http://www.guidetopharmacology.org/GRAC/LigandDisplayForward?ligandId=8749 (p*K*_i_ 7.7) [http://www.ncbi.nlm.nih.gov/pubmed/22239545?dopt=AbstractPlus]	–	–	–	–	–
Comments	Substrates include: omeprazole, proguanil, mephenytoin, diazepam [http://www.ncbi.nlm.nih.gov/pubmed/2495208?dopt=AbstractPlus, http://www.ncbi.nlm.nih.gov/pubmed/12222994?dopt=AbstractPlus, http://www.ncbi.nlm.nih.gov/pubmed/2291871?dopt=AbstractPlus].	Substrates include: debrisoquine, metoprolol, codeine [http://www.ncbi.nlm.nih.gov/pubmed/19102711?dopt=AbstractPlus].	Substrates: Ethanol, p‐nitrophenol [http://www.ncbi.nlm.nih.gov/pubmed/30380359?dopt=AbstractPlus].	Substrate: naphthalene [http://www.ncbi.nlm.nih.gov/pubmed/20408502?dopt=AbstractPlus].	Converts http://www.guidetopharmacology.org/GRAC/LigandDisplayForward?ligandId=2391 to 14(R)‐15(S)‐epoxyeicosatrienoic acid [http://www.ncbi.nlm.nih.gov/pubmed/8631948?dopt=AbstractPlus]. Hydroxylates albendazole[http://www.ncbi.nlm.nih.gov/pubmed/23959307?dopt=AbstractPlus]. Expressed in cardiomyocytes [http://www.ncbi.nlm.nih.gov/pubmed/29695613?dopt=AbstractPlus].	Converts http://www.guidetopharmacology.org/GRAC/LigandDisplayForward?ligandId=2747 to http://www.guidetopharmacology.org/GRAC/LigandDisplayForward?ligandId=6921 [http://www.ncbi.nlm.nih.gov/pubmed/12867411?dopt=AbstractPlus].

## http://www.guidetopharmacology.org/GRAC/FamilyDisplayForward?familyId=263

**Table:** 

Nomenclature	http://www.guidetopharmacology.org/GRAC/ObjectDisplayForward?objectId=1337	http://www.guidetopharmacology.org/GRAC/ObjectDisplayForward?objectId=1338	http://www.guidetopharmacology.org/GRAC/ObjectDisplayForward?objectId=1339	http://www.guidetopharmacology.org/GRAC/ObjectDisplayForward?objectId=1340
HGNC, UniProt	https://www.genenames.org/data/gene‐symbol‐report/#!/hgnc_id/HGNC:2637, http://www.uniprot.org/uniprot/P08684	https://www.genenames.org/data/gene‐symbol‐report/#!/hgnc_id/HGNC:2638, http://www.uniprot.org/uniprot/P20815	https://www.genenames.org/data/gene‐symbol‐report/#!/hgnc_id/HGNC:2640, http://www.uniprot.org/uniprot/P24462	https://www.genenames.org/data/gene‐symbol‐report/#!/hgnc_id/HGNC:17450, http://www.uniprot.org/uniprot/Q9HB55
EC number	http://www.genome.jp/dbget‐bin/www_bget?ec:1.14.14.56: 1,8‐cineole + http://www.guidetopharmacology.org/GRAC/LigandDisplayForward?ligandId=3041 + O_2_ = 2‐exo‐hydroxy‐1,8‐cineole + http://www.guidetopharmacology.org/GRAC/LigandDisplayForward?ligandId=3045 + H_2_O http://www.genome.jp/dbget‐bin/www_bget?ec:1.14.13.97: Taurochenodeoxycholate + http://www.guidetopharmacology.org/GRAC/LigandDisplayForward?ligandId=3041 + O_2_ = taurohyocholate + http://www.guidetopharmacology.org/GRAC/LigandDisplayForward?ligandId=3045 + H_2_O Lithocholate + http://www.guidetopharmacology.org/GRAC/LigandDisplayForward?ligandId=3041 + O_2_ = hyodeoxycholate + http://www.guidetopharmacology.org/GRAC/LigandDisplayForward?ligandId=3045 + H_2_O http://www.genome.jp/dbget‐bin/www_bget?ec:1.14.14.55: http://www.guidetopharmacology.org/GRAC/LigandDisplayForward?ligandId=2510 + http://www.guidetopharmacology.org/GRAC/LigandDisplayForward?ligandId=3041 + O_2_ = 3‐hydroxyquinine + http://www.guidetopharmacology.org/GRAC/LigandDisplayForward?ligandId=3045 + http://www.guidetopharmacology.org/GRAC/LigandDisplayForward?ligandId=2448	http://www.genome.jp/dbget‐bin/www_bget?ec:1.14.14.1	http://www.genome.jp/dbget‐bin/www_bget?ec:1.14.14.1	http://www.genome.jp/dbget‐bin/www_bget?ec:1.14.14.1
Substrates	http://www.guidetopharmacology.org/GRAC/LigandDisplayForward?ligandId=2514 [http://www.ncbi.nlm.nih.gov/pubmed/3514607?dopt=AbstractPlus], http://www.guidetopharmacology.org/GRAC/LigandDisplayForward?ligandId=3342 [http://www.ncbi.nlm.nih.gov/pubmed/12124305?dopt=AbstractPlus]	–	–	–
Inhibitors	http://www.guidetopharmacology.org/GRAC/LigandDisplayForward?ligandId=10209 (p*K*_i_ 7.8) [http://www.ncbi.nlm.nih.gov/pubmed/26002732?dopt=AbstractPlus], http://www.guidetopharmacology.org/GRAC/LigandDisplayForward?ligandId=2568 (p*K*_i_ 7) [http://www.ncbi.nlm.nih.gov/pubmed/25923589?dopt=AbstractPlus], http://www.guidetopharmacology.org/GRAC/LigandDisplayForward?ligandId=8804 (p*K*_i_ >7) [http://www.ncbi.nlm.nih.gov/pubmed/18285471?dopt=AbstractPlus]	http://www.guidetopharmacology.org/GRAC/LigandDisplayForward?ligandId=8804 (p*K*_i_ 6.9) [http://www.ncbi.nlm.nih.gov/pubmed/16248836?dopt=AbstractPlus]	–	–
Comments	Metabolises a vast range of xenobiotics, including antidepressants, benzodiazepines, calcium channel blockers, and chemotherapeutic agents [http://www.ncbi.nlm.nih.gov/pubmed/18473749?dopt=AbstractPlus]. The active site is plastic, with both homotropic and heterotropic cooperativity observed with some substrates [http://www.ncbi.nlm.nih.gov/pubmed/26002732?dopt=AbstractPlus]. CYP3A4 catalyses the 25‐hydroxylation of http://www.guidetopharmacology.org/GRAC/LigandDisplayForward?ligandId=2767 in liver microsomes [http://www.ncbi.nlm.nih.gov/pubmed/9931427?dopt=AbstractPlus].	CYP3A5 is expressed extrahepatically, including in the small intestine. It has overlapping substrate specificity with CYP3A4 [http://www.ncbi.nlm.nih.gov/pubmed/16430309?dopt=AbstractPlus, http://www.ncbi.nlm.nih.gov/pubmed/12124305?dopt=AbstractPlus].	Fetal form, rarely expressed in adults. Has overlapping substrate specificity with CYP3A4 [http://www.ncbi.nlm.nih.gov/pubmed/16430309?dopt=AbstractPlus, http://www.ncbi.nlm.nih.gov/pubmed/12124305?dopt=AbstractPlus].	Fetal expression only and considered an orphan fCYP [http://www.ncbi.nlm.nih.gov/pubmed/21737533?dopt=AbstractPlus]. Testosterone may be a substrate [http://www.ncbi.nlm.nih.gov/pubmed/11160875?dopt=AbstractPlus].

## http://www.guidetopharmacology.org/GRAC/FamilyDisplayForward?familyId=264

**Table:** 

Nomenclature	http://www.guidetopharmacology.org/GRAC/ObjectDisplayForward?objectId=1341	http://www.guidetopharmacology.org/GRAC/ObjectDisplayForward?objectId=1342	http://www.guidetopharmacology.org/GRAC/ObjectDisplayForward?objectId=1343	http://www.guidetopharmacology.org/GRAC/ObjectDisplayForward?objectId=1344	http://www.guidetopharmacology.org/GRAC/ObjectDisplayForward?objectId=1345
HGNC, UniProt	https://www.genenames.org/data/gene‐symbol‐report/#!/hgnc_id/HGNC:2642, http://www.uniprot.org/uniprot/Q02928	https://www.genenames.org/data/gene‐symbol‐report/#!/hgnc_id/HGNC:20575, http://www.uniprot.org/uniprot/Q5TCH4	https://www.genenames.org/data/gene‐symbol‐report/#!/hgnc_id/HGNC:2644, http://www.uniprot.org/uniprot/P13584	https://www.genenames.org/data/gene‐symbol‐report/#!/hgnc_id/HGNC:2645, http://www.uniprot.org/uniprot/P78329	https://www.genenames.org/data/gene‐symbol‐report/#!/hgnc_id/HGNC:2646, http://www.uniprot.org/uniprot/Q08477
EC number	http://www.genome.jp/dbget‐bin/www_bget?ec:1.14.14.80	http://www.genome.jp/dbget‐bin/www_bget?ec:1.14.14.80	http://www.genome.jp/dbget‐bin/www_bget?ec:1.14.14.1	http://www.genome.jp/dbget‐bin/www_bget?ec:1.14.14.78 http://www.genome.jp/dbget‐bin/www_bget?ec:1.14.14.79 http://www.genome.jp/dbget‐bin/www_bget?ec:1.14.14.94	http://www.genome.jp/dbget‐bin/www_bget?ec:1.14.14.78 http://www.genome.jp/dbget‐bin/www_bget?ec:1.14.14.79 http://www.genome.jp/dbget‐bin/www_bget?ec:1.14.14.94
Inhibitors	–	–	–	http://www.guidetopharmacology.org/GRAC/LigandDisplayForward?ligandId=8851 (p*K*_i_ 5.9) [http://www.ncbi.nlm.nih.gov/pubmed/2997155?dopt=AbstractPlus]	–
Comments	Converts http://www.guidetopharmacology.org/GRAC/LigandDisplayForward?ligandId=5534 to http://www.guidetopharmacology.org/GRAC/LigandDisplayForward?ligandId=6922.	Reported to be enzymatically inactive [http://www.ncbi.nlm.nih.gov/pubmed/15611369?dopt=AbstractPlus].	–	Responsible for *ω*‐hydroxylation of http://www.guidetopharmacology.org/GRAC/LigandDisplayForward?ligandId=2487, http://www.guidetopharmacology.org/GRAC/LigandDisplayForward?ligandId=5216 [http://www.ncbi.nlm.nih.gov/pubmed/8389204?dopt=AbstractPlus], and tocopherols, including vitamin E [http://www.ncbi.nlm.nih.gov/pubmed/11997390?dopt=AbstractPlus]	Responsible for *ω*‐hydroxylation of http://www.guidetopharmacology.org/GRAC/LigandDisplayForward?ligandId=2487, http://www.guidetopharmacology.org/GRAC/LigandDisplayForward?ligandId=5216 [http://www.ncbi.nlm.nih.gov/pubmed/8389204?dopt=AbstractPlus], and polyunsaturated fatty acids [http://www.ncbi.nlm.nih.gov/pubmed/18577768?dopt=AbstractPlus, http://www.ncbi.nlm.nih.gov/pubmed/16820285?dopt=AbstractPlus]

**Table:** 

Nomenclature	http://www.guidetopharmacology.org/GRAC/ObjectDisplayForward?objectId=1346	http://www.guidetopharmacology.org/GRAC/ObjectDisplayForward?objectId=1347	http://www.guidetopharmacology.org/GRAC/ObjectDisplayForward?objectId=1348	http://www.guidetopharmacology.org/GRAC/ObjectDisplayForward?objectId=1349	http://www.guidetopharmacology.org/GRAC/ObjectDisplayForward?objectId=1350	http://www.guidetopharmacology.org/GRAC/ObjectDisplayForward?objectId=1351	http://www.guidetopharmacology.org/GRAC/ObjectDisplayForward?objectId=1352
HGNC, UniProt	https://www.genenames.org/data/gene‐symbol‐report/#!/hgnc_id/HGNC:2648, http://www.uniprot.org/uniprot/P98187	https://www.genenames.org/data/gene‐symbol‐report/#!/hgnc_id/HGNC:13265, http://www.uniprot.org/uniprot/Q9HBI6	https://www.genenames.org/data/gene‐symbol‐report/#!/hgnc_id/HGNC:18857, http://www.uniprot.org/uniprot/Q9HCS2	https://www.genenames.org/data/gene‐symbol‐report/#!/hgnc_id/HGNC:26820, http://www.uniprot.org/uniprot/Q6NT55	https://www.genenames.org/data/gene‐symbol‐report/#!/hgnc_id/HGNC:23198, http://www.uniprot.org/uniprot/Q6ZWL3	https://www.genenames.org/data/gene‐symbol‐report/#!/hgnc_id/HGNC:20244, http://www.uniprot.org/uniprot/Q8N118	https://www.genenames.org/data/gene‐symbol‐report/#!/hgnc_id/HGNC:20583, http://www.uniprot.org/uniprot/Q86W10
EC number	http://www.genome.jp/dbget‐bin/www_bget?ec:1.14.14.1	http://www.genome.jp/dbget‐bin/www_bget?ec:1.14.14.1 http://www.genome.jp/dbget‐bin/www_bget?ec:1.14.14.78	http://www.genome.jp/dbget‐bin/www_bget?ec:1.14.14.1	http://www.genome.jp/dbget‐bin/www_bget?ec:1.14.14.‐	http://www.genome.jp/dbget‐bin/www_bget?ec:1.14.14.79	http://www.genome.jp/dbget‐bin/www_bget?ec:1.14.14.1	http://www.genome.jp/dbget‐bin/www_bget?ec:1.14.14.1
Comments	Converts http://www.guidetopharmacology.org/GRAC/LigandDisplayForward?ligandId=4483 to 19‐hydroxyPGH_2_ [http://www.ncbi.nlm.nih.gov/pubmed/10791960?dopt=AbstractPlus] and 8,9‐EET or 11,12‐EET to 18‐hydroxy‐8,9‐EET or 18‐hydroxy‐11,12‐EET [http://www.ncbi.nlm.nih.gov/pubmed/19919823?dopt=AbstractPlus].	–	AC004597.1 (http://www.ensembl.org/Homo_sapiens/Gene/Summary?g=ENSG00000225607;r=19:15880826‐15890774;t=ENST00000412610) is described as being highly similar to CYP4F12	Converts http://www.guidetopharmacology.org/GRAC/LigandDisplayForward?ligandId=2391 to http://www.guidetopharmacology.org/GRAC/LigandDisplayForward?ligandId=6923 and http://www.guidetopharmacology.org/GRAC/LigandDisplayForward?ligandId=6924 [http://www.ncbi.nlm.nih.gov/pubmed/19919823?dopt=AbstractPlus].	Converts http://www.guidetopharmacology.org/GRAC/LigandDisplayForward?ligandId=2806 to 14‐hydroxymyristic acid [http://www.ncbi.nlm.nih.gov/pubmed/19661213?dopt=AbstractPlus].	Converts http://www.guidetopharmacology.org/GRAC/LigandDisplayForward?ligandId=2364 to 14,15‐poxyeicosatrienoic ethanolamide [http://www.ncbi.nlm.nih.gov/pubmed/18549450?dopt=AbstractPlus].	Converts lauric acid to 12‐hydroxylauric acid.

## http://www.guidetopharmacology.org/GRAC/FamilyDisplayForward?familyId=265

**Table:** 

Nomenclature	http://www.guidetopharmacology.org/GRAC/ObjectDisplayForward?objectId=1353	http://www.guidetopharmacology.org/GRAC/ObjectDisplayForward?objectId=1354	http://www.guidetopharmacology.org/GRAC/ObjectDisplayForward?objectId=1355	http://www.guidetopharmacology.org/GRAC/ObjectDisplayForward?objectId=1356	http://www.guidetopharmacology.org/GRAC/ObjectDisplayForward?objectId=1357
Common abbreviation	Thromboxane synthase	–	–	Prostacyclin synthase	–
HGNC, UniProt	https://www.genenames.org/data/gene‐symbol‐report/#!/hgnc_id/HGNC:11609, http://www.uniprot.org/uniprot/P24557	https://www.genenames.org/data/gene‐symbol‐report/#!/hgnc_id/HGNC:2651, http://www.uniprot.org/uniprot/P22680	https://www.genenames.org/data/gene‐symbol‐report/#!/hgnc_id/HGNC:2652, http://www.uniprot.org/uniprot/O75881	https://www.genenames.org/data/gene‐symbol‐report/#!/hgnc_id/HGNC:9603, http://www.uniprot.org/uniprot/Q16647	https://www.genenames.org/data/gene‐symbol‐report/#!/hgnc_id/HGNC:2653, http://www.uniprot.org/uniprot/Q9UNU6
EC number	http://www.genome.jp/dbget‐bin/www_bget?ec:5.3.99.5: http://www.guidetopharmacology.org/GRAC/LigandDisplayForward?ligandId=4483 = http://www.guidetopharmacology.org/GRAC/LigandDisplayForward?ligandId=4482	http://www.genome.jp/dbget‐bin/www_bget?ec:1.14.14.23	http://www.genome.jp/dbget‐bin/www_bget?ec:1.14.14.29	http://www.genome.jp/dbget‐bin/www_bget?ec:5.3.99.4	http://www.genome.jp/dbget‐bin/www_bget?ec:1.14.14.139 http://www.genome.jp/dbget‐bin/www_bget?ec:1.14.18.8
Inhibitors	http://www.guidetopharmacology.org/GRAC/LigandDisplayForward?ligandId=9866 (pIC_50_ 8.4) [http://www.ncbi.nlm.nih.gov/pubmed/3093741?dopt=AbstractPlus]	–	–	http://www.guidetopharmacology.org/GRAC/LigandDisplayForward?ligandId=8795 (pIC_50_ >6) [http://www.ncbi.nlm.nih.gov/pubmed/7861416?dopt=AbstractPlus]	–
Comments	Inhibited by http://www.guidetopharmacology.org/GRAC/LigandDisplayForward?ligandId=5175 [http://www.ncbi.nlm.nih.gov/pubmed/6795753?dopt=AbstractPlus], http://www.guidetopharmacology.org/GRAC/LigandDisplayForward?ligandId=5157 [http://www.ncbi.nlm.nih.gov/pubmed/7778318?dopt=AbstractPlus] and furegrelate sodium (U‐63557A: PubChem https://pubchem.ncbi.nlm.nih.gov/compound/23663954) [http://www.ncbi.nlm.nih.gov/pubmed/6316421?dopt=AbstractPlus].	Converts http://www.guidetopharmacology.org/GRAC/LigandDisplayForward?ligandId=2718 to http://www.guidetopharmacology.org/GRAC/LigandDisplayForward?ligandId=4351 [http://www.ncbi.nlm.nih.gov/pubmed/2384150?dopt=AbstractPlus].	Converts http://www.guidetopharmacology.org/GRAC/LigandDisplayForward?ligandId=2370 to 7α‐DHEA [http://www.ncbi.nlm.nih.gov/pubmed/9144166?dopt=AbstractPlus].	Converts http://www.guidetopharmacology.org/GRAC/LigandDisplayForward?ligandId=4483 to http://www.guidetopharmacology.org/GRAC/LigandDisplayForward?ligandId=1915 [http://www.ncbi.nlm.nih.gov/pubmed/8766713?dopt=AbstractPlus]. Inhibited by http://www.guidetopharmacology.org/GRAC/LigandDisplayForward?ligandId=5281 [http://www.ncbi.nlm.nih.gov/pubmed/824685?dopt=AbstractPlus]	Converts 7α‐hydroxycholest‐4‐en‐3‐one to 7‐alpha, 12α‐dihydroxycholest‐4‐en‐3‐one (in rabbit) [http://www.ncbi.nlm.nih.gov/pubmed/1400444?dopt=AbstractPlus] in the biosynthesis of bile acids.

## http://www.guidetopharmacology.org/GRAC/FamilyDisplayForward?familyId=266

**Table:** 

Nomenclature	http://www.guidetopharmacology.org/GRAC/ObjectDisplayForward?objectId=1358	http://www.guidetopharmacology.org/GRAC/ObjectDisplayForward?objectId=1359	http://www.guidetopharmacology.org/GRAC/ObjectDisplayForward?objectId=1360	http://www.guidetopharmacology.org/GRAC/ObjectDisplayForward?objectId=1361	http://www.guidetopharmacology.org/GRAC/ObjectDisplayForward?objectId=1362	http://www.guidetopharmacology.org/GRAC/ObjectDisplayForward?objectId=1363	http://www.guidetopharmacology.org/GRAC/ObjectDisplayForward?objectId=1364
Common abbreviation	–	–	Aldosterone synthase	–	Aromatase	–	–
HGNC, UniProt	https://www.genenames.org/data/gene‐symbol‐report/#!/hgnc_id/HGNC:2590, http://www.uniprot.org/uniprot/P05108	https://www.genenames.org/data/gene‐symbol‐report/#!/hgnc_id/HGNC:2591, http://www.uniprot.org/uniprot/P15538	https://www.genenames.org/data/gene‐symbol‐report/#!/hgnc_id/HGNC:2592, http://www.uniprot.org/uniprot/P19099	https://www.genenames.org/data/gene‐symbol‐report/#!/hgnc_id/HGNC:2593, http://www.uniprot.org/uniprot/P05093	https://www.genenames.org/data/gene‐symbol‐report/#!/hgnc_id/HGNC:2594, http://www.uniprot.org/uniprot/P11511	https://www.genenames.org/data/gene‐symbol‐report/#!/hgnc_id/HGNC:20576, http://www.uniprot.org/uniprot/Q6UW02	https://www.genenames.org/data/gene‐symbol‐report/#!/hgnc_id/HGNC:2600, http://www.uniprot.org/uniprot/P08686
EC number	http://www.genome.jp/dbget‐bin/www_bget?ec:1.14.15.6	http://www.genome.jp/dbget‐bin/www_bget?ec:1.14.15.4	http://www.genome.jp/dbget‐bin/www_bget?ec:1.14.15.4 http://www.genome.jp/dbget‐bin/www_bget?ec:1.14.15.5	http://www.genome.jp/dbget‐bin/www_bget?ec:1.14.14.19 http://www.genome.jp/dbget‐bin/www_bget?ec:1.14.14.32	http://www.genome.jp/dbget‐bin/www_bget?ec:1.14.14.14	http://www.genome.jp/dbget‐bin/www_bget?ec:1.14.‐.‐	http://www.genome.jp/dbget‐bin/www_bget?ec:1.14.14.16
Inhibitors	http://www.guidetopharmacology.org/GRAC/LigandDisplayForward?ligandId=6957 [http://www.ncbi.nlm.nih.gov/pubmed/23254310?dopt=AbstractPlus, http://www.ncbi.nlm.nih.gov/pubmed/17395972?dopt=AbstractPlus]	http://www.guidetopharmacology.org/GRAC/LigandDisplayForward?ligandId=5224 (pIC_50_ 7.8) [http://www.ncbi.nlm.nih.gov/pubmed/21129965?dopt=AbstractPlus], http://www.guidetopharmacology.org/GRAC/LigandDisplayForward?ligandId=6957	http://www.guidetopharmacology.org/GRAC/LigandDisplayForward?ligandId=8310 (pIC_50_ 9.7) [http://www.ncbi.nlm.nih.gov/pubmed/24899257?dopt=AbstractPlus]	http://www.guidetopharmacology.org/GRAC/LigandDisplayForward?ligandId=6745 (pIC_50_ 7.1–8.4) [http://www.ncbi.nlm.nih.gov/pubmed/18672868?dopt=AbstractPlus, http://www.ncbi.nlm.nih.gov/pubmed/7608911?dopt=AbstractPlus]	http://www.guidetopharmacology.org/GRAC/LigandDisplayForward?ligandId=5137 (pIC_50_ 7.8) [http://www.ncbi.nlm.nih.gov/pubmed/20413308?dopt=AbstractPlus], http://www.guidetopharmacology.org/GRAC/LigandDisplayForward?ligandId=7054 [http://www.ncbi.nlm.nih.gov/pubmed/19470632?dopt=AbstractPlus]	–	http://www.guidetopharmacology.org/GRAC/LigandDisplayForward?ligandId=8871 (pIC_50_ 5.3) [http://www.ncbi.nlm.nih.gov/pubmed/1495014?dopt=AbstractPlus] – Rat
Selective inhibitors	–	–	–	http://www.guidetopharmacology.org/GRAC/LigandDisplayForward?ligandId=8638 (pIC_50_ 6.5) [http://www.ncbi.nlm.nih.gov/pubmed/15828836?dopt=AbstractPlus]	http://www.guidetopharmacology.org/GRAC/LigandDisplayForward?ligandId=5209 (p*K*_i_ 10.7) [http://www.ncbi.nlm.nih.gov/pubmed/22386564?dopt=AbstractPlus], http://www.guidetopharmacology.org/GRAC/LigandDisplayForward?ligandId=7073 (pIC_50_ 7.3) [http://www.ncbi.nlm.nih.gov/pubmed/2951074?dopt=AbstractPlus], http://www.guidetopharmacology.org/GRAC/LigandDisplayForward?ligandId=7303 (p*K*_i_ 4.5) [http://www.ncbi.nlm.nih.gov/pubmed/7083195?dopt=AbstractPlus]	–	–
Comments	Converts http://www.guidetopharmacology.org/GRAC/LigandDisplayForward?ligandId=2718 to http://www.guidetopharmacology.org/GRAC/LigandDisplayForward?ligandId=2376 plus 4‐methylpentanal.	Converts http://www.guidetopharmacology.org/GRAC/LigandDisplayForward?ligandId=3450 and http://www.guidetopharmacology.org/GRAC/LigandDisplayForward?ligandId=5100 to http://www.guidetopharmacology.org/GRAC/LigandDisplayForward?ligandId=5171 and http://www.guidetopharmacology.org/GRAC/LigandDisplayForward?ligandId=2868, respectively. Loss‐of‐function mutations are associated with familial adrenal hyperplasia and hypertension. Inhibited by http://www.guidetopharmacology.org/GRAC/LigandDisplayForward?ligandId=5224 [http://www.ncbi.nlm.nih.gov/pubmed/412519?dopt=AbstractPlus]	Converts http://www.guidetopharmacology.org/GRAC/LigandDisplayForward?ligandId=2869 to http://www.guidetopharmacology.org/GRAC/LigandDisplayForward?ligandId=2872	Converts http://www.guidetopharmacology.org/GRAC/LigandDisplayForward?ligandId=2376 and http://www.guidetopharmacology.org/GRAC/LigandDisplayForward?ligandId=2377 to http://www.guidetopharmacology.org/GRAC/LigandDisplayForward?ligandId=5104 and http://www.guidetopharmacology.org/GRAC/LigandDisplayForward?ligandId=5103, respectively. Converts http://www.guidetopharmacology.org/GRAC/LigandDisplayForward?ligandId=5103 and http://www.guidetopharmacology.org/GRAC/LigandDisplayForward?ligandId=5103 to http://www.guidetopharmacology.org/GRAC/LigandDisplayForward?ligandId=2370 and http://www.guidetopharmacology.org/GRAC/LigandDisplayForward?ligandId=2860, respectively. Converts http://www.guidetopharmacology.org/GRAC/LigandDisplayForward?ligandId=2869 to http://www.guidetopharmacology.org/GRAC/LigandDisplayForward?ligandId=2868.	Converts http://www.guidetopharmacology.org/GRAC/LigandDisplayForward?ligandId=2860 and http://www.guidetopharmacology.org/GRAC/LigandDisplayForward?ligandId=2858 to http://www.guidetopharmacology.org/GRAC/LigandDisplayForward?ligandId=2818 and http://www.guidetopharmacology.org/GRAC/LigandDisplayForward?ligandId=1013, respectively. Inhibited by http://www.guidetopharmacology.org/GRAC/LigandDisplayForward?ligandId=5137 [http://www.ncbi.nlm.nih.gov/pubmed/7949201?dopt=AbstractPlus], and http://www.guidetopharmacology.org/GRAC/LigandDisplayForward?ligandId=5209 [http://www.ncbi.nlm.nih.gov/pubmed/2149502?dopt=AbstractPlus]	–	Converts http://www.guidetopharmacology.org/GRAC/LigandDisplayForward?ligandId=2377 and http://www.guidetopharmacology.org/GRAC/LigandDisplayForward?ligandId=5103 to http://www.guidetopharmacology.org/GRAC/LigandDisplayForward?ligandId=3450 and http://www.guidetopharmacology.org/GRAC/LigandDisplayForward?ligandId=5100, respectively

## http://www.guidetopharmacology.org/GRAC/FamilyDisplayForward?familyId=267

**Table:** 

Nomenclature	http://www.guidetopharmacology.org/GRAC/ObjectDisplayForward?objectId=1365	http://www.guidetopharmacology.org/GRAC/ObjectDisplayForward?objectId=1366	http://www.guidetopharmacology.org/GRAC/ObjectDisplayForward?objectId=1367	http://www.guidetopharmacology.org/GRAC/ObjectDisplayForward?objectId=1368	http://www.guidetopharmacology.org/GRAC/ObjectDisplayForward?objectId=1369	http://www.guidetopharmacology.org/GRAC/ObjectDisplayForward?objectId=1370	http://www.guidetopharmacology.org/GRAC/ObjectDisplayForward?objectId=1371
Common abbreviation	–	–	–	–	Sterol 27‐hydroxylase	–	–
HGNC, UniProt	https://www.genenames.org/data/gene‐symbol‐report/#!/hgnc_id/HGNC:2602, http://www.uniprot.org/uniprot/Q07973	https://www.genenames.org/data/gene‐symbol‐report/#!/hgnc_id/HGNC:2603, http://www.uniprot.org/uniprot/O43174	https://www.genenames.org/data/gene‐symbol‐report/#!/hgnc_id/HGNC:20581, http://www.uniprot.org/uniprot/Q9NR63	https://www.genenames.org/data/gene‐symbol‐report/#!/hgnc_id/HGNC:20577, http://www.uniprot.org/uniprot/Q6V0L0	https://www.genenames.org/data/gene‐symbol‐report/#!/hgnc_id/HGNC:2605, http://www.uniprot.org/uniprot/Q02318	https://www.genenames.org/data/gene‐symbol‐report/#!/hgnc_id/HGNC:2606, http://www.uniprot.org/uniprot/O15528	https://www.genenames.org/data/gene‐symbol‐report/#!/hgnc_id/HGNC:33480, http://www.uniprot.org/uniprot/Q4G0S4
EC number	http://www.genome.jp/dbget‐bin/www_bget?ec:1.14.15.16	http://www.genome.jp/dbget‐bin/www_bget?ec:1.14.‐.‐	http://www.genome.jp/dbget‐bin/www_bget?ec:1.14.‐.‐	http://www.genome.jp/dbget‐bin/www_bget?ec:1.14.‐.‐	http://www.genome.jp/dbget‐bin/www_bget?ec:1.14.15.15	http://www.genome.jp/dbget‐bin/www_bget?ec:1.14.15.18	http://www.genome.jp/dbget‐bin/www_bget?ec:1.14.19.53
Inhibitors	http://www.guidetopharmacology.org/GRAC/LigandDisplayForward?ligandId=8858 (pIC_50_ 8.1) [http://www.ncbi.nlm.nih.gov/pubmed/15615534?dopt=AbstractPlus], http://www.guidetopharmacology.org/GRAC/LigandDisplayForward?ligandId=8819 (pIC_50_ 8.1) [http://www.ncbi.nlm.nih.gov/pubmed/15615534?dopt=AbstractPlus], http://www.guidetopharmacology.org/GRAC/LigandDisplayForward?ligandId=8852 (pIC_50_ 4.8) [http://www.ncbi.nlm.nih.gov/pubmed/20655626?dopt=AbstractPlus]	–	–	–	http://www.guidetopharmacology.org/GRAC/LigandDisplayForward?ligandId=8852 (pIC_50_ 7.2) [http://www.ncbi.nlm.nih.gov/pubmed/20655626?dopt=AbstractPlus], http://www.guidetopharmacology.org/GRAC/LigandDisplayForward?ligandId=8858 (pIC_50_ <6) [http://www.ncbi.nlm.nih.gov/pubmed/15615534?dopt=AbstractPlus]	http://www.guidetopharmacology.org/GRAC/LigandDisplayForward?ligandId=8858 (pIC_50_ 6.3) [http://www.ncbi.nlm.nih.gov/pubmed/15615534?dopt=AbstractPlus]	–
Selective inhibitors	–	http://www.guidetopharmacology.org/GRAC/LigandDisplayForward?ligandId=8790 (pIC_50_ 9.5) [http://www.ncbi.nlm.nih.gov/pubmed/21838328?dopt=AbstractPlus]	–	–	–	–	–
Comments	Converts http://www.guidetopharmacology.org/GRAC/LigandDisplayForward?ligandId=2779 (calcitriol) to 1α,24R,25‐trihydroxyvitamin D_3_.	Converts retinoic acid to 4‐hydroxyretinoic acid. Inhibited by http://www.guidetopharmacology.org/GRAC/LigandDisplayForward?ligandId=5210	Converts retinoic acid to 4‐hydroxyretinoic acid.	Converts retinoic acid to 4‐hydroxyretinoic acid [http://www.ncbi.nlm.nih.gov/pubmed/14532297?dopt=AbstractPlus].	Converts http://www.guidetopharmacology.org/GRAC/LigandDisplayForward?ligandId=2718 to http://www.guidetopharmacology.org/GRAC/LigandDisplayForward?ligandId=2752.	Converts 25‐hydroxyvitamin D_3_ to http://www.guidetopharmacology.org/GRAC/LigandDisplayForward?ligandId=2779 (calcitriol)	Converts http://www.guidetopharmacology.org/GRAC/LigandDisplayForward?ligandId=4053 (vitamin A1) to 3,4‐didehydroretinol (vitamin A2) [http://www.ncbi.nlm.nih.gov/pubmed/27059013?dopt=AbstractPlus].

## http://www.guidetopharmacology.org/GRAC/FamilyDisplayForward?familyId=268

**Table:** 

Nomenclature	http://www.guidetopharmacology.org/GRAC/ObjectDisplayForward?objectId=1372	http://www.guidetopharmacology.org/GRAC/ObjectDisplayForward?objectId=1373	http://www.guidetopharmacology.org/GRAC/ObjectDisplayForward?objectId=1374
Common abbreviation	–	Cholesterol 24‐hydroxylase	Lanosterol 14‐α‐demethylase
HGNC, UniProt	https://www.genenames.org/data/gene‐symbol‐report/#!/hgnc_id/HGNC:17449, http://www.uniprot.org/uniprot/Q9NYL5	https://www.genenames.org/data/gene‐symbol‐report/#!/hgnc_id/HGNC:2641, http://www.uniprot.org/uniprot/Q9Y6A2	https://www.genenames.org/data/gene‐symbol‐report/#!/hgnc_id/HGNC:2649, http://www.uniprot.org/uniprot/Q16850
EC number	http://www.genome.jp/dbget‐bin/www_bget?ec:1.14.14.26	http://www.genome.jp/dbget‐bin/www_bget?ec:1.14.14.25	–
Inhibitors	–	–	http://www.guidetopharmacology.org/GRAC/LigandDisplayForward?ligandId=8799 (p*K*_i_ 9.1) [http://www.ncbi.nlm.nih.gov/pubmed/8340925?dopt=AbstractPlus]
Comments	Converts 24‐hydroxycholesterol to 7α,24‐dihydroxycholesterol [http://www.ncbi.nlm.nih.gov/pubmed/10748047?dopt=AbstractPlus].	Converts http://www.guidetopharmacology.org/GRAC/LigandDisplayForward?ligandId=2718 to http://www.guidetopharmacology.org/GRAC/LigandDisplayForward?ligandId=2750.	Converts http://www.guidetopharmacology.org/GRAC/LigandDisplayForward?ligandId=2746 to 4,4‐dimethylcholesta‐8.14.24‐trienol.

### Further reading on Cytochrome P450

Backman JT *et al*. (2016) Role of Cytochrome P450 2C8 in Drug Metabolism and Interactions. *Pharmacol Rev* **68**: 168‐241 https://www.ncbi.nlm.nih.gov/pubmed/26721703?dopt=AbstractPlus

Davis CM *et al*. (2017) Cytochrome P450 eicosanoids in cerebrovascular function and disease. *Pharmacol Ther* **179**: 31‐46 https://www.ncbi.nlm.nih.gov/pubmed/28527918?dopt=AbstractPlus

Ghosh D *et al*. (2016) Recent Progress in the Discovery of Next Generation Inhibitors of Aromatase from the Structure‐Function Perspective. *J Med Chem*. **59**: 5131‐48 https://www.ncbi.nlm.nih.gov/pubmed/26689671?dopt=AbstractPlus

Go RE *et al*. (2015) Cytochrome P450 1 family and cancers. *J Steroid Biochem Mol Biol*. **147**: 24‐30 https://www.ncbi.nlm.nih.gov/pubmed/25448748?dopt=AbstractPlus

Guengerich FP *et al*. (2016) Recent Structural Insights into Cytochrome P450 Function. *Trends Pharmacol Sci* **37**: 625‐640 https://www.ncbi.nlm.nih.gov/pubmed/27267697?dopt=AbstractPlus

Imig JD. (2018) Prospective for cytochrome P450 epoxygenase cardiovascular and renal therapeutics. *Pharmacol Ther* **192**: 1‐19 https://www.ncbi.nlm.nih.gov/pubmed/29964123?dopt=AbstractPlus

Isvoran A *et al*. (2017) Pharmacogenomics of the cytochrome P450 2C family: impacts of amino acid variations on drug metabolism. *Drug Discov Today* **22**: 366‐376 https://www.ncbi.nlm.nih.gov/pubmed/27693711?dopt=AbstractPlus

Jamieson KL *et al*. (2017) Cytochrome P450‐derived eicosanoids and heart function. *Pharmacol Ther* **179**: 47–83 https://www.ncbi.nlm.nih.gov/pubmed/28551025?dopt=AbstractPlus

Mak PJ *et al*. (2018) Spectroscopic studies of the cytochrome P450 reaction mechanisms. *Biochim Biophys Acta* **1866**: 178‐204 https://www.ncbi.nlm.nih.gov/pubmed/28668640?dopt=AbstractPlus

Moutinho M *et al*. (2016) Cholesterol 24‐hydroxylase: Brain cholesterol metabolism and beyond. *Biochim Biophys Acta* **1861**: 1911‐1920 https://www.ncbi.nlm.nih.gov/pubmed/27663182?dopt=AbstractPlus

Shalan H *et al*. (2018) Keeping the spotlight on cytochrome P450. *Biochim Biophys Acta* **1866**: 80‐87 https://www.ncbi.nlm.nih.gov/pubmed/28599858?dopt=AbstractPlus

## http://www.guidetopharmacology.org/GRAC/FamilyDisplayForward?familyId=851

### Overview

DNA topoisomerases regulate the supercoiling of nuclear DNA to influence the capacity for replication or transcription. The enzymatic function of this series of enzymes involves cutting the DNA to allow unwinding, followed by re‐attachment to reseal the backbone. Members of the family are targetted in anti‐cancer chemotherapy.

**Table:** 

Nomenclature	http://www.guidetopharmacology.org/GRAC/ObjectDisplayForward?objectId=2636	http://www.guidetopharmacology.org/GRAC/ObjectDisplayForward?objectId=2637
HGNC, UniProt	https://www.genenames.org/data/gene‐symbol‐report/#!/hgnc_id/HGNC:11986, http://www.uniprot.org/uniprot/P11387	https://www.genenames.org/data/gene‐symbol‐report/#!/hgnc_id/HGNC:11989, http://www.uniprot.org/uniprot/P11388
EC number	http://www.genome.jp/dbget‐bin/www_bget?ec:5.99.1.2	http://www.genome.jp/dbget‐bin/www_bget?ec:5.99.1.2
Inhibitors	http://www.guidetopharmacology.org/GRAC/LigandDisplayForward?ligandId=6823 [http://www.ncbi.nlm.nih.gov/pubmed/9655905?dopt=AbstractPlus, http://www.ncbi.nlm.nih.gov/pubmed/8182764?dopt=AbstractPlus] – Bovine	http://www.guidetopharmacology.org/GRAC/LigandDisplayForward?ligandId=6815 (pIC_50_ 7.3), http://www.guidetopharmacology.org/GRAC/LigandDisplayForward?ligandId=6843 [http://www.ncbi.nlm.nih.gov/pubmed/2557897?dopt=AbstractPlus] – Mouse

### Further reading on DNA topoisomerases

Bansal S *et al*. (2017) Topoisomerases: Resistance versus Sensitivity, How Far We Can Go? *Med Res Rev* **37**: 404‐438 https://www.ncbi.nlm.nih.gov/pubmed/27687257?dopt=AbstractPlus

Capranico G *et al*. (2017) Type I DNA Topoisomerases. *J. Med. Chem*. **60**: 2169‐2192 https://www.ncbi.nlm.nih.gov/pubmed/28072526?dopt=AbstractPlus

Nagaraja V *et al*. (2017) DNA topoisomerase I and DNA gyrase as targets for TB therapy. *Drug Discov. Today* **22**: 510‐518 https://www.ncbi.nlm.nih.gov/pubmed/27856347?dopt=AbstractPlus

Pommier Y *et al*. (2016) Roles of eukaryotic topoisomerases in transcription, replication and genomic stability. *Nat. Rev. Mol. Cell Biol*. **17**: 703‐721 https://www.ncbi.nlm.nih.gov/pubmed/27649880?dopt=AbstractPlus

Seol Y *et al*. (2016) The dynamic interplay between DNA topoisomerases and DNA topology. *Biophys Rev* **8**: 101‐111 https://www.ncbi.nlm.nih.gov/pubmed/28510219?dopt=AbstractPlus

## http://www.guidetopharmacology.org/GRAC/FamilyDisplayForward?familyId=943

### Overview

The principle endocannabinoids are 2‐acylglycerol esters, such as http://www.guidetopharmacology.org/GRAC/LigandDisplayForward?ligandId=729 (2‐AG), and *N*‐acylethanolamines, such as http://www.guidetopharmacology.org/GRAC/LigandDisplayForward?ligandId=2364 (*N*‐arachidonoylethanolamine, AEA). The glycerol esters and ethanolamides are synthesised and hydrolysed by parallel, independent pathways. Mechanisms for release and re‐uptake of endocannabinoids are unclear, although potent and selective inhibitors of facilitated diffusion of endocannabinoids across cell membranes have been developed [http://www.ncbi.nlm.nih.gov/pubmed/29531087?dopt=AbstractPlus]. http://www.guidetopharmacology.org/GRAC/ObjectDisplayForward?objectId=2535 (https://www.uniprot.org/uniprot/Q01469) has been suggested to act as a canonical intracellular endocannabinoid transporter *in vivo* [http://www.ncbi.nlm.nih.gov/pubmed/28584105?dopt=AbstractPlus]. For the generation of http://www.guidetopharmacology.org/GRAC/LigandDisplayForward?ligandId=729, the key enzyme involved is diacylglycerol lipase (DAGL), whilst several routes for http://www.guidetopharmacology.org/GRAC/LigandDisplayForward?ligandId=2364 synthesis have been described, the best characterized of which involves *N*‐acylphosphatidylethanolamine‐phospholipase D (NAPE‐PLD, [http://www.ncbi.nlm.nih.gov/pubmed/20393650?dopt=AbstractPlus]). A transacylation enzyme which forms *N*‐acylphosphatidylethanolamines has been identified as a cytosolic enzyme, https://www.genenames.org/data/gene‐symbol‐report/#!/hgnc_id/HGNC:24791 (http://www.uniprot.org/uniprot/Q3MJ16) [http://www.ncbi.nlm.nih.gov/pubmed/27399000?dopt=AbstractPlus]. *In vitro* experiments indicate that the endocannabinoids are also substrates for oxidative metabolism *via* cyclooxygenase, lipoxygenase and cytochrome P450 enzyme activities [http://www.ncbi.nlm.nih.gov/pubmed/17876303?dopt=AbstractPlus, http://www.ncbi.nlm.nih.gov/pubmed/17618306?dopt=AbstractPlus, http://www.ncbi.nlm.nih.gov/pubmed/20133390?dopt=AbstractPlus].

## http://www.guidetopharmacology.org/GRAC/FamilyDisplayForward?familyId=273

**Table:** 

Nomenclature	http://www.guidetopharmacology.org/GRAC/ObjectDisplayForward?objectId=1398	http://www.guidetopharmacology.org/GRAC/ObjectDisplayForward?objectId=1400	http://www.guidetopharmacology.org/GRAC/ObjectDisplayForward?objectId=1401	http://www.guidetopharmacology.org/GRAC/ObjectDisplayForward?objectId=1402
Common abbreviation	NAPE‐PLD	FAAH	FAAH2	NAAA
HGNC, UniProt	https://www.genenames.org/data/gene‐symbol‐report/#!/hgnc_id/HGNC:21683, http://www.uniprot.org/uniprot/Q6IQ20	https://www.genenames.org/data/gene‐symbol‐report/#!/hgnc_id/HGNC:3553, http://www.uniprot.org/uniprot/O00519	https://www.genenames.org/data/gene‐symbol‐report/#!/hgnc_id/HGNC:26440, http://www.uniprot.org/uniprot/Q6GMR7	https://www.genenames.org/data/gene‐symbol‐report/#!/hgnc_id/HGNC:736, http://www.uniprot.org/uniprot/Q02083
EC number	–	http://www.genome.jp/dbget‐bin/www_bget?ec:3.5.1.99: anandamide + H_2_O <=> arachidonic acid + ethanolamine oleamide + H_2_O <=> oleic acid + NH_3_	http://www.genome.jp/dbget‐bin/www_bget?ec:3.5.1.99: anandamide + H_2_O <=> arachidonic acid + ethanolamine oleamide + H_2_O <=> oleic acid + NH_3_	http://www.genome.jp/dbget‐bin/www_bget?ec:3.5.1.‐
Rank order of affinity	–	http://www.guidetopharmacology.org/GRAC/LigandDisplayForward?ligandId=2364 > http://www.guidetopharmacology.org/GRAC/LigandDisplayForward?ligandId=284 > http://www.guidetopharmacology.org/GRAC/LigandDisplayForward?ligandId=2661 > http://www.guidetopharmacology.org/GRAC/LigandDisplayForward?ligandId=3622 [http://www.ncbi.nlm.nih.gov/pubmed/17015445?dopt=AbstractPlus]	http://www.guidetopharmacology.org/GRAC/LigandDisplayForward?ligandId=284 > http://www.guidetopharmacology.org/GRAC/LigandDisplayForward?ligandId=2661 > http://www.guidetopharmacology.org/GRAC/LigandDisplayForward?ligandId=2364 > http://www.guidetopharmacology.org/GRAC/LigandDisplayForward?ligandId=3622 [http://www.ncbi.nlm.nih.gov/pubmed/17015445?dopt=AbstractPlus]	http://www.guidetopharmacology.org/GRAC/LigandDisplayForward?ligandId=3622 > http://www.guidetopharmacology.org/GRAC/LigandDisplayForward?ligandId=5221 > http://www.guidetopharmacology.org/GRAC/LigandDisplayForward?ligandId=3621 ≥ http://www.guidetopharmacology.org/GRAC/LigandDisplayForward?ligandId=2661 > http://www.guidetopharmacology.org/GRAC/LigandDisplayForward?ligandId=2364 [http://www.ncbi.nlm.nih.gov/pubmed/11463796?dopt=AbstractPlus]
Selective inhibitors	–	http://www.guidetopharmacology.org/GRAC/LigandDisplayForward?ligandId=10248 (pIC_50_ 8.4) [http://www.ncbi.nlm.nih.gov/pubmed/29017758?dopt=AbstractPlus], http://www.guidetopharmacology.org/GRAC/LigandDisplayForward?ligandId=5206 (pIC_50_ 7.8) [http://www.ncbi.nlm.nih.gov/pubmed/18693015?dopt=AbstractPlus], http://www.guidetopharmacology.org/GRAC/LigandDisplayForward?ligandId=5244 (pIC_50_ 6.3–7.8) [http://www.ncbi.nlm.nih.gov/pubmed/17949010?dopt=AbstractPlus], http://www.guidetopharmacology.org/GRAC/LigandDisplayForward?ligandId=5235 (pIC_50_ 7.4) [http://www.ncbi.nlm.nih.gov/pubmed/17015445?dopt=AbstractPlus], http://www.guidetopharmacology.org/GRAC/LigandDisplayForward?ligandId=9944 (pIC_50_ 7), http://www.guidetopharmacology.org/GRAC/LigandDisplayForward?ligandId=4339 (pIC_50_ 6.3–7) [http://www.ncbi.nlm.nih.gov/pubmed/17015445?dopt=AbstractPlus], http://www.guidetopharmacology.org/GRAC/LigandDisplayForward?ligandId=5243 (pIC_50_ 6.6) [http://www.ncbi.nlm.nih.gov/pubmed/19389627?dopt=AbstractPlus]	http://www.guidetopharmacology.org/GRAC/LigandDisplayForward?ligandId=5235 (pIC_50_ 7.9–8.4) [http://www.ncbi.nlm.nih.gov/pubmed/19095868?dopt=AbstractPlus, http://www.ncbi.nlm.nih.gov/pubmed/17015445?dopt=AbstractPlus], http://www.guidetopharmacology.org/GRAC/LigandDisplayForward?ligandId=4339 (pIC_50_ 7.5–8.3) [http://www.ncbi.nlm.nih.gov/pubmed/19095868?dopt=AbstractPlus, http://www.ncbi.nlm.nih.gov/pubmed/17015445?dopt=AbstractPlus], http://www.guidetopharmacology.org/GRAC/LigandDisplayForward?ligandId=10248 (pIC_50_ 7.2) [http://www.ncbi.nlm.nih.gov/pubmed/29017758?dopt=AbstractPlus]	http://www.guidetopharmacology.org/GRAC/LigandDisplayForward?ligandId=10121 (pIC_50_ 8.1) [http://www.ncbi.nlm.nih.gov/pubmed/28802121?dopt=AbstractPlus, http://www.ncbi.nlm.nih.gov/pubmed/29572189?dopt=AbstractPlus], http://www.guidetopharmacology.org/GRAC/LigandDisplayForward?ligandId=9178 (Irreversible inhibition) (pIC_50_ 7.6) [http://www.ncbi.nlm.nih.gov/pubmed/25874594?dopt=AbstractPlus], http://www.guidetopharmacology.org/GRAC/LigandDisplayForward?ligandId=6493 (pIC_50_ 6.4) [http://www.ncbi.nlm.nih.gov/pubmed/19926854?dopt=AbstractPlus] – Rat, http://www.guidetopharmacology.org/GRAC/LigandDisplayForward?ligandId=5161 (pIC_50_ 5.3) [http://www.ncbi.nlm.nih.gov/pubmed/14686878?dopt=AbstractPlus]
Comments	NAPE‐PLD activity appears to be enhanced by polyamines in the physiological range [http://www.ncbi.nlm.nih.gov/pubmed/12047899?dopt=AbstractPlus], but fails to transphosphatidylate with alcohols [http://www.ncbi.nlm.nih.gov/pubmed/10428468?dopt=AbstractPlus] unlike phosphatidylcholine‐specific phospholipase D.	–	The FAAH2 gene is found in many primate genomes, marsupials, and other distantly related vertebrates, but not a variety of lower placental mammals, including mouse and rat [http://www.ncbi.nlm.nih.gov/pubmed/17015445?dopt=AbstractPlus].	–

### Comments

Routes for *N*‐acylethanolamine biosynthesis other than through NAPE‐PLD activity have been identified [http://www.ncbi.nlm.nih.gov/pubmed/23394527?dopt=AbstractPlus].

## http://www.guidetopharmacology.org/GRAC/FamilyDisplayForward?familyId=944

### Overview

ABHD12 is a 398‐aa protein, with serine hydrolase activity. It has a molecular weight of 45 kDa. A single TM is predicted at 75‐95, with an extracellular catalytic domain. ABHD12 is a monoacylglycerol hydrolase [http://www.ncbi.nlm.nih.gov/pubmed/22969151?dopt=AbstractPlus], but may also regulate lysophosphatidylserine levels [http://www.ncbi.nlm.nih.gov/pubmed/25580854?dopt=AbstractPlus]. Loss‐of‐function mutations in ABHD12 are associated with a disorder known as PHARC (polyneuropathy, hearing loss, ataxia, retinitis pigmentosa, and cataracts) [http://www.ncbi.nlm.nih.gov/pubmed/20797687?dopt=AbstractPlus].

**Table:** 

Nomenclature	http://www.guidetopharmacology.org/GRAC/ObjectDisplayForward?objectId=1396	http://www.guidetopharmacology.org/GRAC/ObjectDisplayForward?objectId=1397	http://www.guidetopharmacology.org/GRAC/ObjectDisplayForward?objectId=1399	http://www.guidetopharmacology.org/GRAC/ObjectDisplayForward?objectId=2919	http://www.guidetopharmacology.org/GRAC/ObjectDisplayForward?objectId=3070
Common abbreviation	DAGLα	DAGLß	MAGL	ABHD6	–
HGNC, UniProt	https://www.genenames.org/data/gene‐symbol‐report/#!/hgnc_id/HGNC:1165, http://www.uniprot.org/uniprot/Q9Y4D2	https://www.genenames.org/data/gene‐symbol‐report/#!/hgnc_id/HGNC:28923, http://www.uniprot.org/uniprot/Q8NCG7	https://www.genenames.org/data/gene‐symbol‐report/#!/hgnc_id/HGNC:17038, http://www.uniprot.org/uniprot/Q99685	https://www.genenames.org/data/gene‐symbol‐report/#!/hgnc_id/HGNC:21398, http://www.uniprot.org/uniprot/Q9BV23	https://www.genenames.org/data/gene‐symbol‐report/#!/hgnc_id/HGNC:15868, http://www.uniprot.org/uniprot/Q8N2K0
EC number	http://www.genome.jp/dbget‐bin/www_bget?ec:3.1.1.‐	http://www.genome.jp/dbget‐bin/www_bget?ec:3.1.1.‐	http://www.genome.jp/dbget‐bin/www_bget?ec:3.1.1.23	http://www.genome.jp/dbget‐bin/www_bget?ec:3.1.1.23	http://www.genome.jp/dbget‐bin/www_bget?ec:3.1.1.23
Endogenous substrates	diacylglycerol	diacylglycerol	http://www.guidetopharmacology.org/GRAC/LigandDisplayForward?ligandId=5112 = http://www.guidetopharmacology.org/GRAC/LigandDisplayForward?ligandId=729 ≫ http://www.guidetopharmacology.org/GRAC/LigandDisplayForward?ligandId=2364 [http://www.ncbi.nlm.nih.gov/pubmed/15492019?dopt=AbstractPlus]	1‐arachidonoylglycerol > http://www.guidetopharmacology.org/GRAC/LigandDisplayForward?ligandId=729 > 1‐oleoylglycerol > http://www.guidetopharmacology.org/GRAC/LigandDisplayForward?ligandId=5112 [http://www.ncbi.nlm.nih.gov/pubmed/22969151?dopt=AbstractPlus]	–
Inhibitors	http://www.guidetopharmacology.org/GRAC/LigandDisplayForward?ligandId=10246 (pIC_50_ 8.5) [http://www.ncbi.nlm.nih.gov/pubmed/26083464?dopt=AbstractPlus], http://www.guidetopharmacology.org/GRAC/LigandDisplayForward?ligandId=10244 (pIC_50_ 8.2) [http://www.ncbi.nlm.nih.gov/pubmed/26668358?dopt=AbstractPlus], http://www.guidetopharmacology.org/GRAC/LigandDisplayForward?ligandId=10243 (pIC_50_ 8.2) [http://www.ncbi.nlm.nih.gov/pubmed/26668358?dopt=AbstractPlus], http://www.guidetopharmacology.org/GRAC/LigandDisplayForward?ligandId=10245 (pIC_50_ 5.6) [http://www.ncbi.nlm.nih.gov/pubmed/23103940?dopt=AbstractPlus]	http://www.guidetopharmacology.org/GRAC/LigandDisplayForward?ligandId=10244 (pIC_50_ 8.6) [http://www.ncbi.nlm.nih.gov/pubmed/26668358?dopt=AbstractPlus], http://www.guidetopharmacology.org/GRAC/LigandDisplayForward?ligandId=10243 (pIC_50_ 8.1) [http://www.ncbi.nlm.nih.gov/pubmed/26668358?dopt=AbstractPlus], http://www.guidetopharmacology.org/GRAC/LigandDisplayForward?ligandId=10246 (pIC_50_ 8.1) [http://www.ncbi.nlm.nih.gov/pubmed/26083464?dopt=AbstractPlus], http://www.guidetopharmacology.org/GRAC/LigandDisplayForward?ligandId=10245 (pIC_50_ 7.1) [http://www.ncbi.nlm.nih.gov/pubmed/23103940?dopt=AbstractPlus]	http://www.guidetopharmacology.org/GRAC/LigandDisplayForward?ligandId=10028 (pIC_50_ 8) [http://www.ncbi.nlm.nih.gov/pubmed/23731016?dopt=AbstractPlus]	–	–
Selective inhibitors	–	–	http://www.guidetopharmacology.org/GRAC/LigandDisplayForward?ligandId=9481 (pIC_50_ 9.3) [http://www.ncbi.nlm.nih.gov/pubmed/23521796?dopt=AbstractPlus], http://www.guidetopharmacology.org/GRAC/LigandDisplayForward?ligandId=9482 (pIC_50_ 8.5) [http://www.ncbi.nlm.nih.gov/pubmed/22542104?dopt=AbstractPlus], http://www.guidetopharmacology.org/GRAC/LigandDisplayForward?ligandId=5207 (pIC_50_ 8.1) [http://www.ncbi.nlm.nih.gov/pubmed/19029917?dopt=AbstractPlus]	http://www.guidetopharmacology.org/GRAC/LigandDisplayForward?ligandId=5289 (pIC_50_ 7.2) [http://www.ncbi.nlm.nih.gov/pubmed/17629278?dopt=AbstractPlus], http://www.guidetopharmacology.org/GRAC/LigandDisplayForward?ligandId=9480 (pIC_50_ 6.4) [http://www.ncbi.nlm.nih.gov/pubmed/21084632?dopt=AbstractPlus]	http://www.guidetopharmacology.org/GRAC/LigandDisplayForward?ligandId=10250 (pIC_50_ 8) [http://www.ncbi.nlm.nih.gov/pubmed/30720278?dopt=AbstractPlus]
Comments	–	–	–	http://www.guidetopharmacology.org/GRAC/LigandDisplayForward?ligandId=5289 has also been suggested to have activity at oxidative metabolic pathways independent of ABHD6 [http://www.ncbi.nlm.nih.gov/pubmed/28086912?dopt=AbstractPlus].	–

### Comments on Endocannabinoid turnover

Many of the compounds described as inhibitors are irreversible and so potency estimates will vary with incubation time. FAAH2 is not found in rodents [http://www.ncbi.nlm.nih.gov/pubmed/17015445?dopt=AbstractPlus] and a limited range of inhibitors have been assessed at this enzyme activity. http://www.guidetopharmacology.org/GRAC/LigandDisplayForward?ligandId=729 has been reported to be hydrolysed by multiple enzyme activities from neural preparations [http://www.ncbi.nlm.nih.gov/pubmed/29751000?dopt=AbstractPlus], including https://www.genenames.org/data/gene‐symbol‐report/#!/hgnc_id/HGNC:21398 (http://www.uniprot.org/uniprot/Q9BV23) [http://www.ncbi.nlm.nih.gov/pubmed/26989199?dopt=AbstractPlus] and carboxylesterase 1 (https://www.genenames.org/data/gene‐symbol‐report/#!/hgnc_id/HGNC:1863, http://www.uniprot.org/uniprot/P23141 [http://www.ncbi.nlm.nih.gov/pubmed/21049984?dopt=AbstractPlus]). https://www.genenames.org/data/gene‐symbol‐report/#!/hgnc_id/HGNC:21398 (http://www.uniprot.org/uniprot/Q9BV23) has also been described as a triacylglycerol lipase and ester hydrolase [http://www.ncbi.nlm.nih.gov/pubmed/27247428?dopt=AbstractPlus], while https://www.genenames.org/data/gene‐symbol‐report/#!/hgnc_id/HGNC:15868 (http://www.uniprot.org/uniprot/Q8N2K0) is also able to hydrolyse lysophosphatidylserine [http://www.ncbi.nlm.nih.gov/pubmed/2397193?dopt=AbstractPlus]. https://www.genenames.org/data/gene‐symbol‐report/#!/hgnc_id/HGNC:15868 (http://www.uniprot.org/uniprot/Q8N2K0) has been described to be inhibited selectively by pentacyclic triterpenoids, such as oleanolic acid [http://www.ncbi.nlm.nih.gov/pubmed/24879289?dopt=AbstractPlus].

### Further reading on Endocannabinoid turnover

Blankman JL *et al*. (2013) Chemical probes of endocannabinoid metabolism. *Pharmacol. Rev*. **65**: 849–71 https://www.ncbi.nlm.nih.gov/pubmed/23512546?dopt=AbstractPlus

Cao JK *et al*. (2019) ABHD6: Its Place in Endocannabinoid Signaling and Beyond. *Trends Pharmacol Sci* **40**: 267–277 https://www.ncbi.nlm.nih.gov/pubmed/30853109

Di Marzo V. (2018) New approaches and challenges to targeting the endocannabinoid system. *Nat Rev Drug Discov* **17**: 623–639 https://www.ncbi.nlm.nih.gov/pubmed/30116049

Fowler CJ. (2017) Endocannabinoid Turnover. *Adv Pharmacol* **80**: 31–66 https://www.ncbi.nlm.nih.gov/pubmed/28826539

Janssen FJ *et al*. (2016) Inhibitors of diacylglycerol lipases in neurodegenerative and metabolic disorders. *Bioorg Med Chem Lett* **26**: 3831–7 https://www.ncbi.nlm.nih.gov/pubmed/27394666?dopt=AbstractPlus

Maccarrone M. (2017) Metabolism of the Endocannabinoid Anandamide: Open Questions after 25 Years. *Front Mol Neurosci* **10**: 166 https://www.ncbi.nlm.nih.gov/pubmed/28611591?dopt=AbstractPlus

Nicolussi S *et al*. (2015) Endocannabinoid transport revisited. *Vitam Horm* **98**: 441–85 https://www.ncbi.nlm.nih.gov/pubmed/25817877?dopt=AbstractPlus

Tsuboi K *et al*. (2018) Endocannabinoids and related N‐acylethanolamines: biological activities and metabolism. *Inflamm Regen* **38**: 28 https://www.ncbi.nlm.nih.gov/pubmed/30288203

## http://www.guidetopharmacology.org/GRAC/FamilyDisplayForward?familyId=243

### Overview

Eicosanoids are 20‐carbon fatty acids, where the usual focus is the polyunsaturated analogue http://www.guidetopharmacology.org/GRAC/LigandDisplayForward?ligandId=2391 and its metabolites. Arachidonic acid is thought primarily to derive from http://www.guidetopharmacology.org/GRAC/FamilyDisplayForward?familyId=244#Phospholipase A2 action on membrane phosphatidylcholine, andmay be re‐cycled to form phospholipid through conjugation with http://www.guidetopharmacology.org/GRAC/LigandDisplayForward?ligandId=3044 and subsequently glycerol derivatives. Oxidative metabolism of arachidonic acid is conducted through three major enzymatic routes: cyclooxygenases; lipoxygenases and cytochrome P450‐like epoxygenases, particularly http://www.guidetopharmacology.org/GRAC/FamilyDisplayForward?familyId=242#show_object_1332. Isoprostanes are structural analogues of the prostanoids (hence the nomenclature D‐, E‐, F‐isoprostanes and isothromboxanes), which are produced in the presence of elevated free radicals in a nonenzymaticmanner, leading to suggestions for their use as biomarkers of oxidative stress. Molecular targets for their action have yet to be defined.

## http://www.guidetopharmacology.org/GRAC/FamilyDisplayForward?familyId=269

### Overview

Prostaglandin (PG) G/H synthase, most commonly referred to as cyclooxygenase (COX, (5Z,8Z,11Z,14Z)‐icosa‐5,8,11,14‐tetraenoate,hydrogen‐donor : oxygen oxidoreductase) activity, catalyses the formation of http://www.guidetopharmacology.org/GRAC/LigandDisplayForward?ligandId=5245 from http://www.guidetopharmacology.org/GRAC/LigandDisplayForward?ligandId=2391. Hydroperoxidase activity inherent in the enzyme catalyses the formation of http://www.guidetopharmacology.org/GRAC/LigandDisplayForward?ligandId=4483 from http://www.guidetopharmacology.org/GRAC/LigandDisplayForward?ligandId=5245. COX‐1 and ‐2 can be nonselectively inhibited by http://www.guidetopharmacology.org/GRAC/LigandDisplayForward?ligandId=2713, http://www.guidetopharmacology.org/GRAC/LigandDisplayForward?ligandId=4795, http://www.guidetopharmacology.org/GRAC/LigandDisplayForward?ligandId=5230, http://www.guidetopharmacology.org/GRAC/LigandDisplayForward?ligandId=1909 and http://www.guidetopharmacology.org/GRAC/LigandDisplayForward?ligandId=5239 (acetaminophen). PGH_2_ may then be metabolised to prostaglandins and thromboxanes by various prostaglandin synthases in an apparently tissue‐dependent manner.

**Table:** 

Nomenclature	http://www.guidetopharmacology.org/GRAC/ObjectDisplayForward?objectId=1375	http://www.guidetopharmacology.org/GRAC/ObjectDisplayForward?objectId=1376
HGNC, UniProt	https://www.genenames.org/data/gene‐symbol‐report/#!/hgnc_id/HGNC:9604, http://www.uniprot.org/uniprot/P23219	https://www.genenames.org/data/gene‐symbol‐report/#!/hgnc_id/HGNC:9605, http://www.uniprot.org/uniprot/P35354
EC number	http://www.genome.jp/dbget‐bin/www_bget?ec:1.14.99.1: Hydrogen donor + http://www.guidetopharmacology.org/GRAC/LigandDisplayForward?ligandId=2391 + 2O_2_ = hydrogen acceptor + H_2_O + http://www.guidetopharmacology.org/GRAC/LigandDisplayForward?ligandId=4483 http://www.guidetopharmacology.org/GRAC/LigandDisplayForward?ligandId=2391 => http://www.guidetopharmacology.org/GRAC/LigandDisplayForward?ligandId=5245 => http://www.guidetopharmacology.org/GRAC/LigandDisplayForward?ligandId=4483 This enzyme is also associated with the following reaction:: http://www.guidetopharmacology.org/GRAC/LigandDisplayForward?ligandId=1051 => PGH_3_	http://www.genome.jp/dbget‐bin/www_bget?ec:1.14.99.1: Hydrogen donor + http://www.guidetopharmacology.org/GRAC/LigandDisplayForward?ligandId=2391 + 2O_2_ = hydrogen acceptor + H_2_O + http://www.guidetopharmacology.org/GRAC/LigandDisplayForward?ligandId=4483 http://www.guidetopharmacology.org/GRAC/LigandDisplayForward?ligandId=2391 => http://www.guidetopharmacology.org/GRAC/LigandDisplayForward?ligandId=5245 => http://www.guidetopharmacology.org/GRAC/LigandDisplayForward?ligandId=4483 This enzyme is also associated with the following reaction:: http://www.guidetopharmacology.org/GRAC/LigandDisplayForward?ligandId=1051 => PGH_3_
Selective activators	–	http://www.guidetopharmacology.org/GRAC/LigandDisplayForward?ligandId=10242 (Inhibition) (pIC_50_ 8) [http://www.ncbi.nlm.nih.gov/pubmed/9135032?dopt=AbstractPlus]
Inhibitors	http://www.guidetopharmacology.org/GRAC/LigandDisplayForward?ligandId=7131 (pIC_50_ 8.1) [22], http://www.guidetopharmacology.org/GRAC/LigandDisplayForward?ligandId=2714 (pIC_50_ 7.9) [http://www.ncbi.nlm.nih.gov/pubmed/16252917?dopt=AbstractPlus], http://www.guidetopharmacology.org/GRAC/LigandDisplayForward?ligandId=7219 (pIC_50_ 7.3) [http://www.ncbi.nlm.nih.gov/pubmed/11844663?dopt=AbstractPlus], http://www.guidetopharmacology.org/GRAC/LigandDisplayForward?ligandId=4194 (pIC_50_ 7.1) [http://www.ncbi.nlm.nih.gov/pubmed/10377455?dopt=AbstractPlus], http://www.guidetopharmacology.org/GRAC/LigandDisplayForward?ligandId=4820 (pIC_50_ 6.8) [22], http://www.guidetopharmacology.org/GRAC/LigandDisplayForward?ligandId=4795 (pIC_50_ 6.5) [http://www.ncbi.nlm.nih.gov/pubmed/18667313?dopt=AbstractPlus], http://www.guidetopharmacology.org/GRAC/LigandDisplayForward?ligandId=7298 (pIC_50_ 6.2) [http://www.ncbi.nlm.nih.gov/pubmed/18667313?dopt=AbstractPlus]	http://www.guidetopharmacology.org/GRAC/LigandDisplayForward?ligandId=7124 (pIC_50_ 8.3) [22], http://www.guidetopharmacology.org/GRAC/LigandDisplayForward?ligandId=4194 (pIC_50_ 8) [http://www.ncbi.nlm.nih.gov/pubmed/10091674?dopt=AbstractPlus], http://www.guidetopharmacology.org/GRAC/LigandDisplayForward?ligandId=7219 (pIC_50_ 7.4) [http://www.ncbi.nlm.nih.gov/pubmed/11844663?dopt=AbstractPlus], http://www.guidetopharmacology.org/GRAC/LigandDisplayForward?ligandId=7141 (pIC_50_ 7) [http://www.ncbi.nlm.nih.gov/pubmed/21873070?dopt=AbstractPlus], http://www.guidetopharmacology.org/GRAC/LigandDisplayForward?ligandId=6661 (pIC_50_ 6.9) [http://www.ncbi.nlm.nih.gov/pubmed/22091869?dopt=AbstractPlus], http://www.guidetopharmacology.org/GRAC/LigandDisplayForward?ligandId=7401 (pIC_50_ 6.2) [http://www.ncbi.nlm.nih.gov/pubmed/15993594?dopt=AbstractPlus], http://www.guidetopharmacology.org/GRAC/LigandDisplayForward?ligandId=4795 (pIC_50_ 6.2) [http://www.ncbi.nlm.nih.gov/pubmed/18667313?dopt=AbstractPlus]
Selective inhibitors	http://www.guidetopharmacology.org/GRAC/LigandDisplayForward?ligandId=6661 (pIC_50_ 9.7) [http://www.ncbi.nlm.nih.gov/pubmed/10377455?dopt=AbstractPlus], http://www.guidetopharmacology.org/GRAC/LigandDisplayForward?ligandId=10241 (pIC_50_ 8.3) [http://www.ncbi.nlm.nih.gov/pubmed/21745460?dopt=AbstractPlus], http://www.guidetopharmacology.org/GRAC/LigandDisplayForward?ligandId=10240 (pIC_50_ 8.1) [http://www.ncbi.nlm.nih.gov/pubmed/9789085?dopt=AbstractPlus], http://www.guidetopharmacology.org/GRAC/LigandDisplayForward?ligandId=5191 (pIC_50_ 7.5) [http://www.ncbi.nlm.nih.gov/pubmed/10720634?dopt=AbstractPlus]	http://www.guidetopharmacology.org/GRAC/LigandDisplayForward?ligandId=2892 (pIC_50_ 8.7) [http://www.ncbi.nlm.nih.gov/pubmed/12643942?dopt=AbstractPlus], http://www.guidetopharmacology.org/GRAC/LigandDisplayForward?ligandId=2894 (pIC_50_ 8.3) [http://www.ncbi.nlm.nih.gov/pubmed/10715145?dopt=AbstractPlus], http://www.guidetopharmacology.org/GRAC/LigandDisplayForward?ligandId=2714 (pIC_50_ 7.7) [http://www.ncbi.nlm.nih.gov/pubmed/17341061?dopt=AbstractPlus], http://www.guidetopharmacology.org/GRAC/LigandDisplayForward?ligandId=2893 (pIC_50_ 6.1–6.5) [http://www.ncbi.nlm.nih.gov/pubmed/10377455?dopt=AbstractPlus], http://www.guidetopharmacology.org/GRAC/LigandDisplayForward?ligandId=2897 (p*K*_i_ 6.5) [http://www.ncbi.nlm.nih.gov/pubmed/17434872?dopt=AbstractPlus], http://www.guidetopharmacology.org/GRAC/LigandDisplayForward?ligandId=7220 (pIC_50_ 6.3) [http://www.ncbi.nlm.nih.gov/pubmed/9083488?dopt=AbstractPlus], http://www.guidetopharmacology.org/GRAC/LigandDisplayForward?ligandId=2896 (pIC_50_ 6) [http://www.ncbi.nlm.nih.gov/pubmed/11160644?dopt=AbstractPlus]

## http://www.guidetopharmacology.org/GRAC/FamilyDisplayForward?familyId=270

### Overview

Subsequent to the formation of http://www.guidetopharmacology.org/GRAC/LigandDisplayForward?ligandId=4483, the http://www.guidetopharmacology.org/GRAC/FamilyDisplayForward?familyId=242 thromboxane synthase (CYP5A1, https://www.genenames.org/data/gene‐symbol‐report/#!/hgnc_id/HGNC:11609, http://www.uniprot.org/uniprot/P24557, http://www.genome.jp/kegg‐bin/search_brite?option=‐a&search_string=5.3.99.5) and prostacyclin synthase (CYP8A1, https://www.genenames.org/data/gene‐symbol‐report/#!/hgnc_id/HGNC:9603, http://www.uniprot.org/uniprot/Q16647, http://www.genome.jp/kegg‐bin/search_brite?option=‐a&search_string=5.3.99.4) generate http://www.guidetopharmacology.org/GRAC/LigandDisplayForward?ligandId=4482 and prostacyclin (http://www.guidetopharmacology.org/GRAC/LigandDisplayForward?ligandId=1915), respectively. Additionally, multiple enzyme activities are able to generate prostaglandin E_2_ (http://www.guidetopharmacology.org/GRAC/LigandDisplayForward?ligandId=1883), prostaglandin D_2_ (http://www.guidetopharmacology.org/GRAC/LigandDisplayForward?ligandId=1881) and prostaglandin F_2α_ (http://www.guidetopharmacology.org/GRAC/LigandDisplayForward?ligandId=1884). PGD_2_ can bemetabolised to 9α,11ß‐prostacyclin F_2α_ through the multifunctional enzyme activity of AKR1C3. PGE2 can be metabolised to http://www.guidetopharmacology.org/GRAC/LigandDisplayForward?ligandId=5129 through the 9‐ketoreductase activity of CBR1. Conversion of the 15‐hydroxyecosanoids, including prostaglandins, lipoxins and leukotrienes to their keto derivatives by the NAD‐dependent enzyme HPGD leads to a reduction in their biological activity.

**Table:** 

Nomenclature	http://www.guidetopharmacology.org/GRAC/ObjectDisplayForward?objectId=1353	http://www.guidetopharmacology.org/GRAC/ObjectDisplayForward?objectId=1356	http://www.guidetopharmacology.org/GRAC/ObjectDisplayForward?objectId=1377	http://www.guidetopharmacology.org/GRAC/ObjectDisplayForward?objectId=1378	http://www.guidetopharmacology.org/GRAC/ObjectDisplayForward?objectId=1379	http://www.guidetopharmacology.org/GRAC/ObjectDisplayForward?objectId=1380
Common abbreviation	Thromboxane synthase	Prostacyclin synthase	–	–	–	–
HGNC, UniProt	https://www.genenames.org/data/gene‐symbol‐report/#!/hgnc_id/HGNC:11609, http://www.uniprot.org/uniprot/P24557	https://www.genenames.org/data/gene‐symbol‐report/#!/hgnc_id/HGNC:9603, http://www.uniprot.org/uniprot/Q16647	https://www.genenames.org/data/gene‐symbol‐report/#!/hgnc_id/HGNC:9599, http://www.uniprot.org/uniprot/O14684	https://www.genenames.org/data/gene‐symbol‐report/#!/hgnc_id/HGNC:17822, http://www.uniprot.org/uniprot/Q9H7Z7	https://www.genenames.org/data/gene‐symbol‐report/#!/hgnc_id/HGNC:16049, http://www.uniprot.org/uniprot/Q15185	https://www.genenames.org/data/gene‐symbol‐report/#!/hgnc_id/HGNC:9592, http://www.uniprot.org/uniprot/P41222
EC number	http://www.genome.jp/dbget‐bin/www_bget?ec:5.3.99.5: http://www.guidetopharmacology.org/GRAC/LigandDisplayForward?ligandId=4483 = http://www.guidetopharmacology.org/GRAC/LigandDisplayForward?ligandId=4482	http://www.genome.jp/dbget‐bin/www_bget?ec:5.3.99.4	http://www.genome.jp/dbget‐bin/www_bget?ec:5.3.99.3: http://www.guidetopharmacology.org/GRAC/LigandDisplayForward?ligandId=4483 = http://www.guidetopharmacology.org/GRAC/LigandDisplayForward?ligandId=1883	http://www.genome.jp/dbget‐bin/www_bget?ec:5.3.99.3: http://www.guidetopharmacology.org/GRAC/LigandDisplayForward?ligandId=4483 = http://www.guidetopharmacology.org/GRAC/LigandDisplayForward?ligandId=1883	http://www.genome.jp/dbget‐bin/www_bget?ec:5.3.99.3: http://www.guidetopharmacology.org/GRAC/LigandDisplayForward?ligandId=4483 = http://www.guidetopharmacology.org/GRAC/LigandDisplayForward?ligandId=1883	http://www.genome.jp/dbget‐bin/www_bget?ec:5.3.99.2: http://www.guidetopharmacology.org/GRAC/LigandDisplayForward?ligandId=4483 = http://www.guidetopharmacology.org/GRAC/LigandDisplayForward?ligandId=1881
Cofactors	–	–	http://www.guidetopharmacology.org/GRAC/LigandDisplayForward?ligandId=6737	http://www.guidetopharmacology.org/GRAC/LigandDisplayForward?ligandId=6738	–	–
Inhibitors	http://www.guidetopharmacology.org/GRAC/LigandDisplayForward?ligandId=9866 (pIC_50_ 8.4) [http://www.ncbi.nlm.nih.gov/pubmed/3093741?dopt=AbstractPlus]	http://www.guidetopharmacology.org/GRAC/LigandDisplayForward?ligandId=8795 (pIC_50_ > 6) [http://www.ncbi.nlm.nih.gov/pubmed/7861416?dopt=AbstractPlus]	http://www.guidetopharmacology.org/GRAC/LigandDisplayForward?ligandId=8761 (pIC_50_ 9) [http://www.ncbi.nlm.nih.gov/pubmed/19748780?dopt=AbstractPlus]	http://www.guidetopharmacology.org/GRAC/LigandDisplayForward?ligandId=8812 (pIC_50_ <6) [http://www.ncbi.nlm.nih.gov/pubmed/15953724?dopt=AbstractPlus]	–	–
Selective inhibitors	–	–	http://www.guidetopharmacology.org/GRAC/LigandDisplayForward?ligandId=8875 (pIC_50_ 8.4) [http://www.ncbi.nlm.nih.gov/pubmed/23623673?dopt=AbstractPlus]	–	–	AT‐56 (pKi 4.1) [http://www.ncbi.nlm.nih.gov/pubmed/19131342?dopt=AbstractPlus]
Comments	Inhibited by http://www.guidetopharmacology.org/GRAC/LigandDisplayForward?ligandId=5175 [http://www.ncbi.nlm.nih.gov/pubmed/6795753?dopt=AbstractPlus], http://www.guidetopharmacology.org/GRAC/LigandDisplayForward?ligandId=5157 [http://www.ncbi.nlm.nih.gov/pubmed/7778318?dopt=AbstractPlus] and furegrelate sodium (U‐63557A: PubChem https://pubchem.ncbi.nlm.nih.gov/compound/23663954) [http://www.ncbi.nlm.nih.gov/pubmed/6316421?dopt=AbstractPlus].	Converts http://www.guidetopharmacology.org/GRAC/LigandDisplayForward?ligandId=4483 to http://www.guidetopharmacology.org/GRAC/LigandDisplayForward?ligandId=1915 [http://www.ncbi.nlm.nih.gov/pubmed/8766713?dopt=AbstractPlus]. Inhibited by http://www.guidetopharmacology.org/GRAC/LigandDisplayForward?ligandId=5281 [http://www.ncbi.nlm.nih.gov/pubmed/824685?dopt=AbstractPlus]	–	–	Phosphorylated and activated by casein kinase 2 (http://www.guidetopharmacology.org/GRAC/ObjectDisplayForward?familyId=565&objectId=1548&familyType=ENZYME) [http://www.ncbi.nlm.nih.gov/pubmed/15040786?dopt=AbstractPlus]. Appears to regulate steroid hormone function by interaction with dimeric hsp90 [http://www.ncbi.nlm.nih.gov/pubmed/11050175?dopt=AbstractPlus, http://www.ncbi.nlm.nih.gov/pubmed/8603045?dopt=AbstractPlus].	–

**Table:** 

Nomenclature	http://www.guidetopharmacology.org/GRAC/ObjectDisplayForward?objectId=1381	http://www.guidetopharmacology.org/GRAC/ObjectDisplayForward?objectId=1382	http://www.guidetopharmacology.org/GRAC/ObjectDisplayForward?objectId=1383	http://www.guidetopharmacology.org/GRAC/ObjectDisplayForward?objectId=1384
HGNC, UniProt	https://www.genenames.org/data/gene‐symbol‐report/#!/hgnc_id/HGNC:17890, http://www.uniprot.org/uniprot/O60760	http://www.guidetopharmacology.org/GRAC/ObjectDisplayForward?objectId=1382, http://www.uniprot.org/uniprot/P42330	http://www.guidetopharmacology.org/GRAC/ObjectDisplayForward?objectId=1383, http://www.uniprot.org/uniprot/P16152	http://www.guidetopharmacology.org/GRAC/ObjectDisplayForward?objectId=1384, http://www.uniprot.org/uniprot/P15428
EC number	http://www.genome.jp/dbget‐bin/www_bget?ec:5.3.99.2: http://www.guidetopharmacology.org/GRAC/LigandDisplayForward?ligandId=4483 = http://www.guidetopharmacology.org/GRAC/LigandDisplayForward?ligandId=1881	http://www.genome.jp/dbget‐bin/www_bget?ec:1.3.1.20 http://www.genome.jp/dbget‐bin/www_bget?ec:1.1.1.188: http://www.guidetopharmacology.org/GRAC/LigandDisplayForward?ligandId=1881 + http://www.guidetopharmacology.org/GRAC/LigandDisplayForward?ligandId=3045 = http://www.guidetopharmacology.org/GRAC/LigandDisplayForward?ligandId=1884 + http://www.guidetopharmacology.org/GRAC/LigandDisplayForward?ligandId=3041 + H^+^ http://www.genome.jp/dbget‐bin/www_bget?ec:1.1.1.64 http://www.genome.jp/dbget‐bin/www_bget?ec:1.1.1.239 http://www.genome.jp/dbget‐bin/www_bget?ec:1.1.1.213	http://www.genome.jp/dbget‐bin/www_bget?ec:1.1.1.184 http://www.genome.jp/dbget‐bin/www_bget?ec:1.1.1.189: http://www.guidetopharmacology.org/GRAC/LigandDisplayForward?ligandId=1883 + http://www.guidetopharmacology.org/GRAC/LigandDisplayForward?ligandId=3045 = http://www.guidetopharmacology.org/GRAC/LigandDisplayForward?ligandId=1884 + http://www.guidetopharmacology.org/GRAC/LigandDisplayForward?ligandId=3041 + H^+^ http://www.genome.jp/dbget‐bin/www_bget?ec:1.1.1.197	http://www.genome.jp/dbget‐bin/www_bget?ec:1.1.1.141 15‐hydroxyprostaglandins => 15‐ketoprostaglandins http://www.guidetopharmacology.org/GRAC/LigandDisplayForward?ligandId=1034 => 15‐keto‐lipoxin A_4_
Cofactors	–	http://www.guidetopharmacology.org/GRAC/LigandDisplayForward?ligandId=3045	http://www.guidetopharmacology.org/GRAC/LigandDisplayForward?ligandId=3045	–
Inhibitors	http://www.guidetopharmacology.org/GRAC/LigandDisplayForward?ligandId=6662 (pIC_50_ 5.3–5.5) [http://www.ncbi.nlm.nih.gov/pubmed/16547010?dopt=AbstractPlus]	http://www.guidetopharmacology.org/GRAC/LigandDisplayForward?ligandId=8769 (p*K*_i_ 8.1) [http://www.ncbi.nlm.nih.gov/pubmed/17166832?dopt=AbstractPlus] http://www.guidetopharmacology.org/GRAC/LigandDisplayForward?ligandId=2447, http://www.guidetopharmacology.org/GRAC/LigandDisplayForward?ligandId=1909, flavonoids such as 2′‐Hydroxyflavanone (pIC_50_ 6.5) [http://www.ncbi.nlm.nih.gov/pubmed/9792917?dopt=AbstractPlus, http://www.ncbi.nlm.nih.gov/pubmed/19007764?dopt=AbstractPlus]	http://www.guidetopharmacology.org/GRAC/LigandDisplayForward?ligandId=5551 (pIC_50_ 5.4) [http://www.ncbi.nlm.nih.gov/pubmed/19097799?dopt=AbstractPlus]	http://www.guidetopharmacology.org/GRAC/LigandDisplayForward?ligandId=8745 (pIC_50_ 8.1) [http://www.ncbi.nlm.nih.gov/pubmed/21650226?dopt=AbstractPlus]
Comments	–	AKR1C3 also exhibits an hydroxysteroid dehydrogenase activity.	–	–

### Comments

http://www.guidetopharmacology.org/GRAC/LigandDisplayForward?ligandId=5293 has been reported to inhibit mPGES1 and 5‐LOX with a *p*IC_50_ value of 5.5 [http://www.ncbi.nlm.nih.gov/pubmed/19053751?dopt=AbstractPlus].

## http://www.guidetopharmacology.org/GRAC/FamilyDisplayForward?familyId=271

### Overview

The lipoxygenases (LOXs) are a structurally related family of non‐heme iron dioxygenases that function in the production, and in some cases metabolism, of fatty acid hydroperoxides. For http://www.guidetopharmacology.org/GRAC/LigandDisplayForward?ligandId=2391 as substrate, these products are hydroperoxyeicosatetraenoic acids (HPETEs). In humans there are five lipoxygenases, the 5S‐(arachidonate : oxygen 5‐oxidoreductase), 12R‐(arachidonate 12‐lipoxygenase, 12R‐type), 12S‐(arachidonate : oxygen 12‐oxidoreductase), and two distinct 15S‐(arachidonate : oxygen 15‐oxidoreductase) LOXs that oxygenate arachidonic acid in different positions along the carbon chain and form the corresponding 5S‐, 12S‐, 12R‐, or 15S‐hydroperoxides, respectively.

**Table:** 

Nomenclature	http://www.guidetopharmacology.org/GRAC/ObjectDisplayForward?objectId=1385	http://www.guidetopharmacology.org/GRAC/ObjectDisplayForward?objectId=1386	http://www.guidetopharmacology.org/GRAC/ObjectDisplayForward?objectId=1387	http://www.guidetopharmacology.org/GRAC/ObjectDisplayForward?objectId=1388	http://www.guidetopharmacology.org/GRAC/ObjectDisplayForward?objectId=1389	http://www.guidetopharmacology.org/GRAC/ObjectDisplayForward?objectId=1390
HGNC, UniProt	https://www.genenames.org/data/gene‐symbol‐report/#!/hgnc_id/HGNC:435, http://www.uniprot.org/uniprot/P09917	https://www.genenames.org/data/gene‐symbol‐report/#!/hgnc_id/HGNC:430, http://www.uniprot.org/uniprot/O75342	https://www.genenames.org/data/gene‐symbol‐report/#!/hgnc_id/HGNC:429, http://www.uniprot.org/uniprot/P18054	https://www.genenames.org/data/gene‐symbol‐report/#!/hgnc_id/HGNC:433, http://www.uniprot.org/uniprot/P16050	https://www.genenames.org/data/gene‐symbol‐report/#!/hgnc_id/HGNC:434, http://www.uniprot.org/uniprot/O15296	https://www.genenames.org/data/gene‐symbol‐report/#!/hgnc_id/HGNC:13743, http://www.uniprot.org/uniprot/Q9BYJ1
EC number	http://www.genome.jp/dbget‐bin/www_bget?ec:1.13.11.34: http://www.guidetopharmacology.org/GRAC/LigandDisplayForward?ligandId=2391 + O_2_ = http://www.guidetopharmacology.org/GRAC/LigandDisplayForward?ligandId=5214 + H_2_O	http://www.genome.jp/dbget‐bin/www_bget?ec:1.13.11.31 http://www.guidetopharmacology.org/GRAC/LigandDisplayForward?ligandId=2391 + O_2_ => http://www.guidetopharmacology.org/GRAC/LigandDisplayForward?ligandId=5101	http://www.genome.jp/dbget‐bin/www_bget?ec:1.13.11.31 http://www.guidetopharmacology.org/GRAC/LigandDisplayForward?ligandId=2391 + O_2_ => http://www.guidetopharmacology.org/GRAC/LigandDisplayForward?ligandId=2481	http://www.genome.jp/dbget‐bin/www_bget?ec:1.13.11.33: http://www.guidetopharmacology.org/GRAC/LigandDisplayForward?ligandId=2391 + O_2_ = http://www.guidetopharmacology.org/GRAC/LigandDisplayForward?ligandId=2482 http://www.guidetopharmacology.org/GRAC/LigandDisplayForward?ligandId=1052 + O_2_ => 13S‐HPODE	http://www.genome.jp/dbget‐bin/www_bget?ec:1.13.11.33: http://www.guidetopharmacology.org/GRAC/LigandDisplayForward?ligandId=2391 + O_2_ = http://www.guidetopharmacology.org/GRAC/LigandDisplayForward?ligandId=2482	http://www.genome.jp/dbget‐bin/www_bget?ec:1.13.11.‐
Substrates	–	http://www.guidetopharmacology.org/GRAC/LigandDisplayForward?ligandId=5222	–	–	–	–
Endogenous substrates	http://www.guidetopharmacology.org/GRAC/LigandDisplayForward?ligandId=2391	–	–	–	–	http://www.guidetopharmacology.org/GRAC/LigandDisplayForward?ligandId=5101
Endogenous activators	http://www.guidetopharmacology.org/GRAC/LigandDisplayForward?ligandId=5183 (https://www.genenames.org/data/gene‐symbol‐report/#!/hgnc_id/HGNC:436, http://www.uniprot.org/uniprot/P20292)	–	–	–	–	–
Endogenous inhibitors	Protein kinase A‐mediated phosphorylation [http://www.ncbi.nlm.nih.gov/pubmed/15280375?dopt=AbstractPlus]	–	–	–	–	–
Inhibitors	–	–	–	http://www.guidetopharmacology.org/GRAC/LigandDisplayForward?ligandId=10263 (pIC_50_ 6.7) [http://www.ncbi.nlm.nih.gov/pubmed/24672829?dopt=AbstractPlus]	http://www.guidetopharmacology.org/GRAC/LigandDisplayForward?ligandId=8750 (pIC_50_ 7.3) [http://www.ncbi.nlm.nih.gov/pubmed/17656086?dopt=AbstractPlus]	–
Selective inhibitors	http://www.guidetopharmacology.org/GRAC/LigandDisplayForward?ligandId=5169 (pIC_50_ 7.2) [http://www.ncbi.nlm.nih.gov/pubmed/15197110?dopt=AbstractPlus], http://www.guidetopharmacology.org/GRAC/LigandDisplayForward?ligandId=9054 (pIC_50_ 6.6) [http://www.ncbi.nlm.nih.gov/pubmed/20378715?dopt=AbstractPlus], http://www.guidetopharmacology.org/GRAC/LigandDisplayForward?ligandId=5297 (pIC_50_ 6.4) [http://www.ncbi.nlm.nih.gov/pubmed/1848634?dopt=AbstractPlus]	–	http://www.guidetopharmacology.org/GRAC/LigandDisplayForward?ligandId=8752 (pIC_50_ 6.5) [http://www.ncbi.nlm.nih.gov/pubmed/24393039?dopt=AbstractPlus]	http://www.guidetopharmacology.org/GRAC/LigandDisplayForward?ligandId=8751 (p*K*_i_ >8) [http://www.ncbi.nlm.nih.gov/pubmed/20866075?dopt=AbstractPlus]	–	–
Comments	FLAP activity can be inhibited by http://www.guidetopharmacology.org/GRAC/LigandDisplayForward?ligandId=2655 [http://www.ncbi.nlm.nih.gov/pubmed/2300173?dopt=AbstractPlus] and http://www.guidetopharmacology.org/GRAC/LigandDisplayForward?ligandId=5148 [http://www.ncbi.nlm.nih.gov/pubmed/8381000?dopt=AbstractPlus] leading to a selective inhibition of 5‐LOX activity	–	–	–	Inhibited by http://www.guidetopharmacology.org/GRAC/LigandDisplayForward?ligandId=10264 (pK_i_ 5.6) [http://www.ncbi.nlm.nih.gov/pubmed/25111178?dopt=AbstractPlus].	E‐LOX metabolises the product from the 12R‐lipoxygenase (12R‐HPETE) to a specific epoxyalcohol compound [http://www.ncbi.nlm.nih.gov/pubmed/12881489?dopt=AbstractPlus].

### Comments

An 8‐LOX(http://www.genome.jp/kegg‐bin/search_brite?option=‐a&search_string=1.13.11.40, arachidonate:oxygen 8‐oxidoreductase) may be the mouse orthologue of 15‐LOX‐2 [http://www.ncbi.nlm.nih.gov/pubmed/12432921?dopt=AbstractPlus]. Some general LOX inhibitors are http://www.guidetopharmacology.org/GRAC/LigandDisplayForward?ligandId=4265 and http://www.guidetopharmacology.org/GRAC/LigandDisplayForward?ligandId=5180. http://www.guidetopharmacology.org/GRAC/LigandDisplayForward?ligandId=5297 and http://www.guidetopharmacology.org/GRAC/LigandDisplayForward?ligandId=5155 are used as 5‐lipoxygenase inhibitors, while http://www.guidetopharmacology.org/GRAC/LigandDisplayForward?ligandId=5144 and http://www.guidetopharmacology.org/GRAC/LigandDisplayForward?ligandId=5162 are 12‐lipoxygenase inhibitors. The specificity of these inhibitors has not been rigorously assessed with all LOX forms: http://www.guidetopharmacology.org/GRAC/LigandDisplayForward?ligandId=5144, along with other flavonoids, such as http://www.guidetopharmacology.org/GRAC/LigandDisplayForward?ligandId=5182 and http://www.guidetopharmacology.org/GRAC/LigandDisplayForward?ligandId=5215, also inhibits 15‐LOX‐1 [http://www.ncbi.nlm.nih.gov/pubmed/12628491?dopt=AbstractPlus].

## http://www.guidetopharmacology.org/GRAC/FamilyDisplayForward?familyId=272

### Overview

Leukotriene A_4_ (http://www.guidetopharmacology.org/GRAC/LigandDisplayForward?ligandId=5214), produced by 5‐LOX activity, and lipoxins may be subject to further oxidative metabolism; ω‐hydroxylation is mediated by http://www.guidetopharmacology.org/GRAC/FamilyDisplayForward?familyId=242#show_object_1344 and http://www.guidetopharmacology.org/GRAC/FamilyDisplayForward?familyId=242#show_object_1345, while ß‐oxidation in mitochondria and peroxisomes proceeds in a manner dependent on coenzyme A conjugation. Conjugation of http://www.guidetopharmacology.org/GRAC/LigandDisplayForward?ligandId=5214 at the 6 position with reduced glutathione to generate http://www.guidetopharmacology.org/GRAC/LigandDisplayForward?ligandId=3354 occurs under the influence of leukotriene C_4_ synthase, with the subsequent formation of http://www.guidetopharmacology.org/GRAC/LigandDisplayForward?ligandId=3353 and http://www.guidetopharmacology.org/GRAC/LigandDisplayForward?ligandId=3352, all three of which are agonists at http://www.guidetopharmacology.org/GRAC/FamilyDisplayForward?familyId=35. http://www.guidetopharmacology.org/GRAC/LigandDisplayForward?ligandId=3353 formation is catalysed by γ‐glutamyltransferase, and subsequently dipeptidase 2 removes the terminal http://www.guidetopharmacology.org/GRAC/LigandDisplayForward?ligandId=727 from http://www.guidetopharmacology.org/GRAC/LigandDisplayForward?ligandId=3353 to generate http://www.guidetopharmacology.org/GRAC/LigandDisplayForward?ligandId=3352. Leukotriene A_4_ hydrolase converts the 5,6‐epoxide LTA_4_ to the 5‐hydroxylated LTB_4_, an agonist for http://www.guidetopharmacology.org/GRAC/FamilyDisplayForward?familyId=35. http://www.guidetopharmacology.org/GRAC/LigandDisplayForward?ligandId=5214 is also acted upon by 12S‐LOX to produce the trihydroxyeicosatetraenoic acids lipoxins http://www.guidetopharmacology.org/GRAC/LigandDisplayForward?ligandId=1034 and http://www.guidetopharmacology.org/GRAC/LigandDisplayForward?ligandId=5216. Treatment with a LTA_4_ hydrolase inhibitor in a murine model of allergic airway inflammation increased LXA_4_ levels, in addition to reducing http://www.guidetopharmacology.org/GRAC/LigandDisplayForward?ligandId=2487, in lung lavage fluid [http://www.ncbi.nlm.nih.gov/pubmed/20110560?dopt=AbstractPlus]. LTA_4_ hydrolase is also involved in biosynthesis of http://www.guidetopharmacology.org/GRAC/FamilyDisplayForward?familyId=134. http://www.guidetopharmacology.org/GRAC/LigandDisplayForward?ligandId=4139 has been reported to increase endogenous formation of 18S‐hydroxyeicosapentaenoate (http://www.guidetopharmacology.org/GRAC/LigandDisplayForward?ligandId=5105) compared with http://www.guidetopharmacology.org/GRAC/LigandDisplayForward?ligandId=3400, a resolvin precursor. Both enantiomers may be metabolised by human recombinant 5‐LOX; recombinant LTA_4_ hydrolase converted chiral 5S(6)‐epoxide‐containing intermediates to http://www.guidetopharmacology.org/GRAC/LigandDisplayForward?ligandId=3333 and http://www.guidetopharmacology.org/GRAC/LigandDisplayForward?ligandId=5106 [http://www.ncbi.nlm.nih.gov/pubmed/21206090?dopt=AbstractPlus].

**Table:** 

Nomenclature	http://www.guidetopharmacology.org/GRAC/ObjectDisplayForward?objectId=1391	http://www.guidetopharmacology.org/GRAC/ObjectDisplayForward?objectId=1392	http://www.guidetopharmacology.org/GRAC/ObjectDisplayForward?objectId=1393	http://www.guidetopharmacology.org/GRAC/ObjectDisplayForward?objectId=1394	http://www.guidetopharmacology.org/GRAC/ObjectDisplayForward?objectId=1395
HGNC, UniProt	https://www.genenames.org/data/gene‐symbol‐report/#!/hgnc_id/HGNC:6719, http://www.uniprot.org/uniprot/Q16873	https://www.genenames.org/data/gene‐symbol‐report/#!/hgnc_id/HGNC:21705, http://www.uniprot.org/uniprot/O75223	https://www.genenames.org/data/gene‐symbol‐report/#!/hgnc_id/HGNC:3002, http://www.uniprot.org/uniprot/P16444	https://www.genenames.org/data/gene‐symbol‐report/#!/hgnc_id/HGNC:23028, http://www.uniprot.org/uniprot/Q9H4A9	https://www.genenames.org/data/gene‐symbol‐report/#!/hgnc_id/HGNC:6710, http://www.uniprot.org/uniprot/P09960
EC number	http://www.genome.jp/dbget‐bin/www_bget?ec:4.4.1.20: http://www.guidetopharmacology.org/GRAC/LigandDisplayForward?ligandId=3354 = http://www.guidetopharmacology.org/GRAC/LigandDisplayForward?ligandId=6737 + http://www.guidetopharmacology.org/GRAC/LigandDisplayForward?ligandId=5214	http://www.genome.jp/dbget‐bin/www_bget?ec:2.3.2.2: (5‐L‐glutamyl)‐peptide + an amino acid = a peptide + a 5‐L‐glutamyl amino acid http://www.guidetopharmacology.org/GRAC/LigandDisplayForward?ligandId=3354 + H_2_O => http://www.guidetopharmacology.org/GRAC/LigandDisplayForward?ligandId=3353 + L‐glutamate	http://www.genome.jp/dbget‐bin/www_bget?ec:3.4.13.19: http://www.guidetopharmacology.org/GRAC/LigandDisplayForward?ligandId=3353 + H_2_O = http://www.guidetopharmacology.org/GRAC/LigandDisplayForward?ligandId=3352 + http://www.guidetopharmacology.org/GRAC/LigandDisplayForward?ligandId=727	http://www.genome.jp/dbget‐bin/www_bget?ec:3.4.13.19: http://www.guidetopharmacology.org/GRAC/LigandDisplayForward?ligandId=3353 + H_2_O = http://www.guidetopharmacology.org/GRAC/LigandDisplayForward?ligandId=3352 + http://www.guidetopharmacology.org/GRAC/LigandDisplayForward?ligandId=727	http://www.genome.jp/dbget‐bin/www_bget?ec:3.3.2.6
Inhibitors	http://www.guidetopharmacology.org/GRAC/LigandDisplayForward?ligandId=9555 (pIC_50_ 8.1) [508], http://www.guidetopharmacology.org/GRAC/LigandDisplayForward?ligandId=8875 (pIC_50_ <5.5) [551]	http://www.guidetopharmacology.org/GRAC/LigandDisplayForward?ligandId=10265 (p*K*_i_ 3.8) [http://www.ncbi.nlm.nih.gov/pubmed/17260973?dopt=AbstractPlus]	http://www.guidetopharmacology.org/GRAC/LigandDisplayForward?ligandId=5166 (p*K*_i_ 6) [http://www.ncbi.nlm.nih.gov/pubmed/3495664?dopt=AbstractPlus]	–	http://www.guidetopharmacology.org/GRAC/LigandDisplayForward?ligandId=5151 (p*K*_i_ 5.4) [http://www.ncbi.nlm.nih.gov/pubmed/1846352?dopt=AbstractPlus]

### Comments

LTA4H is a member of a family of arginyl aminopeptidases (http://www.ensembl.org/Homo_sapiens/Gene/Family/Genes?family=ENSFM00250000001675), which also includes aminopeptidase B (https://www.genenames.org/data/gene‐symbol‐report/#!/hgnc_id/HGNC:10078, http://www.uniprot.org/uniprot/9H4A4) and aminopeptidase B‐like 1 (https://www.genenames.org/data/gene‐symbol‐report/#!/hgnc_id/HGNC:10079, http://www.uniprot.org/uniprot/Q9HAU8). Dipeptidase 1 and 2 are members of a family of membrane dipeptidases, which also includes (https://www.genenames.org/data/gene‐symbol‐report/#!/hgnc_id/HGNC:23029, http://www.uniprot.org/uniprot/Q9H4B8) for which http://www.guidetopharmacology.org/GRAC/LigandDisplayForward?ligandId=3353 appears not to be a substrate.

### Further reading on Eicosanoid turnover

Ackermann JA *et al*. (2017) The double‐edged role of 12/15‐lipoxygenase during inflammation and immunity. *Biochim. Biophys. Acta* **1862**: 371–381 https://www.ncbi.nlm.nih.gov/pubmed/27480217?dopt=AbstractPlus

Grosser T *et al*. (2017) The Cardiovascular Pharmacology of Nonsteroidal Anti‐Inflammatory Drugs. *Trends Pharmacol. Sci*. **38**: 733–748 https://www.ncbi.nlm.nih.gov/pubmed/28651847?dopt=AbstractPlus

Haeggstrom JZ. (2018) Leukotriene biosynthetic enzymes as therapeutic targets. *J Clin Invest* **128**: 2680–2690 https://www.ncbi.nlm.nih.gov/pubmed/30108195

Hafner AK *et al*. (2019) Beyond leukotriene formation‐The noncanonical functions of 5‐lipoxygenase. *Prostaglandins Other Lipid Mediat* **142**: 24–32 https://www.ncbi.nlm.nih.gov/pubmed/30930090

Mitchell JA and Kirkby NS. (2019) Eicosanoids, prostacyclin and cyclooxygenase in the cardiovascular system. *Br J Pharmacol* **176**: 1038–1050 https://www.ncbi.nlm.nih.gov/pubmed/29468666

Koeberle A *et al*. (2015) Perspective of microsomal prostaglandin E2 synthase‐1 as drug target in inflammation‐related disorders. *Biochem. Pharmacol*. **98**: 1–15 https://www.ncbi.nlm.nih.gov/pubmed/26123522?dopt=AbstractPlus

Kuhn H *et al*. (2015) Mammalian lipoxygenases and their biological relevance. *Biochim. Biophys. Acta* **1851**: 308–30 https://www.ncbi.nlm.nih.gov/pubmed/25316652?dopt=AbstractPlus

Patrignani P *et al*. (2015) Cyclooxygenase inhibitors: From pharmacology to clinical read‐outs. *Biochim. Biophys. Acta* **1851**: 422–32 https://www.ncbi.nlm.nih.gov/pubmed/25263946?dopt=AbstractPlus

Rådmark O *et al*. (2015) 5‐Lipoxygenase, a key enzyme for leukotriene biosynthesis in health and disease. *Biochim. Biophys. Acta* **1851**: 331–9 https://www.ncbi.nlm.nih.gov/pubmed/25152163?dopt=AbstractPlus

Sasaki Y *et al*. (2017) Role of prostacyclin synthase in carcinogenesis. *Prostaglandins Other Lipid Mediat*. **133**: 49–52 https://www.ncbi.nlm.nih.gov/pubmed/28506876?dopt=AbstractPlus

Seo MJ *et al*. (2017) Prostaglandin synthases: Molecular characterization and involvement in prostaglandin biosynthesis. *Prog. Lipid Res*. **66**: 50–68 https://www.ncbi.nlm.nih.gov/pubmed/28392405?dopt=AbstractPlus

Vitale P *et al*. (2016) COX‐1 Inhibitors: Beyond Structure Toward Therapy. *Med Res Rev* **36**: 641–71 https://www.ncbi.nlm.nih.gov/pubmed/27111555?dopt=AbstractPlus

## http://www.guidetopharmacology.org/GRAC/FamilyDisplayForward?familyId=764

### Overview

The inhibitory neurotransmitter γ‐aminobutyrate (GABA, 4‐aminobutyrate) is generated in neurones by glutamic acid decarboxylase. GAD1 and GAD2 are differentially expressed during development, whereGAD2 is thought to subserve a trophic role in early life and is distributed throughout the cytoplasm. GAD1 is expressed in later life and is more associated with nerve terminals [http://www.ncbi.nlm.nih.gov/pubmed/8126575?dopt=AbstractPlus] where GABA is principally accumulated in vesicles through the action of the vesicular inhibitory amino acid transporter http://www.guidetopharmacology.org/GRAC/FamilyDisplayForward?familyId=219. The role of γ‐aminobutyraldehyde dehydrogenase (ALDH9A1) in neurotransmitter GABA synthesis is less clear. Following release from neurons, GABA may interact with either GABA_A_ or GABA_B_ receptors and may be accumulated in neurones and glia through the action of members of the http://www.guidetopharmacology.org/GRAC/FamilyDisplayForward?familyId=144. Successive metabolism through GABA transaminase and succinate semialdehyde dehydrogenase generates succinic acid, which may be further metabolized in the mitochondria in the tricarboxylic acid cycle.

**Table:** 

Nomenclature	http://www.guidetopharmacology.org/GRAC/ObjectDisplayForward?objectId=1272	http://www.guidetopharmacology.org/GRAC/ObjectDisplayForward?objectId=1273
Common abbreviation	GAD1	GAD2
HGNC, UniProt	https://www.genenames.org/data/gene‐symbol‐report/#!/hgnc_id/HGNC:4092, http://www.uniprot.org/uniprot/Q99259	https://www.genenames.org/data/gene‐symbol‐report/#!/hgnc_id/HGNC:4093, http://www.uniprot.org/uniprot/Q05329
EC number	http://www.genome.jp/dbget‐bin/www_bget?ec:4.1.1.15: http://www.guidetopharmacology.org/GRAC/LigandDisplayForward?ligandId=1369 + H^+^ ‐> http://www.guidetopharmacology.org/GRAC/LigandDisplayForward?ligandId=1067 + CO_2_	http://www.genome.jp/dbget‐bin/www_bget?ec:4.1.1.15: http://www.guidetopharmacology.org/GRAC/LigandDisplayForward?ligandId=1369 + H^+^ ‐> http://www.guidetopharmacology.org/GRAC/LigandDisplayForward?ligandId=1067 + CO_2_
Endogenous substrates	http://www.guidetopharmacology.org/GRAC/LigandDisplayForward?ligandId=1369, http://www.guidetopharmacology.org/GRAC/LigandDisplayForward?ligandId=3309	http://www.guidetopharmacology.org/GRAC/LigandDisplayForward?ligandId=1369, http://www.guidetopharmacology.org/GRAC/LigandDisplayForward?ligandId=3309
Products	http://www.guidetopharmacology.org/GRAC/LigandDisplayForward?ligandId=1067	http://www.guidetopharmacology.org/GRAC/LigandDisplayForward?ligandId=1067
Cofactors	http://www.guidetopharmacology.org/GRAC/LigandDisplayForward?ligandId=5249	http://www.guidetopharmacology.org/GRAC/LigandDisplayForward?ligandId=5249
Selective inhibitors	http://www.guidetopharmacology.org/GRAC/LigandDisplayForward?ligandId=5267	http://www.guidetopharmacology.org/GRAC/LigandDisplayForward?ligandId=5267
Comments	http://www.guidetopharmacology.org/GRAC/LigandDisplayForward?ligandId=3309 is a less rapidly metabolised substrate of mouse brain glutamic acid decarboxylase generating ß‐alanine [http://www.ncbi.nlm.nih.gov/pubmed/4700449?dopt=AbstractPlus]. Autoantibodies against GAD1 and GAD2 are elevated in type 1 diabetes mellitus and neurological disorders (see Further reading).	

**Table:** 

Nomenclature	http://www.guidetopharmacology.org/GRAC/ObjectDisplayForward?objectId=2467	http://www.guidetopharmacology.org/GRAC/ObjectDisplayForward?objectId=2464	http://www.guidetopharmacology.org/GRAC/ObjectDisplayForward?objectId=2466
Common abbreviation	–	GABA‐T	SSADH
HGNC, UniProt	https://www.genenames.org/data/gene‐symbol‐report/#!/hgnc_id/HGNC:412, http://www.uniprot.org/uniprot/P49189	https://www.genenames.org/data/gene‐symbol‐report/#!/hgnc_id/HGNC:23, http://www.uniprot.org/uniprot/P80404	https://www.genenames.org/data/gene‐symbol‐report/#!/hgnc_id/HGNC:408, http://www.uniprot.org/uniprot/P51649
EC number	http://www.genome.jp/dbget‐bin/www_bget?ec:1.2.1.19: http://www.guidetopharmacology.org/GRAC/LigandDisplayForward?ligandId=6606 + http://www.guidetopharmacology.org/GRAC/LigandDisplayForward?ligandId=2451 + H_2_O = http://www.guidetopharmacology.org/GRAC/LigandDisplayForward?ligandId=1067 + http://www.guidetopharmacology.org/GRAC/LigandDisplayForward?ligandId=4487 + H^+^ http://www.genome.jp/dbget‐bin/www_bget?ec:1.2.1.47: http://www.guidetopharmacology.org/GRAC/LigandDisplayForward?ligandId=6604 + http://www.guidetopharmacology.org/GRAC/LigandDisplayForward?ligandId=2451 + H_2_O = http://www.guidetopharmacology.org/GRAC/LigandDisplayForward?ligandId=6605 + http://www.guidetopharmacology.org/GRAC/LigandDisplayForward?ligandId=3041 + 2H^+^ http://www.genome.jp/dbget‐bin/www_bget?ec:1.2.1.3: an aldehyde + H_2_O + http://www.guidetopharmacology.org/GRAC/LigandDisplayForward?ligandId=2451 = a carboxylate + 2H^+^ + http://www.guidetopharmacology.org/GRAC/LigandDisplayForward?ligandId=4487	http://www.genome.jp/dbget‐bin/www_bget?ec:2.6.1.19: http://www.guidetopharmacology.org/GRAC/LigandDisplayForward?ligandId=1067 + http://www.guidetopharmacology.org/GRAC/LigandDisplayForward?ligandId=3636 = http://www.guidetopharmacology.org/GRAC/LigandDisplayForward?ligandId=1369 + http://www.guidetopharmacology.org/GRAC/LigandDisplayForward?ligandId=6608 http://www.genome.jp/dbget‐bin/www_bget?ec:2.6.1.22: http://www.guidetopharmacology.org/GRAC/LigandDisplayForward?ligandId=6610 + http://www.guidetopharmacology.org/GRAC/LigandDisplayForward?ligandId=3636 = http://www.guidetopharmacology.org/GRAC/LigandDisplayForward?ligandId=6611 + http://www.guidetopharmacology.org/GRAC/LigandDisplayForward?ligandId=1369	http://www.genome.jp/dbget‐bin/www_bget?ec:1.2.1.24: http://www.guidetopharmacology.org/GRAC/LigandDisplayForward?ligandId=6608 + http://www.guidetopharmacology.org/GRAC/LigandDisplayForward?ligandId=2451 + H_2_O = http://www.guidetopharmacology.org/GRAC/LigandDisplayForward?ligandId=3637 + http://www.guidetopharmacology.org/GRAC/LigandDisplayForward?ligandId=4487 + 2H^+^ 4‐hydroxy‐trans‐2‐nonenal + http://www.guidetopharmacology.org/GRAC/LigandDisplayForward?ligandId=2451 + H_2_O = 4‐hydroxy‐trans‐2‐nonenoate + http://www.guidetopharmacology.org/GRAC/LigandDisplayForward?ligandId=4487 + 2H^+^
Cofactors	http://www.guidetopharmacology.org/GRAC/LigandDisplayForward?ligandId=2451	http://www.guidetopharmacology.org/GRAC/LigandDisplayForward?ligandId=5249	http://www.guidetopharmacology.org/GRAC/LigandDisplayForward?ligandId=2451 [http://www.ncbi.nlm.nih.gov/pubmed/22677141?dopt=AbstractPlus]
Inhibitors	–	http://www.guidetopharmacology.org/GRAC/LigandDisplayForward?ligandId=4821 (Irreversible inhibition) (p*K*_i_ 3.1) [http://www.ncbi.nlm.nih.gov/pubmed/856582?dopt=AbstractPlus, http://www.ncbi.nlm.nih.gov/pubmed/22168767?dopt=AbstractPlus]	http://www.guidetopharmacology.org/GRAC/LigandDisplayForward?ligandId=8841 (pIC_50_ 6.5) [http://www.ncbi.nlm.nih.gov/pubmed/16290145?dopt=AbstractPlus]

### Further reading on GABA turnover

Koenig MK *et al*. (2017) Phenotype of GABA‐transaminase deficiency. *Neurology* **88**: 1919–1924 https://www.ncbi.nlm.nih.gov/pubmed/28411234?dopt=AbstractPlus

Lee H *et al*. (2015) Ornithine aminotransferase versus GABA aminotransferase: implications for the design of new anticancer drugs. *Med Res Rev* **35**: 286–305 https://www.ncbi.nlm.nih.gov/pubmed/25145640?dopt=AbstractPlus

## http://www.guidetopharmacology.org/GRAC/FamilyDisplayForward?familyId=244

### Overview

Phospholipids are the basic barrier components of membranes in eukaryotic cells divided into glycerophospholipids (phosphatidic acid, phosphatidylethanolamine, phosphatidylcholine, phosphatidylserine, phosphatidylinositol and its phosphorylated derivatives) and sphingolipids (ceramide phosphorylcholine and ceramide phosphorylethanolamine).

## http://www.guidetopharmacology.org/GRAC/FamilyDisplayForward?familyId=274

### Overview

Phosphoinositide‐specific phospholipase C (PLC, EC 3.1.4.11), catalyses the hydrolysis of http://www.guidetopharmacology.org/GRAC/LigandDisplayForward?ligandId=2387 to http://www.guidetopharmacology.org/GRAC/LigandDisplayForward?ligandId=4222 and 1,2‐diacylglycerol, each of which have major second messenger functions. Two domains, X and Y, essential for catalytic activity, are conserved in the different forms of PLC. Isoforms of PLC‐ß are activated primarily by G protein‐coupled receptors through members of the G_q/11_ family of G proteins. The receptor‐mediated activation of PLC‐γ involves their phosphorylation by http://www.guidetopharmacology.org/GRAC/FamilyDisplayForward?familyId=304 in response to activation of a variety of growth factor receptors and immune system receptors. PLC‐ ∈1 may represent a point of convergence of signalling via both G protein‐coupled and catalytic receptors. Ca^2+^ ions are required for catalytic activity of PLC isoforms and have been suggested to be the major physiological form of regulation of PLC‐δ activity. PLC has been suggested to be activated non‐selectively by the small molecule http://www.guidetopharmacology.org/GRAC/LigandDisplayForward?ligandId=5097 [http://www.ncbi.nlm.nih.gov/pubmed/12695532?dopt=AbstractPlus], although this mechanism of action has been questioned [http://www.ncbi.nlm.nih.gov/pubmed/15302681?dopt=AbstractPlus]. The aminosteroid http://www.guidetopharmacology.org/GRAC/LigandDisplayForward?ligandId=5283 has been described as an inhibitor of phosphoinositide‐specific PLC [http://www.ncbi.nlm.nih.gov/pubmed/2338654?dopt=AbstractPlus], although its selectivity among the isoforms is untested and it has been reported to occupy the H1 histamine receptor [http://www.ncbi.nlm.nih.gov/pubmed/11138848?dopt=AbstractPlus].

**Table:** 

Nomenclature	http://www.guidetopharmacology.org/GRAC/ObjectDisplayForward?objectId=1403	http://www.guidetopharmacology.org/GRAC/ObjectDisplayForward?objectId=1404	http://www.guidetopharmacology.org/GRAC/ObjectDisplayForward?objectId=1405	http://www.guidetopharmacology.org/GRAC/ObjectDisplayForward?objectId=1406
HGNC, UniProt	https://www.genenames.org/data/gene‐symbol‐report/#!/hgnc_id/HGNC:15917, http://www.uniprot.org/uniprot/Q9NQ66	https://www.genenames.org/data/gene‐symbol‐report/#!/hgnc_id/HGNC:9055, http://www.uniprot.org/uniprot/Q00722	https://www.genenames.org/data/gene‐symbol‐report/#!/hgnc_id/HGNC:9056, http://www.uniprot.org/uniprot/Q01970	https://www.genenames.org/data/gene‐symbol‐report/#!/hgnc_id/HGNC:9059, http://www.uniprot.org/uniprot/Q15147
EC number	http://www.genome.jp/dbget‐bin/www_bget?ec:3.1.4.11: 1‐phosphatidyl‐1D‐*myo*‐inositol 4,5‐bisphosphate + H_2_O = 1D‐*myo*‐inositol 1,4,5‐trisphosphate + diacylglycerol			
Endogenous activators	Gαq, Gα11, Gßγ [http://www.ncbi.nlm.nih.gov/pubmed/8314796?dopt=AbstractPlus, http://www.ncbi.nlm.nih.gov/pubmed/8383116?dopt=AbstractPlus, http://www.ncbi.nlm.nih.gov/pubmed/1846707?dopt=AbstractPlus]	Gα16, Gßγ, http://www.guidetopharmacology.org/GRAC/LigandDisplayForward?ligandId=5251 (http://www.guidetopharmacology.org/GRAC/LigandDisplayForward?ligandId=5251, http://www.uniprot.org/uniprot/P15153) [http://www.ncbi.nlm.nih.gov/pubmed/1465133?dopt=AbstractPlus, http://www.ncbi.nlm.nih.gov/pubmed/12441352?dopt=AbstractPlus, http://www.ncbi.nlm.nih.gov/pubmed/12509427?dopt=AbstractPlus, http://www.ncbi.nlm.nih.gov/pubmed/1322889?dopt=AbstractPlus, http://www.ncbi.nlm.nih.gov/pubmed/8383116?dopt=AbstractPlus]	Gαq, Gßγ [http://www.ncbi.nlm.nih.gov/pubmed/8380773?dopt=AbstractPlus, http://www.ncbi.nlm.nih.gov/pubmed/1322889?dopt=AbstractPlus, http://www.ncbi.nlm.nih.gov/pubmed/8383116?dopt=AbstractPlus]	Gαq [http://www.ncbi.nlm.nih.gov/pubmed/8454637?dopt=AbstractPlus]

**Table:** 

Nomenclature	http://www.guidetopharmacology.org/GRAC/ObjectDisplayForward?objectId=1407	http://www.guidetopharmacology.org/GRAC/ObjectDisplayForward?objectId=1408	http://www.guidetopharmacology.org/GRAC/ObjectDisplayForward?objectId=1409	http://www.guidetopharmacology.org/GRAC/ObjectDisplayForward?objectId=1410	http://www.guidetopharmacology.org/GRAC/ObjectDisplayForward?objectId=1411
HGNC, UniProt	https://www.genenames.org/data/gene‐symbol‐report/#!/hgnc_id/HGNC:9065, http://www.uniprot.org/uniprot/P19174	https://www.genenames.org/data/gene‐symbol‐report/#!/hgnc_id/HGNC:9066, http://www.uniprot.org/uniprot/P16885	https://www.genenames.org/data/gene‐symbol‐report/#!/hgnc_id/HGNC:9060, http://www.uniprot.org/uniprot/P51178	https://www.genenames.org/data/gene‐symbol‐report/#!/hgnc_id/HGNC:9061, http://www.uniprot.org/uniprot/Q8N3E9	https://www.genenames.org/data/gene‐symbol‐report/#!/hgnc_id/HGNC:9062, http://www.uniprot.org/uniprot/Q9BRC7
EC number	http://www.genome.jp/dbget‐bin/www_bget?ec:3.1.4.11: 1‐phosphatidyl‐1D‐*myo*‐inositol 4,5‐bisphosphate + H_2_O = 1D‐*myo*‐inositol 1,4,5‐trisphosphate + diacylglycerol				
Endogenous activators	http://www.guidetopharmacology.org/GRAC/LigandDisplayForward?ligandId=2353 [http://www.ncbi.nlm.nih.gov/pubmed/9468499?dopt=AbstractPlus]	http://www.guidetopharmacology.org/GRAC/LigandDisplayForward?ligandId=2353, http://www.guidetopharmacology.org/GRAC/LigandDisplayForward?ligandId=5250 (http://www.guidetopharmacology.org/GRAC/LigandDisplayForward?ligandId=5250, http://www.uniprot.org/uniprot/P63000), http://www.guidetopharmacology.org/GRAC/LigandDisplayForward?ligandId=5251 (http://www.guidetopharmacology.org/GRAC/LigandDisplayForward?ligandId=5251, http://www.uniprot.org/uniprot/P15153), http://www.guidetopharmacology.org/GRAC/LigandDisplayForward?ligandId=5252 (https://www.genenames.org/data/gene‐symbol‐report/#!/hgnc_id/HGNC:9803, http://www.uniprot.org/uniprot/P60763) [http://www.ncbi.nlm.nih.gov/pubmed/9468499?dopt=AbstractPlus, http://www.ncbi.nlm.nih.gov/pubmed/16172125?dopt=AbstractPlus, http://www.ncbi.nlm.nih.gov/pubmed/18728011?dopt=AbstractPlus]	Transglutaminase II, http://www.guidetopharmacology.org/GRAC/LigandDisplayForward?ligandId=5237 {Rat}, http://www.guidetopharmacology.org/GRAC/LigandDisplayForward?ligandId=710, Gßγ [http://www.ncbi.nlm.nih.gov/pubmed/1654825?dopt=AbstractPlus, http://www.ncbi.nlm.nih.gov/pubmed/7835339?dopt=AbstractPlus, http://www.ncbi.nlm.nih.gov/pubmed/10518533?dopt=AbstractPlus, http://www.ncbi.nlm.nih.gov/pubmed/8383116?dopt=AbstractPlus]	–	–
Endogenous inhibitors	–	–	Sphingomyelin [http://www.ncbi.nlm.nih.gov/pubmed/1497353?dopt=AbstractPlus]	–	–
Inhibitors	–	http://www.guidetopharmacology.org/GRAC/LigandDisplayForward?ligandId=8820 (pIC_50_ 5.5) [http://www.ncbi.nlm.nih.gov/pubmed/19303309?dopt=AbstractPlus]	–	–	–

**Table:** 

Nomenclature	http://www.guidetopharmacology.org/GRAC/ObjectDisplayForward?objectId=1412	http://www.guidetopharmacology.org/GRAC/ObjectDisplayForward?objectId=1413	http://www.guidetopharmacology.org/GRAC/ObjectDisplayForward?objectId=1414	http://www.guidetopharmacology.org/GRAC/ObjectDisplayForward?objectId=1415
HGNC, UniProt	https://www.genenames.org/data/gene‐symbol‐report/#!/hgnc_id/HGNC:17175, http://www.uniprot.org/uniprot/Q9P212	https://www.genenames.org/data/gene‐symbol‐report/#!/hgnc_id/HGNC:19218, http://www.uniprot.org/uniprot/Q86YW0	https://www.genenames.org/data/gene‐symbol‐report/#!/hgnc_id/HGNC:29185, http://www.uniprot.org/uniprot/Q4KWH8	https://www.genenames.org/data/gene‐symbol‐report/#!/hgnc_id/HGNC:29037, http://www.uniprot.org/uniprot/O75038
EC number	http://www.genome.jp/dbget‐bin/www_bget?ec:3.1.4.11: 1‐phosphatidyl‐1D‐*myo*‐inositol 4,5‐bisphosphate + H_2_O = 1D‐myo‐inositol 1,4,5‐trisphosphate + diacylglycerol			
Endogenous activators	Ras, rho [http://www.ncbi.nlm.nih.gov/pubmed/11022048?dopt=AbstractPlus, http://www.ncbi.nlm.nih.gov/pubmed/14993441?dopt=AbstractPlus]	–	–	Gßγ [http://www.ncbi.nlm.nih.gov/pubmed/16107206?dopt=AbstractPlus]

### Comments

A series of PLC‐like proteins (https://www.genenames.org/data/gene‐symbol‐report/#!/hgnc_id/HGNC:9063, http://www.uniprot.org/uniprot/Q15111; https://www.genenames.org/data/gene‐symbol‐report/#!/hgnc_id/HGNC:9064, http://www.uniprot.org/uniprot/Q9UPR0 and https://www.genenames.org/data/gene‐symbol‐report/#!/hgnc_id/HGNC:29185, http://www.uniprot.org/uniprot/Q4KWH8) form a family with PLCδ and PLCζ1 isoforms, but appear to lack catalytic activity. PLC‐δ2 has been cloned from bovine sources [http://www.ncbi.nlm.nih.gov/pubmed/1848183?dopt=AbstractPlus].

### Further reading on Phosphoinositide‐specific phospholipase C

Cocco L *et al*. (2015) Phosphoinositide‐specific phospholipase C in health and disease. *J. Lipid Res*.**56**: 1853–60 https://www.ncbi.nlm.nih.gov/pubmed/25821234?dopt=AbstractPlus

Cockcroft S *et al*. (2016) Topological organisation of the phosphatidylinositol 4,5‐bisphosphatephospholipase C resynthesis cycle: PITPs bridge the ER‐PM gap. *Biochem. J*. **473**: 4289–4310 https://www.ncbi.nlm.nih.gov/pubmed/27888240?dopt=AbstractPlus

Litosch I. (2015) Regulating G protein activity by lipase‐independent functions of phospholipase C. *Life Sci*. **137**: 116–24 https://www.ncbi.nlm.nih.gov/pubmed/26239437?dopt=AbstractPlus

Nakamura Y *et al*. (2017) Regulation and physiological functions of mammalian phospholipase C. *J. Biochem*. **161**: 315–321 https://www.ncbi.nlm.nih.gov/pubmed/28130414?dopt=AbstractPlus

Swann K *et al*. (2016) The sperm phospholipase C‐ζ and Ca2+ signalling at fertilization in mammals. *Biochem. Soc. Trans*. **44**: 267–72 https://www.ncbi.nlm.nih.gov/pubmed/26862214?dopt=AbstractPlus

## http://www.guidetopharmacology.org/GRAC/FamilyDisplayForward?familyId=244#Phospholipase A2

### Overview

Phospholipase A_2_ (PLA_2_, EC 3.1.1.4) cleaves the *sn*‐2 fatty acid of phospholipids, primarily phosphatidylcholine, to generate http://www.guidetopharmacology.org/GRAC/LigandDisplayForward?ligandId=2508 and http://www.guidetopharmacology.org/GRAC/LigandDisplayForward?ligandId=2391. Most commonly‐used inhibitors (*e.g*. http://www.guidetopharmacology.org/GRAC/LigandDisplayForward?ligandId=5149, http://www.guidetopharmacology.org/GRAC/LigandDisplayForward?ligandId=5142 or http://www.guidetopharmacology.org/GRAC/LigandDisplayForward?ligandId=5218) are either non‐selective within the family of phospholipase A_2_ enzymes or have activity against other eicosanoid‐metabolising enzymes.

### Secreted or extracellular forms

sPLA_2_‐1B, sPLA_2_‐2A, sPLA_2_‐2D, sPLA_2_‐2E, sPLA_2_‐2F, sPLA_2_‐3, sPLA_2_‐10 and sPLA_2_‐12A

### Cytosolic, calcium‐dependent forms

cPLA_2_‐4A, cPLA_2_‐4B, cPLA_2_‐4C, cPLA_2_‐4D, cPLA_2_‐4E and cPLA_2_‐4F

### Other forms

PLA_2_‐G5, iPLA_2_‐G6, PLA_2_‐G7 and PAFAH2 (platelet‐activating factor acetylhydrolase 2)

**Table:** 

Nomenclature	http://www.guidetopharmacology.org/GRAC/ObjectDisplayForward?objectId=1416	http://www.guidetopharmacology.org/GRAC/ObjectDisplayForward?objectId=1417	http://www.guidetopharmacology.org/GRAC/ObjectDisplayForward?objectId=1418	http://www.guidetopharmacology.org/GRAC/ObjectDisplayForward?objectId=1419	http://www.guidetopharmacology.org/GRAC/ObjectDisplayForward?objectId=1420	http://www.guidetopharmacology.org/GRAC/ObjectDisplayForward?objectId=1421
HGNC, UniProt	https://www.genenames.org/data/gene‐symbol‐report/#!/hgnc_id/HGNC:9030, http://www.uniprot.org/uniprot/P04054	https://www.genenames.org/data/gene‐symbol‐report/#!/hgnc_id/HGNC:9031, http://www.uniprot.org/uniprot/P14555	https://www.genenames.org/data/gene‐symbol‐report/#!/hgnc_id/HGNC:9033, http://www.uniprot.org/uniprot/Q9UNK4	https://www.genenames.org/data/gene‐symbol‐report/#!/hgnc_id/HGNC:13414, http://www.uniprot.org/uniprot/Q9NZK7	https://www.genenames.org/data/gene‐symbol‐report/#!/hgnc_id/HGNC:30040, http://www.uniprot.org/uniprot/Q9BZM2	https://www.genenames.org/data/gene‐symbol‐report/#!/hgnc_id/HGNC:17934, http://www.uniprot.org/uniprot/Q9NZ20
EC number	http://www.genome.jp/dbget‐bin/www_bget?ec:3.1.1.4	http://www.genome.jp/dbget‐bin/www_bget?ec:3.1.1.4	http://www.genome.jp/dbget‐bin/www_bget?ec:3.1.1.4	http://www.genome.jp/dbget‐bin/www_bget?ec:3.1.1.4	http://www.genome.jp/dbget‐bin/www_bget?ec:3.1.1.4	http://www.genome.jp/dbget‐bin/www_bget?ec:3.1.1.4
Inhibitors	http://www.guidetopharmacology.org/GRAC/LigandDisplayForward?ligandId=8764 (pIC_50_ 8.9) [http://www.ncbi.nlm.nih.gov/pubmed/8809154?dopt=AbstractPlus]	–	http://www.guidetopharmacology.org/GRAC/LigandDisplayForward?ligandId=8787 (pIC_50_ 8.1) [http://www.ncbi.nlm.nih.gov/pubmed/18605714?dopt=AbstractPlus]	http://www.guidetopharmacology.org/GRAC/LigandDisplayForward?ligandId=8787 (pIC_50_ 8.1) [http://www.ncbi.nlm.nih.gov/pubmed/18605714?dopt=AbstractPlus]	http://www.guidetopharmacology.org/GRAC/LigandDisplayForward?ligandId=8787 (pIC_50_ 7.3) [http://www.ncbi.nlm.nih.gov/pubmed/18605714?dopt=AbstractPlus]	–
Comments	–	–	–	–	–	–

**Table:** 

Nomenclature	http://www.guidetopharmacology.org/GRAC/ObjectDisplayForward?objectId=1424	http://www.guidetopharmacology.org/GRAC/ObjectDisplayForward?objectId=1425	http://www.guidetopharmacology.org/GRAC/ObjectDisplayForward?objectId=1426	http://www.guidetopharmacology.org/GRAC/ObjectDisplayForward?objectId=1427	http://www.guidetopharmacology.org/GRAC/ObjectDisplayForward?objectId=1428	http://www.guidetopharmacology.org/GRAC/ObjectDisplayForward?objectId=1429
HGNC, UniProt	https://www.genenames.org/data/gene‐symbol‐report/#!/hgnc_id/HGNC:9035, http://www.uniprot.org/uniprot/P47712	https://www.genenames.org/data/gene‐symbol‐report/#!/hgnc_id/HGNC:9036, http://www.uniprot.org/uniprot/P0C869	https://www.genenames.org/data/gene‐symbol‐report/#!/hgnc_id/HGNC:9037, http://www.uniprot.org/uniprot/Q9UP65	https://www.genenames.org/data/gene‐symbol‐report/#!/hgnc_id/HGNC:30038, http://www.uniprot.org/uniprot/Q86XP0	https://www.genenames.org/data/gene‐symbol‐report/#!/hgnc_id/HGNC:24791, http://www.uniprot.org/uniprot/Q3MJ16	https://www.genenames.org/data/gene‐symbol‐report/#!/hgnc_id/HGNC:27396, http://www.uniprot.org/uniprot/Q68DD2
EC number	http://www.genome.jp/dbget‐bin/www_bget?ec:3.1.1.4	http://www.genome.jp/dbget‐bin/www_bget?ec:3.1.1.4	http://www.genome.jp/dbget‐bin/www_bget?ec:3.1.1.4	http://www.genome.jp/dbget‐bin/www_bget?ec:3.1.1.4	http://www.genome.jp/dbget‐bin/www_bget?ec:3.1.1.4	http://www.genome.jp/dbget‐bin/www_bget?ec:3.1.1.4
Inhibitors	http://www.guidetopharmacology.org/GRAC/LigandDisplayForward?ligandId=8771 (pIC_50_ 8.4) [http://www.ncbi.nlm.nih.gov/pubmed/16610804?dopt=AbstractPlus]	–	–	–	–	–
Comments	cPLA_2_‐4A also expresses lysophospholipase (http://www.genome.jp/kegg‐bin/search_brite?option=‐a&search_string=3.1.1.5) activity [http://www.ncbi.nlm.nih.gov/pubmed/8083230?dopt=AbstractPlus].	–	–	–	–	–

**Table:** 

Nomenclature	http://www.guidetopharmacology.org/GRAC/ObjectDisplayForward?objectId=1430	http://www.guidetopharmacology.org/GRAC/ObjectDisplayForward?objectId=1431	http://www.guidetopharmacology.org/GRAC/ObjectDisplayForward?objectId=1432	http://www.guidetopharmacology.org/GRAC/ObjectDisplayForward?objectId=1422	http://www.guidetopharmacology.org/GRAC/ObjectDisplayForward?objectId=1423	http://www.guidetopharmacology.org/GRAC/ObjectDisplayForward?objectId=2508
HGNC, UniProt	https://www.genenames.org/data/gene‐symbol‐report/#!/hgnc_id/HGNC:9038, http://www.uniprot.org/uniprot/P39877	https://www.genenames.org/data/gene‐symbol‐report/#!/hgnc_id/HGNC:9039, http://www.uniprot.org/uniprot/O60733	https://www.genenames.org/data/gene‐symbol‐report/#!/hgnc_id/HGNC:9040, http://www.uniprot.org/uniprot/Q13093	https://www.genenames.org/data/gene‐symbol‐report/#!/hgnc_id/HGNC:9029, http://www.uniprot.org/uniprot/O15496	https://www.genenames.org/data/gene‐symbol‐report/#!/hgnc_id/HGNC:18554, http://www.uniprot.org/uniprot/Q9BZM1	https://www.genenames.org/data/gene‐symbol‐report/#!/hgnc_id/HGNC:8579, http://www.uniprot.org/uniprot/Q99487
EC number	http://www.genome.jp/dbget‐bin/www_bget?ec:3.1.1.4	http://www.genome.jp/dbget‐bin/www_bget?ec:3.1.1.4	http://www.genome.jp/dbget‐bin/www_bget?ec:3.1.1.4	http://www.genome.jp/dbget‐bin/www_bget?ec:3.1.1.4	http://www.genome.jp/dbget‐bin/www_bget?ec:3.1.1.4	http://www.genome.jp/dbget‐bin/www_bget?ec:3.1.1.47
Inhibitors	http://www.guidetopharmacology.org/GRAC/LigandDisplayForward?ligandId=8787 (pIC_50_ 7.5) [http://www.ncbi.nlm.nih.gov/pubmed/18605714?dopt=AbstractPlus]	–	http://www.guidetopharmacology.org/GRAC/LigandDisplayForward?ligandId=6696 (pIC_50_ 10) [http://www.ncbi.nlm.nih.gov/pubmed/12643913?dopt=AbstractPlus]	http://www.guidetopharmacology.org/GRAC/LigandDisplayForward?ligandId=8787 (pIC_50_ 7.7) [http://www.ncbi.nlm.nih.gov/pubmed/18605714?dopt=AbstractPlus]	–	–
Selective inhibitors	–	–	http://www.guidetopharmacology.org/GRAC/LigandDisplayForward?ligandId=7376 (Competitive) (pIC_50_ 9.6) [http://www.ncbi.nlm.nih.gov/pubmed/19667981?dopt=AbstractPlus]	–	–	–
Comments	–	–	–	–	–	PAFAH2 also expresses PAF hydrolase activity (EC 3.1.1.47)

### Comments

The sequence of PLA_2_‐2C suggests a lack of catalytic activity, while PLA_2_‐12B (GXIIB, GXIII sPLA_2_‐like) appears to be catalytically inactive [http://www.ncbi.nlm.nih.gov/pubmed/14516201?dopt=AbstractPlus]. A further fragment has been identified with sequence similarities to Group II PLA_2_ members. Otoconin 90 (OC90) shows sequence homology to PLA_2_‐G10.

A binding protein for secretory phospholipase A_2_ has been identified which shows modest selectivity for sPLA_2_‐1B over sPLA_2_‐2A, and also binds snake toxin phospholipase A_2_ [http://www.ncbi.nlm.nih.gov/pubmed/7548076?dopt=AbstractPlus]. The binding protein appears to have clearance function for circulating secretory phospholipase A_2_, as well as signalling functions, and is a candidate antigen for idiopathic membraneous nephropathy [http://www.ncbi.nlm.nih.gov/pubmed/19571279?dopt=AbstractPlus].

PLA_2_‐G7 and PAFAH2 also express platelet‐activating factor acetylhydrolase activity (http://www.genome.jp/kegg‐bin/search_brite?option=‐a&search_string=3.1.1.47).

### Further reading on Phospholipase A_2_

Astudillo AM. (2019) Selectivity of phospholipid hydrolysis by phospholipase A2 enzymes in activated cells leading to polyunsaturated fatty acid mobilization. *Biochim Biophys Acta Mol Cell Biol Lipids* **1864**: 772–783 https://www.ncbi.nlm.nih.gov/pubmed/30010011

Kita Y *etal*. (2019) Cytosolic phospholipase A2 and lysophospholipid acyltransferases. *Biochim Biophys Acta Mol Cell Biol Lipids* **1864**: 838–845 https://www.ncbi.nlm.nih.gov/pubmed/30905348

Mouchlis VD and Dennis EA. (2019) Phospholipase A2 catalysis and lipid. mediator lipidomics. *Biochim Biophys Acta Mol Cell Biol Lipids* **1864**: 766–771 https://www.ncbi.nlm.nih.gov/pubmed/30905345

Murakami M *et al*. (2019) Group IID, IIE, IIF and III secreted phospholipase A2s. *Biochim Biophys Acta Mol Cell Biol Lipids*. **1864**: 803–818 https://www.ncbi.nlm.nih.gov/pubmed/30905347

Samuchiwal SK and Balestrieri B. (2019) Harmful and protective roles of group V phospholipase A2: Current perspectives and future directions. *Biochim Biophys Acta Mol Cell Biol Lipids* **1864**: 819–826 https://www.ncbi.nlm.nih.gov/pubmed/30308324

Shayman JA and Tesmer JJG. (2019) Lysosomal phospholipase A2. *Biochim Biophys Acta Mol Cell Biol Lipids*. **1864**: 932–940 https://www.ncbi.nlm.nih.gov/pubmed/30077006

## http://www.guidetopharmacology.org/GRAC/FamilyDisplayForward?familyId=276

### Overview

Phosphatidylcholine‐specific phospholipase D (PLD, EC 3.1.4.4) catalyses the formation of phosphatidic acid from phosphatidylcholine. In addition, the enzyme can make use of alcohols, such as butanol in a transphosphatidylation reaction [http://www.ncbi.nlm.nih.gov/pubmed/2186929?dopt=AbstractPlus].

**Table:** 

Nomenclature	http://www.guidetopharmacology.org/GRAC/ObjectDisplayForward?objectId=1433	http://www.guidetopharmacology.org/GRAC/ObjectDisplayForward?objectId=1434
HGNC, UniProt	http://www.guidetopharmacology.org/GRAC/ObjectDisplayForward?objectId=1433, http://www.uniprot.org/uniprot/Q13393	http://www.guidetopharmacology.org/GRAC/ObjectDisplayForward?objectId=1434, http://www.uniprot.org/uniprot/O14939
EC number	http://www.genome.jp/dbget‐bin/www_bget?ec:3.1.4.4	http://www.genome.jp/dbget‐bin/www_bget?ec:3.1.4.4 A phosphatidylcholine + H_2_O <=> choline + a phosphatidate
Endogenous activators	http://www.guidetopharmacology.org/GRAC/LigandDisplayForward?ligandId=5305 (https://www.genenames.org/data/gene‐symbol‐report/#!/hgnc_id/HGNC:652, http://www.uniprot.org/uniprot/P84077), http://www.guidetopharmacology.org/GRAC/LigandDisplayForward?ligandId=2387, RhoA, PKC evoked phosphorylation, RalA [http://www.ncbi.nlm.nih.gov/pubmed/9013646?dopt=AbstractPlus, http://www.ncbi.nlm.nih.gov/pubmed/9207251?dopt=AbstractPlus]	http://www.guidetopharmacology.org/GRAC/LigandDisplayForward?ligandId=5305 (https://www.genenames.org/data/gene‐symbol‐report/#!/hgnc_id/HGNC:652, http://www.uniprot.org/uniprot/P84077), http://www.guidetopharmacology.org/GRAC/LigandDisplayForward?ligandId=2387 [http://www.ncbi.nlm.nih.gov/pubmed/9582313?dopt=AbstractPlus], http://www.guidetopharmacology.org/GRAC/LigandDisplayForward?ligandId=1054 [http://www.ncbi.nlm.nih.gov/pubmed/12374567?dopt=AbstractPlus]
Endogenous inhibitors	Gßγ [http://www.ncbi.nlm.nih.gov/pubmed/16638972?dopt=AbstractPlus]	Gßγ [http://www.ncbi.nlm.nih.gov/pubmed/16638972?dopt=AbstractPlus]
Inhibitors	http://www.guidetopharmacology.org/GRAC/LigandDisplayForward?ligandId=8781 (pIC_50_ 8) [http://www.ncbi.nlm.nih.gov/pubmed/19136975?dopt=AbstractPlus]	–
Selective inhibitors	http://www.guidetopharmacology.org/GRAC/LigandDisplayForward?ligandId=8782 (pIC_50_ 7.3) [http://www.ncbi.nlm.nih.gov/pubmed/19136975?dopt=AbstractPlus]	http://www.guidetopharmacology.org/GRAC/LigandDisplayForward?ligandId=5287 (pIC_50_ 7.7) [http://www.ncbi.nlm.nih.gov/pubmed/20735042?dopt=AbstractPlus]

### Comments

A lysophospholipase D activity (https://www.genenames.org/data/gene‐symbol‐report/#!/hgnc_id/HGNC:3357, http://www.uniprot.org/uniprot/Q13822, also known as ectonucleotide pyrophosphatase/ phosphodiesterase 2, phosphodiesterase I, nucleotide pyrophosphatase 2, autotaxin) has been described, which not only catalyses the production of lysophosphatidic acid (http://www.guidetopharmacology.org/GRAC/LigandDisplayForward?ligandId=2906) from http://www.guidetopharmacology.org/GRAC/LigandDisplayForward?ligandId=2508, but also cleaves http://www.guidetopharmacology.org/GRAC/LigandDisplayForward?ligandId=1713 (see Goding *et al*., 2003 [http://www.ncbi.nlm.nih.gov/pubmed/12757929?dopt=AbstractPlus]). Additionally, an N‐acylethanolaminespecific phospholipase D (https://www.genenames.org/data/gene‐symbol‐report/#!/hgnc_id/HGNC:21683, http://www.uniprot.org/uniprot/Q6IQ20) has been characterized, which appears to have a role in the generation of http://www.guidetopharmacology.org/GRAC/FamilyDisplayForward?familyId=273/endovanilloids, including http://www.guidetopharmacology.org/GRAC/LigandDisplayForward?ligandId=2364 [http://www.ncbi.nlm.nih.gov/pubmed/14634025?dopt=AbstractPlus]. This enzyme activity appears to be enhanced by polyamines in the physiological range [http://www.ncbi.nlm.nih.gov/pubmed/12047899?dopt=AbstractPlus] and fails to transphosphatidylate with alcohols [http://www.ncbi.nlm.nih.gov/pubmed/10428468?dopt=AbstractPlus].

Three further, less well‐characterised isoforms are PLD3 (https://www.genenames.org/data/gene‐symbol‐report/#!/hgnc_id/HGNC:17158, http://www.uniprot.org/uniprot/Q8IV08, other names Choline phosphatase 3, HindIII K4L homolog, Hu‐K4), PLD4 (https://www.genenames.org/data/gene‐symbol‐report/#!/hgnc_id/HGNC:23792, http://www.uniprot.org/uniprot/Q96BZ4, other names Choline phosphatase 4, Phosphatidylcholine‐hydrolyzing phospholipase, D4C14orf175 UNQ2488/PRO5775) and PLD5 (https://www.genenames.org/data/gene‐symbol‐report/#!/hgnc_id/HGNC:26879, http://www.uniprot.org/uniprot/Q8N7P1). PLD3 has been reported to be involved in myogenesis [http://www.ncbi.nlm.nih.gov/pubmed/22428023?dopt=AbstractPlus]. PLD4 is described not to have phospholipase D catalytic activity [http://www.ncbi.nlm.nih.gov/pubmed/21085684?dopt=AbstractPlus], but has been associated with inflammatory disorders [http://www.ncbi.nlm.nih.gov/pubmed/22446963?dopt=AbstractPlus, http://www.ncbi.nlm.nih.gov/pubmed/23577190?dopt=AbstractPlus, http://www.ncbi.nlm.nih.gov/pubmed/23124809?dopt=AbstractPlus]. Sequence analysis suggests that PLD5 is catalytically inactive.

### Further reading on Phosphatidylcholine‐specific phospholipase D

Brown HA *et al*. (2017) Targeting phospholipase D in cancer, infection and neurodegenerative disorders. *Nat Rev Drug Discov* **16**: 351‐367 https://www.ncbi.nlm.nih.gov/pubmed/28209987?dopt=AbstractPlus

Frohman MA. (2015) The phospholipase D superfamily as therapeutic targets. *Trends Pharmacol. Sci*. **36**: 137‐44 https://www.ncbi.nlm.nih.gov/pubmed/25661257?dopt=AbstractPlus

Nelson RK *et al*. (2015) Physiological and pathophysiological roles for phospholipase D. *J. Lipid Res*. **56**: 2229‐37 https://www.ncbi.nlm.nih.gov/pubmed/25926691?dopt=AbstractPlus

## http://www.guidetopharmacology.org/GRAC/FamilyDisplayForward?familyId=277

### Overview

Lipid phosphate phosphatases, divided into phosphatidic acid phosphatases or lipins catalyse the dephosphorylation of phosphatidic acid (and other phosphorylated lipid derivatives) to generate inorganic phosphate and diacylglycerol. PTEN, a phosphatase and tensin homolog (BZS, MHAM, MMAC1, PTEN1, TEP1) is a phosphatidylinositol 3,4,5‐trisphosphate 3‐phosphatase which acts as a tumour suppressor by reducing cellular levels of PI 3,4,5‐P, thereby toning down activity of PDK1 and PKB. Loss‐of‐function mutations are frequently identified as somatic mutations in cancers.

**Table:** 

Nomenclature	http://www.guidetopharmacology.org/GRAC/ObjectDisplayForward?objectId=1435	http://www.guidetopharmacology.org/GRAC/ObjectDisplayForward?objectId=1436	http://www.guidetopharmacology.org/GRAC/ObjectDisplayForward?objectId=1437	http://www.guidetopharmacology.org/GRAC/ObjectDisplayForward?objectId=1438	http://www.guidetopharmacology.org/GRAC/ObjectDisplayForward?objectId=1439	http://www.guidetopharmacology.org/GRAC/ObjectDisplayForward?objectId=1440	http://www.guidetopharmacology.org/GRAC/ObjectDisplayForward?objectId=2497
Common abbreviation	–	–	–	–	–	–	PTEN
HGNC, UniProt	https://www.genenames.org/data/gene‐symbol‐report/#!/hgnc_id/HGNC:13345, http://www.uniprot.org/uniprot/Q14693	https://www.genenames.org/data/gene‐symbol‐report/#!/hgnc_id/HGNC:14450, http://www.uniprot.org/uniprot/Q92539	https://www.genenames.org/data/gene‐symbol‐report/#!/hgnc_id/HGNC:14451, http://www.uniprot.org/uniprot/Q9BQK8	https://www.genenames.org/data/gene‐symbol‐report/#!/hgnc_id/HGNC:9228, http://www.uniprot.org/uniprot/O14494	https://www.genenames.org/data/gene‐symbol‐report/#!/hgnc_id/HGNC:9229, http://www.uniprot.org/uniprot/O14495	https://www.genenames.org/data/gene‐symbol‐report/#!/hgnc_id/HGNC:9230, http://www.uniprot.org/uniprot/O43688	https://www.genenames.org/data/gene‐symbol‐report/#!/hgnc_id/HGNC:9588, http://www.uniprot.org/uniprot/P60484
EC number	http://www.genome.jp/dbget‐bin/www_bget?ec:3.1.3.4	http://www.genome.jp/dbget‐bin/www_bget?ec:3.1.3.4	http://www.genome.jp/dbget‐bin/www_bget?ec:3.1.3.4	http://www.genome.jp/dbget‐bin/www_bget?ec:3.1.3.4	http://www.genome.jp/dbget‐bin/www_bget?ec:3.1.3.4	http://www.genome.jp/dbget‐bin/www_bget?ec:3.1.3.4	http://www.genome.jp/dbget‐bin/www_bget?ec:3.1.3.67 http://www.genome.jp/dbget‐bin/www_bget?ec:3.1.3.48 http://www.genome.jp/dbget‐bin/www_bget?ec:3.1.3.16
Substrates	–	phosphatidic acid	–	–	phosphatidic acid	–	phosphatidylinositol (3,4,5)‐trisphosphate

### Further reading on Lipid phosphate phosphatases

Knafo S and Esteban JA. (2017) PTEN: Local and Global Modulation of Neuronal Function in Health and Disease. *Trends Neurosci* **40**: 83‐91 https://www.ncbi.nlm.nih.gov/pubmed/28081942

Lee YR *et al*. (2018) The functions and regulation of the PTEN tumour suppressor: new modes and prospects. *Nat Rev Mol Cell Biol* **19**: 547‐562 https://www.ncbi.nlm.nih.gov/pubmed/29858604

Yehia L *et al*. (2019) PTEN‐opathies: from biological insights to evidence‐based precision medicine. *J Clin Invest* **129**: 452‐464 https://www.ncbi.nlm.nih.gov/pubmed/30614812

## http://www.guidetopharmacology.org/GRAC/FamilyDisplayForward?familyId=781

### Overview

Phosphatidylinositol may be phosphorylated at either 3‐ or 4‐ positions on the inositol ring by PI 3‐kinases or PI 4‐kinases, respectively.

### Phosphatidylinositol 3‐kinases

Phosphatidylinositol 3‐kinases (PI3K, provisional nomenclature) catalyse the introduction of a phosphate into the 3‐position of phosphatidylinositol (PI), phosphatidylinositol 4‐phosphate (PIP) or phosphatidylinositol 4,5‐bisphosphate (PIP_2_). There is evidence that PI3K can also phosphorylate serine/threonine residues on proteins. In addition to the classes described below, further serine/threonine protein kinases, including https://www.genenames.org/data/gene‐symbol‐report/#!/hgnc_id/HGNC:795 (http://www.uniprot.org/uniprot/Q13315) and https://www.genenames.org/data/gene‐symbol‐report/#!/hgnc_id/HGNC:3942 (http://www.uniprot.org/uniprot/P42345), have been described to phosphorylate phosphatidylinositol and have been termed PI3Krelated kinases. Structurally, PI3Ks have common motifs of at least one C2, calcium‐binding domain and helical domains, alongside structurally‐conserved catalytic domains. http://www.guidetopharmacology.org/GRAC/LigandDisplayForward?ligandId=6060 and http://www.guidetopharmacology.org/GRAC/LigandDisplayForward?ligandId=6004 are widely‐used inhibitors of PI3K activities. http://www.guidetopharmacology.org/GRAC/LigandDisplayForward?ligandId=6060 is irreversible and shows modest selectivity between Class I and Class II PI3K, while LY294002 is reversible and selective for Class I compared to Class II PI3K.

**Class I PI3Ks** (EC 2.7.1.153) phosphorylate phosphatidylinositol 4,5‐bisphosphate to generate phosphatidylinositol 3,4,5‐trisphosphate and are heterodimeric, matching catalytic and regulatory subunits. Class IA PI3Ks include p110α, p110ß and p110δ catalytic subunits, with predominantly p85 and p55 regulatory subunits. The single catalytic subunit that forms Class IB PI3K is p110γ. Class IA PI3Ks are more associated with receptor tyrosine kinase pathways, while the Class IB PI3K is linkedmore with GPCR signalling.

**Class II PI3Ks** (EC 2.7.1.154) phosphorylate phosphatidylinositol to generate phosphatidylinositol 3‐phosphate (and possibly phosphatidylinositol 4‐phosphate to generate phosphatidylinositol 3,4‐bisphosphate). Three monomeric members exist, PI3KC2α, ß and ß, and include Ras‐binding, Phox homology and two C2domains.

The only **class III PI3K** isoform (EC 2.7.1.137) is a heterodimer formed of a catalytic subunit (VPS34) and regulatory subunit (VPS15).

**Phosphatidylinositol 4‐kinases**

Phosphatidylinositol 4‐kinases (EC 2.7.1.67) generate phosphatidylinositol 4‐phosphate and may be divided into higher molecular weight type III and lower molecular weight type II forms.

## http://www.guidetopharmacology.org/GRAC/FamilyDisplayForward?familyId=638

**Table:** 

Nomenclature	http://www.guidetopharmacology.org/GRAC/ObjectDisplayForward?objectId=2148	http://www.guidetopharmacology.org/GRAC/ObjectDisplayForward?objectId=2149
Common abbreviation	PI4KIIIα/PIK4CA	PI4KIIIß/PIK4CB
HGNC, UniProt	https://www.genenames.org/data/gene‐symbol‐report/#!/hgnc_id/HGNC:8983, http://www.uniprot.org/uniprot/P42356	https://www.genenames.org/data/gene‐symbol‐report/#!/hgnc_id/HGNC:8984, http://www.uniprot.org/uniprot/Q9UBF8
EC number	http://www.genome.jp/dbget‐bin/www_bget?ec:2.7.1.67	http://www.genome.jp/dbget‐bin/www_bget?ec:2.7.1.67
Endogenous activation	–	PKD‐mediated phosphorylation [http://www.ncbi.nlm.nih.gov/pubmed/16100512?dopt=AbstractPlus]
Sub/family‐selective inhibitors	http://www.guidetopharmacology.org/GRAC/LigandDisplayForward?ligandId=6060 (pIC_50_ 6.7–6.8) [http://www.ncbi.nlm.nih.gov/pubmed/10101268?dopt=AbstractPlus, http://www.ncbi.nlm.nih.gov/pubmed/9020160?dopt=AbstractPlus]	http://www.guidetopharmacology.org/GRAC/LigandDisplayForward?ligandId=6060 (pIC_50_ 6.7–6.8) [http://www.ncbi.nlm.nih.gov/pubmed/10101268?dopt=AbstractPlus, http://www.ncbi.nlm.nih.gov/pubmed/9020160?dopt=AbstractPlus]
Selective inhibitors	–	http://www.guidetopharmacology.org/GRAC/LigandDisplayForward?ligandId=6062 (pIC_50_ 7.7) [http://www.ncbi.nlm.nih.gov/pubmed/18077555?dopt=AbstractPlus, http://www.ncbi.nlm.nih.gov/pubmed/16647110?dopt=AbstractPlus]

## http://www.guidetopharmacology.org/GRAC/FamilyDisplayForward?familyId=671

**Table:** 

Nomenclature	http://www.guidetopharmacology.org/GRAC/ObjectDisplayForward?objectId=2150	http://www.guidetopharmacology.org/GRAC/ObjectDisplayForward?objectId=2151	http://www.guidetopharmacology.org/GRAC/ObjectDisplayForward?objectId=2288
Common abbreviation	C2α/PIK3C2A	C2ß/PIK3C2B	C2γ/PIK3C2G
HGNC, UniProt	https://www.genenames.org/data/gene‐symbol‐report/#!/hgnc_id/HGNC:8971, http://www.uniprot.org/uniprot/O00443	https://www.genenames.org/data/gene‐symbol‐report/#!/hgnc_id/HGNC:8972, http://www.uniprot.org/uniprot/O00750	https://www.genenames.org/data/gene‐symbol‐report/#!/hgnc_id/HGNC:8973, http://www.uniprot.org/uniprot/O75747
EC number	http://www.genome.jp/dbget‐bin/www_bget?ec:2.7.1.154	http://www.genome.jp/dbget‐bin/www_bget?ec:2.7.1.154	http://www.genome.jp/dbget‐bin/www_bget?ec:2.7.1.154
Inhibitors	http://www.guidetopharmacology.org/GRAC/LigandDisplayForward?ligandId=8839 (pIC_50_ 7.6) [http://www.ncbi.nlm.nih.gov/pubmed/21322566?dopt=AbstractPlus]	http://www.guidetopharmacology.org/GRAC/LigandDisplayForward?ligandId=5701 (pIC_50_ 8) [http://www.ncbi.nlm.nih.gov/pubmed/17601739?dopt=AbstractPlus]	–

## http://www.guidetopharmacology.org/GRAC/FamilyDisplayForward?familyId=672

**Table:** 

Nomenclature	http://www.guidetopharmacology.org/GRAC/ObjectDisplayForward?objectId=2152
Common abbreviation	VPS34
HGNC, UniProt	https://www.genenames.org/data/gene‐symbol‐report/#!/hgnc_id/HGNC:8974, http://www.uniprot.org/uniprot/Q8NEB9
EC number	http://www.genome.jp/dbget‐bin/www_bget?ec:2.7.1.137

## http://www.guidetopharmacology.org/GRAC/FamilyDisplayForward?familyId=673

### Overview

PI3K activation is one of the most important signal transduction pathways used to transmit signals from cell‐surface receptors to regulate intracellular processes (cell growth, survival, proliferation and movement). PI3K catalytic (and regulatory) subunits play vital roles in normal cell function and in disease. Progress made in developing PI3K‐targeted agents as potential therapeutics for treating cancer and other diseases is reviewed by Fruman *et al*. (2017) [http://www.ncbi.nlm.nih.gov/pubmed/28802037?dopt=AbstractPlus].

**Table:** 

Nomenclature	http://www.guidetopharmacology.org/GRAC/ObjectDisplayForward?objectId=2153	http://www.guidetopharmacology.org/GRAC/ObjectDisplayForward?objectId=2154
Common abbreviation	PI3Kα	PI3Kß
HGNC, UniProt	https://www.genenames.org/data/gene‐symbol‐report/#!/hgnc_id/HGNC:8975, http://www.uniprot.org/uniprot/P42336	https://www.genenames.org/data/gene‐symbol‐report/#!/hgnc_id/HGNC:8976, http://www.uniprot.org/uniprot/P42338
EC number	http://www.genome.jp/dbget‐bin/www_bget?ec:2.7.1.153 http://www.genome.jp/dbget‐bin/www_bget?ec:2.7.11.1	http://www.genome.jp/dbget‐bin/www_bget?ec:2.7.1.153
Inhibitors	http://www.guidetopharmacology.org/GRAC/LigandDisplayForward?ligandId=8012 (pIC_50_ 9.5) [http://www.ncbi.nlm.nih.gov/pubmed/17601739?dopt=AbstractPlus], http://www.guidetopharmacology.org/GRAC/LigandDisplayForward?ligandId=7940 (pIC_50_ 9.4) [http://www.ncbi.nlm.nih.gov/pubmed/20166697?dopt=AbstractPlus], http://www.guidetopharmacology.org/GRAC/LigandDisplayForward?ligandId=7936 (p*K*_i_ 9.2) [http://www.ncbi.nlm.nih.gov/pubmed/24900269?dopt=AbstractPlus], http://www.guidetopharmacology.org/GRAC/LigandDisplayForward?ligandId=5701 (pIC_50_ 8.7) [http://www.ncbi.nlm.nih.gov/pubmed/19584227?dopt=AbstractPlus], http://www.guidetopharmacology.org/GRAC/LigandDisplayForward?ligandId=7951 (pIC_50_ 8.4) [http://www.ncbi.nlm.nih.gov/pubmed/22357447?dopt=AbstractPlus], http://www.guidetopharmacology.org/GRAC/LigandDisplayForward?ligandId=8011 (pIC_50_ 8.4) [http://www.ncbi.nlm.nih.gov/pubmed/23855836?dopt=AbstractPlus], http://www.guidetopharmacology.org/GRAC/LigandDisplayForward?ligandId=7950 (pIC_50_ 8.4) [http://www.ncbi.nlm.nih.gov/pubmed/18606717?dopt=AbstractPlus], http://www.guidetopharmacology.org/GRAC/LigandDisplayForward?ligandId=7888 (pIC_50_ 8.3) [http://www.ncbi.nlm.nih.gov/pubmed/21981714?dopt=AbstractPlus], http://www.guidetopharmacology.org/GRAC/LigandDisplayForward?ligandId=8012 (pIC_50_ 8.2) [http://www.ncbi.nlm.nih.gov/pubmed/16647110?dopt=AbstractPlus]	http://www.guidetopharmacology.org/GRAC/LigandDisplayForward?ligandId=8011 (pIC_50_ 9.3) [http://www.ncbi.nlm.nih.gov/pubmed/23855836?dopt=AbstractPlus], http://www.guidetopharmacology.org/GRAC/LigandDisplayForward?ligandId=5701 (pIC_50_ 8.5) [http://www.ncbi.nlm.nih.gov/pubmed/19584227?dopt=AbstractPlus], http://www.guidetopharmacology.org/GRAC/LigandDisplayForward?ligandId=8059 (pIC_50_ 8) [http://www.ncbi.nlm.nih.gov/pubmed/22906130?dopt=AbstractPlus], http://www.guidetopharmacology.org/GRAC/LigandDisplayForward?ligandId=7965 (pIC_50_ 7.4–7.8) [665, http://www.ncbi.nlm.nih.gov/pubmed/16622124?dopt=AbstractPlus], http://www.guidetopharmacology.org/GRAC/LigandDisplayForward?ligandId=7888 (pIC_50_ 7.6) [http://www.ncbi.nlm.nih.gov/pubmed/21981714?dopt=AbstractPlus], http://www.guidetopharmacology.org/GRAC/LigandDisplayForward?ligandId=7951 (pIC_50_ 7.2) [http://www.ncbi.nlm.nih.gov/pubmed/22357447?dopt=AbstractPlus]
Sub/family‐selective inhibitors	http://www.guidetopharmacology.org/GRAC/LigandDisplayForward?ligandId=5682 (pIC_50_ 8.5) [http://www.ncbi.nlm.nih.gov/pubmed/18754654?dopt=AbstractPlus]	http://www.guidetopharmacology.org/GRAC/LigandDisplayForward?ligandId=5682 (pIC_50_ 7.5) [http://www.ncbi.nlm.nih.gov/pubmed/18754654?dopt=AbstractPlus]
Selective inhibitors	http://www.guidetopharmacology.org/GRAC/LigandDisplayForward?ligandId=9826 (pIC_50_ 8.7) [http://www.ncbi.nlm.nih.gov/pubmed/24900173?dopt=AbstractPlus]	–

**Table:** 

Nomenclature	http://www.guidetopharmacology.org/GRAC/ObjectDisplayForward?objectId=2156	http://www.guidetopharmacology.org/GRAC/ObjectDisplayForward?objectId=2155
Common abbreviation	PI3Kγ	PI3Kδ
HGNC, UniProt	https://www.genenames.org/data/gene‐symbol‐report/#!/hgnc_id/HGNC:8978, http://www.uniprot.org/uniprot/P48736	https://www.genenames.org/data/gene‐symbol‐report/#!/hgnc_id/HGNC:8977, http://www.uniprot.org/uniprot/O00329
EC number	http://www.genome.jp/dbget‐bin/www_bget?ec:2.7.1.153	http://www.genome.jp/dbget‐bin/www_bget?ec:2.7.1.153
Inhibitors	http://www.guidetopharmacology.org/GRAC/LigandDisplayForward?ligandId=7950 (pIC_50_ 8.3) [http://www.ncbi.nlm.nih.gov/pubmed/18606717?dopt=AbstractPlus], http://www.guidetopharmacology.org/GRAC/LigandDisplayForward?ligandId=7888 (pIC_50_ 7.8) [http://www.ncbi.nlm.nih.gov/pubmed/21981714?dopt=AbstractPlus], http://www.guidetopharmacology.org/GRAC/LigandDisplayForward?ligandId=5701 (pIC_50_ 7.8) [http://www.ncbi.nlm.nih.gov/pubmed/19584227?dopt=AbstractPlus], http://www.guidetopharmacology.org/GRAC/LigandDisplayForward?ligandId=7951 (pIC_50_ 7.4) [http://www.ncbi.nlm.nih.gov/pubmed/22357447?dopt=AbstractPlus], http://www.guidetopharmacology.org/GRAC/LigandDisplayForward?ligandId=7965 (pIC_50_ 7.3–7.3) [665, http://www.ncbi.nlm.nih.gov/pubmed/16622124?dopt=AbstractPlus], http://www.guidetopharmacology.org/GRAC/LigandDisplayForward?ligandId=5715 (pIC_50_ 7.1) [http://www.ncbi.nlm.nih.gov/pubmed/17685602?dopt=AbstractPlus], http://www.guidetopharmacology.org/GRAC/LigandDisplayForward?ligandId=7955 (pIC_50_ 6.6) [http://www.ncbi.nlm.nih.gov/pubmed/23726034?dopt=AbstractPlus], http://www.guidetopharmacology.org/GRAC/LigandDisplayForward?ligandId=8011 (pIC_50_ 6.2) [http://www.ncbi.nlm.nih.gov/pubmed/23855836?dopt=AbstractPlus]	http://www.guidetopharmacology.org/GRAC/LigandDisplayForward?ligandId=8011 (pIC_50_ > 10) [http://www.ncbi.nlm.nih.gov/pubmed/23855836?dopt=AbstractPlus], http://www.guidetopharmacology.org/GRAC/LigandDisplayForward?ligandId=6741 (*in vitro* activity against recombinant enzyme) (pIC_50_ 8.6) [http://www.ncbi.nlm.nih.gov/pubmed/20959606?dopt=AbstractPlus], http://www.guidetopharmacology.org/GRAC/LigandDisplayForward?ligandId=5701 (pIC_50_ 8.5) [http://www.ncbi.nlm.nih.gov/pubmed/19584227?dopt=AbstractPlus], http://www.guidetopharmacology.org/GRAC/LigandDisplayForward?ligandId=7965 (pIC_50_ 8.2–8.3) [665, http://www.ncbi.nlm.nih.gov/pubmed/16622124?dopt=AbstractPlus], http://www.guidetopharmacology.org/GRAC/LigandDisplayForward?ligandId=7888 (pIC_50_ 8.2) [http://www.ncbi.nlm.nih.gov/pubmed/21981714?dopt=AbstractPlus], http://www.guidetopharmacology.org/GRAC/LigandDisplayForward?ligandId=7950 (pIC_50_ 8.1) [http://www.ncbi.nlm.nih.gov/pubmed/18606717?dopt=AbstractPlus], http://www.guidetopharmacology.org/GRAC/LigandDisplayForward?ligandId=7955 (pIC_50_ 6.5) [http://www.ncbi.nlm.nih.gov/pubmed/23726034?dopt=AbstractPlus]
Sub/family‐selective inhibitors	http://www.guidetopharmacology.org/GRAC/LigandDisplayForward?ligandId=5682 (pIC_50_ 7.1) [http://www.ncbi.nlm.nih.gov/pubmed/18754654?dopt=AbstractPlus]	http://www.guidetopharmacology.org/GRAC/LigandDisplayForward?ligandId=5682 (pIC_50_ 8.5) [http://www.ncbi.nlm.nih.gov/pubmed/18754654?dopt=AbstractPlus]
Selective inhibitors	http://www.guidetopharmacology.org/GRAC/LigandDisplayForward?ligandId=6653 (pIC_50_ 7.6) [http://www.ncbi.nlm.nih.gov/pubmed/22544264?dopt=AbstractPlus]	–

## http://www.guidetopharmacology.org/GRAC/FamilyDisplayForward?familyId=913

**Table:** 

Nomenclature	http://www.guidetopharmacology.org/GRAC/ObjectDisplayForward?objectId=2857
HGNC, UniProt	https://www.genenames.org/data/gene‐symbol‐report/#!/hgnc_id/HGNC:23785, http://www.uniprot.org/uniprot/Q9Y2I7
EC number	http://www.genome.jp/dbget‐bin/www_bget?ec:2.7.1.150: ATP + 1‐phosphatidyl‐1D‐myo‐inositol 3‐phosphate = ADP + 1‐phosphatidyl‐1D‐myo‐inositol 3,5‐bisphosphate

## http://www.guidetopharmacology.org/GRAC/FamilyDisplayForward?familyId=675

### Overview

Type I PIP kinases are required for the production of the second messenger phosphatidylinositol 4,5‐bisphosphate (PtdIns(4,5)P2) by phosphorylating PtdIns(4)P [http://www.ncbi.nlm.nih.gov/pubmed/9367159?dopt=AbstractPlus]. This enzyme family is also known as type I PIP(5)Ks.

**Table:** 

Nomenclature	http://www.guidetopharmacology.org/GRAC/ObjectDisplayForward?objectId=2164	http://www.guidetopharmacology.org/GRAC/ObjectDisplayForward?objectId=2165
Common abbreviation	PIP5K1A	PIP5K1C
HGNC, UniProt	https://www.genenames.org/data/gene‐symbol‐report/#!/hgnc_id/HGNC:8994, http://www.uniprot.org/uniprot/Q99755	https://www.genenames.org/data/gene‐symbol‐report/#!/hgnc_id/HGNC:8996, http://www.uniprot.org/uniprot/O60331
EC number	http://www.genome.jp/dbget‐bin/www_bget?ec:2.7.1.68	http://www.genome.jp/dbget‐bin/www_bget?ec:2.7.1.68
Inhibitors	http://www.guidetopharmacology.org/GRAC/LigandDisplayForward?ligandId=8444 [http://www.ncbi.nlm.nih.gov/pubmed/25071204?dopt=AbstractPlus]	–

## http://www.guidetopharmacology.org/GRAC/FamilyDisplayForward?familyId=674

### Overview

Type II PIP kinases are essential for the production of the second messenger phosphatidylinositol 4,5‐bisphosphate (PtdIns(4,5)P2) by phosphorylating PtdIns(5)P [http://www.ncbi.nlm.nih.gov/pubmed/9367159?dopt=AbstractPlus]. This enzyme family is also known as type II PIP(5)Ks.

**Table:** 

Nomenclature	http://www.guidetopharmacology.org/GRAC/ObjectDisplayForward?objectId=2858	http://www.guidetopharmacology.org/GRAC/ObjectDisplayForward?objectId=2162	http://www.guidetopharmacology.org/GRAC/ObjectDisplayForward?objectId=2163
Common abbreviation	PIP4K2A	PIP4K2B	PIP4K2C
HGNC, UniProt	https://www.genenames.org/data/gene‐symbol‐report/#!/hgnc_id/HGNC:8997, http://www.uniprot.org/uniprot/P48426	https://www.genenames.org/data/gene‐symbol‐report/#!/hgnc_id/HGNC:8998, http://www.uniprot.org/uniprot/P78356	https://www.genenames.org/data/gene‐symbol‐report/#!/hgnc_id/HGNC:23786, http://www.uniprot.org/uniprot/Q8TBX8
EC number	http://www.genome.jp/dbget‐bin/www_bget?ec:2.7.1.149 ATP + 1‐phosphatidyl‐1D‐*myo*‐inositol 5‐phosphate <=> ADP + 1‐phosphatidyl‐1D‐*myo*‐inositol 4,5‐bisphosphate	http://www.genome.jp/dbget‐bin/www_bget?ec:2.7.1.149	http://www.genome.jp/dbget‐bin/www_bget?ec:2.7.1.149

## http://www.guidetopharmacology.org/GRAC/FamilyDisplayForward?familyId=787

### Overview

SPHK1 and SPHK2 are encoded by different genes with some redundancy of function; genetic deletion of both Sphk1 and Sphk2, but not either alone, is embryonic lethal in mice. There are splice variants of each isoform (SphK1a‐c and SphK2a, b), distinguished by their N‐terminal sequences. SPHK1 and SPHK2 differ in tissue distribution, sub‐cellular localisation, biochemical properties and regulation. They regulate discrete pools of S1P. Receptor stimulation induces SPHK1 translocation from the cytoplasm to the plasma membrane. SPHK1 translocation is regulated by phosphorylation/dephosphorylation, specific protein:protein interactions and interaction with specific lipids at the plasma membrane. SPHK1 is a dimeric protein, as confirmed by its crystal structure which forms a positive cluster, between protomers, essential for interaction with anionic phospholipids in the plasma membrane. SPHK2 is localised to the ER or associated with mitochondria or shuttles in/out of the nucleus, regulated by phosphorylation. Intracellular targets of nuclear S1P include the catalytic subunit of telomerase (TERT) and regulators of gene expression including histone deacetylases (HDAC 1/2) and peroxisome proliferator‐activated receptor gamma (PPARγ). SPHK2 phosphorylates the pro‐drug FTY720 (http://www.guidetopharmacology.org/GRAC/LigandDisplayForward?ligandId=2407, which is used to treat some forms of multiple sclerosis) to a mimic of S1P and that acts as a functional antagonist of S1P_1_ receptors. Inhibitors of SPHK1 and SPHK2 have therapeutic potential in many diseases.

**Table:** 

Nomenclature	http://www.guidetopharmacology.org/GRAC/ObjectDisplayForward?objectId=2204	http://www.guidetopharmacology.org/GRAC/ObjectDisplayForward?objectId=2205
Common abbreviation	SPHK1	SPHK2
HGNC, UniProt	https://www.genenames.org/data/gene‐symbol‐report/#!/hgnc_id/HGNC:11240, http://www.uniprot.org/uniprot/Q9NYA1	https://www.genenames.org/data/gene‐symbol‐report/#!/hgnc_id/HGNC:18859, http://www.uniprot.org/uniprot/Q9NRA0
EC number	http://www.genome.jp/dbget‐bin/www_bget?ec:2.7.1.91: http://www.guidetopharmacology.org/GRAC/LigandDisplayForward?ligandId=2452 + http://www.guidetopharmacology.org/GRAC/LigandDisplayForward?ligandId=1713 = http://www.guidetopharmacology.org/GRAC/LigandDisplayForward?ligandId=911 + http://www.guidetopharmacology.org/GRAC/LigandDisplayForward?ligandId=1712 dihydrosphingosine + http://www.guidetopharmacology.org/GRAC/LigandDisplayForward?ligandId=1713 = http://www.guidetopharmacology.org/GRAC/LigandDisplayForward?ligandId=911 + http://www.guidetopharmacology.org/GRAC/LigandDisplayForward?ligandId=1712	http://www.genome.jp/dbget‐bin/www_bget?ec:2.7.1.91: http://www.guidetopharmacology.org/GRAC/LigandDisplayForward?ligandId=2452 + http://www.guidetopharmacology.org/GRAC/LigandDisplayForward?ligandId=1713 = http://www.guidetopharmacology.org/GRAC/LigandDisplayForward?ligandId=911 + http://www.guidetopharmacology.org/GRAC/LigandDisplayForward?ligandId=1712 dihydrosphingosine + http://www.guidetopharmacology.org/GRAC/LigandDisplayForward?ligandId=1713 = http://www.guidetopharmacology.org/GRAC/LigandDisplayForward?ligandId=911 + http://www.guidetopharmacology.org/GRAC/LigandDisplayForward?ligandId=1712
Cofactors	http://www.guidetopharmacology.org/GRAC/LigandDisplayForward?ligandId=708 [http://www.ncbi.nlm.nih.gov/pubmed/22677141?dopt=AbstractPlus]	http://www.guidetopharmacology.org/GRAC/LigandDisplayForward?ligandId=708
Inhibitors	http://www.guidetopharmacology.org/GRAC/LigandDisplayForward?ligandId=6041 (p*K*_i_ 4.8) [http://www.ncbi.nlm.nih.gov/pubmed/20061445?dopt=AbstractPlus], http://www.guidetopharmacology.org/GRAC/LigandDisplayForward?ligandId=10219 (pIC_50_ 4.6) [http://www.ncbi.nlm.nih.gov/pubmed/25788259?dopt=AbstractPlus]	http://www.guidetopharmacology.org/GRAC/LigandDisplayForward?ligandId=10219 (p*K*_i_ 5.2) [http://www.ncbi.nlm.nih.gov/pubmed/25788259?dopt=AbstractPlus], http://www.guidetopharmacology.org/GRAC/LigandDisplayForward?ligandId=6041 (p*K*_i_ 5.1) [http://www.ncbi.nlm.nih.gov/pubmed/22970244?dopt=AbstractPlus]
Selective inhibitors	http://www.guidetopharmacology.org/GRAC/LigandDisplayForward?ligandId=6623 (p*K*_i_ 8.4) [http://www.ncbi.nlm.nih.gov/pubmed/22397330?dopt=AbstractPlus]	http://www.guidetopharmacology.org/GRAC/LigandDisplayForward?ligandId=10217 (p*K*_i_ 7.1) [http://www.ncbi.nlm.nih.gov/pubmed/28406646?dopt=AbstractPlus], http://www.guidetopharmacology.org/GRAC/LigandDisplayForward?ligandId=10218 (pIC_50_ 6.8) [http://www.ncbi.nlm.nih.gov/pubmed/28231433?dopt=AbstractPlus], http://www.guidetopharmacology.org/GRAC/LigandDisplayForward?ligandId=6624 (p*K*_i_ 5) [http://www.ncbi.nlm.nih.gov/pubmed/20061445?dopt=AbstractPlus], http://www.guidetopharmacology.org/GRAC/LigandDisplayForward?ligandId=6625 (p*K*_i_ 4.8) [http://www.ncbi.nlm.nih.gov/pubmed/21620961?dopt=AbstractPlus]
Comments	SK1 inhibitors induce its proteasomal degradation [http://www.ncbi.nlm.nih.gov/pubmed/20926375?dopt=AbstractPlus, http://www.ncbi.nlm.nih.gov/pubmed/26934645?dopt=AbstractPlus]. SK1 crystal structures confirm that it is dimeric [http://www.ncbi.nlm.nih.gov/pubmed/27021309?dopt=AbstractPlus]; there is no crystal structure available for SK2.	There is no crystal structure available for SK2.

### Comments

http://www.guidetopharmacology.org/GRAC/LigandDisplayForward?ligandId=10219 is competitive with ATP; other SPHK inhibitors are competitive with sphingosine. ABC294640 (http://www.guidetopharmacology.org/GRAC/LigandDisplayForward?ligandId=6624) has known off‐target effects on dihydroceramide desaturase (*DEGS1*) [http://www.ncbi.nlm.nih.gov/pubmed/26934645?dopt=AbstractPlus, http://www.ncbi.nlm.nih.gov/pubmed/26494858?dopt=AbstractPlus]) and induces proteasomal degradation of SK1 [http://www.ncbi.nlm.nih.gov/pubmed/26934645?dopt=AbstractPlus]. ABC294640 is in clinical trials for advanced cholangiocarcinoma, advanced hepatocellular carcinoma and refractory/relapsed multiple myeloma (to view ClinicalTrials.gov list click https://clinicaltrials.gov/ct2/results?cond=&term=ABC294640).

### Further reading on Sphingosine kinase

Adams DR *et al*. (2016) Sphingosine Kinases: Emerging Structure‐Function Insights. *Trends Biochem. Sci*. **41**: 395‐409 https://www.ncbi.nlm.nih.gov/pubmed/27021309?dopt=AbstractPlus

Lynch KR *et al*. (2016) Sphingosine kinase inhibitors: a review of patent literature (2006‐2015). *Expert Opin Ther Pat* **26**: 1409‐1416 https://www.ncbi.nlm.nih.gov/pubmed/27539678?dopt=AbstractPlus

Pitman MR *et al*. (2016) Recent advances in the development of sphingosine kinase inhibitors. *Cell. Signal*. **28**: 1349‐63 https://www.ncbi.nlm.nih.gov/pubmed/27297359?dopt=AbstractPlus

Pulkoski‐Gross MJ *et al*. (2018) An intrinsic lipid‐binding interface controls sphingosine kinase 1 function. *J. Lipid Res*. **59**: 462‐474 https://www.ncbi.nlm.nih.gov/pubmed/29326159?dopt=AbstractPlus

Pyne NJ *et al*. (2017) Sphingosine Kinase 2 in Autoimmune/Inflammatory Disease and the Development of Sphingosine Kinase 2 Inhibitors. *Trends Pharmacol. Sci*. **38**: 581‐591 https://www.ncbi.nlm.nih.gov/pubmed/28606480?dopt=AbstractPlus

Pyne S *et al*. (2018) Sphingosine Kinases as Druggable Targets. *Handb Exp Pharmacol* https://www.ncbi.nlm.nih.gov/pubmed/29460151?dopt=AbstractPlus

**Table:** 

Nomenclature	http://www.guidetopharmacology.org/GRAC/ObjectDisplayForward?objectId=2156	http://www.guidetopharmacology.org/GRAC/ObjectDisplayForward?objectId=2499	http://www.guidetopharmacology.org/GRAC/ObjectDisplayForward?objectId=2155	http://www.guidetopharmacology.org/GRAC/ObjectDisplayForward?objectId=2150	http://www.guidetopharmacology.org/GRAC/ObjectDisplayForward?objectId=2151	http://www.guidetopharmacology.org/GRAC/ObjectDisplayForward?objectId=2288	http://www.guidetopharmacology.org/GRAC/ObjectDisplayForward?objectId=2152
Common abbreviation	PI3Kγ	PI4KIIß/PI4K2B	PI3Kδ	C2α/PIK3C2A	C2ß/PIK3C2B	C2γ/PIK3C2G	VPS34
HGNC, UniProt	https://www.genenames.org/data/gene‐symbol‐report/#!/hgnc_id/HGNC:8978, http://www.uniprot.org/uniprot/P48736	https://www.genenames.org/data/gene‐symbol‐report/#!/hgnc_id/HGNC:18215, http://www.uniprot.org/uniprot/Q8TCG2	https://www.genenames.org/data/gene‐symbol‐report/#!/hgnc_id/HGNC:8977, http://www.uniprot.org/uniprot/O00329	https://www.genenames.org/data/gene‐symbol‐report/#!/hgnc_id/HGNC:8971, http://www.uniprot.org/uniprot/O00443	https://www.genenames.org/data/gene‐symbol‐report/#!/hgnc_id/HGNC:8972, http://www.uniprot.org/uniprot/O00750	https://www.genenames.org/data/gene‐symbol‐report/#!/hgnc_id/HGNC:8973, http://www.uniprot.org/uniprot/O75747	https://www.genenames.org/data/gene‐symbol‐report/#!/hgnc_id/HGNC:8974, http://www.uniprot.org/uniprot/Q8NEB9
EC number	http://www.genome.jp/dbget‐bin/www_bget?ec:2.7.1.153	http://www.genome.jp/dbget‐bin/www_bget?ec:2.7.1.67	http://www.genome.jp/dbget‐bin/www_bget?ec:2.7.1.153	http://www.genome.jp/dbget‐bin/www_bget?ec:2.7.1.154	http://www.genome.jp/dbget‐bin/www_bget?ec:2.7.1.154	http://www.genome.jp/dbget‐bin/www_bget?ec:2.7.1.154	http://www.genome.jp/dbget‐bin/www_bget?ec:2.7.1.137
Inhibitors	http://www.guidetopharmacology.org/GRAC/LigandDisplayForward?ligandId=7950 (pIC_50_ 8.3) [http://www.ncbi.nlm.nih.gov/pubmed/18606717?dopt=AbstractPlus], http://www.guidetopharmacology.org/GRAC/LigandDisplayForward?ligandId=7888 (pIC_50_ 7.8) [http://www.ncbi.nlm.nih.gov/pubmed/21981714?dopt=AbstractPlus], http://www.guidetopharmacology.org/GRAC/LigandDisplayForward?ligandId=5701 (pIC_50_ 7.8) [http://www.ncbi.nlm.nih.gov/pubmed/19584227?dopt=AbstractPlus], http://www.guidetopharmacology.org/GRAC/LigandDisplayForward?ligandId=7951 (pIC_50_ 7.4) [http://www.ncbi.nlm.nih.gov/pubmed/22357447?dopt=AbstractPlus], http://www.guidetopharmacology.org/GRAC/LigandDisplayForward?ligandId=7965 (pIC_50_ 7.3–7.3) [651, http://www.ncbi.nlm.nih.gov/pubmed/16622124?dopt=AbstractPlus], http://www.guidetopharmacology.org/GRAC/LigandDisplayForward?ligandId=5715 (pIC_50_ 7.1) [http://www.ncbi.nlm.nih.gov/pubmed/17685602?dopt=AbstractPlus], http://www.guidetopharmacology.org/GRAC/LigandDisplayForward?ligandId=7955 (pIC_50_ 6.6) [http://www.ncbi.nlm.nih.gov/pubmed/23726034?dopt=AbstractPlus], http://www.guidetopharmacology.org/GRAC/LigandDisplayForward?ligandId=8011 (pIC_50_ 6.2) [http://www.ncbi.nlm.nih.gov/pubmed/23855836?dopt=AbstractPlus]	–	http://www.guidetopharmacology.org/GRAC/LigandDisplayForward?ligandId=8011 (pIC_50_ > 10) [http://www.ncbi.nlm.nih.gov/pubmed/23855836?dopt=AbstractPlus], http://www.guidetopharmacology.org/GRAC/LigandDisplayForward?ligandId=6741 (*in vitro* activity against recombinant enzyme) (pIC_50_ 8.6) [http://www.ncbi.nlm.nih.gov/pubmed/20959606?dopt=AbstractPlus], http://www.guidetopharmacology.org/GRAC/LigandDisplayForward?ligandId=5701 (pIC_50_ 8.5) [http://www.ncbi.nlm.nih.gov/pubmed/19584227?dopt=AbstractPlus], http://www.guidetopharmacology.org/GRAC/LigandDisplayForward?ligandId=7965 (pIC_50_ 8.2–8.3) [651, http://www.ncbi.nlm.nih.gov/pubmed/16622124?dopt=AbstractPlus], http://www.guidetopharmacology.org/GRAC/LigandDisplayForward?ligandId=7888 (pIC_50_ 8.2) [http://www.ncbi.nlm.nih.gov/pubmed/21981714?dopt=AbstractPlus], http://www.guidetopharmacology.org/GRAC/LigandDisplayForward?ligandId=7950 (pIC_50_ 8.1) [http://www.ncbi.nlm.nih.gov/pubmed/18606717?dopt=AbstractPlus], http://www.guidetopharmacology.org/GRAC/LigandDisplayForward?ligandId=7955 (pIC_50_ 6.5) [http://www.ncbi.nlm.nih.gov/pubmed/23726034?dopt=AbstractPlus]	http://www.guidetopharmacology.org/GRAC/LigandDisplayForward?ligandId=8839 (pIC_50_ 7.6) [http://www.ncbi.nlm.nih.gov/pubmed/21322566?dopt=AbstractPlus]	http://www.guidetopharmacology.org/GRAC/LigandDisplayForward?ligandId=5701 (pIC_50_ 8) [http://www.ncbi.nlm.nih.gov/pubmed/17601739?dopt=AbstractPlus]	–	–
Sub/family‐selective inhibitors	http://www.guidetopharmacology.org/GRAC/LigandDisplayForward?ligandId=5682 (pIC_50_ 7.1) [http://www.ncbi.nlm.nih.gov/pubmed/18754654?dopt=AbstractPlus]	http://www.guidetopharmacology.org/GRAC/LigandDisplayForward?ligandId=2844 (pIC_50_ 4.5–5) [http://www.ncbi.nlm.nih.gov/pubmed/21704602?dopt=AbstractPlus]	http://www.guidetopharmacology.org/GRAC/LigandDisplayForward?ligandId=5682 (pIC_50_ 8.5) [http://www.ncbi.nlm.nih.gov/pubmed/18754654?dopt=AbstractPlus]	–	–	–	–
Selective inhibitors	http://www.guidetopharmacology.org/GRAC/LigandDisplayForward?ligandId=6653 (pIC_50_ 7.6) [http://www.ncbi.nlm.nih.gov/pubmed/22544264?dopt=AbstractPlus]	–	–	–	–	–	–

### Comments

http://www.guidetopharmacology.org/GRAC/LigandDisplayForward?ligandId=6060 also inhibits type III phosphatidylinositol 4‐kinases and polo‐like kinase [http://www.ncbi.nlm.nih.gov/pubmed/15664519?dopt=AbstractPlus]. PIK93 also inhibits PI 3‐kinases [http://www.ncbi.nlm.nih.gov/pubmed/16647110?dopt=AbstractPlus]. Adenosine activates http://www.guidetopharmacology.org/GRAC/FamilyDisplayForward?familyId=3.

### Further reading on Phosphatidylinositol kinases

Raphael J *et al*. (2018) Phosphoinositide 3‐kinase inhibitors in advanced breast cancer: A systematic review and meta‐analysis. *Eur J Cancer* **91**: 38‐46 https://www.ncbi.nlm.nih.gov/pubmed/29331750

Wang D *et al*. (2019) Upstream regulators of phosphoinositide 3‐kinase and their role in diseases. *J Cell Physiol*. https://www.ncbi.nlm.nih.gov/pubmed/30710358

Goncalves MD *et al*. (2018) Phosphatidylinositol 3‐Kinase, Growth Disorders, and Cancer. *N Engl J Med* **379**: 2052‐2062 https://www.ncbi.nlm.nih.gov/pubmed/30462943

## http://www.guidetopharmacology.org/GRAC/FamilyDisplayForward?familyId=782

### Overview

PIP_2_ is generated by phosphorylation of PI 4‐phosphate or PI 5‐phosphate by type I PI 4‐phosphate 5‐kinases or type II PI 5‐phosphate 4‐kinases.

**Table:** 

Nomenclature	http://www.guidetopharmacology.org/GRAC/ObjectDisplayForward?objectId=2164	http://www.guidetopharmacology.org/GRAC/ObjectDisplayForward?objectId=2500	http://www.guidetopharmacology.org/GRAC/ObjectDisplayForward?objectId=2165	http://www.guidetopharmacology.org/GRAC/ObjectDisplayForward?objectId=2858	http://www.guidetopharmacology.org/GRAC/ObjectDisplayForward?objectId=2162	http://www.guidetopharmacology.org/GRAC/ObjectDisplayForward?objectId=2163
Common abbreviation	PIP5K1A	PIP5K1B	PIP5K1C	PIP4K2A	PIP4K2B	PIP4K2C
HGNC, UniProt	https://www.genenames.org/data/gene‐symbol‐report/#!/hgnc_id/HGNC:8994, http://www.uniprot.org/uniprot/Q99755	https://www.genenames.org/data/gene‐symbol‐report/#!/hgnc_id/HGNC:8995, http://www.uniprot.org/uniprot/O14986	https://www.genenames.org/data/gene‐symbol‐report/#!/hgnc_id/HGNC:8996, http://www.uniprot.org/uniprot/O60331	https://www.genenames.org/data/gene‐symbol‐report/#!/hgnc_id/HGNC:8997, http://www.uniprot.org/uniprot/P48426	https://www.genenames.org/data/gene‐symbol‐report/#!/hgnc_id/HGNC:8998, http://www.uniprot.org/uniprot/P78356	https://www.genenames.org/data/gene‐symbol‐report/#!/hgnc_id/HGNC:23786, http://www.uniprot.org/uniprot/Q8TBX8
EC number	http://www.genome.jp/dbget‐bin/www_bget?ec:2.7.1.68	http://www.genome.jp/dbget‐bin/www_bget?ec:2.7.1.68	http://www.genome.jp/dbget‐bin/www_bget?ec:2.7.1.68	http://www.genome.jp/dbget‐bin/www_bget?ec:2.7.1.149 ATP + 1‐phosphatidyl‐1D‐*myo*‐inositol 5‐phosphate <=> ADP + 1‐phosphatidyl‐1D‐*myo*‐inositol 4,5‐bisphosphate	http://www.genome.jp/dbget‐bin/www_bget?ec:2.7.1.149	http://www.genome.jp/dbget‐bin/www_bget?ec:2.7.1.149
Inhibitors	http://www.guidetopharmacology.org/GRAC/LigandDisplayForward?ligandId=8444 [http://www.ncbi.nlm.nih.gov/pubmed/25071204?dopt=AbstractPlus]	–	–	–	–	–

### Further reading on Glycerophospholipid turnover

Cauvin C *et al*. (2015) Phosphoinositides: Lipids with informative heads and mastermind functions in cell division. *Biochim. Biophys. Acta* **1851**: 832‐43 https://www.ncbi.nlm.nih.gov/pubmed/25449648?dopt=AbstractPlus

Irvine RF. (2016) A short history of inositol lipids. *J. Lipid Res*. **57**: 1987‐1994 https://www.ncbi.nlm.nih.gov/pubmed/27623846?dopt=AbstractPlus

Poli A *et al*. (2016) Nuclear Phosphatidylinositol Signaling: Focus on Phosphatidylinositol Phosphate Kinases and Phospholipases C. *J. Cell. Physiol*. **231**: 1645‐55 https://www.ncbi.nlm.nih.gov/pubmed/26626942?dopt=AbstractPlus

## http://www.guidetopharmacology.org/GRAC/FamilyDisplayForward?familyId=278

### Overview

Haem oxygenase (heme,hydrogen‐donor:oxygen oxidoreductase (α‐methene‐oxidizing, hydroxylating)), http://www.genome.jp/kegg‐bin/search_brite?option=‐a&search_string=1.14.99.3, converts http://www.guidetopharmacology.org/GRAC/LigandDisplayForward?ligandId=4349 into http://www.guidetopharmacology.org/GRAC/LigandDisplayForward?ligandId=5153 and carbonmonoxide, utilizing http://www.guidetopharmacology.org/GRAC/LigandDisplayForward?ligandId=3041 as cofactor.

**Table:** 

Nomenclature	http://www.guidetopharmacology.org/GRAC/ObjectDisplayForward?objectId=1441	http://www.guidetopharmacology.org/GRAC/ObjectDisplayForward?objectId=1442
Common abbreviation	HO1	HO2
HGNC, UniProt	https://www.genenames.org/data/gene‐symbol‐report/#!/hgnc_id/HGNC:5013, http://www.uniprot.org/uniprot/P09601	https://www.genenames.org/data/gene‐symbol‐report/#!/hgnc_id/HGNC:5014, http://www.uniprot.org/uniprot/P30519
EC number	http://www.genome.jp/dbget‐bin/www_bget?ec:1.14.14.18 Protoheme + 3 [reduced NADPH‐hemoprotein reductase] + 3 O(2) <=> biliverdin + Fe(2+) + CO + 3 [oxidized NADPH‐hemoprotein reductase] + 3 H(2)O	http://www.genome.jp/dbget‐bin/www_bget?ec:1.14.14.18 Protoheme + 3 [reduced NADPH–hemoprotein reductase] + 3 O(2) <=> biliverdin + Fe(2+) + CO + 3 [oxidized NADPH–hemoprotein reductase] + 3 H(2)O
Inhibitors	–	http://www.guidetopharmacology.org/GRAC/LigandDisplayForward?ligandId=8873 (pIC_50_ 3.5) [http://www.ncbi.nlm.nih.gov/pubmed/16821802?dopt=AbstractPlus] – Rat

### Comments

The existence of a third non‐catalytic version of haem oxygenase, HO3, has been proposed, although this has been suggested to be a pseudogene [http://www.ncbi.nlm.nih.gov/pubmed/15246535?dopt=AbstractPlus]. The chemical http://www.guidetopharmacology.org/GRAC/LigandDisplayForward?ligandId=5279 acts as a haem oxygenase inhibitor in rat liver with an IC_50_ value of 11 nM [http://www.ncbi.nlm.nih.gov/pubmed/6947237?dopt=AbstractPlus].

### Further reading on Haem oxygenase

Magierowska K *et al*. (2018) Emerging role of carbon monoxide in regulation of cellular pathways and in the maintenance of gastric mucosal integrity. *Pharmacol Res* **129**: 56‐64 https://www.ncbi.nlm.nih.gov/pubmed/29360501

Rochette L *et al*. (2018) Redox Functions of Heme Oxygenase‐1 and Biliverdin Reductase in Diabetes Trends. *Endocrinol Metab*. **29**: 74‐85 https://www.ncbi.nlm.nih.gov/pubmed/29249571

Salerno L *et al*. (2017) Heme oxygenase‐1: A new druggable target in the management of chronic and acute myeloid leukemia. *Eur J Med Chem*. **142**: 163‐178 https://www.ncbi.nlm.nih.gov/pubmed/28756878

Sebastian VP *et al*. (2018) Heme Oxygenase‐1 as a Modulator of Intestinal Inflammation Development and Progression. *Front Immunol*. **9**: 1956 https://www.ncbi.nlm.nih.gov/pubmed/30258436

Tomczyk M *et al*. (2019) Modulation of the monocyte/macrophage system in heart failure by targeting heme oxygenase‐1. *Vascul Pharmacol*. **112**: 79‐90 https://www.ncbi.nlm.nih.gov/pubmed/30213580

Vijayan V *et al*. (2018) The macrophage heme‐heme oxygenase‐1 system and its role in inflammation. *Biochem Pharmacol*. **153**: 159‐167 https://www.ncbi.nlm.nih.gov/pubmed/29452096

## http://www.guidetopharmacology.org/GRAC/FamilyDisplayForward?familyId=279

### Overview

Hydrogen sulfide is a gasotransmitter, with similarities to nitric oxide and carbon monoxide. Although the enzymes indicated below have multiple enzymatic activities, the focus here is the generation of hydrogen sulphide (H_2_S) and the enzymatic characteristics are described accordingly. Cystathionine β‐synthase (CBS) and cystathionine γ‐lyase (CSE) are pyridoxal phosphate (PLP)‐dependent enzymes. 3‐mercaptopyruvate sulfurtransferase (3‐MPST) functions to generate H_2_S; only CAT is PLP‐dependent, while 3‐MPST is not. Thus, this third pathway is sometimes referred to as PLP‐independent. CBS and CSE are predominantly cytosolic enzymes, while 3‐MPST is found both in the cytosol and the mitochondria. For an authoritative review on the pharmacological modulation of H_2_S levels, see Szabo and Papapetropoulos, 2017 [http://www.ncbi.nlm.nih.gov/pubmed/28978633?dopt=AbstractPlus].

**Table:** 

Nomenclature	http://www.guidetopharmacology.org/GRAC/ObjectDisplayForward?objectId=1443	http://www.guidetopharmacology.org/GRAC/ObjectDisplayForward?objectId=1444	http://www.guidetopharmacology.org/GRAC/ObjectDisplayForward?objectId=1445	http://www.guidetopharmacology.org/GRAC/ObjectDisplayForward?objectId=1446
Common abbreviation	CBS	CSE	CAT	MPST
HGNC, UniProt	https://www.genenames.org/data/gene‐symbol‐report/#!/hgnc_id/HGNC:1550, http://www.uniprot.org/uniprot/P35520	https://www.genenames.org/data/gene‐symbol‐report/#!/hgnc_id/HGNC:2501, http://www.uniprot.org/uniprot/P32929	https://www.genenames.org/data/gene‐symbol‐report/#!/hgnc_id/HGNC:1564, http://www.uniprot.org/uniprot/Q16773	https://www.genenames.org/data/gene‐symbol‐report/#!/hgnc_id/HGNC:7223, http://www.uniprot.org/uniprot/P25325
EC number	http://www.genome.jp/dbget‐bin/www_bget?ec:4.2.1.22	http://www.genome.jp/dbget‐bin/www_bget?ec:4.4.1.1	http://www.genome.jp/dbget‐bin/www_bget?ec:4.4.1.13	http://www.genome.jp/dbget‐bin/www_bget?ec:2.8.1.2
Endogenous substrates	http://www.guidetopharmacology.org/GRAC/LigandDisplayForward?ligandId=4782 (*K*_m_ 6×10^‐3^M) [http://www.ncbi.nlm.nih.gov/pubmed/15520012?dopt=AbstractPlus], http://www.guidetopharmacology.org/GRAC/LigandDisplayForward?ligandId=5198 [http://www.ncbi.nlm.nih.gov/pubmed/15520012?dopt=AbstractPlus]	http://www.guidetopharmacology.org/GRAC/LigandDisplayForward?ligandId=4782	http://www.guidetopharmacology.org/GRAC/LigandDisplayForward?ligandId=4782	http://www.guidetopharmacology.org/GRAC/LigandDisplayForward?ligandId=5118 (*K*_m_ 1.2×10^‐3^M) [http://www.ncbi.nlm.nih.gov/pubmed/7608189?dopt=AbstractPlus]
Products	http://www.guidetopharmacology.org/GRAC/LigandDisplayForward?ligandId=5173	NH_3_, http://www.guidetopharmacology.org/GRAC/LigandDisplayForward?ligandId=4809	NH_3_, http://www.guidetopharmacology.org/GRAC/LigandDisplayForward?ligandId=4809	http://www.guidetopharmacology.org/GRAC/LigandDisplayForward?ligandId=4809
Cofactors	http://www.guidetopharmacology.org/GRAC/LigandDisplayForward?ligandId=5249	http://www.guidetopharmacology.org/GRAC/LigandDisplayForward?ligandId=5249	http://www.guidetopharmacology.org/GRAC/LigandDisplayForward?ligandId=5249	http://www.guidetopharmacology.org/GRAC/LigandDisplayForward?ligandId=566
Inhibitors	http://www.guidetopharmacology.org/GRAC/LigandDisplayForward?ligandId=5136 (pIC_50_ 5.1) [http://www.ncbi.nlm.nih.gov/pubmed/23488457?dopt=AbstractPlus]	http://www.guidetopharmacology.org/GRAC/LigandDisplayForward?ligandId=8860 (pIC_50_ 6) [http://www.ncbi.nlm.nih.gov/pubmed/23488457?dopt=AbstractPlus], http://www.guidetopharmacology.org/GRAC/LigandDisplayForward?ligandId=5136 (pIC_50_ 6) [http://www.ncbi.nlm.nih.gov/pubmed/23488457?dopt=AbstractPlus], http://www.guidetopharmacology.org/GRAC/LigandDisplayForward?ligandId=8859 (pIC_50_ 5.8) [http://www.ncbi.nlm.nih.gov/pubmed/23488457?dopt=AbstractPlus], http://www.guidetopharmacology.org/GRAC/LigandDisplayForward?ligandId=5247 (pIC_50_ 4.4) [http://www.ncbi.nlm.nih.gov/pubmed/23488457?dopt=AbstractPlus]	–	http://www.guidetopharmacology.org/GRAC/LigandDisplayForward?ligandId=10210 (pIC_50_ 5.6) [http://www.ncbi.nlm.nih.gov/pubmed/28079151?dopt=AbstractPlus]

### Further reading on Hydrogen sulphide synthesis

Asimakopoulou A *et al*. (2013) Selectivity of commonly used pharmacological inhibitors for cystathionine β synthase (CBS) and cystathionine γ lyase (CSE). *Br J Pharmacol*. **169**: 922‐32 https://www.ncbi.nlm.nih.gov/pubmed/23488457?dopt=AbstractPlus

Szabo C *et al*. (2017) International Union of Basic and Clinical Pharmacology. CII: Pharmacological Modulation of H2S Levels: H2S Donors and H2S Biosynthesis Inhibitors. *Pharmacol. Rev*. **69**: 497‐564 https://www.ncbi.nlm.nih.gov/pubmed/28978633?dopt=AbstractPlus

## http://www.guidetopharmacology.org/GRAC/FamilyDisplayForward?familyId=799

### Overview

Listed in this section are hydrolases not accumulated in other parts of the Concise Guide, such as monoacylglycerol lipase and acetylcholinesterase. Pancreatic lipase is the predominant mechanism of fat digestion in the alimentary system; its inhibition is associated with decreased fat absorption. CES1 is present at lower levels in the gut than CES2 (http://www.uniprot.org/uniprot/P23141), but predominates in the liver, where it is responsible for the hydrolysis of many aliphatic, aromatic and steroid esters. Hormone‐sensitive lipase is also a relatively non‐selective esterase associated with steroid ester hydrolysis and triglyceride metabolism, particularly in adipose tissue. Endothelial lipase is secreted from endothelial cells and regulates circulating cholesterol in high density lipoproteins.

**Table:** 

Nomenclature	http://www.guidetopharmacology.org/GRAC/ObjectDisplayForward?objectId=2590	http://www.guidetopharmacology.org/GRAC/ObjectDisplayForward?objectId=2593	http://www.guidetopharmacology.org/GRAC/ObjectDisplayForward?objectId=2591	http://www.guidetopharmacology.org/GRAC/ObjectDisplayForward?objectId=2592	http://www.guidetopharmacology.org/GRAC/ObjectDisplayForward?objectId=2888	http://www.guidetopharmacology.org/GRAC/ObjectDisplayForward?objectId=2889
Systematic nomenclature	–	–	–	–	CD39	CD39L1
Common abbreviation	PNLIP	LIPE	LIPG	CES1	NTPDase‐1	NTPDase‐2
HGNC, UniProt	https://www.genenames.org/data/gene‐symbol‐report/#!/hgnc_id/HGNC:9155, http://www.uniprot.org/uniprot/P16233	https://www.genenames.org/data/gene‐symbol‐report/#!/hgnc_id/HGNC:6621, http://www.uniprot.org/uniprot/Q05469	https://www.genenames.org/data/gene‐symbol‐report/#!/hgnc_id/HGNC:6623, http://www.uniprot.org/uniprot/Q9Y5X9	https://www.genenames.org/data/gene‐symbol‐report/#!/hgnc_id/HGNC:1863, http://www.uniprot.org/uniprot/P23141	https://www.genenames.org/data/gene‐symbol‐report/#!/hgnc_id/HGNC:3363, http://www.uniprot.org/uniprot/P49961	https://www.genenames.org/data/gene‐symbol‐report/#!/hgnc_id/HGNC:3364, http://www.uniprot.org/uniprot/Q9Y5L3
EC number	http://www.genome.jp/dbget‐bin/www_bget?ec:3.1.1.3	http://www.genome.jp/dbget‐bin/www_bget?ec:3.1.1.79	http://www.genome.jp/dbget‐bin/www_bget?ec:3.1.1.3	http://www.genome.jp/dbget‐bin/www_bget?ec:3.1.1.1	http://www.genome.jp/dbget‐bin/www_bget?ec:3.6.1.5 Hydrolyzes NTPs to nucleotide monophosphates (NMPs): A nucleoside 5′‐triphosphate + 2 H_2_O <=> a nucleoside 5′‐phosphate + 2 phosphate	http://www.genome.jp/dbget‐bin/www_bget?ec:3.6.1.‐ Hydrolyzes extracellular nucleotide 5′‐triphosphates: NTP>NMP + 2 phosphate
Inhibitors	http://www.guidetopharmacology.org/GRAC/LigandDisplayForward?ligandId=5277 (pIC_50_ 8.9) [66]	–	–	–	–	–
Selective inhibitors	–	–	–	–	–	http://www.guidetopharmacology.org/GRAC/LigandDisplayForward?ligandId=9033 (p*K*_i_ 5.1) [http://www.ncbi.nlm.nih.gov/pubmed/18630897?dopt=AbstractPlus]
Comments	–	–	–	–	ENTPD1 sequentially converts extracellular purine nucleotides (ATP and ADP) to the monophosphate form. Adenosine is then generated by the action of http://www.guidetopharmacology.org/GRAC/guidetopharmacology.org/GRAC/ObjectDisplayForward?objectId=1232 (CD73). ENTPD1 is the rate‐limiting step. Extracellular ATP acts as a damage‐associated molecular pattern (DAMP) that activates innate immune cells through adenosine‐induced activation of P2X and P2Y purinogenic receptors.	–

### Further reading on Hydrolases

Allard B *et al*.. (2017) The ectonucleotidases CD39 and CD73: Novel checkpoint inhibitor targets. *Immunol Rev*. **276**: 121‐144 https://www.ncbi.nlm.nih.gov/pubmed/28258700

Kishore BK *et al*. (2018) CD39‐adenosinergic axis in renal pathophysiology and therapeutics. *Purinergic Signal* **14**: 109‐120 https://www.ncbi.nlm.nih.gov/pubmed/29332180

Rasmussen HB *et al*. (2018) Carboxylesterase 1 genes: systematic review and evaluation of existing genotyping procedures. *Drug Metab Pers Ther* **33**: 3‐14 https://www.ncbi.nlm.nih.gov/pubmed/29427553

Zou LW *et al*. (2018) Carboxylesterase Inhibitors: An Update. *Curr Med Chem*. **25**: 1627‐1649 https://www.ncbi.nlm.nih.gov/pubmed/29210644

## http://www.guidetopharmacology.org/GRAC/FamilyDisplayForward?familyId=245

### Overview

The sugar alcohol D‐*myo*‐inositol is a component of the http://www.guidetopharmacology.org/GRAC/FamilyDisplayForward?familyId=244, where the principal second messenger is inositol 1,4,5‐trisphosphate, http://www.guidetopharmacology.org/GRAC/LigandDisplayForward?ligandId=4222, which acts at intracellular ligand‐gated ion channels, http://www.guidetopharmacology.org/GRAC/FamilyDisplayForward?familyId=123 to elevate intracellular calcium. http://www.guidetopharmacology.org/GRAC/LigandDisplayForward?ligandId=4222 is recycled to inositol by phosphatases or phosphorylated to form other active inositol polyphosphates. Inositol produced from dephosphorylation of http://www.guidetopharmacology.org/GRAC/LigandDisplayForward?ligandId=4222 is recycled into membrane phospholipid under the influence of phosphatidyinositol synthase activity (CDP‐diacylglycerol‐inositol 3‐phosphatidyltransferase [http://www.genome.jp/kegg‐bin/search_brite?option=‐a&search_string=2.7.8.11]).

## http://www.guidetopharmacology.org/GRAC/FamilyDisplayForward?familyId=280

### Overview

Inositol 1,4,5‐trisphosphate 3‐kinases (http://www.genome.jp/kegg‐bin/search_brite?option=‐a&search_string=2.7.1.127, http://www.ensembl.org/Homo_sapiens/Gene/Family/Genes?family=ENSFM00250000001260) catalyse the generation of inositol 1,3,4,5‐tetrakisphosphate (http://www.guidetopharmacology.org/GRAC/LigandDisplayForward?ligandId=5202) from http://www.guidetopharmacology.org/GRAC/LigandDisplayForward?ligandId=4222. IP_3_ kinase activity is enhanced in the presence of calcium/http://www.guidetopharmacology.org/GRAC/LigandDisplayForward?ligandId=2351 (https://www.genenames.org/data/gene‐symbol‐report/#!/hgnc_id/HGNC:1442 https://www.genenames.org/data/gene‐symbol‐report/#!/hgnc_id/HGNC:1445 https://www.genenames.org/data/gene‐symbol‐report/#!/hgnc_id/HGNC:1449, http://www.uniprot.org/uniprot/P62158) [http://www.ncbi.nlm.nih.gov/pubmed/2559811?dopt=AbstractPlus].

Information on members of this family may be found in the http://www.guidetopharmacology.org/GRAC/FamilyDisplayForward?familyId=280.

## http://www.guidetopharmacology.org/GRAC/FamilyDisplayForward?familyId=281

### Overview

Members of this family exhibit phosphatase activity towards IP_3_, as well as towards other inositol derivatives, including the phospholipids http://www.guidetopharmacology.org/GRAC/LigandDisplayForward?ligandId=2387 and http://www.guidetopharmacology.org/GRAC/LigandDisplayForward?ligandId=2353. With http://www.guidetopharmacology.org/GRAC/LigandDisplayForward?ligandId=4222 as substrate, 1‐phosphatase (http://www.genome.jp/dbget‐bin/www_bget?ec:3.1.3.57) generates http://www.guidetopharmacology.org/GRAC/LigandDisplayForward?ligandId=5119, 4‐phosphatases (http://www.genome.jp/dbget‐bin/www_bget?ec:3.1.3.66, http://www.ensembl.org/Homo_sapiens/Gene/Family/Genes?family=ENSFM00250000001432) generate http://www.guidetopharmacology.org/GRAC/LigandDisplayForward?ligandId=5099 and 5‐phosphatases (http://www.genome.jp/dbget‐bin/www_bget?ec:3.1.3.36 or http://www.genome.jp/dbget‐bin/www_bget?ec:3.1.3.56) generate http://www.guidetopharmacology.org/GRAC/LigandDisplayForward?ligandId=5098.

Information on members of this family may be found in the http://www.guidetopharmacology.org/GRAC/FamilyDisplayForward?familyId=281.

### Comments

*In vitro* analysis suggested http://www.guidetopharmacology.org/GRAC/LigandDisplayForward?ligandId=4222 and http://www.guidetopharmacology.org/GRAC/LigandDisplayForward?ligandId=5202 were poor substrates for SKIP, synaptojanin 1 and synaptojanin 2, but suggested that http://www.guidetopharmacology.org/GRAC/LigandDisplayForward?ligandId=2387 and http://www.guidetopharmacology.org/GRAC/LigandDisplayForward?ligandId=2353 were more efficiently hydrolysed [http://www.ncbi.nlm.nih.gov/pubmed/15474001?dopt=AbstractPlus].

## http://www.guidetopharmacology.org/GRAC/FamilyDisplayForward?familyId=282

### Overview

Inositol monophosphatase (http://www.genome.jp/kegg‐bin/search_brite?option=‐a&search_string=3.1.3.25, IMPase, *myo*‐inositol‐1(or 4)‐phosphate phosphohydrolase) is a magnesium‐dependent homodimer which hydrolyses myoinositol monophosphate to generate http://www.guidetopharmacology.org/GRAC/LigandDisplayForward?ligandId=4495 and phosphate. http://www.guidetopharmacology.org/GRAC/LigandDisplayForward?ligandId=5195 may be a physiological phosphate acceptor. http://www.guidetopharmacology.org/GRAC/LigandDisplayForward?ligandId=5212 is a nonselective un‐competitive inhibitor more potent at IMPase 1 (*p*K_i_ *ca*. 3.5, [http://www.ncbi.nlm.nih.gov/pubmed/1377913?dopt=AbstractPlus]; *p*IC_50_ 3.2, [http://www.ncbi.nlm.nih.gov/pubmed/17068342?dopt=AbstractPlus]) than IMPase 2 (*p*IC_50_ 1.8‐2.1, [http://www.ncbi.nlm.nih.gov/pubmed/17068342?dopt=AbstractPlus]). IMPase activity may be inhibited competitively by http://www.guidetopharmacology.org/GRAC/LigandDisplayForward?ligandId=5208 (*p*K_i_ 5.5, [http://www.ncbi.nlm.nih.gov/pubmed/1377913?dopt=AbstractPlus]), although the enzyme selectivity is not yet established.

**Table:** 

Nomenclature	http://www.guidetopharmacology.org/GRAC/ObjectDisplayForward?objectId=1463	http://www.guidetopharmacology.org/GRAC/ObjectDisplayForward?objectId=1464
HGNC, UniProt	https://www.genenames.org/data/gene‐symbol‐report/#!/hgnc_id/HGNC:6050, http://www.uniprot.org/uniprot/P29218	https://www.genenames.org/data/gene‐symbol‐report/#!/hgnc_id/HGNC:6051, http://www.uniprot.org/uniprot/O14732
EC number	http://www.genome.jp/dbget‐bin/www_bget?ec:3.1.3.25	http://www.genome.jp/dbget‐bin/www_bget?ec:3.1.3.25
Rank order of affinity	http://www.guidetopharmacology.org/GRAC/LigandDisplayForward?ligandId=5205 > http://www.guidetopharmacology.org/GRAC/LigandDisplayForward?ligandId=5204 > http://www.guidetopharmacology.org/GRAC/LigandDisplayForward?ligandId=5203 [http://www.ncbi.nlm.nih.gov/pubmed/1377913?dopt=AbstractPlus]	–
Inhibitors	http://www.guidetopharmacology.org/GRAC/LigandDisplayForward?ligandId=5212 (p*K*_i_ 3.5) [http://www.ncbi.nlm.nih.gov/pubmed/1377913?dopt=AbstractPlus]	–

### Comments

Polymorphisms in either of the genes encoding these enzymes have been linked with bipolar disorder [http://www.ncbi.nlm.nih.gov/pubmed/10822345?dopt=AbstractPlus, http://www.ncbi.nlm.nih.gov/pubmed/9339367?dopt=AbstractPlus, http://www.ncbi.nlm.nih.gov/pubmed/9322233?dopt=AbstractPlus]. Disruption of the gene encoding IMPase 1, but not IMPase 2, appears to mimic the effects of http://www.guidetopharmacology.org/GRAC/LigandDisplayForward?ligandId=5212 in mice [http://www.ncbi.nlm.nih.gov/pubmed/16841073?dopt=AbstractPlus, http://www.ncbi.nlm.nih.gov/pubmed/17460611?dopt=AbstractPlus].

### Further reading on Inositol phosphate turnover

Irvine R. (2016) A tale of two inositol trisphosphates. *Biochem. Soc. Trans*. **44**: 202‐11 https://www.ncbi.nlm.nih.gov/pubmed/26862207?dopt=AbstractPlus

Livermore TM *et al*. (2016) Phosphate, inositol and polyphosphates. *Biochem. Soc. Trans*. **44**: 253‐9 https://www.ncbi.nlm.nih.gov/pubmed/26862212?dopt=AbstractPlus

Miyamoto A *et al*. (2017) Probes for manipulating and monitoring IP_3. *Cell Calcium* **64**: 57‐64 https://www.ncbi.nlm.nih.gov/pubmed/27887748?dopt=AbstractPlus

Windhorst S *et al*. (2017) Inositol‐1,4,5‐trisphosphate 3‐kinase‐A (ITPKA) is frequently over‐expressed and functions as an oncogene in several tumor types. *Biochem. Pharmacol*. **137**: 1‐9 https://www.ncbi.nlm.nih.gov/pubmed/28377279?dopt=AbstractPlus

## http://www.guidetopharmacology.org/GRAC/FamilyDisplayForward?familyId=698

### Overview

Protein kinases (http://www.genome.jp/kegg‐bin/search_brite?option=‐a&search_string=2.7.11.1) use the co‐substrate http://www.guidetopharmacology.org/GRAC/LigandDisplayForward?ligandId=1713 to phosphorylate serine and/or threonine residues on target proteins. Analysis of the human genome suggests the presence of 518 protein kinases in man (divided into 15 subfamilies), with over 100 protein kinase‐like pseudogenes [http://www.ncbi.nlm.nih.gov/pubmed/12471243?dopt=AbstractPlus]. It is beyond the scope of the Concise Guide to list all these protein kinase activities, but full listings are available on the ’Detailed page’ provided for each enzyme.

Most inhibitors of these enzymes have been assessed in cell‐free investigations and so may appear to ’lose’ potency and selectivity in intact cell assays. In particular, ambient http://www.guidetopharmacology.org/GRAC/LigandDisplayForward?ligandId=1713 concentrations may be influential in responses to inhibitors, since the majority are directed at the ATP binding site [http://www.ncbi.nlm.nih.gov/pubmed/10998351?dopt=AbstractPlus].

## http://www.guidetopharmacology.org/GRAC/FamilyDisplayForward?familyId=289

### Overview

Rho kinase (also known as P160ROCK, Rho‐activated kinase) is activated by members of the Rho small G protein family, which are activated by GTP exchange factors, such as https://www.genenames.org/data/gene‐symbol‐report/#!/hgnc_id/HGNC:681 (http://www.uniprot.org/uniprot/Q92888, p115‐RhoGEF), which in turn may be activated by Gα_12/13_ subunits [http://www.ncbi.nlm.nih.gov/pubmed/9641915?dopt=AbstractPlus].

**Table:** 

Nomenclature	http://www.guidetopharmacology.org/GRAC/ObjectDisplayForward?objectId=1503	http://www.guidetopharmacology.org/GRAC/ObjectDisplayForward?objectId=1504
Systematic nomenclature	ROCK1	ROCK2
Common abbreviation	Rho kinase 1	Rho kinase 2
HGNC, UniProt	https://www.genenames.org/data/gene‐symbol‐report/#!/hgnc_id/HGNC:10251, http://www.uniprot.org/uniprot/Q13464	https://www.genenames.org/data/gene‐symbol‐report/#!/hgnc_id/HGNC:10252, http://www.uniprot.org/uniprot/O75116
EC number	http://www.genome.jp/dbget‐bin/www_bget?ec:2.7.11.1	http://www.genome.jp/dbget‐bin/www_bget?ec:2.7.11.1
Inhibitors	http://www.guidetopharmacology.org/GRAC/LigandDisplayForward?ligandId=8152 (pIC_50_ >9) [http://www.ncbi.nlm.nih.gov/pubmed/23275831?dopt=AbstractPlus], http://www.guidetopharmacology.org/GRAC/LigandDisplayForward?ligandId=5290 (pIC_50_ 5.9–7.3) [http://www.ncbi.nlm.nih.gov/pubmed/19597037?dopt=AbstractPlus, http://www.ncbi.nlm.nih.gov/pubmed/20462760?dopt=AbstractPlus], http://www.guidetopharmacology.org/GRAC/LigandDisplayForward?ligandId=5181 (p*K*_i_ 7) [http://www.ncbi.nlm.nih.gov/pubmed/21145740?dopt=AbstractPlus], http://www.guidetopharmacology.org/GRAC/LigandDisplayForward?ligandId=5290 (p*K*_i_ 6.8) [http://www.ncbi.nlm.nih.gov/pubmed/9353125?dopt=AbstractPlus], http://www.guidetopharmacology.org/GRAC/LigandDisplayForward?ligandId=5181 (pIC_50_ 5.5–5.6) [http://www.ncbi.nlm.nih.gov/pubmed/19597037?dopt=AbstractPlus, http://www.ncbi.nlm.nih.gov/pubmed/21145740?dopt=AbstractPlus]	http://www.guidetopharmacology.org/GRAC/LigandDisplayForward?ligandId=8152 (pIC_50_ >9) [http://www.ncbi.nlm.nih.gov/pubmed/23275831?dopt=AbstractPlus], http://www.guidetopharmacology.org/GRAC/LigandDisplayForward?ligandId=8197 (pIC_50_ >9) [95], http://www.guidetopharmacology.org/GRAC/LigandDisplayForward?ligandId=8037 (pIC_50_ 8.4) [http://www.ncbi.nlm.nih.gov/pubmed/17018693?dopt=AbstractPlus], http://www.guidetopharmacology.org/GRAC/LigandDisplayForward?ligandId=8184 (pIC_50_ 8.4) [http://www.ncbi.nlm.nih.gov/pubmed/20471253?dopt=AbstractPlus], http://www.guidetopharmacology.org/GRAC/LigandDisplayForward?ligandId=8205 (pIC_50_ 7.7) [http://www.ncbi.nlm.nih.gov/pubmed/20462760?dopt=AbstractPlus], http://www.guidetopharmacology.org/GRAC/LigandDisplayForward?ligandId=5290 (pIC_50_ 6.3–7.2) [http://www.ncbi.nlm.nih.gov/pubmed/19597037?dopt=AbstractPlus, http://www.ncbi.nlm.nih.gov/pubmed/20462760?dopt=AbstractPlus], http://www.guidetopharmacology.org/GRAC/LigandDisplayForward?ligandId=5290 (p*K*_i_ 6.8–6.9) [http://www.ncbi.nlm.nih.gov/pubmed/19597037?dopt=AbstractPlus, http://www.ncbi.nlm.nih.gov/pubmed/9353125?dopt=AbstractPlus], http://www.guidetopharmacology.org/GRAC/LigandDisplayForward?ligandId=5181 (pIC_50_ 5.9–5.9) [http://www.ncbi.nlm.nih.gov/pubmed/19597037?dopt=AbstractPlus, http://www.ncbi.nlm.nih.gov/pubmed/21145740?dopt=AbstractPlus]
Selective inhibitors	http://www.guidetopharmacology.org/GRAC/LigandDisplayForward?ligandId=8037 (pIC_50_ 8.8) [http://www.ncbi.nlm.nih.gov/pubmed/17018693?dopt=AbstractPlus]	–

### Further reading on Rho kinase

Feng Y *et al*. (2016) Rho Kinase (ROCK) Inhibitors and Their Therapeutic Potential. *J. Med. Chem*. **59**: 2269‐300 https://www.ncbi.nlm.nih.gov/pubmed/26486225?dopt=AbstractPlus

Nishioka T *et al*. (2015) Developing novel methods to search for substrates of protein kinases such as Rho‐kinase. *Biochim. Biophys. Acta* **1854**: 1663‐6 https://www.ncbi.nlm.nih.gov/pubmed/25770685?dopt=AbstractPlus

Shimokawa H *et al*. (2016) RhoA/Rho‐Kinase in the Cardiovascular System. *Circ. Res*. **118**: 352‐66 https://www.ncbi.nlm.nih.gov/pubmed/26838319?dopt=AbstractPlus

## http://www.guidetopharmacology.org/GRAC/FamilyDisplayForward?familyId=286

### Overview

Protein kinase C is the target for the tumour‐promoting phorbol esters, such as tetradecanoyl‐β‐phorbol acetate (TPA, also known as http://www.guidetopharmacology.org/GRAC/LigandDisplayForward?ligandId=2341).

*Classical protein kinase C isoforms:* **PKC**α, **PKC**β, and **PKC**γ are activated by http://www.guidetopharmacology.org/GRAC/LigandDisplayForward?ligandId=707 and diacylglycerol, and may be inhibited by http://www.guidetopharmacology.org/GRAC/LigandDisplayForward?ligandId=5193, http://www.guidetopharmacology.org/GRAC/LigandDisplayForward?ligandId=5156, http://www.guidetopharmacology.org/GRAC/LigandDisplayForward?ligandId=5192, http://www.guidetopharmacology.org/GRAC/LigandDisplayForward?ligandId=5953 and http://www.guidetopharmacology.org/GRAC/LigandDisplayForward?ligandId=5259.

*Novel protein kinase C isoforms:* **PKC** *δ*, **PKC** *∈*, **PKC** *η*, **PKC** *ϑ* and **PKC** *μ* are activated by diacylglycerol and may be inhibited by http://www.guidetopharmacology.org/GRAC/LigandDisplayForward?ligandId=5156, http://www.guidetopharmacology.org/GRAC/LigandDisplayForward?ligandId=5192 and http://www.guidetopharmacology.org/GRAC/LigandDisplayForward?ligandId=5953.

*Atypical protein kinase C isoforms:* **PKC** *ι*, **PKC** *ζ*.

## http://www.guidetopharmacology.org/GRAC/FamilyDisplayForward?familyId=532

**Table:** 

Nomenclature	http://www.guidetopharmacology.org/GRAC/ObjectDisplayForward?objectId=1483	http://www.guidetopharmacology.org/GRAC/ObjectDisplayForward?objectId=1484
Common abbreviation	PKCβ	PKCγ
HGNC, UniProt	https://www.genenames.org/data/gene‐symbol‐report/#!/hgnc_id/HGNC:9395, http://www.uniprot.org/uniprot/P05771	https://www.genenames.org/data/gene‐symbol‐report/#!/hgnc_id/HGNC:9402, http://www.uniprot.org/uniprot/P05129
EC number	http://www.genome.jp/dbget‐bin/www_bget?ec:2.7.11.13	http://www.genome.jp/dbget‐bin/www_bget?ec:2.7.11.13
Inhibitors	http://www.guidetopharmacology.org/GRAC/LigandDisplayForward?ligandId=7946 (pIC_50_ 8.7) [http://www.ncbi.nlm.nih.gov/pubmed/19827831?dopt=AbstractPlus], http://www.guidetopharmacology.org/GRAC/LigandDisplayForward?ligandId=5192 (pIC_50_ 8.1) [http://www.ncbi.nlm.nih.gov/pubmed/8772178?dopt=AbstractPlus], http://www.guidetopharmacology.org/GRAC/LigandDisplayForward?ligandId=5193 (pIC_50_ 7.8) [http://www.ncbi.nlm.nih.gov/pubmed/1874734?dopt=AbstractPlus] – Bovine, http://www.guidetopharmacology.org/GRAC/LigandDisplayForward?ligandId=7907 (pIC_50_ 7.5) [http://www.ncbi.nlm.nih.gov/pubmed/8022414?dopt=AbstractPlus]	http://www.guidetopharmacology.org/GRAC/LigandDisplayForward?ligandId=5192 (pIC_50_ 8.2) [zhttp://www.ncbi.nlm.nih.gov/pubmed/8772178?dopt=AbstractPlus], http://www.guidetopharmacology.org/GRAC/LigandDisplayForward?ligandId=7907 (pIC_50_ 7.5) [http://www.ncbi.nlm.nih.gov/pubmed/8022414?dopt=AbstractPlus]
Selective inhibitors	http://www.guidetopharmacology.org/GRAC/LigandDisplayForward?ligandId=5263 (pIC_50_ 8.2) [http://www.ncbi.nlm.nih.gov/pubmed/8709095?dopt=AbstractPlus], http://www.guidetopharmacology.org/GRAC/LigandDisplayForward?ligandId=5693 (pIC_50_ 7.5) [http://www.ncbi.nlm.nih.gov/pubmed/12749884?dopt=AbstractPlus], http://www.guidetopharmacology.org/GRAC/LigandDisplayForward?ligandId=5163 (pIC_50_ 6.4) [http://www.ncbi.nlm.nih.gov/pubmed/9121494?dopt=AbstractPlus]	–

## http://www.guidetopharmacology.org/GRAC/FamilyDisplayForward?familyId=533

**Table:** 

		
Nomenclature	http://www.guidetopharmacology.org/GRAC/ObjectDisplayForward?objectId=1482	http://www.guidetopharmacology.org/GRAC/ObjectDisplayForward?objectId=1485
Common abbreviation	PKCα	PKC*δ*
HGNC, UniProt	https://www.genenames.org/data/gene‐symbol‐report/#!/hgnc_id/HGNC:9393, http://www.uniprot.org/uniprot/P17252	https://www.genenames.org/data/gene‐symbol‐report/#!/hgnc_id/HGNC:9399, http://www.uniprot.org/uniprot/Q05655
EC number	http://www.genome.jp/dbget‐bin/www_bget?ec:2.7.11.13	http://www.genome.jp/dbget‐bin/www_bget?ec:2.7.11.13
Activators	–	http://www.guidetopharmacology.org/GRAC/LigandDisplayForward?ligandId=7443 (p*K*_i_ 9.4) [http://www.ncbi.nlm.nih.gov/pubmed/15126366?dopt=AbstractPlus]
Inhibitors	http://www.guidetopharmacology.org/GRAC/LigandDisplayForward?ligandId=7946 (pIC_50_ 8.7) [http://www.ncbi.nlm.nih.gov/pubmed/19827831?dopt=AbstractPlus], http://www.guidetopharmacology.org/GRAC/LigandDisplayForward?ligandId=5192 (pIC_50_ 8.1) [http://www.ncbi.nlm.nih.gov/pubmed/8772178?dopt=AbstractPlus], http://www.guidetopharmacology.org/GRAC/LigandDisplayForward?ligandId=7907 (pIC_50_ 7.5) [http://www.ncbi.nlm.nih.gov/pubmed/8022414?dopt=AbstractPlus]	http://www.guidetopharmacology.org/GRAC/LigandDisplayForward?ligandId=7946 (pIC_50_ 8.9) [http://www.ncbi.nlm.nih.gov/pubmed/19827831?dopt=AbstractPlus], http://www.guidetopharmacology.org/GRAC/LigandDisplayForward?ligandId=5192 (pIC_50_ 8) [http://www.ncbi.nlm.nih.gov/pubmed/8772178?dopt=AbstractPlus]

## http://www.guidetopharmacology.org/GRAC/FamilyDisplayForward?familyId=534

**Table:** 

Nomenclature	http://www.guidetopharmacology.org/GRAC/ObjectDisplayForward?objectId=1486
Common abbreviation	PKC*∈*
HGNC, UniProt	https://www.genenames.org/data/gene‐symbol‐report/#!/hgnc_id/HGNC:9401, http://www.uniprot.org/uniprot/Q02156
EC number	http://www.genome.jp/dbget‐bin/www_bget?ec:2.7.11.13
Inhibitors	http://www.guidetopharmacology.org/GRAC/LigandDisplayForward?ligandId=7946 (pIC_50_ 8.2) [http://www.ncbi.nlm.nih.gov/pubmed/19827831?dopt=AbstractPlus]

## Further reading on Protein kinase C (PKC) family

Igumenova TI. (2015) Dynamics and Membrane Interactions of Protein Kinase C. *Biochemistry* **54**: 4953‐68 https://www.ncbi.nlm.nih.gov/pubmed/26214365?dopt=AbstractPlus

Newton AC *et al*. (2017) Reversing the Paradigm: Protein Kinase C as a Tumor Suppressor. *Trends Pharmacol. Sci*. **38**: 438‐447 https://www.ncbi.nlm.nih.gov/pubmed/28283201?dopt=AbstractPlus

Salzer E *et al*. (2016) Protein Kinase C δ: a Gatekeeper of Immune Homeostasis. *J. Clin. Immunol*. **36**: 631‐40 https://www.ncbi.nlm.nih.gov/pubmed/27541826?dopt=AbstractPlus

## http://www.guidetopharmacology.org/GRAC/FamilyDisplayForward?familyId=529

**Table:** 

Nomenclature	http://www.guidetopharmacology.org/GRAC/ObjectDisplayForward?objectId=2109
Common abbreviation	mTOR
HGNC, UniProt	https://www.genenames.org/data/gene‐symbol‐report/#!/hgnc_id/HGNC:3942, http://www.uniprot.org/uniprot/P42345
EC number	http://www.genome.jp/dbget‐bin/www_bget?ec:2.7.11.1
Inhibitors	http://www.guidetopharmacology.org/GRAC/LigandDisplayForward?ligandId=7884 (pIC_50_ 9.7) [http://www.ncbi.nlm.nih.gov/pubmed/21482695?dopt=AbstractPlus], http://www.guidetopharmacology.org/GRAC/LigandDisplayForward?ligandId=8004 (pIC_50_ 9.5) [http://www.ncbi.nlm.nih.gov/pubmed/20860370?dopt=AbstractPlus], http://www.guidetopharmacology.org/GRAC/LigandDisplayForward?ligandId=7933 (pIC_50_ 9) [http://www.ncbi.nlm.nih.gov/pubmed/22367541?dopt=AbstractPlus], http://www.guidetopharmacology.org/GRAC/LigandDisplayForward?ligandId=7933 (p*K*_i_ 8.9) [http://www.ncbi.nlm.nih.gov/pubmed/22367541?dopt=AbstractPlus], http://www.guidetopharmacology.org/GRAC/LigandDisplayForward?ligandId=7940 (pIC_50_ 8.8) [http://www.ncbi.nlm.nih.gov/pubmed/20166697?dopt=AbstractPlus], http://www.guidetopharmacology.org/GRAC/LigandDisplayForward?ligandId=7950 (pIC_50_ 8.2) [http://www.ncbi.nlm.nih.gov/pubmed/18606717?dopt=AbstractPlus], http://www.guidetopharmacology.org/GRAC/LigandDisplayForward?ligandId=8013 (pIC_50_ 8) [http://www.ncbi.nlm.nih.gov/pubmed/18849971?dopt=AbstractPlus], http://www.guidetopharmacology.org/GRAC/LigandDisplayForward?ligandId=7973 (pIC_50_ 8) [http://www.ncbi.nlm.nih.gov/pubmed/23394126?dopt=AbstractPlus], http://www.guidetopharmacology.org/GRAC/LigandDisplayForward?ligandId=7936 (p*K*_i_ 7.8) [http://www.ncbi.nlm.nih.gov/pubmed/24900269?dopt=AbstractPlus], http://www.guidetopharmacology.org/GRAC/LigandDisplayForward?ligandId=7888 (p*K*_i_ 7.8) [http://www.ncbi.nlm.nih.gov/pubmed/21981714?dopt=AbstractPlus]
Selective inhibitors	http://www.guidetopharmacology.org/GRAC/LigandDisplayForward?ligandId=5889 (pIC_50_ 8.7) [http://www.ncbi.nlm.nih.gov/pubmed/9723437?dopt=AbstractPlus], http://www.guidetopharmacology.org/GRAC/LigandDisplayForward?ligandId=5704 (pIC_50_ 8.1) [http://www.ncbi.nlm.nih.gov/pubmed/18849971?dopt=AbstractPlus], http://www.guidetopharmacology.org/GRAC/LigandDisplayForward?ligandId=5892 (pIC_50_ 5.8) [http://www.ncbi.nlm.nih.gov/pubmed/21438579?dopt=AbstractPlus]

## Further reading on FRAP subfamily

Hukelmann JL *et al*. (2016) The cytotoxic T cell proteome and its shaping by the kinase mTOR. *Nat. Immunol*. **1**7: 104‐12 https://www.ncbi.nlm.nih.gov/pubmed/26551880?dopt=AbstractPlus

Saxton RA *et al*. (2017) mTOR Signaling in Growth, Metabolism, and Disease. *Cell* **169**: 361‐371 https://www.ncbi.nlm.nih.gov/pubmed/28388417?dopt=AbstractPlus

## http://www.guidetopharmacology.org/GRAC/FamilyDisplayForward?familyId=453

### Overview

Five of the cyclin‐dependent kinases (CDKs: 7, 8, 9, 12, and 13) are involved in the phosphorylation of serine residues in the C‐terminal domain of RNA polymerase II, the enzyme that is responsible for the transcription of protein‐coding genes into mRNA in eukaryotes. Phosphorylation of RNA polymerase II at Ser5 is essential for transcriptional initiation, and phosphorylation of Ser 2 contributes to transcriptional elongation and termination. All five of the C‐terminal domain kinases can phosphorylate Ser5, but only CDK9, CDK12, and CDK13 can phosphorylate at Ser2 [http://www.ncbi.nlm.nih.gov/pubmed/24879308?dopt=AbstractPlus, http://www.ncbi.nlm.nih.gov/pubmed/22512864?dopt=AbstractPlus, http://www.ncbi.nlm.nih.gov/pubmed/25561469?dopt=AbstractPlus].

## http://www.guidetopharmacology.org/GRAC/FamilyDisplayForward?familyId=488

**Table:** 

Nomenclature	http://www.guidetopharmacology.org/GRAC/ObjectDisplayForward?objectId=1976	http://www.guidetopharmacology.org/GRAC/ObjectDisplayForward?objectId=1978
Common abbreviation	CDK4	CDK6
HGNC, UniProt	https://www.genenames.org/data/gene‐symbol‐report/#!/hgnc_id/HGNC:1773, http://www.uniprot.org/uniprot/P11802	https://www.genenames.org/data/gene‐symbol‐report/#!/hgnc_id/HGNC:1777, http://www.uniprot.org/uniprot/Q00534
EC number	http://www.genome.jp/dbget‐bin/www_bget?ec:2.7.11.22	http://www.genome.jp/dbget‐bin/www_bget?ec:2.7.11.22
Inhibitors	http://www.guidetopharmacology.org/GRAC/LigandDisplayForward?ligandId=5707 (p*K*_i_ 9) [http://www.ncbi.nlm.nih.gov/pubmed/17121911?dopt=AbstractPlus], http://www.guidetopharmacology.org/GRAC/LigandDisplayForward?ligandId=7380 (pIC_50_ 8) [http://www.ncbi.nlm.nih.gov/pubmed/15542782?dopt=AbstractPlus], http://www.guidetopharmacology.org/GRAC/LigandDisplayForward?ligandId=8203 (pIC_50_ 7.7) [144], http://www.guidetopharmacology.org/GRAC/LigandDisplayForward?ligandId=7934 (pIC_50_ 7.2) [http://www.ncbi.nlm.nih.gov/pubmed/17363486?dopt=AbstractPlus], http://www.guidetopharmacology.org/GRAC/LigandDisplayForward?ligandId=5680 (p*K*_i_ 7.2) [http://www.ncbi.nlm.nih.gov/pubmed/8674031?dopt=AbstractPlus]	http://www.guidetopharmacology.org/GRAC/LigandDisplayForward?ligandId=7380 (pIC_50_ 7.8) [http://www.ncbi.nlm.nih.gov/pubmed/15542782?dopt=AbstractPlus]

### Comments on Cyclin‐dependent kinase (CDK) family

The development of CDK inhibitors as anticancer drugs is reviewed in [http://www.ncbi.nlm.nih.gov/pubmed/26115571?dopt=AbstractPlus], with detailed content covering CDK4 and CDK6 inhibitors that are under clinical evaluation. Data produced by Jorda et al. (2018) highlights the caution that must be used when deploying commercially available CDK inhibitors as pharmacological probes [http://www.ncbi.nlm.nih.gov/pubmed/30234987?dopt=AbstractPlus], as most of them are more promiscuous in their selectivity than indicated. To make their findings easily accessible the Jorda data is hosted on the http://rustreg.upol.cz/CDKiDB/.

## http://www.guidetopharmacology.org/GRAC/FamilyDisplayForward?familyId=509

**Table:** 

Nomenclature	http://www.guidetopharmacology.org/GRAC/ObjectDisplayForward?objectId=2030
Common abbreviation	GSK3B
HGNC, UniProt	https://www.genenames.org/data/gene‐symbol‐report/#!/hgnc_id/HGNC:4617, http://www.uniprot.org/uniprot/P49841
EC number	http://www.genome.jp/dbget‐bin/www_bget?ec:2.7.11.26
Inhibitors	http://www.guidetopharmacology.org/GRAC/LigandDisplayForward?ligandId=8016 (pIC_50_ 9.2) [http://www.ncbi.nlm.nih.gov/pubmed/12606497?dopt=AbstractPlus], http://www.guidetopharmacology.org/GRAC/LigandDisplayForward?ligandId=7958 (pIC_50_ 9) [http://www.ncbi.nlm.nih.gov/pubmed/15267232?dopt=AbstractPlus], http://www.guidetopharmacology.org/GRAC/LigandDisplayForward?ligandId=8014 (pIC_50_ 8.2) [http://www.ncbi.nlm.nih.gov/pubmed/12606497?dopt=AbstractPlus], http://www.guidetopharmacology.org/GRAC/LigandDisplayForward?ligandId=8015 (pIC_50_ ~8.1) [http://www.ncbi.nlm.nih.gov/pubmed/11033082?dopt=AbstractPlus], http://www.guidetopharmacology.org/GRAC/LigandDisplayForward?ligandId=8018 (pIC_50_ 7.7) [http://www.ncbi.nlm.nih.gov/pubmed/14698171?dopt=AbstractPlus], http://www.guidetopharmacology.org/GRAC/LigandDisplayForward?ligandId=8019 (pIC_50_ ~7.4) [http://www.ncbi.nlm.nih.gov/pubmed/11033082?dopt=AbstractPlus], http://www.guidetopharmacology.org/GRAC/LigandDisplayForward?ligandId=8017 (pIC_50_ 7.3) [http://www.ncbi.nlm.nih.gov/pubmed/20708937?dopt=AbstractPlus]
Selective inhibitors	http://www.guidetopharmacology.org/GRAC/LigandDisplayForward?ligandId=8478 (p*K*_i_ 8.3) [http://www.ncbi.nlm.nih.gov/pubmed/22489897?dopt=AbstractPlus]
Comments	Due to its Tau phosphorylating activity, small molecule inhibitors of GSK‐3β are being investigated as potential treatments for Alzheimer's disease (AD) [http://www.ncbi.nlm.nih.gov/pubmed/22489897?dopt=AbstractPlus]. GSK‐3β also plays a role in canonical Wnt pathway signalling, the normal activity of which is crucial for the maintenance of normal bone mass. It is hypothesised that small molecule inhibitors of GSK‐3β may provide effective therapeutics for the treatment of diseases characterised by low bone mass [http://www.ncbi.nlm.nih.gov/pubmed/22142634?dopt=AbstractPlus].

### Further reading on GSK subfamily

Beurel E *et al*. (2015) Glycogen synthase kinase‐3 (GSK3): regulation, actions, and diseases. *Pharmacol. Ther*. **148**: 114‐31 https://www.ncbi.nlm.nih.gov/pubmed/25435019?dopt=AbstractPlus

Domoto T *et al*. (2016) Glycogen synthase kinase‐3β is a pivotal mediator of cancer invasion and resistance to therapy. *Cancer Sci*. **107**: 1363‐1372 https://www.ncbi.nlm.nih.gov/pubmed/27486911?dopt=AbstractPlus

Khan I *et al*. (2017) Natural and synthetic bioactive inhibitors of glycogen synthase kinase. *Eur J Med Chem* **125**: 464‐477 https://www.ncbi.nlm.nih.gov/pubmed/27689729?dopt=AbstractPlus

Maqbool M *et al*. (2016) Pivotal role of glycogen synthase kinase‐3: A therapeutic target for Alzheimer's disease. *Eur J Med Chem* **107**: 63‐81 https://www.ncbi.nlm.nih.gov/pubmed/26562543?dopt=AbstractPlus

## http://www.guidetopharmacology.org/GRAC/FamilyDisplayForward?familyId=602

**Table:** 

Nomenclature	http://www.guidetopharmacology.org/GRAC/ObjectDisplayForward?objectId=2171
Common abbreviation	PLK4
HGNC, UniProt	https://www.genenames.org/data/gene‐symbol‐report/#!/hgnc_id/HGNC:11397, http://www.uniprot.org/uniprot/O00444
EC number	http://www.genome.jp/dbget‐bin/www_bget?ec:2.7.11.21
Inhibitors	http://www.guidetopharmacology.org/GRAC/LigandDisplayForward?ligandId=8063 (β 8.6) [http://www.ncbi.nlm.nih.gov/pubmed/25043604?dopt=AbstractPlus]

## http://www.guidetopharmacology.org/GRAC/FamilyDisplayForward?familyId=623

**Table:** 

Nomenclature	http://www.guidetopharmacology.org/GRAC/ObjectDisplayForward?objectId=2062	http://www.guidetopharmacology.org/GRAC/ObjectDisplayForward?objectId=2063
Common abbreviation	MEK1	MEK2
HGNC, UniProt	https://www.genenames.org/data/gene‐symbol‐report/#!/hgnc_id/HGNC:6840, http://www.uniprot.org/uniprot/Q02750	https://www.genenames.org/data/gene‐symbol‐report/#!/hgnc_id/HGNC:6842, http://www.uniprot.org/uniprot/P36507
EC number	http://www.genome.jp/dbget‐bin/www_bget?ec:2.7.12.2	http://www.genome.jp/dbget‐bin/www_bget?ec:2.7.12.2
Inhibitors	http://www.guidetopharmacology.org/GRAC/LigandDisplayForward?ligandId=6495 (pIC_50_ 9–9.1) [http://www.ncbi.nlm.nih.gov/pubmed/21245089?dopt=AbstractPlus, http://www.ncbi.nlm.nih.gov/pubmed/21523318?dopt=AbstractPlus], http://www.guidetopharmacology.org/GRAC/LigandDisplayForward?ligandId=7935 (pIC_50_ 8.1) [http://www.ncbi.nlm.nih.gov/pubmed/23474388?dopt=AbstractPlus]	http://www.guidetopharmacology.org/GRAC/LigandDisplayForward?ligandId=6495 (pIC_50_ 8.7) [http://www.ncbi.nlm.nih.gov/pubmed/21523318?dopt=AbstractPlus]
Allosteric modulators	http://www.guidetopharmacology.org/GRAC/LigandDisplayForward?ligandId=7921 (Negative) (pIC_50_ 7.9) [468], http://www.guidetopharmacology.org/GRAC/LigandDisplayForward?ligandId=7942 (Negative) (pIC_50_ 7.7) [http://www.ncbi.nlm.nih.gov/pubmed/19706763?dopt=AbstractPlus], http://www.guidetopharmacology.org/GRAC/LigandDisplayForward?ligandId=5676 (Negative) (p*K*_d_ 6.9) [http://www.ncbi.nlm.nih.gov/pubmed/22037378?dopt=AbstractPlus]	http://www.guidetopharmacology.org/GRAC/LigandDisplayForward?ligandId=7921 (Negative) (pIC_50_ 7.9) [468], http://www.guidetopharmacology.org/GRAC/LigandDisplayForward?ligandId=7942 (Negative) (pIC_50_ 7.3) [http://www.ncbi.nlm.nih.gov/pubmed/19706763?dopt=AbstractPlus]
Selective allosteric modulators	http://www.guidetopharmacology.org/GRAC/LigandDisplayForward?ligandId=7626 (Negative) (pIC_50_ 9.1) [500]	–

## http://www.guidetopharmacology.org/GRAC/FamilyDisplayForward?familyId=553

**Table:** 

Nomenclature	http://www.guidetopharmacology.org/GRAC/ObjectDisplayForward?objectId=1923
Common abbreviation	Abl
HGNC, UniProt	https://www.genenames.org/data/gene‐symbol‐report/#!/hgnc_id/HGNC:76, http://www.uniprot.org/uniprot/P00519
EC number	http://www.genome.jp/dbget‐bin/www_bget?ec:2.7.10.2
Inhibitors	http://www.guidetopharmacology.org/GRAC/LigandDisplayForward?ligandId=8170 (pIC_50_ 9.7) [http://www.ncbi.nlm.nih.gov/pubmed/21561767?dopt=AbstractPlus], http://www.guidetopharmacology.org/GRAC/LigandDisplayForward?ligandId=5678 (pIC_50_ 9.6) [http://www.ncbi.nlm.nih.gov/pubmed/23279183?dopt=AbstractPlus], http://www.guidetopharmacology.org/GRAC/LigandDisplayForward?ligandId=8147 (pIC_50_ 9.3) [http://www.ncbi.nlm.nih.gov/pubmed/23441572?dopt=AbstractPlus], http://www.guidetopharmacology.org/GRAC/LigandDisplayForward?ligandId=5699 (p*K*_d_ 9.2) [http://www.ncbi.nlm.nih.gov/pubmed/22037378?dopt=AbstractPlus], http://www.guidetopharmacology.org/GRAC/LigandDisplayForward?ligandId=5710 (pIC_50_ 9) [http://www.ncbi.nlm.nih.gov/pubmed/12543790?dopt=AbstractPlus], http://www.guidetopharmacology.org/GRAC/LigandDisplayForward?ligandId=5699 (pIC_50_ ~8.3) [http://www.ncbi.nlm.nih.gov/pubmed/12154025?dopt=AbstractPlus], http://www.guidetopharmacology.org/GRAC/LigandDisplayForward?ligandId=7906 (pIC_50_ 7.6–8.2) [http://www.ncbi.nlm.nih.gov/pubmed/17376680?dopt=AbstractPlus, http://www.ncbi.nlm.nih.gov/pubmed/16105974?dopt=AbstractPlus], http://www.guidetopharmacology.org/GRAC/LigandDisplayForward?ligandId=5890 (pIC_50_ 8.1) [http://www.ncbi.nlm.nih.gov/pubmed/20513156?dopt=AbstractPlus], http://www.guidetopharmacology.org/GRAC/LigandDisplayForward?ligandId=5697 (pIC_50_ 7.8) [http://www.ncbi.nlm.nih.gov/pubmed/15930265?dopt=AbstractPlus], http://www.guidetopharmacology.org/GRAC/LigandDisplayForward?ligandId=8013 (pIC_50_ 7.7) [http://www.ncbi.nlm.nih.gov/pubmed/18849971?dopt=AbstractPlus], http://www.guidetopharmacology.org/GRAC/LigandDisplayForward?ligandId=5687 (pIC_50_ 6.7) [http://www.ncbi.nlm.nih.gov/pubmed/17376680?dopt=AbstractPlus], http://www.guidetopharmacology.org/GRAC/LigandDisplayForward?ligandId=8065 (pIC_50_ 6.7) [http://www.ncbi.nlm.nih.gov/pubmed/20072125?dopt=AbstractPlus]

## http://www.guidetopharmacology.org/GRAC/FamilyDisplayForward?familyId=554

**Table:** 

Nomenclature	http://www.guidetopharmacology.org/GRAC/ObjectDisplayForward?objectId=2246
Common abbreviation	Ack
HGNC, UniProt	https://www.genenames.org/data/gene‐symbol‐report/#!/hgnc_id/HGNC:19297, http://www.uniprot.org/uniprot/Q07912
EC number	http://www.genome.jp/dbget‐bin/www_bget?ec:2.7.10.2
Inhibitors	http://www.guidetopharmacology.org/GRAC/LigandDisplayForward?ligandId=8193 (pIC_50_ 9) [http://www.ncbi.nlm.nih.gov/pubmed/17280833?dopt=AbstractPlus]

## http://www.guidetopharmacology.org/GRAC/FamilyDisplayForward?familyId=581

### Overview

Janus kinases (JAKs) are a fam.ily of four enzymes; JAK1, JAK2, JAK3 and tyrosine kinase 2 (TYK2). They are essential for cytokine signalling and are strongly linked to both cancer and inflammatory diseases.

**Table:** 

Nomenclature	http://www.guidetopharmacology.org/GRAC/ObjectDisplayForward?objectId=2047	http://www.guidetopharmacology.org/GRAC/ObjectDisplayForward?objectId=2048	http://www.guidetopharmacology.org/GRAC/ObjectDisplayForward?objectId=2049	http://www.guidetopharmacology.org/GRAC/ObjectDisplayForward?objectId=2269
Common abbreviation	JAK1	JAK2	JAK3	Tyk2
HGNC, UniProt	https://www.genenames.org/data/gene‐symbol‐report/#!/hgnc_id/HGNC:6190, http://www.uniprot.org/uniprot/P23458	https://www.genenames.org/data/gene‐symbol‐report/#!/hgnc_id/HGNC:6192, http://www.uniprot.org/uniprot/O60674	https://www.genenames.org/data/gene‐symbol‐report/#!/hgnc_id/HGNC:6193, http://www.uniprot.org/uniprot/P52333	https://www.genenames.org/data/gene‐symbol‐report/#!/hgnc_id/HGNC:12440, http://www.uniprot.org/uniprot/P29597
EC number	http://www.genome.jp/dbget‐bin/www_bget?ec:2.7.10.2	http://www.genome.jp/dbget‐bin/www_bget?ec:2.7.10.2	http://www.genome.jp/dbget‐bin/www_bget?ec:2.7.10.2	http://www.genome.jp/dbget‐bin/www_bget?ec:2.7.10.2
Inhibitors	http://www.guidetopharmacology.org/GRAC/LigandDisplayForward?ligandId=5688 (pIC_50_ 8.5–10.1) [http://www.ncbi.nlm.nih.gov/pubmed/23061660?dopt=AbstractPlus, http://www.ncbi.nlm.nih.gov/pubmed/20130243?dopt=AbstractPlus], http://www.guidetopharmacology.org/GRAC/LigandDisplayForward?ligandId=7913 (pIC_50_ 8) [http://www.ncbi.nlm.nih.gov/pubmed/24006460?dopt=AbstractPlus]	http://www.guidetopharmacology.org/GRAC/LigandDisplayForward?ligandId=7839 (pIC_50_ 9.1) [http://www.ncbi.nlm.nih.gov/pubmed/22829185?dopt=AbstractPlus], http://www.guidetopharmacology.org/GRAC/LigandDisplayForward?ligandId=7954 (pIC_50_ 9) [http://www.ncbi.nlm.nih.gov/pubmed/22015772?dopt=AbstractPlus], http://www.guidetopharmacology.org/GRAC/LigandDisplayForward?ligandId=7949 (pIC_50_ 8.9) [http://www.ncbi.nlm.nih.gov/pubmed/19143567?dopt=AbstractPlus], http://www.guidetopharmacology.org/GRAC/LigandDisplayForward?ligandId=7971 (pIC_50_ 8.7) [http://www.ncbi.nlm.nih.gov/pubmed/23127890?dopt=AbstractPlus], http://www.guidetopharmacology.org/GRAC/LigandDisplayForward?ligandId=5716 (pIC_50_ 8.5) [http://www.ncbi.nlm.nih.gov/pubmed/21106455?dopt=AbstractPlus, http://www.ncbi.nlm.nih.gov/pubmed/18394554?dopt=AbstractPlus], http://www.guidetopharmacology.org/GRAC/LigandDisplayForward?ligandId=7909 (pIC_50_ 8.4) [http://www.ncbi.nlm.nih.gov/pubmed/23584399?dopt=AbstractPlus]	http://www.guidetopharmacology.org/GRAC/LigandDisplayForward?ligandId=7949 (pIC_50_ 9) [http://www.ncbi.nlm.nih.gov/pubmed/19143567?dopt=AbstractPlus]	–
Selective inhibitors	–	http://www.guidetopharmacology.org/GRAC/LigandDisplayForward?ligandId=8123 (pIC_50_ >9) [http://www.ncbi.nlm.nih.gov/pubmed/21493067?dopt=AbstractPlus]	–	–
Comments	–	The JAK2^V617F^ mutation, which causes constitutive activation, plays an oncogenic role in the pathogenesis of the myeloproliferative disorders, polycythemia vera, essential thrombocythemia, and idiopathic myelofibrosis [http://www.ncbi.nlm.nih.gov/pubmed/17151367?dopt=AbstractPlus, http://www.ncbi.nlm.nih.gov/pubmed/17131059?dopt=AbstractPlus]. Small molecule compounds which inhibit aberrant JAK2 activity are being developed as novel anti‐cancer pharmaceuticals.	–	–

## http://www.guidetopharmacology.org/GRAC/FamilyDisplayForward?familyId=619

### Overview

Activation of Src‐family kinases leads to both stimulatory and inhibitory signaling responses, with cell‐specific and signaling pathway‐specific outcomes and redundancy of kinase function.

### Immune system

In immune cells Src kinases are involved in many signalling pathways, including ITAM‐ and ITIM‐domain‐containing receptor signaling, integrin signaling, and responses to chemokines/chemoattractants, cytokines, innate immune stimuli and a large variety of non‐immune cell specific stimuli (UV irradiation, heat, osmotic shock *etc*.). In many cases Src kinases signal to MAP kinase or NF‐κB pathways, but they can also modulate other pathways through less well characterized mechanisms.

The primary T cell Src kinases are Lck and Fyn; the main B cell Srcs are Lyn, Fyn and Blk. Mast cells express Fyn and Lyn, with low expression of Src.

**Table:** 

Nomenclature	http://www.guidetopharmacology.org/GRAC/ObjectDisplayForward?objectId=1940	http://www.guidetopharmacology.org/GRAC/ObjectDisplayForward?objectId=2025	http://www.guidetopharmacology.org/GRAC/ObjectDisplayForward?objectId=2026	http://www.guidetopharmacology.org/GRAC/ObjectDisplayForward?objectId=2060	http://www.guidetopharmacology.org/GRAC/ObjectDisplayForward?objectId=2206
Common abbreviation	Blk	FRK	Fyn	Lyn	Src
HGNC, UniProt	https://www.genenames.org/data/gene‐symbol‐report/#!/hgnc_id/HGNC:1057, http://www.uniprot.org/uniprot/P51451	https://www.genenames.org/data/gene‐symbol‐report/#!/hgnc_id/HGNC:3955, http://www.uniprot.org/uniprot/P42685	https://www.genenames.org/data/gene‐symbol‐report/#!/hgnc_id/HGNC:4037, http://www.uniprot.org/uniprot/P06241	https://www.genenames.org/data/gene‐symbol‐report/#!/hgnc_id/HGNC:6735, http://www.uniprot.org/uniprot/P07948	https://www.genenames.org/data/gene‐symbol‐report/#!/hgnc_id/HGNC:11283, http://www.uniprot.org/uniprot/P12931
EC number	http://www.genome.jp/dbget‐bin/www_bget?ec:2.7.10.2	http://www.genome.jp/dbget‐bin/www_bget?ec:2.7.10.2	http://www.genome.jp/dbget‐bin/www_bget?ec:2.7.10.2	http://www.genome.jp/dbget‐bin/www_bget?ec:2.7.10.2	http://www.genome.jp/dbget‐bin/www_bget?ec:2.7.10.2
Inhibitors	–	–	http://www.guidetopharmacology.org/GRAC/LigandDisplayForward?ligandId=8836 (pIC_50_ 8.2) [http://www.ncbi.nlm.nih.gov/pubmed/8557675?dopt=AbstractPlus]	http://www.guidetopharmacology.org/GRAC/LigandDisplayForward?ligandId=7906 (pIC_50_ 8) [http://www.ncbi.nlm.nih.gov/pubmed/17376680?dopt=AbstractPlus]	http://www.guidetopharmacology.org/GRAC/LigandDisplayForward?ligandId=8068 (pIC_50_ 8.2) [http://www.ncbi.nlm.nih.gov/pubmed/16884310?dopt=AbstractPlus], http://www.guidetopharmacology.org/GRAC/LigandDisplayForward?ligandId=8183 (p*K*_i_ 8.1) [http://www.ncbi.nlm.nih.gov/pubmed/9400019?dopt=AbstractPlus], http://www.guidetopharmacology.org/GRAC/LigandDisplayForward?ligandId=8013 (pIC_50_ 7.8) [http://www.ncbi.nlm.nih.gov/pubmed/18849971?dopt=AbstractPlus], http://www.guidetopharmacology.org/GRAC/LigandDisplayForward?ligandId=7885 (pIC_50_ 7.7) [http://www.ncbi.nlm.nih.gov/pubmed/19320489?dopt=AbstractPlus]

## http://www.guidetopharmacology.org/GRAC/FamilyDisplayForward?familyId=629

**Table:** 

Nomenclature	http://www.guidetopharmacology.org/GRAC/ObjectDisplayForward?objectId=1942	http://www.guidetopharmacology.org/GRAC/ObjectDisplayForward?objectId=1948	http://www.guidetopharmacology.org/GRAC/ObjectDisplayForward?objectId=2268
Common abbreviation	Etk	Btk	TXK
HGNC, UniProt	https://www.genenames.org/data/gene‐symbol‐report/#!/hgnc_id/HGNC:1079, http://www.uniprot.org/uniprot/P51813	https://www.genenames.org/data/gene‐symbol‐report/#!/hgnc_id/HGNC:1133, http://www.uniprot.org/uniprot/Q06187	https://www.genenames.org/data/gene‐symbol‐report/#!/hgnc_id/HGNC:12434, http://www.uniprot.org/uniprot/P42681
EC number	http://www.genome.jp/dbget‐bin/www_bget?ec:2.7.10.2	http://www.genome.jp/dbget‐bin/www_bget?ec:2.7.10.2	http://www.genome.jp/dbget‐bin/www_bget?ec:2.7.10.2
Inhibitors	http://www.guidetopharmacology.org/GRAC/LigandDisplayForward?ligandId=8145 (pIC_50_ 9.1) [http://www.ncbi.nlm.nih.gov/pubmed/24915291?dopt=AbstractPlus], http://www.guidetopharmacology.org/GRAC/LigandDisplayForward?ligandId=6912 (pIC_50_ 9.1) [http://www.ncbi.nlm.nih.gov/pubmed/22394077?dopt=AbstractPlus], http://www.guidetopharmacology.org/GRAC/LigandDisplayForward?ligandId=8148 (pIC_50_ 8.7) [http://www.ncbi.nlm.nih.gov/pubmed/24915291?dopt=AbstractPlus]	http://www.guidetopharmacology.org/GRAC/LigandDisplayForward?ligandId=6912 (pIC_50_ 9.3) [http://www.ncbi.nlm.nih.gov/pubmed/17154430?dopt=AbstractPlus], http://www.guidetopharmacology.org/GRAC/LigandDisplayForward?ligandId=8148 (pIC_50_ 8.4) [http://www.ncbi.nlm.nih.gov/pubmed/24915291?dopt=AbstractPlus], http://www.guidetopharmacology.org/GRAC/LigandDisplayForward?ligandId=8145 (pIC_50_ >8.4) [http://www.ncbi.nlm.nih.gov/pubmed/24915291?dopt=AbstractPlus]	–
Selective inhibitors	http://www.guidetopharmacology.org/GRAC/LigandDisplayForward?ligandId=9269 (pIC_50_ 8.1) [http://www.ncbi.nlm.nih.gov/pubmed/23594111?dopt=AbstractPlus]	http://www.guidetopharmacology.org/GRAC/LigandDisplayForward?ligandId=8066 (pIC_50_ 8.7) [http://www.ncbi.nlm.nih.gov/pubmed/21113169?dopt=AbstractPlus], http://www.guidetopharmacology.org/GRAC/LigandDisplayForward?ligandId=9516 (Irreversible inhibition) (pIC_50_ 7.6) [http://www.ncbi.nlm.nih.gov/pubmed/28352114?dopt=AbstractPlus]	–

## http://www.guidetopharmacology.org/GRAC/FamilyDisplayForward?familyId=610

**Table:** 

Nomenclature	http://www.guidetopharmacology.org/GRAC/ObjectDisplayForward?objectId=1943	http://www.guidetopharmacology.org/GRAC/ObjectDisplayForward?objectId=2184
Common abbreviation	B‐Raf	c‐Raf
HGNC, UniProt	https://www.genenames.org/data/gene‐symbol‐report/#!/hgnc_id/HGNC:1097, http://www.uniprot.org/uniprot/P15056	https://www.genenames.org/data/gene‐symbol‐report/#!/hgnc_id/HGNC:9829, http://www.uniprot.org/uniprot/P04049
EC number	http://www.genome.jp/dbget‐bin/www_bget?ec:2.7.11.1	http://www.genome.jp/dbget‐bin/www_bget?ec:2.7.11.1
Inhibitors	http://www.guidetopharmacology.org/GRAC/LigandDisplayForward?ligandId=5681 (pIC_50_ 9.7–9.9) [http://www.ncbi.nlm.nih.gov/pubmed/22037378?dopt=AbstractPlus, http://www.ncbi.nlm.nih.gov/pubmed/18676143?dopt=AbstractPlus], http://www.guidetopharmacology.org/GRAC/LigandDisplayForward?ligandId=6494 (pIC_50_ 8.5) [337], http://www.guidetopharmacology.org/GRAC/LigandDisplayForward?ligandId=5891 (pIC_50_ 7.6) [http://www.ncbi.nlm.nih.gov/pubmed/22222036?dopt=AbstractPlus], http://www.guidetopharmacology.org/GRAC/LigandDisplayForward?ligandId=5893 (pIC_50_ 7) [http://www.ncbi.nlm.nih.gov/pubmed/22808911?dopt=AbstractPlus], http://www.guidetopharmacology.org/GRAC/LigandDisplayForward?ligandId=5703 (p*K*_d_ 6.5) [http://www.ncbi.nlm.nih.gov/pubmed/22037378?dopt=AbstractPlus], http://www.guidetopharmacology.org/GRAC/LigandDisplayForward?ligandId=8548 (p*K*_d_ 6.3) [http://www.ncbi.nlm.nih.gov/pubmed/26061392?dopt=AbstractPlus], http://www.guidetopharmacology.org/GRAC/LigandDisplayForward?ligandId=5674 (p*K*_d_ 5.9) [http://www.ncbi.nlm.nih.gov/pubmed/22037378?dopt=AbstractPlus]	–
Selective inhibitors	–	http://www.guidetopharmacology.org/GRAC/LigandDisplayForward?ligandId=8072 (pIC_50_ 8.1) [http://www.ncbi.nlm.nih.gov/pubmed/15255937?dopt=AbstractPlus]

### Further reading on Kinases (EC 2.7.x.x)

Eglen R *et al*. (2011) Drug discovery and the human kinome: recent trends. *Pharmacol. Ther*. **130**: 144‐56 https://www.ncbi.nlm.nih.gov/pubmed/21256157?dopt=AbstractPlus

Graves LM *et al*. (2013) The dynamic nature of the kinome. *Biochem. J*. **450**: 1‐8 https://www.ncbi.nlm.nih.gov/pubmed/23343193?dopt=AbstractPlus

Liu Q *et al*. (2013) Developing irreversible inhibitors of the protein kinase cysteinome. *Chem. Biol*. **20**: 146‐59 https://www.ncbi.nlm.nih.gov/pubmed/23438744?dopt=AbstractPlus

Martin KJ *et al*. (2012) Selective kinase inhibitors as tools for neuroscience research. *Neuropharmacology* **63**: 1227–37 https://www.ncbi.nlm.nih.gov/pubmed/22846224?dopt=AbstractPlus

Tarrant MK *et al*. (2009) The chemical biology of protein phosphorylation. *Annu. Rev. Biochem*. **78**: 797‐825 https://www.ncbi.nlm.nih.gov/pubmed/19489734?dopt=AbstractPlus

Wu‐Zhang AX *et al*. (2013) Protein kinase C pharmacology: refining the toolbox. *Biochem. J*. **452**: 195‐209 https://www.ncbi.nlm.nih.gov/pubmed/23662807?dopt=AbstractPlus

## http://www.guidetopharmacology.org/GRAC/FamilyDisplayForward?familyId=104

### Overview

Lanosterol is a precursor for cholesterol, which is synthesized primarily in the liver in a pathway often described as the mevalonate or HMG‐CoA reductase pathway. The first two steps (formation of http://www.guidetopharmacology.org/GRAC/LigandDisplayForward?ligandId=3039 and the mitochondrial generation of http://www.guidetopharmacology.org/GRAC/LigandDisplayForward?ligandId=3040) are also associated with oxidation of fatty acids.

**Table:** 

Nomenclature	http://www.guidetopharmacology.org/GRAC/ObjectDisplayForward?objectId=2435	http://www.guidetopharmacology.org/GRAC/ObjectDisplayForward?objectId=2436	http://www.guidetopharmacology.org/GRAC/ObjectDisplayForward?objectId=638	http://www.guidetopharmacology.org/GRAC/ObjectDisplayForward?objectId=2432
HGNC, UniProt	https://www.genenames.org/data/gene‐symbol‐report/#!/hgnc_id/HGNC:93, http://www.uniprot.org/uniprot/P24752	https://www.genenames.org/data/gene‐symbol‐report/#!/hgnc_id/HGNC:94, http://www.uniprot.org/uniprot/Q9BWD1	https://www.genenames.org/data/gene‐symbol‐report/#!/hgnc_id/HGNC:5007, http://www.uniprot.org/uniprot/Q01581	https://www.genenames.org/data/gene‐symbol‐report/#!/hgnc_id/HGNC:5008, http://www.uniprot.org/uniprot/P54868
EC number	http://www.genome.jp/dbget‐bin/www_bget?ec:2.3.1.9: 2http://www.guidetopharmacology.org/GRAC/LigandDisplayForward?ligandId=3038 = http://www.guidetopharmacology.org/GRAC/LigandDisplayForward?ligandId=3039 + http://www.guidetopharmacology.org/GRAC/LigandDisplayForward?ligandId=3044	http://www.genome.jp/dbget‐bin/www_bget?ec:2.3.1.9 2http://www.guidetopharmacology.org/GRAC/LigandDisplayForward?ligandId=3038 = http://www.guidetopharmacology.org/GRAC/LigandDisplayForward?ligandId=3039 + http://www.guidetopharmacology.org/GRAC/LigandDisplayForward?ligandId=3044	http://www.genome.jp/dbget‐bin/www_bget?ec:2.3.3.10: http://www.guidetopharmacology.org/GRAC/LigandDisplayForward?ligandId=3038 + H_2_O+ http://www.guidetopharmacology.org/GRAC/LigandDisplayForward?ligandId=3039 ‐> http://www.guidetopharmacology.org/GRAC/LigandDisplayForward?ligandId=3040 + http://www.guidetopharmacology.org/GRAC/LigandDisplayForward?ligandId=3044	http://www.genome.jp/dbget‐bin/www_bget?ec:2.3.3.10: http://www.guidetopharmacology.org/GRAC/LigandDisplayForward?ligandId=3038 + H_2_O+ http://www.guidetopharmacology.org/GRAC/LigandDisplayForward?ligandId=3039 ‐> http://www.guidetopharmacology.org/GRAC/LigandDisplayForward?ligandId=3040 + http://www.guidetopharmacology.org/GRAC/LigandDisplayForward?ligandId=3044
Comments	–	–	HMGCoA synthase is found in cytosolic (HMGCoA synthase 1) and mitochondrial (HMGCoA synthase 2) versions; the former associated with http://www.guidetopharmacology.org/GRAC/LigandDisplayForward?ligandId=3042 synthesis and the latter with ketogenesis.	

**Table:** 

Nomenclature	http://www.guidetopharmacology.org/GRAC/ObjectDisplayForward?objectId=639
HGNC, UniProt	https://www.genenames.org/data/gene‐symbol‐report/#!/hgnc_id/HGNC:5006, http://www.uniprot.org/uniprot/P04035
EC number	http://www.genome.jp/dbget‐bin/www_bget?ec:1.1.1.34: http://www.guidetopharmacology.org/GRAC/LigandDisplayForward?ligandId=3040 + http://www.guidetopharmacology.org/GRAC/LigandDisplayForward?ligandId=3041 ‐>http://www.guidetopharmacology.org/GRAC/LigandDisplayForward?ligandId=3042 + http://www.guidetopharmacology.org/GRAC/LigandDisplayForward?ligandId=3044 + http://www.guidetopharmacology.org/GRAC/LigandDisplayForward?ligandId=3045 Reaction mechanism:: **First step:** (S)‐3‐hydroxy‐3‐methylglutaryl‐CoA + NADPH ‐> mevaldyl‐CoA + NADP^+^ **Second step:** mevaldyl‐CoA + H2O ‐>(R)‐mevalonate + NADP^+^
Inhibitors	http://www.guidetopharmacology.org/GRAC/LigandDisplayForward?ligandId=2739 (Competitive) (p*K*_i_ 9.2) [http://www.ncbi.nlm.nih.gov/pubmed/6933445?dopt=AbstractPlus], http://www.guidetopharmacology.org/GRAC/LigandDisplayForward?ligandId=2954 (Competitive) (pIC_50_ 8.3) [http://www.ncbi.nlm.nih.gov/pubmed/11349148?dopt=AbstractPlus], http://www.guidetopharmacology.org/GRAC/LigandDisplayForward?ligandId=2950 (Competitive) (p*K*_i_ 8.2) [http://www.ncbi.nlm.nih.gov/pubmed/16128575?dopt=AbstractPlus], http://www.guidetopharmacology.org/GRAC/LigandDisplayForward?ligandId=2949 (Competitive) (pIC_50_ 8.1) [http://www.ncbi.nlm.nih.gov/pubmed/11349148?dopt=AbstractPlus], http://www.guidetopharmacology.org/GRAC/LigandDisplayForward?ligandId=2950 (Competitive) (pIC_50_ 8) [http://www.ncbi.nlm.nih.gov/pubmed/15686906?dopt=AbstractPlus], http://www.guidetopharmacology.org/GRAC/LigandDisplayForward?ligandId=2955 (Competitive) (pIC_50_ 8) [http://www.ncbi.nlm.nih.gov/pubmed/11349148?dopt=AbstractPlus], http://www.guidetopharmacology.org/GRAC/LigandDisplayForward?ligandId=2951 (Competitive) (pIC_50_ 7.6) [http://www.ncbi.nlm.nih.gov/pubmed/11349148?dopt=AbstractPlus]
Comments	HMGCoA reductase is associated with intracellular membranes; enzymatic activity is inhibited by phosphorylation by AMP‐activated kinase. The enzymatic reaction is a three‐step reaction involving the intermediate generation of mevaldehyde‐CoA and mevaldehyde.

**Table:** 

Nomenclature	http://www.guidetopharmacology.org/GRAC/ObjectDisplayForward?objectId=640	http://www.guidetopharmacology.org/GRAC/ObjectDisplayForward?objectId=641	http://www.guidetopharmacology.org/GRAC/ObjectDisplayForward?objectId=642
HGNC, UniProt	https://www.genenames.org/data/gene‐symbol‐report/#!/hgnc_id/HGNC:7530, http://www.uniprot.org/uniprot/Q03426	https://www.genenames.org/data/gene‐symbol‐report/#!/hgnc_id/HGNC:9141, http://www.uniprot.org/uniprot/Q15126	https://www.genenames.org/data/gene‐symbol‐report/#!/hgnc_id/HGNC:7529, http://www.uniprot.org/uniprot/P53602
EC number	http://www.genome.jp/dbget‐bin/www_bget?ec:2.7.1.36: http://www.guidetopharmacology.org/GRAC/LigandDisplayForward?ligandId=1713 + http://www.guidetopharmacology.org/GRAC/LigandDisplayForward?ligandId=3042 ‐> http://www.guidetopharmacology.org/GRAC/LigandDisplayForward?ligandId=1712 + http://www.guidetopharmacology.org/GRAC/LigandDisplayForward?ligandId=3046	http://www.genome.jp/dbget‐bin/www_bget?ec:2.7.4.2: http://www.guidetopharmacology.org/GRAC/LigandDisplayForward?ligandId=1713 + http://www.guidetopharmacology.org/GRAC/LigandDisplayForward?ligandId=3046 = http://www.guidetopharmacology.org/GRAC/LigandDisplayForward?ligandId=1712 + http://www.guidetopharmacology.org/GRAC/LigandDisplayForward?ligandId=3047	http://www.genome.jp/dbget‐bin/www_bget?ec:4.1.1.33: http://www.guidetopharmacology.org/GRAC/LigandDisplayForward?ligandId=1713 + http://www.guidetopharmacology.org/GRAC/LigandDisplayForward?ligandId=3047 ‐> http://www.guidetopharmacology.org/GRAC/LigandDisplayForward?ligandId=1712 + http://www.guidetopharmacology.org/GRAC/LigandDisplayForward?ligandId=3048 + CO_2_ + PO_3_^4‐^
Comments	Mevalonate kinase activity is regulated by the downstream products http://www.guidetopharmacology.org/GRAC/LigandDisplayForward?ligandId=2910 and http://www.guidetopharmacology.org/GRAC/LigandDisplayForward?ligandId=3051 as an example of feedback inhibition.	–	–

**Table:** 

Nomenclature	http://www.guidetopharmacology.org/GRAC/ObjectDisplayForward?objectId=646	http://www.guidetopharmacology.org/GRAC/ObjectDisplayForward?objectId=647	http://www.guidetopharmacology.org/GRAC/ObjectDisplayForward?objectId=643
HGNC, UniProt	https://www.genenames.org/data/gene‐symbol‐report/#!/hgnc_id/HGNC:5387, http://www.uniprot.org/uniprot/Q13907	https://www.genenames.org/data/gene‐symbol‐report/#!/hgnc_id/HGNC:23487, http://www.uniprot.org/uniprot/Q9BXS1	https://www.genenames.org/data/gene‐symbol‐report/#!/hgnc_id/HGNC:4249, http://www.uniprot.org/uniprot/O95749
EC number	http://www.genome.jp/dbget‐bin/www_bget?ec:5.3.3.2: http://www.guidetopharmacology.org/GRAC/LigandDisplayForward?ligandId=3048 = http://www.guidetopharmacology.org/GRAC/LigandDisplayForward?ligandId=3049	http://www.genome.jp/dbget‐bin/www_bget?ec:5.3.3.2: http://www.guidetopharmacology.org/GRAC/LigandDisplayForward?ligandId=3048 = http://www.guidetopharmacology.org/GRAC/LigandDisplayForward?ligandId=3049	http://www.genome.jp/dbget‐bin/www_bget?ec:2.5.1.29: http://www.guidetopharmacology.org/GRAC/LigandDisplayForward?ligandId=3050 + http://www.guidetopharmacology.org/GRAC/LigandDisplayForward?ligandId=3048 ‐>http://www.guidetopharmacology.org/GRAC/LigandDisplayForward?ligandId=3052 + diphosphate http://www.genome.jp/dbget‐bin/www_bget?ec:2.5.1.10: http://www.guidetopharmacology.org/GRAC/LigandDisplayForward?ligandId=3051 + http://www.guidetopharmacology.org/GRAC/LigandDisplayForward?ligandId=3048 ‐> http://www.guidetopharmacology.org/GRAC/LigandDisplayForward?ligandId=3050 + diphosphate http://www.genome.jp/dbget‐bin/www_bget?ec:2.5.1.1: http://www.guidetopharmacology.org/GRAC/LigandDisplayForward?ligandId=3049 + http://www.guidetopharmacology.org/GRAC/LigandDisplayForward?ligandId=3048 = http://www.guidetopharmacology.org/GRAC/LigandDisplayForward?ligandId=3051 +diphosphate

**Table:** 

Nomenclature	http://www.guidetopharmacology.org/GRAC/ObjectDisplayForward?objectId=644	http://www.guidetopharmacology.org/GRAC/ObjectDisplayForward?objectId=645	http://www.guidetopharmacology.org/GRAC/ObjectDisplayForward?objectId=2433	http://www.guidetopharmacology.org/GRAC/ObjectDisplayForward?objectId=2434
HGNC, UniProt	https://www.genenames.org/data/gene‐symbol‐report/#!/hgnc_id/HGNC:3631, http://www.uniprot.org/uniprot/P14324	https://www.genenames.org/data/gene‐symbol‐report/#!/hgnc_id/HGNC:3629, http://www.uniprot.org/uniprot/P37268	https://www.genenames.org/data/gene‐symbol‐report/#!/hgnc_id/HGNC:11279, http://www.uniprot.org/uniprot/Q14534	https://www.genenames.org/data/gene‐symbol‐report/#!/hgnc_id/HGNC:6708, http://www.uniprot.org/uniprot/P48449
EC number	http://www.genome.jp/dbget‐bin/www_bget?ec:2.5.1.10: http://www.guidetopharmacology.org/GRAC/LigandDisplayForward?ligandId=3051 + http://www.guidetopharmacology.org/GRAC/LigandDisplayForward?ligandId=3048 ‐> http://www.guidetopharmacology.org/GRAC/LigandDisplayForward?ligandId=3050 + diphosphate http://www.genome.jp/dbget‐bin/www_bget?ec:2.5.1.1: http://www.guidetopharmacology.org/GRAC/LigandDisplayForward?ligandId=3049 + http://www.guidetopharmacology.org/GRAC/LigandDisplayForward?ligandId=3048 = http://www.guidetopharmacology.org/GRAC/LigandDisplayForward?ligandId=3051 + diphosphate	http://www.genome.jp/dbget‐bin/www_bget?ec:2.5.1.21: 2http://www.guidetopharmacology.org/GRAC/LigandDisplayForward?ligandId=3050 ‐> http://www.guidetopharmacology.org/GRAC/LigandDisplayForward?ligandId=3053 + diphosphate http://www.guidetopharmacology.org/GRAC/LigandDisplayForward?ligandId=3053 + NAD(P)H + H^+^ ‐> http://www.guidetopharmacology.org/GRAC/LigandDisplayForward?ligandId=3054 + diphosphate + NAD(P)^+^	http://www.genome.jp/dbget‐bin/www_bget?ec:1.14.13.132: H^+^ + http://www.guidetopharmacology.org/GRAC/LigandDisplayForward?ligandId=3041 + O_2_ + http://www.guidetopharmacology.org/GRAC/LigandDisplayForward?ligandId=3054 = H_2_O + http://www.guidetopharmacology.org/GRAC/LigandDisplayForward?ligandId=3045 + http://www.guidetopharmacology.org/GRAC/LigandDisplayForward?ligandId=6551	http://www.genome.jp/dbget‐bin/www_bget?ec:5.4.99.7: http://www.guidetopharmacology.org/GRAC/LigandDisplayForward?ligandId=6551 = http://www.guidetopharmacology.org/GRAC/LigandDisplayForward?ligandId=2746
Cofactors	–	http://www.guidetopharmacology.org/GRAC/LigandDisplayForward?ligandId=3041	–	–
Inhibitors	http://www.guidetopharmacology.org/GRAC/LigandDisplayForward?ligandId=3176 (pIC_50_ 8.4) [http://www.ncbi.nlm.nih.gov/pubmed/10620343?dopt=AbstractPlus], http://www.guidetopharmacology.org/GRAC/LigandDisplayForward?ligandId=3177 (p*K*_i_ 7.1) [http://www.ncbi.nlm.nih.gov/pubmed/18327899?dopt=AbstractPlus], http://www.guidetopharmacology.org/GRAC/LigandDisplayForward?ligandId=3141 (pIC_50_ 6.3) [http://www.ncbi.nlm.nih.gov/pubmed/10620343?dopt=AbstractPlus]	http://www.guidetopharmacology.org/GRAC/LigandDisplayForward?ligandId=3057 (p*K*_i_ 10.1) [http://www.ncbi.nlm.nih.gov/pubmed/8419946?dopt=AbstractPlus] – Rat, http://www.guidetopharmacology.org/GRAC/LigandDisplayForward?ligandId=3057 (pIC_50_ 9.2) [http://www.ncbi.nlm.nih.gov/pubmed/9473303?dopt=AbstractPlus]	–	–
Selective inhibitors	http://www.guidetopharmacology.org/GRAC/LigandDisplayForward?ligandId=3059 (p*K*_i_ 6.7) [http://www.ncbi.nlm.nih.gov/pubmed/18327899?dopt=AbstractPlus], http://www.guidetopharmacology.org/GRAC/LigandDisplayForward?ligandId=7259 (pIC_50_ 6.7) [http://www.ncbi.nlm.nih.gov/pubmed/18327899?dopt=AbstractPlus]	–	–	–

### Further reading on Lanosterol biosynthesis pathway

Moutinho M *et al*. (2017) The mevalonate pathway in neurons: It's not just about cholesterol. *Exp. Cell Res*. **360**: 55–60 https://www.ncbi.nlm.nih.gov/pubmed/28232115?dopt=AbstractPlus

Mullen PJ *et al*. (2016) The interplay between cell signalling and the mevalonate pathway in cancer. *Nat. Rev. Cancer* **16**: 718–731 https://www.ncbi.nlm.nih.gov/pubmed/27562463?dopt=AbstractPlus

Ness GC. (2015) Physiological feedback regulation of cholesterol biosynthesis: Role of translational control of hepatic HMG‐CoA reductase and possible involvement of oxylanosterols. *Biochim. Biophys. Acta* **1851**: 667–73 https://www.ncbi.nlm.nih.gov/pubmed/25701719?dopt=AbstractPlus

Porter TD. (2015) Electron Transfer Pathways in Cholesterol Synthesis. *Lipids* **50**: 927–36 https://www.ncbi.nlm.nih.gov/pubmed/26344922?dopt=AbstractPlus

Samaras K *et al*. (2016) Does statin use cause memory decline in the elderly? *Trends Cardiovasc. Med*. **26**: 550–65 https://www.ncbi.nlm.nih.gov/pubmed/27177529?dopt=AbstractPlus

## http://www.guidetopharmacology.org/GRAC/FamilyDisplayForward?familyId=920

### Overview

The *de novo* synthesis and salvage of nucleosides have been targetted for therapeutic advantage in the treatment of particular cancers and gout. Dihydrofolate reductase produces tetrahydrofolate, a cofactor required for synthesis of purines, pyrimidines and amino acids. GART allows formylation of phosphoribosylglycinamide, an early step in purine biosynthesis. Dihydroorotate dehydrogenase produces orotate, a key intermediate in pyrimidine synthesis. IMP dehydrogenase generates xanthosine monophosphate, an intermediate in GTP synthesis.

**Table:** 

Nomenclature	http://www.guidetopharmacology.org/GRAC/ObjectDisplayForward?objectId=2603	http://www.guidetopharmacology.org/GRAC/ObjectDisplayForward?objectId=2612	http://www.guidetopharmacology.org/GRAC/ObjectDisplayForward?objectId=2604	http://www.guidetopharmacology.org/GRAC/ObjectDisplayForward?objectId=2624	http://www.guidetopharmacology.org/GRAC/ObjectDisplayForward?objectId=2625	http://www.guidetopharmacology.org/GRAC/ObjectDisplayForward?objectId=2642
Common abbreviation	DHFR	GART	DHODH	IMPDH1	IMPDH2	TYMS
HGNC, UniProt	https://www.genenames.org/data/gene‐symbol‐report/#!/hgnc_id/HGNC:2861, http://www.uniprot.org/uniprot/P00374	https://www.genenames.org/data/gene‐symbol‐report/#!/hgnc_id/HGNC:4163, http://www.uniprot.org/uniprot/P22102	https://www.genenames.org/data/gene‐symbol‐report/#!/hgnc_id/HGNC:2867, http://www.uniprot.org/uniprot/Q02127	https://www.genenames.org/data/gene‐symbol‐report/#!/hgnc_id/HGNC:6052, http://www.uniprot.org/uniprot/P20839	https://www.genenames.org/data/gene‐symbol‐report/#!/hgnc_id/HGNC:6053, http://www.uniprot.org/uniprot/P12268	https://www.genenames.org/data/gene‐symbol‐report/#!/hgnc_id/HGNC:12441, http://www.uniprot.org/uniprot/P04818
EC number	http://www.genome.jp/dbget‐bin/www_bget?ec:1.5.1.3	http://www.genome.jp/dbget‐bin/www_bget?ec:2.1.2.2 http://www.genome.jp/dbget‐bin/www_bget?ec:6.3.3.1 http://www.genome.jp/dbget‐bin/www_bget?ec:6.3.4.13	http://www.genome.jp/dbget‐bin/www_bget?ec:1.3.5.2	http://www.genome.jp/dbget‐bin/www_bget?ec:1.1.1.205	http://www.genome.jp/dbget‐bin/www_bget?ec:1.1.1.205	http://www.genome.jp/dbget‐bin/www_bget?ec:2.1.1.45
Inhibitors	–	http://www.guidetopharmacology.org/GRAC/LigandDisplayForward?ligandId=6837 (p*K*_i_ 5) [http://www.ncbi.nlm.nih.gov/pubmed/9762351?dopt=AbstractPlus] – Mouse	http://www.guidetopharmacology.org/GRAC/LigandDisplayForward?ligandId=6844 (p*K*_i_ 7.5) [http://www.ncbi.nlm.nih.gov/pubmed/17228860?dopt=AbstractPlus]	http://www.guidetopharmacology.org/GRAC/LigandDisplayForward?ligandId=6832 (pIC_50_ 7.7) [http://www.ncbi.nlm.nih.gov/pubmed/1967654?dopt=AbstractPlus]	http://www.guidetopharmacology.org/GRAC/LigandDisplayForward?ligandId=6832 (pIC_50_ 7.7) [http://www.ncbi.nlm.nih.gov/pubmed/1967654?dopt=AbstractPlus]	–
Selective inhibitors	http://www.guidetopharmacology.org/GRAC/LigandDisplayForward?ligandId=4815 (p*K*_i_ 8.9) [http://www.ncbi.nlm.nih.gov/pubmed/7877140?dopt=AbstractPlus]	–	–	–	–	http://www.guidetopharmacology.org/GRAC/LigandDisplayForward?ligandId=7403 (pIC_50_ 6.5) [http://www.ncbi.nlm.nih.gov/pubmed/22739090?dopt=AbstractPlus]

**Table:** 

Nomenclature	http://www.guidetopharmacology.org/GRAC/ObjectDisplayForward?objectId=2841	http://www.guidetopharmacology.org/GRAC/ObjectDisplayForward?objectId=2646	http://www.guidetopharmacology.org/GRAC/ObjectDisplayForward?objectId=2630	http://www.guidetopharmacology.org/GRAC/ObjectDisplayForward?objectId=2631	http://www.guidetopharmacology.org/GRAC/ObjectDisplayForward?objectId=2754
Common abbreviation	PNP	XDH	ribonucleotide reductase M1	ribonucleotide reductase M2	ribonucleotide reductase M2B (TP53 inducible)
HGNC, UniProt	https://www.genenames.org/data/gene‐symbol‐report/#!/hgnc_id/HGNC:7892, http://www.uniprot.org/uniprot/P00491	https://www.genenames.org/data/gene‐symbol‐report/#!/hgnc_id/HGNC:12805, http://www.uniprot.org/uniprot/P47989	https://www.genenames.org/data/gene‐symbol‐report/#!/hgnc_id/HGNC:10451, http://www.uniprot.org/uniprot/P23921	https://www.genenames.org/data/gene‐symbol‐report/#!/hgnc_id/HGNC:10452, http://www.uniprot.org/uniprot/P31350	https://www.genenames.org/data/gene‐symbol‐report/#!/hgnc_id/HGNC:17296, http://www.uniprot.org/uniprot/Q7LG56
EC number	http://www.genome.jp/dbget‐bin/www_bget?ec:1.4.2.1 Purine‐nucleoside phosphorylase: Purine nucleoside + phosphate <=> purine + alpha‐D‐ribose 1‐phosphate Purine deoxynucleoside + phosphate <=> purine + 2′‐deoxy‐alpha‐D‐ribose 1‐phosphate	http://www.genome.jp/dbget‐bin/www_bget?ec:1.17.1.4	http://www.genome.jp/dbget‐bin/www_bget?ec:1.17.14.1	http://www.genome.jp/dbget‐bin/www_bget?ec:1.17.4.1	http://www.genome.jp/dbget‐bin/www_bget?ec:1.17.1.4
Inhibitors	–	http://www.guidetopharmacology.org/GRAC/LigandDisplayForward?ligandId=6817 (pIC_50_ 8.9) [http://www.ncbi.nlm.nih.gov/pubmed/24508129?dopt=AbstractPlus]	–	–	–

### Comments

TYMS allows the interconversion of dUMP and dTMP, thereby acting as a crucial step in DNA synthesis. PNP allows separation of a nucleoside into the nucleobase and ribose phosphate for nucleotide salvage. XDH generates urate in the purine degradation pathway. Post‐translational modifications of XDH convert the enzymatic reaction to a xanthine oxidase, allowing the interconversion of hypoxanthine and xanthine, with the production (or consumption) of reactive oxygen species.

### Further reading on Nucleoside synthesis and metabolism

Day RO *et al*. (2016) Xanthine oxidoreductase and its inhibitors: relevance for gout. *Clin Sci (Lond)*. **130**: 2167–2180 https://www.ncbi.nlm.nih.gov/pubmed/27798228

Okafor ON *et al*. (2017) Allopurinol as a therapeutic option in cardiovascular disease. *Pharmacol Ther*. **172**: 139–150 https://www.ncbi.nlm.nih.gov/pubmed/27916655

## http://www.guidetopharmacology.org/GRAC/FamilyDisplayForward?familyId=1018

### Overview

Paraoxonases (PON) are calcium‐dependent esterases, whichmay be involved in lipoprotein turnover and the conversion of lactone statin prodrugs, as well as being targets of organophosphates, such as the insecticide paraoxon.

**Table:** 

Nomenclature	http://www.guidetopharmacology.org/GRAC/ObjectDisplayForward?objectId=3052	http://www.guidetopharmacology.org/GRAC/ObjectDisplayForward?objectId=3053	http://www.guidetopharmacology.org/GRAC/ObjectDisplayForward?objectId=3054
Common abbreviation	PON1	PON2	PON3
HGNC, UniProt	https://www.genenames.org/data/gene‐symbol‐report/#!/hgnc_id/HGNC:9204, http://www.uniprot.org/uniprot/P27169	https://www.genenames.org/data/gene‐symbol‐report/#!/hgnc_id/HGNC:9205, http://www.uniprot.org/uniprot/Q15165	https://www.genenames.org/data/gene‐symbol‐report/#!/hgnc_id/HGNC:9206, http://www.uniprot.org/uniprot/Q15166
EC number	http://www.genome.jp/dbget‐bin/www_bget?ec:3.1.8.1 An aryl dialkyl phosphate + H(2)O <=> dialkyl phosphate + an aryl alcohol http://www.genome.jp/dbget‐bin/www_bget?ec:3.1.1.2 A phenyl acetate + H(2)O <=> a phenol + acetate http://www.genome.jp/dbget‐bin/www_bget?ec:3.1.1.81 An N‐acyl‐L‐homoserine lactone + H(2)O <=> an N‐acyl‐L‐homoserine	http://www.genome.jp/dbget‐bin/www_bget?ec:3.1.1.2 A phenyl acetate + H(2)O <=> a phenol + acetate http://www.genome.jp/dbget‐bin/www_bget?ec:3.1.1.81 A N‐acyl‐L‐homoserine lactone + H(2)O <=> a N‐acyl‐L‐homoserine	http://www.genome.jp/dbget‐bin/www_bget?ec:3.1.8.1 An aryl dialkyl phosphate + H(2)O <=> dialkyl phosphate + an aryl alcohol http://www.genome.jp/dbget‐bin/www_bget?ec:3.1.1.2 A phenyl acetate + H(2)O <=> a phenol + acetate http://www.genome.jp/dbget‐bin/www_bget?ec:3.1.1.81 A N‐acyl‐L‐homoserine lactone + H(2)O <=> a N‐acyl‐L‐homoserine
Comments	PON1 forms homodimers. Loss‐of‐function mutations in PON1 are associated with microvascular complications of diabetes [http://www.ncbi.nlm.nih.gov/pubmed/11918623?dopt=AbstractPlus, http://www.ncbi.nlm.nih.gov/pubmed/9661650?dopt=AbstractPlus].	PON2 forms heterotrimers [http://www.ncbi.nlm.nih.gov/pubmed/15772423?dopt=AbstractPlus].	PON3 likely forms heterodimers *in vivo* [http://www.ncbi.nlm.nih.gov/pubmed/15772423?dopt=AbstractPlus].

### Further reading on Paraoxonase

Dardiotis E *et al*. (2019) Paraoxonase‐1 genetic polymorphisms in organophosphate metabolism. *Toxicology*. **411**: 24–31 https://www.ncbi.nlm.nih.gov/pubmed/30359673

Lioudaki S *et al*. (2019) Paraoxonase‐1: Characteristics and Role in Atherosclerosis and Carotid Artery Disease. *Curr Vasc Pharmacol*. **17**: 141–146 https://www.ncbi.nlm.nih.gov/pubmed/29189170

## http://www.guidetopharmacology.org/GRAC/FamilyDisplayForward?familyId=759

### Overview

Peptidases and proteinases hydrolyse peptide bonds, and can be simply divided on the basis of whether terminal peptide bonds are cleaved (exopeptidases and exoproteinases) at the amino terminus (aminopeptidases) or carboxy terminus (carboxypeptidases). Non‐terminal peptide bonds are cleaved by endopeptidases and endoproteinases, which are divided into serine endopeptidases (EC 3.4.21.‐), cysteine endopeptidases (EC 3.4.22.‐), aspartate endopeptidases (EC 3.4.23.‐), metalloendopeptidases (EC 3.4.24.‐) and threonine endopeptidases (EC 3.4.25.‐).

Since it is beyond the scope of the Guide to list all peptidase and proteinase activities, this summary focuses on selected enzymes of significant pharmacological interest that have ligands (mostly small‐molecules) directed against them. For those interested in detailed background we recommend the MEROPS database [493] (with whom we collaborate) as an information resource [http://www.ncbi.nlm.nih.gov/pubmed/26527717?dopt=AbstractPlus].

## http://www.guidetopharmacology.org/GRAC/FamilyDisplayForward?familyId=726

**Table:** 

Nomenclature	http://www.guidetopharmacology.org/GRAC/ObjectDisplayForward?objectId=2413
HGNC, UniProt	https://www.genenames.org/data/gene‐symbol‐report/#!/hgnc_id/HGNC:9958, http://www.uniprot.org/uniprot/P00797
EC number	http://www.genome.jp/dbget‐bin/www_bget?ec:3.4.23.15
Inhibitors	http://www.guidetopharmacology.org/GRAC/LigandDisplayForward?ligandId=4812 (pIC_50_ 9.2) [http://www.ncbi.nlm.nih.gov/pubmed/18307734?dopt=AbstractPlus]

## http://www.guidetopharmacology.org/GRAC/FamilyDisplayForward?familyId=727

### Overview

Presenilin (PS)‐1 or ‐2 act as the catalytic component/ essential co‐factor of the γ‐secretase complex responsible for the final carboxy‐terminal cleavage of amyloid precursor protein (APP) [http://www.ncbi.nlm.nih.gov/pubmed/2881207?dopt=AbstractPlus] in the generation of amyloid beta (Aβ) [http://www.ncbi.nlm.nih.gov/pubmed/21115843?dopt=AbstractPlus, http://www.ncbi.nlm.nih.gov/pubmed/12660785?dopt=AbstractPlus]. Given that the accumulation and aggregation of Aβ in the brain is pivotal in the development of Alzheimer's disease (AD), inhibition of PS activity is one mechanism being investigated as a therapeutic option for AD [http://www.ncbi.nlm.nih.gov/pubmed/11378516?dopt=AbstractPlus]. Several small molecule inhibitors of PS‐1 have been investigated, with some reaching early clinical trials, but none have been formally approved. Dewji *et al*. (2015) have reported that small peptide fragments of human PS‐1 can significantly inhibit Aβ production (total Aβ, Aβ40 and Aβ42) both *in vitro* and when infused in to the brains of APP transgenic mice [http://www.ncbi.nlm.nih.gov/pubmed/25923432?dopt=AbstractPlus]. The most active small peptides in this report were http://www.guidetopharmacology.org/GRAC/LigandDisplayForward?ligandId=8344 and http://www.guidetopharmacology.org/GRAC/LigandDisplayForward?ligandId=8345, from the amino‐terminal domain of PS‐1.

Information on members of this family may be found in the http://www.guidetopharmacology.org/GRAC/FamilyDisplayForward?familyId=727.

## http://www.guidetopharmacology.org/GRAC/FamilyDisplayForward?familyId=734

### Overview

Caspases, (http://www.genome.jp/kegg‐bin/search_brite?option=‐a&search_string=3.4.22.‐) which derive their name from Cysteine ASPartate‐specific proteASES, include at least two families; initiator caspases (caspases 2, 8, 9 and 10), which are able to hydrolyse and activate a second family of effector caspases (caspases 3, 6 and 7), which themselves are able to hydrolyse further cellular proteins to bring about programmed cell death. Caspases are heterotetrameric, being made up of two pairs of subunits, generated by a single gene product, which is proteolysed to form the mature protein. Members of the mammalian inhibitors of apoptosis proteins (IAP) are able to bind the procaspases, thereby preventing maturation to active proteinases.

Information on members of this family may be found in the http://www.guidetopharmacology.org/GRAC/FamilyDisplayForward?familyId=734.

### Comments

CARD16 (Caspase recruitment domain‐containing protein 16, caspase‐1 inhibitor COP, CARD only domain‐containing protein 1, pseudo interleukin‐1β converting enzyme, pseudo‐ICE, http://www.ensembl.org/Homo_sapiens/Gene/Summary?g=ENSG00000204397;r=11:104912053‐104972158) shares sequence similarity with some of the caspases.

## http://www.guidetopharmacology.org/GRAC/FamilyDisplayForward?familyId=737

### Overview

Aminopeptidases catalyze the cleavage of amino acids from the amino (N) terminus of protein or peptide substrates, and are involved in many essential cellular functions. Members of this enzyme family may be monomeric or multi‐subunit complexes, and many are zinc metalloenzymes [http://www.ncbi.nlm.nih.gov/pubmed/8440407?dopt=AbstractPlus].

**Table:** 

Nomenclature	http://www.guidetopharmacology.org/GRAC/ObjectDisplayForward?objectId=1395
HGNC, UniProt	https://www.genenames.org/data/gene‐symbol‐report/#!/hgnc_id/HGNC:6710, http://www.uniprot.org/uniprot/P09960
EC number	http://www.genome.jp/dbget‐bin/www_bget?ec:3.3.2.6
Inhibitors	http://www.guidetopharmacology.org/GRAC/LigandDisplayForward?ligandId=5151 (p*K*_i_ 5.4) [http://www.ncbi.nlm.nih.gov/pubmed/1846352?dopt=AbstractPlus]

## http://www.guidetopharmacology.org/GRAC/FamilyDisplayForward?familyId=741

**Table:** 

Nomenclature	http://www.guidetopharmacology.org/GRAC/ObjectDisplayForward?objectId=1613
Common abbreviation	ACE
HGNC, UniProt	https://www.genenames.org/data/gene‐symbol‐report/#!/hgnc_id/HGNC:2707, http://www.uniprot.org/uniprot/P12821
EC number	http://www.genome.jp/dbget‐bin/www_bget?ec:3.4.15.1
Substrates	http://www.guidetopharmacology.org/GRAC/LigandDisplayForward?ligandId=10060
Endogenous substrates	http://www.guidetopharmacology.org/GRAC/LigandDisplayForward?ligandId=583 (https://www.genenames.org/data/gene‐symbol‐report/#!/hgnc_id/HGNC:333, http://www.uniprot.org/uniprot/P01019) > http://www.guidetopharmacology.org/GRAC/LigandDisplayForward?ligandId=2504 (https://www.genenames.org/data/gene‐symbol‐report/#!/hgnc_id/HGNC:333, http://www.uniprot.org/uniprot/P01019)
Inhibitors	http://www.guidetopharmacology.org/GRAC/LigandDisplayForward?ligandId=6463 (p*K*_i_ 9.4) [http://www.ncbi.nlm.nih.gov/pubmed/2836590?dopt=AbstractPlus] – Rabbit, http://www.guidetopharmacology.org/GRAC/LigandDisplayForward?ligandId=5158 (pK_i_ 8.4) [http://www.ncbi.nlm.nih.gov/pubmed/9187274?dopt=AbstractPlus], http://www.guidetopharmacology.org/GRAC/LigandDisplayForward?ligandId=6462
Selective inhibitors	http://www.guidetopharmacology.org/GRAC/LigandDisplayForward?ligandId=6373 (pIC_50_ 9) [http://www.ncbi.nlm.nih.gov/pubmed/17716647?dopt=AbstractPlus], http://www.guidetopharmacology.org/GRAC/LigandDisplayForward?ligandId=6461 (pIC_50_ 8.7) [http://www.ncbi.nlm.nih.gov/pubmed/2527528?dopt=AbstractPlus] – Rabbit, http://www.guidetopharmacology.org/GRAC/LigandDisplayForward?ligandId=6378 (pIC_50_ 8.7) [http://www.ncbi.nlm.nih.gov/pubmed/17547476?dopt=AbstractPlus], http://www.guidetopharmacology.org/GRAC/LigandDisplayForward?ligandId=7893 (C‐domain assay) (pIC_50_ 8.2) [http://www.ncbi.nlm.nih.gov/pubmed/20233165?dopt=AbstractPlus], http://www.guidetopharmacology.org/GRAC/LigandDisplayForward?ligandId=7894 (N‐domain selective inhibition) (pIC_50_ 8.1) [http://www.ncbi.nlm.nih.gov/pubmed/22628311?dopt=AbstractPlus], http://www.guidetopharmacology.org/GRAC/LigandDisplayForward?ligandId=6457 (pIC_50_ 8) [http://www.ncbi.nlm.nih.gov/pubmed/2481187?dopt=AbstractPlus] – Rabbit, http://www.guidetopharmacology.org/GRAC/LigandDisplayForward?ligandId=6332 (pIC_50_ 7.5) [http://www.ncbi.nlm.nih.gov/pubmed/7527095?dopt=AbstractPlus], http://www.guidetopharmacology.org/GRAC/LigandDisplayForward?ligandId=6375 (pIC_50_ 6.6) [http://www.ncbi.nlm.nih.gov/pubmed/17506720?dopt=AbstractPlus]
Comments	Reports of ACE GPI hydrolase activity [http://www.ncbi.nlm.nih.gov/pubmed/15665832?dopt=AbstractPlus] have been refuted [http://www.ncbi.nlm.nih.gov/pubmed/16270062?dopt=AbstractPlus]

## http://www.guidetopharmacology.org/GRAC/FamilyDisplayForward?familyId=738

### Overview

Matrix metalloproteinases (MMP) are calcium‐ and zinc‐dependent proteinases regulating the extracellular matrix and are often divided (*e.g*. [http://www.ncbi.nlm.nih.gov/pubmed/17275314?dopt=AbstractPlus]) on functional and structural bases into gelatinases, collagenases, stromyelinases and matrilysins, as well as membrane type‐MMP (MT‐MMP).

**Table:** 

Nomenclature	http://www.guidetopharmacology.org/GRAC/ObjectDisplayForward?objectId=1629	http://www.guidetopharmacology.org/GRAC/ObjectDisplayForward?objectId=1632
HGNC, UniProt	https://www.genenames.org/data/gene‐symbol‐report/#!/hgnc_id/HGNC:7166, http://www.uniprot.org/uniprot/P08253	https://www.genenames.org/data/gene‐symbol‐report/#!/hgnc_id/HGNC:7175, http://www.uniprot.org/uniprot/P22894
EC number	http://www.genome.jp/dbget‐bin/www_bget?ec:3.4.24.24	http://www.genome.jp/dbget‐bin/www_bget?ec:3.4.24.34
Selective inhibitors	http://www.guidetopharmacology.org/GRAC/LigandDisplayForward?ligandId=5140 [http://www.ncbi.nlm.nih.gov/pubmed/16483784?dopt=AbstractPlus]	–
Comments	MMP2 is categorised as a gelatinase with substrate specificity for gelatinase A.	MMP8 is categorised as a collagenase.

### Comments

A number of small molecule ‘broad spectrum’ inhibitors of MMP have been described, including http://www.guidetopharmacology.org/GRAC/LigandDisplayForward?ligandId=5220 and http://www.guidetopharmacology.org/GRAC/LigandDisplayForward?ligandId=5145.

**Tissue inhibitors of metalloproteinase (TIMP)** proteins are endogenous inhibitors acting to chelate MMP proteins: http://www.guidetopharmacology.org/GRAC/LigandDisplayForward?ligandId=5309 (https://www.genenames.org/data/gene‐symbol‐report/#!/hgnc_id/HGNC:11820, http://www.uniprot.org/uniprot/P01033), http://www.guidetopharmacology.org/GRAC/LigandDisplayForward?ligandId=5310 (https://www.genenames.org/data/gene‐symbol‐report/#!/hgnc_id/HGNC:11821, http://www.uniprot.org/uniprot/P16035), http://www.guidetopharmacology.org/GRAC/LigandDisplayForward?ligandId=5311 (https://www.genenames.org/data/gene‐symbol‐report/#!/hgnc_id/HGNC:11822, http://www.uniprot.org/uniprot/P35625), http://www.guidetopharmacology.org/GRAC/LigandDisplayForward?ligandId=5312 (https://www.genenames.org/data/gene‐symbol‐report/#!/hgnc_id/HGNC:11823, http://www.uniprot.org/uniprot/Q99727)

## http://www.guidetopharmacology.org/GRAC/FamilyDisplayForward?familyId=739

### Overview

ADAM (A Disintegrin And Metalloproteinase domain containing proteins) metalloproteinases cleave cell‐surface or transmembrane proteins to generate soluble and membrane‐limited products.

ADAMTS (with thrombospondin motifs) metalloproteinases cleave cell‐surface or transmembrane proteins to generate soluble and membrane‐limited products.

Information on members of this family may be found in the http://www.guidetopharmacology.org/GRAC/FamilyDisplayForward?familyId=739.

### Comments

Additional ADAM family members include AC123767.2 (cDNA FLJ58962, moderately similar tomouse ADAM3, ENSG00000231168), AL160191.3 (ADAM21‐like protein, http://www.ensembl.org/Homo_sapiens/Gene/Summary?g=ENSG00000235812;r=14:70712470‐70714518), AC136428.3‐2 (ENSG00000185520) and ADAMDEC1 (decysin 1, http://www.ensembl.org/Homo_sapiens/Gene/Summary?g=ENSG00000134028;r=8:24241798‐24263526).

Other ADAMTS family members include AC104758.12‐5 (FLJ00317 protein Fragment ENSG00000231463), AC139425.3‐1 (ENSG00000225577), and AC126339.6‐1 (ENSG00000225734).

## http://www.guidetopharmacology.org/GRAC/FamilyDisplayForward?familyId=748

**Table:** 

Nomenclature	http://www.guidetopharmacology.org/GRAC/ObjectDisplayForward?objectId=1606
HGNC, UniProt	https://www.genenames.org/data/gene‐symbol‐report/#!/hgnc_id/HGNC:3788, http://www.uniprot.org/uniprot/Q04609
EC number	http://www.genome.jp/dbget‐bin/www_bget?ec:3.4.17.21
Antibodies	http://www.guidetopharmacology.org/GRAC/LigandDisplayForward?ligandId=6878 (Binding)
Comments	Folate hydrolase is also known as NAALADase as it is responsible for the hydrolysis of N‐acetaspartylglutamate to form N‐acetylaspartate and L‐glutamate (http://www.guidetopharmacology.org/GRAC/LigandDisplayForward?ligandId=1369). In the gut, the enzyme assists in the assimilation of folate by hydrolysing dietary poly‐gamma‐glutamylfolate. The enzyme is highly expressed in the prostate, and its expression is up‐regulated in cancerous tissue. A tagged version of the antibody http://www.guidetopharmacology.org/GRAC/LigandDisplayForward?ligandId=6878 has been used for imaging purposes.

### Comments

Folate hydrolase is also known as NAALADase as it is responsible for the hydrolysis of N‐acetaspartylglutamate to form N‐acetylaspartate and L‐glutamate. In the gut, the enzyme assists in the assimilation of folate by hydrolysing dietary poly‐gamma‐glutamylfolate. The enzyme is highly expressed in the prostate, and its expression is up‐regulated in cancerous tissue. A tagged version of the antibody http://www.guidetopharmacology.org/GRAC/LigandDisplayForward?ligandId=6878 has been used for imaging purposes.

## http://www.guidetopharmacology.org/GRAC/FamilyDisplayForward?familyId=749

**Table:** 

Nomenclature	http://www.guidetopharmacology.org/GRAC/ObjectDisplayForward?objectId=1393
HGNC, UniProt	https://www.genenames.org/data/gene‐symbol‐report/#!/hgnc_id/HGNC:3002, http://www.uniprot.org/uniprot/P16444
EC number	http://www.genome.jp/dbget‐bin/www_bget?ec:3.4.13.19: http://www.guidetopharmacology.org/GRAC/LigandDisplayForward?ligandId=3353 + H_2_O = http://www.guidetopharmacology.org/GRAC/LigandDisplayForward?ligandId=3352 + http://www.guidetopharmacology.org/GRAC/LigandDisplayForward?ligandId=727
Inhibitors	http://www.guidetopharmacology.org/GRAC/LigandDisplayForward?ligandId=5166 (p*K*_i_ 6) [http://www.ncbi.nlm.nih.gov/pubmed/3495664?dopt=AbstractPlus]

## http://www.guidetopharmacology.org/GRAC/FamilyDisplayForward?familyId=751

**Table:** 

Nomenclature	http://www.guidetopharmacology.org/GRAC/ObjectDisplayForward?objectId=2334	http://www.guidetopharmacology.org/GRAC/ObjectDisplayForward?objectId=2362	http://www.guidetopharmacology.org/GRAC/ObjectDisplayForward?objectId=2359
HGNC, UniProt	https://www.genenames.org/data/gene‐symbol‐report/#!/hgnc_id/HGNC:1246, http://www.uniprot.org/uniprot/P00736	https://www.genenames.org/data/gene‐symbol‐report/#!/hgnc_id/HGNC:3535, http://www.uniprot.org/uniprot/P00734	https://www.genenames.org/data/gene‐symbol‐report/#!/hgnc_id/HGNC:3528, http://www.uniprot.org/uniprot/P00742
EC number	http://www.genome.jp/dbget‐bin/www_bget?ec:3.4.21.41	http://www.genome.jp/dbget‐bin/www_bget?ec:3.4.21.5	http://www.genome.jp/dbget‐bin/www_bget?ec:3.4.21.6
Inhibitors	http://www.guidetopharmacology.org/GRAC/LigandDisplayForward?ligandId=4262 (pIC_50_ 4.9) [http://www.ncbi.nlm.nih.gov/pubmed/9544206?dopt=AbstractPlus]	http://www.guidetopharmacology.org/GRAC/LigandDisplayForward?ligandId=6469 (p*K*_i_ 13) [http://www.ncbi.nlm.nih.gov/pubmed/16363236?dopt=AbstractPlus], http://www.guidetopharmacology.org/GRAC/LigandDisplayForward?ligandId=6458 (p*K*_i_ 12.7) [http://www.ncbi.nlm.nih.gov/pubmed/1894196?dopt=AbstractPlus], http://www.guidetopharmacology.org/GRAC/LigandDisplayForward?ligandId=7727 (p*K*_i_ 9.5) [21], http://www.guidetopharmacology.org/GRAC/LigandDisplayForward?ligandId=6382 (p*K*_i_ 8.7) [http://www.ncbi.nlm.nih.gov/pubmed/9459334?dopt=AbstractPlus], http://www.guidetopharmacology.org/GRAC/LigandDisplayForward?ligandId=6470 (p*K*_i_ 8.6) [http://www.ncbi.nlm.nih.gov/pubmed/1290488?dopt=AbstractPlus], http://www.guidetopharmacology.org/GRAC/LigandDisplayForward?ligandId=6380 (p*K*_i_ 8.3) [http://www.ncbi.nlm.nih.gov/pubmed/11960487?dopt=AbstractPlus], http://www.guidetopharmacology.org/GRAC/LigandDisplayForward?ligandId=6385 (p*K*_i_ 7.7) [http://www.ncbi.nlm.nih.gov/pubmed/9361377?dopt=AbstractPlus]	http://www.guidetopharmacology.org/GRAC/LigandDisplayForward?ligandId=6390 (p*K*_i_ 10.1) [http://www.ncbi.nlm.nih.gov/pubmed/18315548?dopt=AbstractPlus], http://www.guidetopharmacology.org/GRAC/LigandDisplayForward?ligandId=6388 (p*K*_i_ 9.4) [http://www.ncbi.nlm.nih.gov/pubmed/20139357?dopt=AbstractPlus], http://www.guidetopharmacology.org/GRAC/LigandDisplayForward?ligandId=7575 (p*K*_i_ 9.2) [http://www.ncbi.nlm.nih.gov/pubmed/20503967?dopt=AbstractPlus]
Selective inhibitors	–	http://www.guidetopharmacology.org/GRAC/LigandDisplayForward?ligandId=8760 (p*K*_i_ 10.4) [192], http://www.guidetopharmacology.org/GRAC/LigandDisplayForward?ligandId=7688 (pIC_50_ 8.4) [http://www.ncbi.nlm.nih.gov/pubmed/19492147?dopt=AbstractPlus]	–

**Table:** 

Nomenclature	http://www.guidetopharmacology.org/GRAC/ObjectDisplayForward?objectId=2358	http://www.guidetopharmacology.org/GRAC/ObjectDisplayForward?objectId=2394	http://www.guidetopharmacology.org/GRAC/ObjectDisplayForward?objectId=2392	http://www.guidetopharmacology.org/GRAC/ObjectDisplayForward?objectId=2397	http://www.guidetopharmacology.org/GRAC/ObjectDisplayForward?objectId=2424
HGNC, UniProt	https://www.genenames.org/data/gene‐symbol‐report/#!/hgnc_id/HGNC:3309, http://www.uniprot.org/uniprot/P08246	https://www.genenames.org/data/gene‐symbol‐report/#!/hgnc_id/HGNC:9071, http://www.uniprot.org/uniprot/P00747	https://www.genenames.org/data/gene‐symbol‐report/#!/hgnc_id/HGNC:9051, http://www.uniprot.org/uniprot/P00750	https://www.genenames.org/data/gene‐symbol‐report/#!/hgnc_id/HGNC:9475, http://www.uniprot.org/uniprot/P07477	https://www.genenames.org/data/gene‐symbol‐report/#!/hgnc_id/HGNC:12019, http://www.uniprot.org/uniprot/Q15661
EC number	http://www.genome.jp/dbget‐bin/www_bget?ec:3.4.21.37	http://www.genome.jp/dbget‐bin/www_bget?ec:3.4.21.7	http://www.genome.jp/dbget‐bin/www_bget?ec:3.4.21.68	http://www.genome.jp/dbget‐bin/www_bget?ec:3.4.21.4	http://www.genome.jp/dbget‐bin/www_bget?ec:3.4.21.59
Inhibitors	http://www.guidetopharmacology.org/GRAC/LigandDisplayForward?ligandId=6476 (p*K*_i_ 8) [http://www.ncbi.nlm.nih.gov/pubmed/21791628?dopt=AbstractPlus], http://www.guidetopharmacology.org/GRAC/LigandDisplayForward?ligandId=6441 (pIC_50_ 7.4) [http://www.ncbi.nlm.nih.gov/pubmed/21741848?dopt=AbstractPlus]	http://www.guidetopharmacology.org/GRAC/LigandDisplayForward?ligandId=6570 {Bovine} (Binding) (pIC_50_ 6.8) [http://www.ncbi.nlm.nih.gov/pubmed/17666018?dopt=AbstractPlus], http://www.guidetopharmacology.org/GRAC/LigandDisplayForward?ligandId=6573 (Binding) (pIC_50_ 3.6) [http://www.ncbi.nlm.nih.gov/pubmed/17666018?dopt=AbstractPlus]	–	http://www.guidetopharmacology.org/GRAC/LigandDisplayForward?ligandId=4262 (pIC_50_ 7.8) [http://www.ncbi.nlm.nih.gov/pubmed/9544206?dopt=AbstractPlus]	http://www.guidetopharmacology.org/GRAC/LigandDisplayForward?ligandId=4262 (pIC_50_ 10) [http://www.ncbi.nlm.nih.gov/pubmed/12939527?dopt=AbstractPlus]
Selective inhibitors	–	http://www.guidetopharmacology.org/GRAC/LigandDisplayForward?ligandId=6574 (Binding) (pIC_50_ 4.4) [http://www.ncbi.nlm.nih.gov/pubmed/24900876?dopt=AbstractPlus]	–	–	http://www.guidetopharmacology.org/GRAC/LigandDisplayForward?ligandId=7863 (pIC_50_ 8.5) [http://www.ncbi.nlm.nih.gov/pubmed/11172730?dopt=AbstractPlus]

## http://www.guidetopharmacology.org/GRAC/FamilyDisplayForward?familyId=752

### Overview

The T1 macropain beta subunits form the catalytic proteinase core of the 20S proteasome complex [http://www.ncbi.nlm.nih.gov/pubmed/16142822?dopt=AbstractPlus]. This catalytic core enables the degradation of peptides with Arg, Phe, Tyr, Leu, and Glu adjacent to the cleavage site. The β5 subunit is the principal target of the approved drug proteasome inhibitor http://www.guidetopharmacology.org/GRAC/LigandDisplayForward?ligandId=6391.

**Table:** 

Nomenclature	http://www.guidetopharmacology.org/GRAC/ObjectDisplayForward?objectId=2406
HGNC, UniProt	https://www.genenames.org/data/gene‐symbol‐report/#!/hgnc_id/HGNC:9542, http://www.uniprot.org/uniprot/P28074
EC number	http://www.genome.jp/dbget‐bin/www_bget?ec:3.4.25.1
Inhibitors	http://www.guidetopharmacology.org/GRAC/LigandDisplayForward?ligandId=6391 (pIC_50_ 7.7) [http://www.ncbi.nlm.nih.gov/pubmed/19428245?dopt=AbstractPlus]
Selective inhibitors	http://www.guidetopharmacology.org/GRAC/LigandDisplayForward?ligandId=8450 (p*K*_i_ 9) [http://www.ncbi.nlm.nih.gov/pubmed/20160034?dopt=AbstractPlus]

## http://www.guidetopharmacology.org/GRAC/FamilyDisplayForward?familyId=755

### Overview

One member of this family has garnered intense interest as a clinical drug target. As liver PCSK9 acts to maintain cholesterol homeostasis, it has become a target of intense interest for clinical drug development. Inhibition of PCSK9 can lower low‐density cholesterol (LDL‐C) by clearing LDLR‐bound LDL particles, thereby lowering circulating cholesterol levels. It is hypothesised that this action may improve outcomes in patients with atherosclerotic cardiovascular disease [http://www.ncbi.nlm.nih.gov/pubmed/18836590?dopt=AbstractPlus, http://www.ncbi.nlm.nih.gov/pubmed/25083925?dopt=AbstractPlus, http://www.ncbi.nlm.nih.gov/pubmed/19506257?dopt=AbstractPlus]. Therapeutics which inhibit PCSK9 are viewed as potentially lucrative replacements for statins, upon statin patent expiry. Several monoclonal antibodies including http://www.guidetopharmacology.org/GRAC/LigandDisplayForward?ligandId=6744, http://www.guidetopharmacology.org/GRAC/LigandDisplayForward?ligandId=7343, http://www.guidetopharmacology.org/GRAC/LigandDisplayForward?ligandId=7730, RG‐7652 and LY3015014 are under development. One RNAi therapeutic, code named ALN‐PCS02, is also in development [http://www.ncbi.nlm.nih.gov/pubmed/24145894?dopt=AbstractPlus, http://www.ncbi.nlm.nih.gov/pubmed/24094767?dopt=AbstractPlus, http://www.ncbi.nlm.nih.gov/pubmed/18695239?dopt=AbstractPlus].

Information on members of this family may be found in the http://www.guidetopharmacology.org/GRAC/FamilyDisplayForward?familyId=755.

## http://www.guidetopharmacology.org/GRAC/FamilyDisplayForward?familyId=758

**Table:** 

Nomenclature	http://www.guidetopharmacology.org/GRAC/ObjectDisplayForward?objectId=1612
HGNC, UniProt	https://www.genenames.org/data/gene‐symbol‐report/#!/hgnc_id/HGNC:3009, http://www.uniprot.org/uniprot/P27487
EC number	http://www.genome.jp/dbget‐bin/www_bget?ec:3.4.14.5
Endogenous substrates	http://www.guidetopharmacology.org/GRAC/LigandDisplayForward?ligandId=5194 (https://www.genenames.org/data/gene‐symbol‐report/#!/hgnc_id/HGNC:4191, http://www.uniprot.org/uniprot/P01275)
Inhibitors	http://www.guidetopharmacology.org/GRAC/LigandDisplayForward?ligandId=6316 (p*K*_i_ 9.2) [http://www.ncbi.nlm.nih.gov/pubmed/19149538?dopt=AbstractPlus], http://www.guidetopharmacology.org/GRAC/LigandDisplayForward?ligandId=6318 (p*K*_i_ 9) [http://www.ncbi.nlm.nih.gov/pubmed/18052023?dopt=AbstractPlus], http://www.guidetopharmacology.org/GRAC/LigandDisplayForward?ligandId=6286 (pIC_50_ 8.1) [http://www.ncbi.nlm.nih.gov/pubmed/20927248?dopt=AbstractPlus], http://www.guidetopharmacology.org/GRAC/LigandDisplayForward?ligandId=6310 (p*K*_i_ 7.8) [http://www.ncbi.nlm.nih.gov/pubmed/19149538?dopt=AbstractPlus]
Selective inhibitors	http://www.guidetopharmacology.org/GRAC/LigandDisplayForward?ligandId=9627 (Competitive) (p*K*_i_ 8.3) [http://www.ncbi.nlm.nih.gov/pubmed/28452143?dopt=AbstractPlus]

## http://www.guidetopharmacology.org/GRAC/FamilyDisplayForward?familyId=883

### Overview

The Poly ADP‐ribose polymerase family is a series of enzymes, where the best characterised members are nuclear proteins which are thought to function by binding to single strand breaks in DNA, allowing the recruitment of repair enzymes by the synthesis of NAD‐derived ADP‐ribose polymers, which are subsequently degraded by a glycohydrolase (https://www.genenames.org/data/gene‐symbol‐report/#!/hgnc_id/HGNC:8605, http://www.uniprot.org/uniprot/Q86W56).

**Table:** 

Nomenclature	http://www.guidetopharmacology.org/GRAC/ObjectDisplayForward?objectId=2771	http://www.guidetopharmacology.org/GRAC/ObjectDisplayForward?objectId=2772	http://www.guidetopharmacology.org/GRAC/ObjectDisplayForward?objectId=2864
Common abbreviation	PARP1	PARP2	PARP3
HGNC, UniProt	https://www.genenames.org/data/gene‐symbol‐report/#!/hgnc_id/HGNC:270, http://www.uniprot.org/uniprot/P09874	https://www.genenames.org/data/gene‐symbol‐report/#!/hgnc_id/HGNC:272, http://www.uniprot.org/uniprot/Q9UGN5	https://www.genenames.org/data/gene‐symbol‐report/#!/hgnc_id/HGNC:273, http://www.uniprot.org/uniprot/Q9Y6F1
EC number	http://www.genome.jp/dbget‐bin/www_bget?ec:2.4.2.30	http://www.genome.jp/dbget‐bin/www_bget?ec:2.4.2.30	–

### Further reading on Poly ADP‐ribose polymerases

Berger NA *et al*. (2018) Opportunities for the repurposing of PARP inhibitors for the therapy of non‐oncological diseases. *Br J Pharmacol*. **175**: 192‐222 https://www.ncbi.nlm.nih.gov/pubmed/28213892

Faraoni I *et al*. (2019) Targeting ADP‐ribosylation by PARP inhibitors in acute myeloid leukaemia and related disorders. *Biochem Pharmacol* https://www.ncbi.nlm.nih.gov/pubmed/31028744

Zeniou M *et al*. (2019) Therapeutic considerations of PARP in stem cell biology: Relevance in cancer and beyond. *Biochem Pharmacol* https://www.ncbi.nlm.nih.gov/pubmed/31202733

## http://www.guidetopharmacology.org/GRAC/FamilyDisplayForward?familyId=900

### Overview

Hypoxia‐inducible factors (HIFs) are rapidly‐responding sensors of reductions in local oxygen tensions, prompting changes in gene transcription. Listed here are the 4‐prolyl hydroxylase family, members of which have been identified to hydroxylate proline residues in HIF1α (https://www.genenames.org/data/gene‐symbol‐report/#!/hgnc_id/HGNC:4910; https://www.uniprot.org/uniprot/Q16665) leading to an increased degradation through proteasomal hydrolysis. This action requires molecular oxygen and 2‐oxoglutarate, and so reduced oxygen tensions prevents HIF1α hydroxylation, allowing its translocation to the nucleus and dimerisation with HIF1β (also known as https://www.genenames.org/data/gene‐symbol‐report/#!/hgnc_id/HGNC:700; https://www.uniprot.org/uniprot/P27540), thereby allowing interaction with the genome as a transcription factor.

**Table:** 

Nomenclature	http://www.guidetopharmacology.org/GRAC/ObjectDisplayForward?objectId=2832	http://www.guidetopharmacology.org/GRAC/ObjectDisplayForward?objectId=2833	http://www.guidetopharmacology.org/GRAC/ObjectDisplayForward?objectId=2834
Common abbreviation	PHD1	PHD2	PHD3
HGNC, UniProt	https://www.genenames.org/data/gene‐symbol‐report/#!/hgnc_id/HGNC:14660, http://www.uniprot.org/uniprot/Q96KS0	https://www.genenames.org/data/gene‐symbol‐report/#!/hgnc_id/HGNC:1232, http://www.uniprot.org/uniprot/Q9GZT9	https://www.genenames.org/data/gene‐symbol‐report/#!/hgnc_id/HGNC:14661, http://www.uniprot.org/uniprot/Q9H6Z9
EC number	http://www.genome.jp/dbget‐bin/www_bget?ec:1.14.11.29	http://www.genome.jp/dbget‐bin/www_bget?ec:1.14.11.29	http://www.genome.jp/dbget‐bin/www_bget?ec:1.14.11.29

### Further reading on Prolyl hydroxylases

Joharapurkar AA *et al*. (2018) Prolyl Hydroxylase Inhibitors: A Breakthrough in the Therapy of Anemia Associated with Chronic Diseases. *J Med Chem* **61**: 6964‐6982 https://www.ncbi.nlm.nih.gov/pubmed/29712435

Lanigan SM and O’Connor JJ. (2019) Prolyl hydroxylase domain inhibitors: can multiple mechanisms be an opportunity for ischemic stroke? *Neuropharmacology* **148**: 117‐130 https://www.ncbi.nlm.nih.gov/pubmed/30578795

Singh L *et al*. (2018) Prolyl hydroxylase 2: a promising target to inhibit hypoxia‐induced cellular metabolism in cancer cells. *Drug Discov Today* **23**: 1873‐1882 https://www.ncbi.nlm.nih.gov/pubmed/29772209

Vasta JD and Raines RT *et al*. (2018) Collagen Prolyl 4‐Hydroxylase as a Therapeutic Target. *J Med Chem* **61**: 10403‐10411 https://www.ncbi.nlm.nih.gov/pubmed/29986141

Watts ER and Walmsley SR. (2019) Inflammation and Hypoxia: HIF and PHD Isoform Selectivity. *Trends Mol Med* **25**: 33‐46 https://www.ncbi.nlm.nih.gov/pubmed/30442494

## http://www.guidetopharmacology.org/GRAC/FamilyDisplayForward?familyId=776

### Overview

S1P (http://www.guidetopharmacology.org/GRAC/LigandDisplayForward?ligandId=911) is a bioactive lipid which, after release from cells via certain transporters, acts as a ligand for a family of five S1P‐specific G protein‐coupled receptors (S1P1‐5). However, it also has a number of intracellular targets. S1P is formed by the ATP‐dependent phosphorylation of sphingosine, catalysed by two isoforms of sphingosine kinase (EC 2.7.1.91). It can be dephosphorylated back to sphingosine by sphingosine 1‐phosphate phosphatase (EC 3.1.3) or cleaved into phosphoethanolamine and hexadecenal by sphingosine 1‐phosphate lyase (EC 4.1.2.27). Recessive mutations in the S1P lyase (SPL) gene underlie a recently identified sphingolipidosis: SPL Insufficiency Syndrome (SPLIS). In general, S1P promotes cell survival, proliferation, migration, adhesion and inhibition of apoptosis. Intracellular S1P affects epigenetic regulation, endosomal processing, mitochondrial function and cell proliferation/senescence. S1P has myriad physiological functions, including vascular development, lymphocyte trafficking and neurogenesis. However, S1P is also involved in a number of diseases such as cancer, inflammation and fibrosis. Therefore, its GPCRs and enzymes of synthesis and degradation are a major focus for drug discovery.

## http://www.guidetopharmacology.org/GRAC/FamilyDisplayForward?familyId=777

### Overview

SPHK1 and SPHK2 are encoded by different genes with some redundancy of function; genetic deletion of both Sphk1 and Sphk2, but not either alone, is embryonic lethal in mice. There are splice variants of each isoform (SphK1a‐c and SphK2a, b), distinguished by their N‐terminal sequences. SPHK1 and SPHK2 differ in tissue distribution, sub‐cellular localisation, biochemical properties and regulation. They regulate discrete pools of S1P. Receptor stimulation induces SPHK1 translocation from the cytoplasm to the plasma membrane. SPHK1 translocation is regulated by phosphorylation/dephosphorylation, specific protein:protein interactions and interaction with specific lipids at the plasma membrane. SPHK1 is a dimeric protein, as confirmed by its crystal structure which forms a positive cluster, between protomers, essential for interaction with anionic phospholipids in the plasma membrane. SPHK2 is localised to the ER or associated with mitochondria or shuttles in/out of the nucleus, regulated by phosphorylation. Intracellular targets of nuclear S1P include the catalytic subunit of telomerase (TERT) and regulators of gene expression including histone deacetylases (HDAC 1/2) and peroxisome proliferator‐activated receptor gamma (PPARγ). SPHK2 phosphorylates the pro‐drug FTY720 (http://www.guidetopharmacology.org/GRAC/LigandDisplayForward?ligandId=2407, which is used to treat some forms of multiple sclerosis) to a mimic of S1P and that acts as a functional antagonist of S1P_1_ receptors. Inhibitors of SPHK1 and SPHK2 have therapeutic potential in many diseases.

**Table:** 

Nomenclature	http://www.guidetopharmacology.org/GRAC/ObjectDisplayForward?objectId=2204	http://www.guidetopharmacology.org/GRAC/ObjectDisplayForward?objectId=2205
Common abbreviation	SPHK1	SPHK2
HGNC, UniProt	https://www.genenames.org/data/gene‐symbol‐report/#!/hgnc_id/HGNC:11240, http://www.uniprot.org/uniprot/Q9NYA1	https://www.genenames.org/data/gene‐symbol‐report/#!/hgnc_id/HGNC:18859, http://www.uniprot.org/uniprot/Q9NRA0
EC number	http://www.genome.jp/dbget‐bin/www_bget?ec:2.7.1.91 + http://www.guidetopharmacology.org/GRAC/LigandDisplayForward?ligandId=1713 = http://www.guidetopharmacology.org/GRAC/LigandDisplayForward?ligandId=911 + http://www.guidetopharmacology.org/GRAC/LigandDisplayForward?ligandId=1712 dihydrosphingosine + http://www.guidetopharmacology.org/GRAC/LigandDisplayForward?ligandId=1713 = http://www.guidetopharmacology.org/GRAC/LigandDisplayForward?ligandId=911 + http://www.guidetopharmacology.org/GRAC/LigandDisplayForward?ligandId=1712	http://www.genome.jp/dbget‐bin/www_bget?ec:2.7.1.91: http://www.guidetopharmacology.org/GRAC/LigandDisplayForward?ligandId=2452 + http://www.guidetopharmacology.org/GRAC/LigandDisplayForward?ligandId=1713 = http://www.guidetopharmacology.org/GRAC/LigandDisplayForward?ligandId=911 + http://www.guidetopharmacology.org/GRAC/LigandDisplayForward?ligandId=1712 dihydrosphingosine + http://www.guidetopharmacology.org/GRAC/LigandDisplayForward?ligandId=1713 = http://www.guidetopharmacology.org/GRAC/LigandDisplayForward?ligandId=911 + http://www.guidetopharmacology.org/GRAC/LigandDisplayForward?ligandId=1712
Cofactors	http://www.guidetopharmacology.org/GRAC/LigandDisplayForward?ligandId=708 [http://www.ncbi.nlm.nih.gov/pubmed/22677141?dopt=AbstractPlus]	http://www.guidetopharmacology.org/GRAC/LigandDisplayForward?ligandId=708
Inhibitors	http://www.guidetopharmacology.org/GRAC/LigandDisplayForward?ligandId=6041 (p*K*_i_ 4.8) [http://www.ncbi.nlm.nih.gov/pubmed/20061445?dopt=AbstractPlus], http://www.guidetopharmacology.org/GRAC/LigandDisplayForward?ligandId=10219 (pIC_50_ 4.6) [http://www.ncbi.nlm.nih.gov/pubmed/25788259?dopt=AbstractPlus]	http://www.guidetopharmacology.org/GRAC/LigandDisplayForward?ligandId=10219 (p*K*_i_ 5.2) [http://www.ncbi.nlm.nih.gov/pubmed/25788259?dopt=AbstractPlus], http://www.guidetopharmacology.org/GRAC/LigandDisplayForward?ligandId=6041 (p*K*_i_ 5.1) [http://www.ncbi.nlm.nih.gov/pubmed/22970244?dopt=AbstractPlus]
Selective inhibitors	http://www.guidetopharmacology.org/GRAC/LigandDisplayForward?ligandId=6623 (p*K*_i_ 8.4) [http://www.ncbi.nlm.nih.gov/pubmed/22397330?dopt=AbstractPlus]	http://www.guidetopharmacology.org/GRAC/LigandDisplayForward?ligandId=10217 (p*K*_i_ 7.1) [http://www.ncbi.nlm.nih.gov/pubmed/28406646?dopt=AbstractPlus], http://www.guidetopharmacology.org/GRAC/LigandDisplayForward?ligandId=10218 (pIC_50_ 6.8) [http://www.ncbi.nlm.nih.gov/pubmed/28231433?dopt=AbstractPlus], http://www.guidetopharmacology.org/GRAC/LigandDisplayForward?ligandId=6624 (p*K*_i_ 5) [http://www.ncbi.nlm.nih.gov/pubmed/20061445?dopt=AbstractPlus], http://www.guidetopharmacology.org/GRAC/LigandDisplayForward?ligandId=6625 (p*K*_i_ 4.8) [http://www.ncbi.nlm.nih.gov/pubmed/21620961?dopt=AbstractPlus]
Comments	SK1 inhibitors induce its proteasomal degradation [http://www.ncbi.nlm.nih.gov/pubmed/20926375?dopt=AbstractPlus, http://www.ncbi.nlm.nih.gov/pubmed/26934645?dopt=AbstractPlus]. SK1 crystal structures confirm that it is dimeric [http://www.ncbi.nlm.nih.gov/pubmed/27021309?dopt=AbstractPlus]; there is no crystal structure available for SK2.	There is no crystal structure available for SK2.

### Comments

http://www.guidetopharmacology.org/GRAC/LigandDisplayForward?ligandId=10219 is competitive with ATP; other SPHK inhibitors are competitive with sphingosine. ABC294640 (http://www.guidetopharmacology.org/GRAC/LigandDisplayForward?ligandId=6624) has known off‐target effects on dihydroceramide desaturase (*DEGS1*) [http://www.ncbi.nlm.nih.gov/pubmed/26934645?dopt=AbstractPlus, http://www.ncbi.nlm.nih.gov/pubmed/26494858?dopt=AbstractPlus]) and induces proteasomal degradation of SK1 [http://www.ncbi.nlm.nih.gov/pubmed/26934645?dopt=AbstractPlus]. ABC294640 is in clinical trials for advanced cholangiocarcinoma, advanced hepatocellular carcinoma and refractory/relapsed multiple myeloma (to view ClinicalTrials.gov list click https://clinicaltrials.gov/ct2/results?cond=&term=ABC294640).

### Further reading on Sphingosine kinase

Adams DR *et al*. (2016) Sphingosine Kinases: Emerging Structure‐Function Insights. *Trends Biochem. Sci*. **41**: 395‐409 https://www.ncbi.nlm.nih.gov/pubmed/27021309?dopt=AbstractPlus

Lynch KR *et al*. (2016) Sphingosine kinase inhibitors: a review of patent literature (2006‐2015). *Expert Opin Ther Pat* **26**: 1409‐1416 https://www.ncbi.nlm.nih.gov/pubmed/27539678?dopt=AbstractPlus

Pitman MR *et al*. (2016) Recent advances in the development of sphingosine kinase inhibitors. *Cell. Signal*. **28**: 1349‐63 https://www.ncbi.nlm.nih.gov/pubmed/27297359?dopt=AbstractPlus

Pulkoski‐Gross MJ *et al*. (2018) An intrinsic lipid‐binding interface controls sphingosine kinase 1 function. *J. Lipid Res*. **59**: 462‐474 https://www.ncbi.nlm.nih.gov/pubmed/29326159?dopt=AbstractPlus

Pyne NJ *et al*. (2017) Sphingosine Kinase 2 in Autoimmune/Inflammatory Disease and the Development of Sphingosine Kinase 2 Inhibitors. *Trends Pharmacol. Sci*. **38**: 581‐591 https://www.ncbi.nlm.nih.gov/pubmed/28606480?dopt=AbstractPlus

Pyne S *et al*. (2018) Sphingosine Kinases as Druggable Targets. *Handb Exp Pharmacol* https://www.ncbi.nlm.nih.gov/pubmed/29460151?dopt=AbstractPlus

## http://www.guidetopharmacology.org/GRAC/FamilyDisplayForward?familyId=778

**Table:** 

Nomenclature	http://www.guidetopharmacology.org/GRAC/ObjectDisplayForward?objectId=2523	http://www.guidetopharmacology.org/GRAC/ObjectDisplayForward?objectId=2524
Common abbreviation	SGPP1	SGPP2
HGNC, UniProt	https://www.genenames.org/data/gene‐symbol‐report/#!/hgnc_id/HGNC:17720, http://www.uniprot.org/uniprot/Q9BX95	https://www.genenames.org/data/gene‐symbol‐report/#!/hgnc_id/HGNC:19953, http://www.uniprot.org/uniprot/Q8IWX5
EC number	http://www.genome.jp/dbget‐bin/www_bget?ec:3.1.3.‐: http://www.guidetopharmacology.org/GRAC/LigandDisplayForward?ligandId=911 ‐> http://www.guidetopharmacology.org/GRAC/LigandDisplayForward?ligandId=2452 + inorganic phosphate	http://www.genome.jp/dbget‐bin/www_bget?ec:3.1.3.‐: http://www.guidetopharmacology.org/GRAC/LigandDisplayForward?ligandId=911 ‐> http://www.guidetopharmacology.org/GRAC/LigandDisplayForward?ligandId=2452 + inorganic phosphate
Comments	Depletion of S1P phosphohydrolase‐1 (SPP1), which degrades intracellular S1P, induces the unfolded protein response and endoplasmic reticulum stress‐induced autophagy [http://www.ncbi.nlm.nih.gov/pubmed/22052905?dopt=AbstractPlus].	–

### Comments

SGPP1 and SGPP2 are non‐redundant endoplasmic reticulum enzymes that dephosphorylate intracellular http://www.guidetopharmacology.org/GRAC/LigandDisplayForward?ligandId=911. The phenotype of *Sgpp1*(‐/‐) mice differ with genetic background. *Sgpp2*(‐/‐) mice are also available. No specific SGPP inhibitors available [http://www.ncbi.nlm.nih.gov/pubmed/22052905?dopt=AbstractPlus].

### Further reading on Sphingosine 1‐phosphate phosphatase

Allende ML *et al*. (2013) Sphingosine‐1‐phosphate phosphatase 1 regulates keratinocyte differentiation and epidermal homeostasis. *J. Biol. Chem*. **288**: 18381‐91 https://www.ncbi.nlm.nih.gov/pubmed/23637227?dopt=AbstractPlus

Huang WC *et al*. (2016) Sphingosine‐1‐phosphate phosphatase 2 promotes disruption of mucosal integrity, and contributes to ulcerative colitis in mice and humans. *FASEB J*. **30**: 2945‐58 https://www.ncbi.nlm.nih.gov/pubmed/27130484?dopt=AbstractPlus

Lépine S *et al*. (2011) Sphingosine‐1‐phosphate phosphohydrolase‐1 regulates ER stress‐induced autophagy. *Cell Death Differ*. **18**: 350‐61 https://www.ncbi.nlm.nih.gov/pubmed/20798685?dopt=AbstractPlus

Mandala SM *et al*. (2000) Molecular cloning and characterization of a lipid phosphohydrolase that degrades sphingosine‐1‐ phosphate and induces cell death. *Proc. Natl. Acad. Sci. U.S.A*. **97**: 7859‐64 https://www.ncbi.nlm.nih.gov/pubmed/10859351?dopt=AbstractPlus

Taguchi Y *et al*. (2016) Sphingosine‐1‐phosphate Phosphatase 2 Regulates Pancreatic Islet β‐Cell Endoplasmic Reticulum Stress and Proliferation. *J. Biol. Chem*. **291**: 12029‐38 https://www.ncbi.nlm.nih.gov/pubmed/27059959?dopt=AbstractPlus

## http://www.guidetopharmacology.org/GRAC/FamilyDisplayForward?familyId=777

**Table:** 

Nomenclature	http://www.guidetopharmacology.org/GRAC/ObjectDisplayForward?objectId=2522
HGNC, UniProt	https://www.genenames.org/data/gene‐symbol‐report/#!/hgnc_id/HGNC:10817, http://www.uniprot.org/uniprot/O95470
EC number	http://www.genome.jp/dbget‐bin/www_bget?ec:4.1.2.27: http://www.guidetopharmacology.org/GRAC/LigandDisplayForward?ligandId=911 ‐> http://www.guidetopharmacology.org/GRAC/LigandDisplayForward?ligandId=6628 + hexadecenal http://www.guidetopharmacology.org/GRAC/LigandDisplayForward?ligandId=2921 ‐> http://www.guidetopharmacology.org/GRAC/LigandDisplayForward?ligandId=6628 + http://www.guidetopharmacology.org/GRAC/LigandDisplayForward?ligandId=6627
Cofactors	http://www.guidetopharmacology.org/GRAC/LigandDisplayForward?ligandId=5249
Inhibitors	http://www.guidetopharmacology.org/GRAC/LigandDisplayForward?ligandId=8465 (pIC_50_ 6.7) [http://www.ncbi.nlm.nih.gov/pubmed/27519818?dopt=AbstractPlus, http://www.ncbi.nlm.nih.gov/pubmed/23499842?dopt=AbstractPlus, http://www.ncbi.nlm.nih.gov/pubmed/25630683?dopt=AbstractPlus, http://www.ncbi.nlm.nih.gov/pubmed/24809814?dopt=AbstractPlus]

### Comments

http://www.guidetopharmacology.org/GRAC/LigandDisplayForward?ligandId=6626 (2‐Acetyl‐5‐tetrahydroxybutyl imidazole) inhibits the enzyme activity in intact cell preparations [http://www.ncbi.nlm.nih.gov/pubmed/16151014?dopt=AbstractPlus]. Recessive mutations in the S1P lyase (*SGPL1*) gene underlie a recently identified sphingolipidosis: SPL Insufficiency Syndrome (SPLIS) [http://www.ncbi.nlm.nih.gov/pubmed/30274713?dopt=AbstractPlus]. A Phase 2 clinical trial of LX3305 (http://www.guidetopharmacology.org/GRAC/LigandDisplayForward?ligandId=9851) for rheumatoid arthritis has been completed (see https://clinicaltrials.gov/ct2/show/NCT00903383).

### Further reading on Sphingosine 1‐phosphate lyase

Bamborschke D *et al*. (2018) A novel mutation in sphingosine‐1‐phosphate lyase causing congenital brain malformation. *Brain Dev*. **40**: 480‐483 https://www.ncbi.nlm.nih.gov/pubmed/29501407?dopt=AbstractPlus

Choi YJ *et al*. (2019) Sphingosine phosphate lyase insufficiency syndrome (SPLIS): A novel inborn error of sphingolipid metabolism. *Adv Biol Regul* **71**: 128‐140 https://www.ncbi.nlm.nih.gov/pubmed/30274713

Lovric S *et al*. (2017) Mutations in sphingosine‐1‐phosphate lyase cause nephrosis with ichthyosis and adrenal insufficiency. *J. Clin. Invest*. **127**: 912‐928 https://www.ncbi.nlm.nih.gov/pubmed/28165339?dopt=AbstractPlus

Prasad R *et al*. (2017) Sphingosine‐1‐phosphate lyase mutations cause primary adrenal insufficiency and steroid‐resistant nephrotic syndrome. *J. Clin. Invest*. **127**: 942‐953 https://www.ncbi.nlm.nih.gov/pubmed/28165343?dopt=AbstractPlus

## http://www.guidetopharmacology.org/GRAC/FamilyDisplayForward?familyId=779

### Overview

The thyroid hormones triiodothyronine and thyroxine, usually abbreviated as http://www.guidetopharmacology.org/GRAC/LigandDisplayForward?ligandId=2634 and http://www.guidetopharmacology.org/GRAC/LigandDisplayForward?ligandId=2635, respectively, are synthesized in the thyroid gland by sequential metabolism of tyrosine residues in the glycosylated homodimeric protein thyroglobulin (https://www.genenames.org/data/gene‐symbol‐report/#!/hgnc_id/HGNC:11764, http://www.uniprot.org/uniprot/P01266) under the influence of the haem‐containing protein iodide peroxidase. Iodide peroxidase/TPO is a haem‐containing enzyme, from the same structural family as eosinophil peroxidase (https://www.genenames.org/data/gene‐symbol‐report/#!/hgnc_id/HGNC:3423, http://www.uniprot.org/uniprot/P11678), lactoperoxidase (https://www.genenames.org/data/gene‐symbol‐report/#!/hgnc_id/HGNC:6678, http://www.uniprot.org/uniprot/P22079) and myeloperoxidase (https://www.genenames.org/data/gene‐symbol‐report/#!/hgnc_id/HGNC:7218, http://www.uniprot.org/uniprot/P05164). Circulating thyroid hormone is bound to thyroxine‐binding globulin (https://www.genenames.org/data/gene‐symbol‐report/#!/hgnc_id/HGNC:11583, http://www.uniprot.org/uniprot/P05543).

### Tissue deiodinases

These are 1 TM selenoproteins that remove an iodine from http://www.guidetopharmacology.org/GRAC/LigandDisplayForward?ligandId=2635 (3,3′,5,5′‐tetraiodothyronine) to generate http://www.guidetopharmacology.org/GRAC/LigandDisplayForward?ligandId=2634 (3,3′,5‐triiodothyronine, a more potent agonist at thyroid hormone receptors) or http://www.guidetopharmacology.org/GRAC/LigandDisplayForward?ligandId=2636 (rT3, 3,3′,5′‐triiodothyronine, a relatively inactive analogue). DIO1 is also able to deiodinate RT_3_ to form 3,3′‐diidothyronine (http://www.guidetopharmacology.org/GRAC/LigandDisplayForward?ligandId=6648). Iodotyrosine deiodinase is a 1TM homodimeric enzyme.

**Table:** 

Nomenclature	http://www.guidetopharmacology.org/GRAC/ObjectDisplayForward?objectId=2526	http://www.guidetopharmacology.org/GRAC/ObjectDisplayForward?objectId=2481	http://www.guidetopharmacology.org/GRAC/ObjectDisplayForward?objectId=2482	http://www.guidetopharmacology.org/GRAC/ObjectDisplayForward?objectId=2483	http://www.guidetopharmacology.org/GRAC/ObjectDisplayForward?objectId=2488
Common abbreviation	TPO	DIO1	DIO2	DIO3	IYD
HGNC, UniProt	https://www.genenames.org/data/gene‐symbol‐report/#!/hgnc_id/HGNC:12015, http://www.uniprot.org/uniprot/P07202	https://www.genenames.org/data/gene‐symbol‐report/#!/hgnc_id/HGNC:2883, http://www.uniprot.org/uniprot/P49895	https://www.genenames.org/data/gene‐symbol‐report/#!/hgnc_id/HGNC:2884, http://www.uniprot.org/uniprot/Q92813	https://www.genenames.org/data/gene‐symbol‐report/#!/hgnc_id/HGNC:2885, http://www.uniprot.org/uniprot/P55073	https://www.genenames.org/data/gene‐symbol‐report/#!/hgnc_id/HGNC:21071, http://www.uniprot.org/uniprot/Q6PHW0
EC number	http://www.genome.jp/dbget‐bin/www_bget?ec:1.11.1.8: [Thyroglobulin]‐L‐tyrosine + http://www.genome.jp/dbget‐bin/www_bget?ec:1.97.1.10 + H^+^ + I^‐^ ‐> [Thyroglobulin]‐3,5,3′‐triiodo‐L‐thyronine + [thyroglobulin]‐aminoacrylate + H_2_O	http://www.genome.jp/dbget‐bin/www_bget?ec:1.97.1.10: http://www.guidetopharmacology.org/GRAC/LigandDisplayForward?ligandId=2635 ‐> http://www.guidetopharmacology.org/GRAC/LigandDisplayForward?ligandId=2634 http://www.guidetopharmacology.org/GRAC/LigandDisplayForward?ligandId=2636 ‐> http://www.guidetopharmacology.org/GRAC/LigandDisplayForward?ligandId=6648	http://www.genome.jp/dbget‐bin/www_bget?ec:1.97.1.10: http://www.guidetopharmacology.org/GRAC/LigandDisplayForward?ligandId=2635 ‐> http://www.guidetopharmacology.org/GRAC/LigandDisplayForward?ligandId=2634 http://www.guidetopharmacology.org/GRAC/LigandDisplayForward?ligandId=2636 ‐> http://www.guidetopharmacology.org/GRAC/LigandDisplayForward?ligandId=6648	http://www.genome.jp/dbget‐bin/www_bget?ec:1.97.1.11: http://www.guidetopharmacology.org/GRAC/LigandDisplayForward?ligandId=2635 ‐> http://www.guidetopharmacology.org/GRAC/LigandDisplayForward?ligandId=2634 http://www.guidetopharmacology.org/GRAC/LigandDisplayForward?ligandId=2636 ‐> http://www.guidetopharmacology.org/GRAC/LigandDisplayForward?ligandId=6648	http://www.genome.jp/dbget‐bin/www_bget?ec:1.22.1.1: http://www.guidetopharmacology.org/GRAC/LigandDisplayForward?ligandId=5117 ‐> http://www.guidetopharmacology.org/GRAC/LigandDisplayForward?ligandId=4791 + I^‐^ http://www.guidetopharmacology.org/GRAC/LigandDisplayForward?ligandId=6651 ‐> http://www.guidetopharmacology.org/GRAC/LigandDisplayForward?ligandId=5117 + I^‐^
Cofactors	http://www.guidetopharmacology.org/GRAC/LigandDisplayForward?ligandId=707	–	–	–	http://www.guidetopharmacology.org/GRAC/LigandDisplayForward?ligandId=5184, http://www.guidetopharmacology.org/GRAC/LigandDisplayForward?ligandId=3041
Inhibitors	http://www.guidetopharmacology.org/GRAC/LigandDisplayForward?ligandId=6649 [http://www.ncbi.nlm.nih.gov/pubmed/748042?dopt=AbstractPlus], http://www.guidetopharmacology.org/GRAC/LigandDisplayForward?ligandId=6650 [http://www.ncbi.nlm.nih.gov/pubmed/748042?dopt=AbstractPlus]	–	–	–	–
Comments	Carbimazole is a pro‐drug for http://www.guidetopharmacology.org/GRAC/LigandDisplayForward?ligandId=6649	–	–	–	–

### Further reading on Thyroid hormone turnover

Darras VM *et al*. (2015) Intracellular thyroid hormone metabolism as a local regulator of nuclear thyroid hormone receptor‐mediated impact on vertebrate development. *Biochim. Biophys. Acta* **1849**: 130‐41 https://www.ncbi.nlm.nih.gov/pubmed/24844179?dopt=AbstractPlus

Gereben B *et al*. (2015) Scope and limitations of iodothyronine deiodinases in hypothyroidism. *Nat Rev Endocrinol* **11**: 642‐652 https://www.ncbi.nlm.nih.gov/pubmed/26416219?dopt=AbstractPlus

Mondal S *et al*. (2017) Novel thyroid hormone analogues, enzyme inhibitors andmimetics, and their action. *Mol. Cell. Endocrinol*. **458**: 91‐104 https://www.ncbi.nlm.nih.gov/pubmed/28408161?dopt=AbstractPlus

Schweizer U *et al*. (2015) New insights into the structure and mechanism of iodothyronine deiodinases. *J. Mol. Endocrinol*. **55**: R37‐52 https://www.ncbi.nlm.nih.gov/pubmed/26390881?dopt=AbstractPlus

van der Spek AH *et al*. (2017) Thyroid hormone metabolism in innate immune cells. *J. Endocrinol*. **232**: R67‐R81 https://www.ncbi.nlm.nih.gov/pubmed/27852725?dopt=AbstractPlus

## http://www.guidetopharmacology.org/GRAC/FamilyDisplayForward?familyId=923

**Table:** 

Nomenclature	http://www.guidetopharmacology.org/GRAC/ObjectDisplayForward?objectId=2886
HGNC, UniProt	https://www.genenames.org/data/gene‐symbol‐report/#!/hgnc_id/HGNC:6381, http://www.uniprot.org/uniprot/O15229
EC number	http://www.genome.jp/dbget‐bin/www_bget?ec:1.14.13.9 L‐kynurenine + NADPH + O_2_ <=> 3‐hydroxy‐L‐kynurenine + NADP(+) + H_2_O
Comments	Kynurenine 3‐monooxygenase participates in metabolism of the essential amino acid tryptophan.

### Further reading on 1.14.13.9 Kynurenine 3‐monooxygenase

Dounay AB *et al*. (2015) Challenges and Opportunities in the Discovery of New Therapeutics Targeting the Kynurenine Pathway. *J. Med. Chem*. **58**: 8762‐82 https://www.ncbi.nlm.nih.gov/pubmed/26207924?dopt=AbstractPlus

Erhardt S *et al*. (2017) The kynurenine pathway in schizophrenia and bipolar disorder. *Neuropharmacology* **112**: 297‐306 https://www.ncbi.nlm.nih.gov/pubmed/27245499?dopt=AbstractPlus

Fujigaki H *et al*. (2017) L‐Tryptophan‐kynurenine pathway enzymes are therapeutic target for neuropsychiatric diseases: Focus on cell type differences. *Neuropharmacology* **112**: 264‐274 https://www.ncbi.nlm.nih.gov/pubmed/26767951?dopt=AbstractPlus

Smith JR *et al*. (2016) Kynurenine‐3‐monooxygenase: a review of structure, mechanism, and inhibitors. *Drug Discov. Today* **21**: 315‐24 https://www.ncbi.nlm.nih.gov/pubmed/26589832?dopt=AbstractPlus

Song P *et al*. (2017) Abnormal kynurenine pathway of tryptophan catabolism in cardiovascular diseases. *Cell. Mol. Life Sci*. **74**: 2899‐2916 https://www.ncbi.nlm.nih.gov/pubmed/28314892?dopt=AbstractPlus

## http://www.guidetopharmacology.org/GRAC/FamilyDisplayForward?familyId=898

### Overview

Farnesyltransferase is a member of the prenyltransferases family which also includes geranylgeranyltransferase types I (EC 2.5.1.59) and II (EC 2.5.1.60) [http://www.ncbi.nlm.nih.gov/pubmed/8621375?dopt=AbstractPlus]. Protein farnesyltransferase catalyses the post‐translational formation of a thioether linkage between the C‐1 of an isoprenyl group and a cysteine residue fourth from the C‐terminus of a protein (*ie* to the CaaX motif, where ’a’ is an aliphatic amino acid and ’X’ is usually serine, methionine, alanine or glutamine; leucine for EC 2.5.1.59) [http://www.ncbi.nlm.nih.gov/pubmed/7756316?dopt=AbstractPlus]. Farnesyltransferase is a dimer, composed of an alpha and beta subunit and requires Mg^2+^ and Zn^2+^ ions as cofactors. The active site is located between the subunits. Prenylation creates a hydrophobic domain on protein tails which acts as a membrane anchor.

Substrates of the prenyltransferases include Ras, Rho, Rab, other Ras‐related small GTP‐binding proteins, G‐protein γ‐subunits, nuclear lamins, centromeric proteins and many proteins involved in visual signal transduction.

In relation to the causative association between oncogenic Ras proteins and cancer, farnesyltransferase has become an important mechanistic drug discovery target.

Information on members of this family may be found in the http://www.guidetopharmacology.org/GRAC/FamilyDisplayForward?familyId=898.

### Further reading on 2.5.1.58 Protein farnesyltransferase

Gao S *et al*. (2016) The Role of Geranylgeranyltransferase I‐Mediated Protein Prenylation in the Brain. *Mol. Neurobiol*. **53**: 6925‐6937 https://www.ncbi.nlm.nih.gov/pubmed/26666664?dopt=AbstractPlus

Shen M *et al*. (2015) Farnesyltransferase and geranylgeranyltransferase I: structures, mechanism, inhibitors and molecular modeling. *Drug Discov. Today* **20**: 267‐76 https://www.ncbi.nlm.nih.gov/pubmed/25450772?dopt=AbstractPlus

Shen Y *et al*. (2015) The Recent Development of Farnesyltransferase Inhibitors as Anticancer and Antimalarial Agents. *Mini Rev Med Chem* **15**: 837‐57 https://www.ncbi.nlm.nih.gov/pubmed/25963569?dopt=AbstractPlus

Wang M *et al*. (2016) Protein prenylation: unique fats make their mark on biology. *Nat. Rev. Mol. Cell Biol*. **17**: 110‐22 https://www.ncbi.nlm.nih.gov/pubmed/26790532?dopt=AbstractPlus

## http://www.guidetopharmacology.org/GRAC/FamilyDisplayForward?familyId=848

### Overview

Histone deacetylases act as erasers of epigenetic acetylation marks on lysine residues in histones. Removal of the acetyl groups facilitates tighter packing of chromatin (heterochromatin formation) leading to transcriptional repression.

The histone deacetylase family has been classified in to five subfamilies based on phylogenetic comparison with yeast homologues:

Class I contains HDACs 1, 2, 3 and 8

Class IIa contains HDACs 4, 5, 7 and 9

Class IIb contains HDACs 6 and 10

Class III contains the sirtuins (SIRT1‐7)

Class IV contains only HDAC11.

Classes I, II and IV use Zn^+^ as a co‐factor, whereas catalysis by Class III enzymes requires NAD^+^ as a co‐factor, and members of this subfamily have ADP‐ribosylase activity in addition to protein deacetylase function [http://www.ncbi.nlm.nih.gov/pubmed/20132909?dopt=AbstractPlus].

HDACs have more general protein deacetylase activity, being able to deacetylate lysine residues in non‐histone proteins [http://www.ncbi.nlm.nih.gov/pubmed/19608861?dopt=AbstractPlus] such as microtubules [http://www.ncbi.nlm.nih.gov/pubmed/12024216?dopt=AbstractPlus], the hsp90 chaperone [http://www.ncbi.nlm.nih.gov/pubmed/15916966?dopt=AbstractPlus] and the tumour suppressor p53 [http://www.ncbi.nlm.nih.gov/pubmed/11099047?dopt=AbstractPlus].

Dysregulated HDACactivity has been identified in cancer cells and tumour tissues [http://www.ncbi.nlm.nih.gov/pubmed/11704848?dopt=AbstractPlus, http://www.ncbi.nlm.nih.gov/pubmed/19383284?dopt=AbstractPlus], making HDACs attractive molecular targets in the search for novel mechanisms to treat cancer [http://www.ncbi.nlm.nih.gov/pubmed/24382387?dopt=AbstractPlus]. Several small molecule HDAC inhibitors are already approved for clinical use: http://www.guidetopharmacology.org/GRAC/LigandDisplayForward?ligandId=7006, http://www.guidetopharmacology.org/GRAC/LigandDisplayForward?ligandId=7496, http://www.guidetopharmacology.org/GRAC/LigandDisplayForward?ligandId=6852, http://www.guidetopharmacology.org/GRAC/LigandDisplayForward?ligandId=7489, http://www.guidetopharmacology.org/GRAC/LigandDisplayForward?ligandId=7496, http://www.guidetopharmacology.org/GRAC/LigandDisplayForward?ligandId=7009 and http://www.guidetopharmacology.org/GRAC/LigandDisplayForward?ligandId=8305. HDACs and HDAC inhibitors currently in development as potential anti‐cancer therapeutics are reviewed by Simó‐Riudalbas and Esteller (2015) [http://www.ncbi.nlm.nih.gov/pubmed/25039449?dopt=AbstractPlus].

**Table:** 

Nomenclature	http://www.guidetopharmacology.org/GRAC/ObjectDisplayForward?objectId=2618
HGNC, UniProt	https://www.genenames.org/data/gene‐symbol‐report/#!/hgnc_id/HGNC:14064, http://www.uniprot.org/uniprot/Q9UBN7
EC number	http://www.genome.jp/dbget‐bin/www_bget?ec:3.5.1.98
Inhibitors	http://www.guidetopharmacology.org/GRAC/LigandDisplayForward?ligandId=7005 (p*K*_i_ 9) [http://www.ncbi.nlm.nih.gov/pubmed/20139990?dopt=AbstractPlus], http://www.guidetopharmacology.org/GRAC/LigandDisplayForward?ligandId=6852 (p*K*_i_ 8.8) [http://www.ncbi.nlm.nih.gov/pubmed/20139990?dopt=AbstractPlus], http://www.guidetopharmacology.org/GRAC/LigandDisplayForward?ligandId=7006 (p*K*_i_ 8) [http://www.ncbi.nlm.nih.gov/pubmed/20139990?dopt=AbstractPlus]
Selective inhibitors	http://www.guidetopharmacology.org/GRAC/LigandDisplayForward?ligandId=7010 (pIC_50_ 8.3) [http://www.ncbi.nlm.nih.gov/pubmed/22262760?dopt=AbstractPlus]

### Further reading on 3.5.1.‐ Histone deacetylases (HDACs)

Ellmeier W *et al*. (2018) Histone deacetylase function in CD4^+^ T cells. *Nat. Rev. Immunol*. **18**: 617‐634 https://www.ncbi.nlm.nih.gov/pubmed/30022149?dopt=AbstractPlus

Maolanon AR *et al*. (2017) Natural and Synthetic Macrocyclic Inhibitors of the Histone Deacetylase Enzymes. *Chembiochem* **18**: 5‐49 https://www.ncbi.nlm.nih.gov/pubmed/27748555?dopt=AbstractPlus

Micelli C *et al*. (2015) Histone deacetylases: structural determinants of inhibitor selectivity. *Drug Discov. Today* **20**: 718‐35 https://www.ncbi.nlm.nih.gov/pubmed/25687212?dopt=AbstractPlus

Millard CJ *et al*. (2017) Targeting Class I Histone Deacetylases in a "Complex" Environment. *Trends Pharmacol. Sci*. **38**: 363‐377 https://www.ncbi.nlm.nih.gov/pubmed/28139258?dopt=AbstractPlus

Roche J *et al*. (2016) Inside HDACs with more selective HDAC inhibitors. *Eur J Med Chem* **121**: 451‐483 https://www.ncbi.nlm.nih.gov/pubmed/27318122?dopt=AbstractPlus

Zagni C *et al*. (2017) The Search for Potent, Small‐Molecule HDACIs in Cancer Treatment: A Decade After Vorinostat. *Med Res Rev* **37**: 1373‐1428 https://www.ncbi.nlm.nih.gov/pubmed/28181261?dopt=AbstractPlus

## http://www.guidetopharmacology.org/GRAC/FamilyDisplayForward?familyId=918

### Overview

In humans, the peptidyl arginine deiminases (PADIs; http://www.genenames.org/cgi‐bin/genefamilies/set/677) are a family of five enzymes, PADI1‐4 and PADI6. PADIs catalyze the deimination of protein L‐arginine residues to L‐citrulline and ammonia, generating peptidyl‐citrulline on histones, fibrinogen, and other biologically relevant proteins. The human isozymes exhibit tissue‐specific expression patterns [http://www.ncbi.nlm.nih.gov/pubmed/12606753?dopt=AbstractPlus]. Overexpression and/or increased PADI activity is observed in several diseases, including rheumatoid arthritis, Alzheimer's disease, multiple sclerosis, lupus, Parkinson's disease, and cancer [http://www.ncbi.nlm.nih.gov/pubmed/23175390?dopt=AbstractPlus]. Pharmacological PADI inhibition reverses protein‐hypercitrullination and disease in mouse models of multiple sclerosis [http://www.ncbi.nlm.nih.gov/pubmed/23118341?dopt=AbstractPlus].

Information on members of this family may be found in the http://www.guidetopharmacology.org/GRAC/FamilyDisplayForward?familyId=918.

### Further reading on 3.5.3.15 Peptidyl arginine deiminases (PADI)

Koushik S *et al*. (2017) PAD4: pathophysiology, current therapeutics and future perspective in rheumatoid arthritis. *Expert Opin. Ther. Targets* **21**: 433‐447 https://www.ncbi.nlm.nih.gov/pubmed/28281906?dopt=AbstractPlus

Tu R *et al*. (2016) Peptidyl Arginine Deiminases and Neurodegenerative Diseases. *Curr. Med. Chem*. **23**: 104‐14 https://www.ncbi.nlm.nih.gov/pubmed/26577926?dopt=AbstractPlus

Whiteley CG. (2014) Arginine metabolising enzymes as targets against Alzheimers’ disease. *Neurochem. Int*. **67**: 23‐31 https://www.ncbi.nlm.nih.gov/pubmed/24508404?dopt=AbstractPlus

## http://www.guidetopharmacology.org/GRAC/FamilyDisplayForward?familyId=896

### Overview

small G‐proteins, are a family of hydrolase enzymes that can bind and hydrolyze guanosine triphosphate (GTP). They are a type of G‐protein found in the cytosol that are homologous to the alpha subunit of heterotrimeric G‐proteins, but unlike the alpha subunit of G proteins, a small GTPase can function independently as a hydrolase enzyme to bind to and hydrolyze a guanosine triphosphate (GTP) to form guanosine diphosphate (GDP). The best‐known members are the Ras GTPases and hence they are sometimes called Ras subfamily GTPases.

## http://www.guidetopharmacology.org/GRAC/FamilyDisplayForward?familyId=897

### Overview

The RAS proteins (HRAS, NRAS and KRAS) are small membrane‐localised G protein‐likemolecules of 21 kd. They act as an on/off switch linking receptor and non‐receptor tyrosine kinase activation to downstream cytoplasmic or nuclear events. Binding of GTP activates the switch, and hydrolysis of the GTP to GDP inactivates the switch.

The RAS proto‐oncogenes are the most frequently mutated class of proteins in human cancers. Common mutations compromise the GTP‐hydrolysing ability of the proteins causing constitutive activation [http://www.ncbi.nlm.nih.gov/pubmed/7900159?dopt=AbstractPlus], which leads to increased cell proliferation and decreased apoptosis [http://www.ncbi.nlm.nih.gov/pubmed/17721087?dopt=AbstractPlus]. Because of their importance in oncogenic transformation these proteins have become the targets of intense drug discovery effort [http://www.ncbi.nlm.nih.gov/pubmed/22004085?dopt=AbstractPlus].

Information on members of this family may be found in the http://www.guidetopharmacology.org/GRAC/FamilyDisplayForward?familyId=897.

### Further reading on RAS subfamily

Dorard C *et al*. (2017) Deciphering the RAS/ERK pathway *in vivo*. *Biochem. Soc. Trans*. **45**: 27‐36 https://www.ncbi.nlm.nih.gov/pubmed/28202657?dopt=AbstractPlus

Keeton AB *et al*. (2017) The RAS‐Effector Interaction as a Drug Target. *Cancer Res*. **77**: 221‐226 https://www.ncbi.nlm.nih.gov/pubmed/28062402?dopt=AbstractPlus

Lu S *et al*. (2016) Ras Conformational Ensembles, Allostery, and Signaling. *Chem. Rev*. **116**: 6607‐65 https://www.ncbi.nlm.nih.gov/pubmed/26815308?dopt=AbstractPlus

Ostrem JM *et al*. (2016) Direct small‐molecule inhibitors of KRAS: from structural insights to mechanism‐based design. *Nat Rev Drug Discov* **15**: 771‐785 https://www.ncbi.nlm.nih.gov/pubmed/27469033?dopt=AbstractPlus

Papke B *et al*. (2017) Drugging RAS: Know the enemy. *Science* **355**: 1158‐1163 https://www.ncbi.nlm.nih.gov/pubmed/28302824?dopt=AbstractPlus

Quah SY *et al*. (2016) Pharmacological modulation of oncogenic Ras by natural products and their derivatives: Renewed hope in the discovery of novel anti‐Ras drugs. *Pharmacol. Ther*. **162**: 35‐57 https://www.ncbi.nlm.nih.gov/pubmed/27016467?dopt=AbstractPlus

Simanshu DK *et al*. (2017) RAS Proteins and Their Regulators in Human Disease. *Cell* **170**: 17‐33 https://www.ncbi.nlm.nih.gov/pubmed/28666118?dopt=AbstractPlus

## http://www.guidetopharmacology.org/GRAC/FamilyDisplayForward?familyId=938

### Overview

The Rab family of proteins is a member of the Ras superfamily of monomeric G proteins. Rab GTPases regulate many steps of membrane traffic, including vesicle formation, vesicle movement along actin and tubulin networks, and membrane fusion. These processes make up the route through which cell surface proteins are trafficked from the Golgi to the plasma membrane and are recycled. Surface protein recycling returns proteins to the surface whose function involves carrying another protein or substance inside the cell, such as the transferrin receptor, or serves as a means of regulating the number of a certain type of protein molecules on the surface ( see http://www.genenames.org/cgi‐bin/genefamilies/set/388, http://www.genenames.org/cgi‐bin/genefamilies/set/388 ).

Information on members of this family may be found in the http://www.guidetopharmacology.org/GRAC/FamilyDisplayForward?familyId=938.
